# Complex intron generation in the yeast genus *Lipomyces*

**DOI:** 10.1038/s41598-020-63239-6

**Published:** 2020-04-07

**Authors:** Norbert Ág, Napsugár Kavalecz, Fruzsina Pénzes, Levente Karaffa, Claudio Scazzocchio, Michel Flipphi, Erzsébet Fekete

**Affiliations:** 1 Dept. of Biochemical Engineering, Faculty of Science and Technology, University of Debrecen, Debrecen, 4032 Hungary; 2 Juhász-Nagy Pál Doctoral School of Biology and Environmental Sciences, University of Debrecen, Debrecen, 4032 Hungary; 3 Dept. of Microbiology, Imperial College London, SW7 2AZ London, UK; 4 Institut de Biologie Intégrative de la Cellule, Centre National de la Recherche Scientifique – Unité Mixte de Recherche UMR 9198, Gif-sur-Yvette, 91190 France

**Keywords:** Genome informatics, RNA splicing

## Abstract

In primary transcripts of eukaryotic nuclear genes, coding sequences are often interrupted by U2-type introns. Such intervening sequences can constitute complex introns excised by consecutive splicing reactions. The origin of spliceosomal introns is a vexing problem. Sequence variation existent across fungal taxa provides means to study their structure and evolution. In one class of complex introns called [D] stwintrons, an (internal) U2 intron is nested within the 5'-donor element of another (external) U2 intron. In the gene for a reticulon-like protein in species of the ascomycete yeast genus *Lipomyces*, the most 5' terminal intron position is occupied by one of three complex intervening sequences consistent of differently nested U2 intron units, as demonstrated in *L. lipofer*, *L. suomiensis*, and *L. starkeyi*. In *L. starkeyi*, the donor elements of the constituent introns are abutting and the complex intervening sequence can be excised alternatively either with one standard splicing reaction or, as a [D] stwintron, by two consecutive reactions. Our work suggests how [D] stwintrons could emerge by the appearance of new functional splice sites within an extant intron. The stepwise stwintronisation mechanism may involve duplication of the functional intron donor element of the ancestor intron.

## Introduction

In the eukaryote nuclear genome, coding sequences are often interspersed with intervening sequences (introns). Introns have to be precisely excised from the primary transcript to generate the open reading frame that translates into the appropriate peptide. Excision of U2-type introns and splicing of the bordering exons is catalysed by the major spliceosome^1–3^, a large supramolecular organelle within the nucleus. (NB. Few U12-type introns are excised by the minor spliceosome). Differently from spliceosomal introns, group I and group II introns are ribozymes, which may or not need accessory proteins for self splicing. In eukaryotes, group I and group II introns are primarily restricted to mitochondrial and chloroplast DNAs.

**Figure 1:**
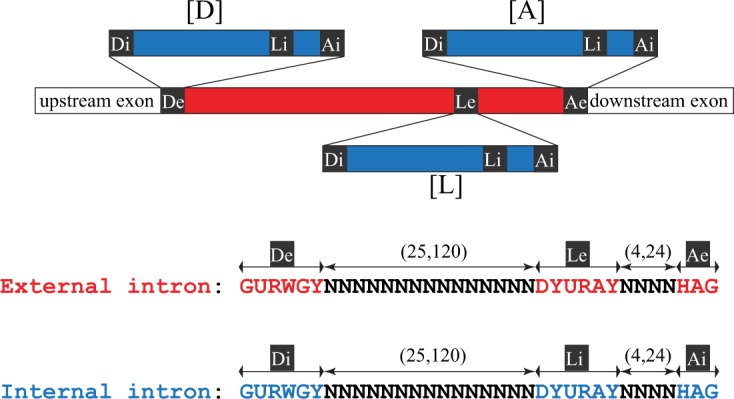
Definition of stwintrons: The tree classes of stwintron (spliceosomal twin intron). Stwintrons are complex intervening sequences consistent of nested U2 introns. In a [D] stwintron, the internal intron is nested within the 5' donor element (consensus sequence: 5'-GURWGY) of the external intron. In a [L] stwintron, the lariat branch point sequence element (consensus sequence: 5'-DYURAY) of the external intron is disrupted, while in an [A] stwintron, it interrupts the 3' acceptor (consensus sequence: 5'-HAG). Consequently, excision of the internal intron is indispensable for the subsequent excision of the external intron. The external intron is shown as a red bar and the internal intron as a blue bar. The three conserved intronic sequence elements essential for U2 splicing are indicated as black boxes labelled as “D”, “L” or “A”: external and internal elements are labelled by “e” and “i”, respectively. Underneath, consensus sequences of the 5'-donor, the lariat branch point element and the 3'-acceptor in fungi^18^ and their relative positions in the internal and external U2 introns are depicted.

Intervening sequences of any of the three main classes – group I, group II and spliceosomal – can be made up of more than one functional intron unit^4–6^. Previously, we have described a particular class of nested U2 introns, the stwintron, for which the excision of the internal intron is required to accomplish the subsequent excision of the external intron because the former interrupts one of the three conserved intron splicing elements of the latter, the 5'-donor, the sequence element around the lariat branch point adenosine, or the 3'-acceptor^7–11^ (Fig. 1). We named stwintron types according to where the insertion of the internal intron occurred – ‘D', indicating insertions within the donor sequence, ‘L’, within the conserved sequence element around the lariat branch point adenosine, and ‘A’, within the acceptor – followed by the numbers of the successive nucleotides (nt) in the consensus sequence separated by the internal intron. To date, we have demonstrated the existence of [D1,2], [D2,3] and [D5,6] stwintrons (i.e. internal intron situated between the first and the second nt, the second and the third nt, and the fifth and the sixth nt of the donor of the external intron, respectively) and an [A2,3] stwintron (i.e. internal intron splitting the acceptor of the external intron between its second and third nt). We defined stwintrons as the spliceosomal analogues of those original group II/III twin introns (twintrons) in the *Euglena gracilis* plastid genome^12,13^, in which the internal intron disrupts a sequence element essential for the excision of the external intron. (NB. Group III introns are abbreviated versions of group II introns). Correct stwintron excision requires intron definition whereby the nearest 5’- and 3'-splice sites are paired across the intron prior to splicing^14–16^. This results in excision of the smallest possible U2 intron; in the case of a stwintron, this is thus the internal intron. In fungi, spliceosomal introns are generally quite small – often <100 nucleotide (nt). Nevertheless, one of the fungal stwintrons we have described is crucial to an authentic alternative splicing event^11^.

**Figure 2:**
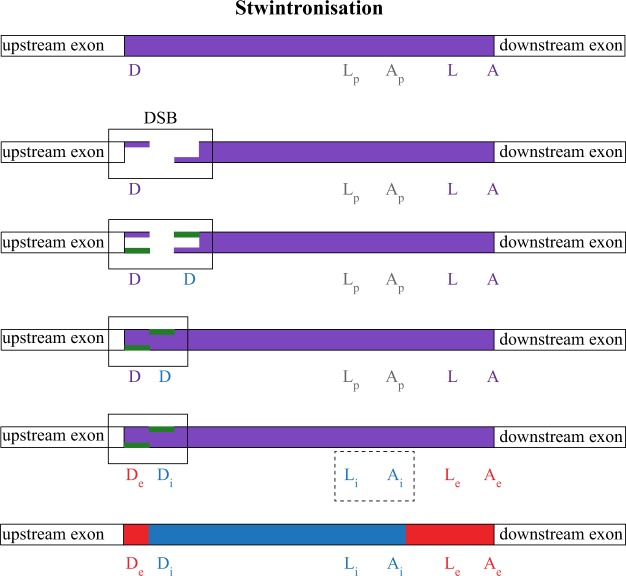
The stwintronisation mechanism of complex intron generation. Scheme of events that result in the stepwise formation of new functional 5'- and 3'-splice sites within an extant U2 intron –stwintronisation – illustrated for the emergence of a [D] stwintron. The original host intron is of a length such that it can be split into two functional U2 introns and is indicated by the purple bar. Duplication of functional donor elements may result from the repair of an asymmetric DNA double-strand break (DSB) leaving small 3' overhangs. These overhangs are filled in (green) by polymerase activity associated with the canonical nonhomologous DNA end-joining (cNHEJ) machinery before blunt end ligation. In a separate event, precursor sequences (“p”) resembling lariat branch point (L) and associated acceptor (A) elements in the middle of the host intron morph into functional 3' splice elements (“i”) by mutation. The newly formed 5'-donor- and lariat branch point elements allow definition of a new internal intron disrupting the functional donor element of the new external intron. Donor duplication and generation of functional 3' consensus sites are independent events and can take place in any order.

Two [D] stwintrons conserved across whole classes of filamentous fungi are located at exactly the same intron position as a canonical U2 intron present in early divergent *Pezizomycotina* taxa^8^. One possible mechanism by which such a stwintron could emerge involves the stepwise formation of new 5'- and 3' splice sites within an extant intron of sufficient size to evolve into two U2 introns (Fig. 2). We propose to call this mechanism “stwintronisation” by analogy with intronisation, the formation of intronic sequences from exonic sequences^17^. The genesis of a new 5' splice site within or next to the donor element of the host intron is necessary for the formation of a [D] stwintron. A small duplication including part or the entirety of the canonical six-nt donor element (consensus sequence: 5'-GURWGY)^18^ could occur during repair of an asymmetric DNA double-strand break (DSB) which would leave a 3' overhang. In an independent event, mutations in the central region of the host intron could result in functional 3'-splice sites. The new internal splice sites may pair to define the new U2 unit, whose subsequent excision is necessary for the formation of a functional donor for the second U2 definition and standard splicing reaction to properly remove all intervening sequences.

In a previous paper^11^, we identified a [D5,6] stwintron in the gene encoding a reticulon-like protein in the *Pezizomycotina*, one of the three *Ascomycota* subphyla. The gene product is a “protein of unknown function which associates with the endoplasmic reticulum” (protein family PF02453). While assessing the ancestry of this [D5,6] stwintron, we encountered different complex intervening sequences (CIS) consistent of nested U2 introns in the orthologous gene in seven species of *Lipomyces*, a taxon of the *Saccharomycotina*, another subphylum of the *Ascomycota*. The parallel evolution of these three different CIS at the very same intron position in species of the same fungal genus supports the stwintronisation mechanism of stwintron evolution.

## Results

### Evolution of the intron-exon structure of the transcript of the genes for a reticulon-like protein in the genus *Lipomyces*

**Figure 3:**
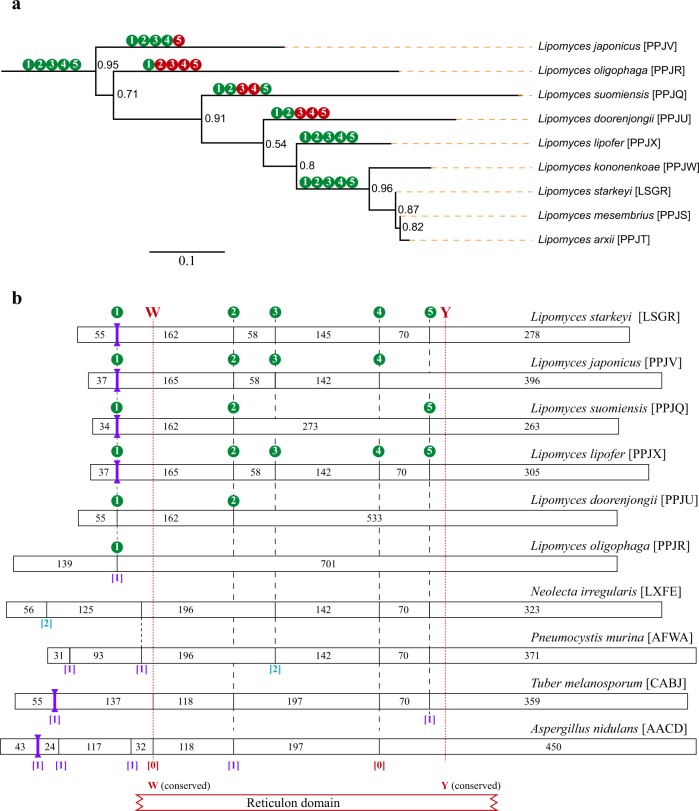
Evolutionary relations between *Lipomyces* species and the intron-exon structure of their genes for the reticulon-like protein. **(a)** Monophyletic clade of the *Lipomyces* genus drawn from an extensive maximum likelihood tree, inferred from a trimmed alignment of 902 ascomycete reticulon-like proteins. Branch support is estimated with approximate likelihood-ratio tests for branches^32^. The scale bar gives the evolutionary distance of 0.1 amino acid substitutions per site. The circles along some branches indicate the presence (green) or absence (red) of introns at five conserved positions in the primary transcript(s) of the species at the end of that branch. Species names are labelled with the unique four-letter combination of their respective Whole Genome Shotgun Master Accessions between square brackets. **(b)** Schematic representation of deduced gene models of the genes for the reticulon-like protein in *Lipomyces* species compared with selected species of *Ascomycota*. The size of each exon is given in nucleotides (nt): for the first exon with coding sequences, the size is given from the start codon to the first intron and for the last exon with coding sequences, from the last intron to the stop codon. The intron positions between the exons are given by the uninterrupted vertical lines in the gene bars. The phase of the introns is given underneath between square brackets: phase zero in red, phase one in purple and phase two in cyan. CIS are represented by thick purple vertical lines. The sequences encoding the conserved PF02453 domain are roughly indicated by the red lined bar underneath the gene models; the strictly conserved Trp (W_73_ in *A. nidulans*) and Tyr (Y_212_ in *A. nidulans*) residues are situated near the N-terminus and C-terminus of the PF02453 domain, respectively. The introns at conserved positions 2–5 are connected by dashed lines between the gene bars.

The conservation of intron positions in multiple species of *Taphrinomycotina* and in the family of the *Lipomycetaceae (Saccharomycotina)* is consistent with their reticulon-like genes being genuine orthologues of the one in *Aspergillus nidulans (rtnA)* and in other *Pezizomycotina*^11^. Figure 3a shows the monophyletic cluster of the *Lipomyces* genus (nine species) drawn from an extensive maximum likelihood tree of the reticulon-like protein in all *Ascomycota* (see Methods section for details). *Lipomycetaceae* occupy a unique phylogenetic position at the basis of the *Saccharomycotina* clade^19^. The species *Lipomyces starkeyi* harbours many more ORF-interrupting introns than ascomycete yeasts outside the *Lipomycetaceae*^20^. The intron density of *L. starkeyi* resembles that seen in *Pezizomycotina* rather than that apparent in other *Saccharomycotina*.

Exceptionally within the *Saccharomycotina*, the primary transcript of the gene for the reticulon-like protein harbours multiple introns in eight genome-sequenced *Lipomyces*. However, the intron content varies considerably from the maximum of five introns in *L. starkeyi* and three closely related species, as well as in *L. lipofer* (Fig. 3b). *L. japonicus* lacks the most 3' of these five introns while in *L. suomiensis*, the intron positions 3 and 4 are not occupied. Furthermore, *L. doorenjongii* only carries introns at the first two positions and *L. oligophaga* harbours one lone intron corresponding with the most 5' intron in the eight other species. The four most 3' introns in *L. starkeyi* interrupt the DNA coding for the conserved PF02453 domain and are positionally conserved in either species of *Pezizomycotina* (position 2), or species of *Taphrinomycotina* (position 3), or species from both these other subphyla (positions 4 and 5). The observed simplification of the intron-exon structure of the gene encoding the reticulon-like protein suggests that some early divergent *Lipomyces* species have lost introns rather than that new introns have been gained in the other taxa, completely independent of the massive intron loss observed in all other families of *Saccharomycotina*^21^.

### Identification of different complex intervening sequences (CIS) at the same intron position in the gene for the reticulon-like protein

**Figure 4:**
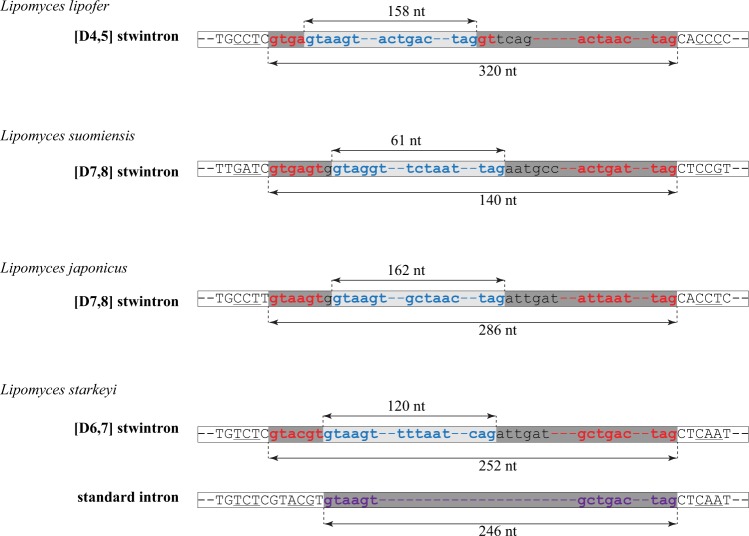
The three complex intervening sequences [D4,5], [D6,7], [D7,8] in the transcript of the gene coding for the reticulon-like protein of selected *Lipomyces* species schematically aligned at their splice sites. The alternative splice option in *L. starkeyi*, excision of one large canonical U2 intron instead of the [D6,7] stwintron, is also depicted. The last uninterrupted codon up- and the first uninterrupted codon downstream the intervening sequences is underlined. Intronic sequences are in lower case letter. The sequences of the three conserved intronic sequence elements, the 5' donor (D), the lariat branch point element (L) and the 3' acceptor (A), are color coded: Those of the internal introns are in blue, those of the external introns are in red and those of the large alternatively spliced canonical U2 intron (*L. starkeyi*) are in purple. For convenience, the underlying internal introns are highlighted by the light grey background and the external introns by the dark grey background. The exact sizes of the internal introns and the complex intervening sequences are given for each species.

The first intervening sequence in the primary transcript of the reticulon-like gene is at a conserved position, unique to the nine *Lipomyces* species. The second exon is either 162 or 165 nt (*L. japonicus* & *L. lipofer*) long. The extra codon is located upstream of the codon of the absolutely conserved Trp of the PF02453 domain (Fig. 3b), within the DNA encoding the N-terminal extension of the reticulon-like protein. In *L. oligophaga* and *L. doorenjongii*, the intervening sequences at the most 5' intron position are short canonical U2 introns, respectively 78 and 46 nt in length. However, in the other seven species, the corresponding sequences are considerably longer – from 140 nt in *L. suomiensis* to 320 nt in *L. lipofer* – and in each case, appear to consist of two nested canonical U2 introns. We have detected three modes of organisation of the CIS at the first intron position in the gene for the reticulon-like protein in different *Lipomyces* species, each with the putative internal U2 intron disrupting the putative external U2 intron near the 5' extremity of the latter at a slightly different position. This is illustrated in Fig. 4. We have experimentally verified the predicted internal introns in three representative species by targeted RT-PCR of the respective stwintron splicing intermediates and subsequent cDNA sequencing (see Methods section for details).

**Figure 5:**
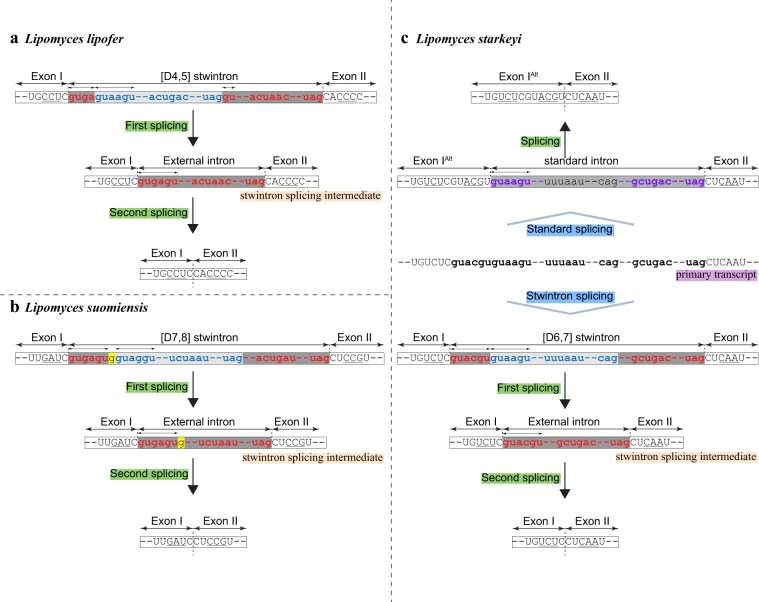
Splicing schemes for the different complex intervening sequences (CIS) in the orthologue gene coding for the reticulon-like protein in three *Lipomyces* species. **(a)** Schematic representation of the structure of the *L. lipofer* [D4,5] stwintron and its two-step excision. Intronic sequences are in lower case letters. The internal intron is represented by the light grey bar and its 5′-donor-, lariat branch point domain- and 3′-acceptor sequences are in blue lettering. The external intron is marked by the darker grey bar; its 5′-donor-, lariat branch point domain- and 3′-acceptor sequences are in red lettering. Note that the internal intron is nested in the donor element of the external intron between a_4_ and g_5_. **(b)** The structure of the *L. suomiensis* [D7,8] CIS (stwintron *sensu lato*) and its two-step excision. The internal intron is nested in the external intron between g_7_ and a_8_. The nt between the two functional 5'-donor elements (g_7_) is highlighted by the yellow background. **(c)** The structure of the *L. starkeyi* [D6,7] CIS (stwintron *sensu lato*) and its two-step excision (from the primary transcript in the centre of the Panel downwards). The internal intron is nested in the external intron between u_6_ and a_7_. Alternatively, the intervening sequence is excised by one standard splicing reaction (from the primary transcript in the centre of the Panel upwards). The large canonical U2 intron is marked by a darker grey bar; its 5′-donor-, lariat branch point domain- and 3′-acceptor sequences are in purple lettering. Note that the most 5' of the abutting donor sequences (5'-GUACGU) is exonic when the transcript is alternatively spliced with one standard splicing reaction. As a consequence, the upstream exon (Exon I ^ALT^) is six nt longer.

In *L. lipofer*, the CIS is 320 nt long. An 158-nt long internal intron (5' GUAAGU – 131-nt – ACUGAC – 12-nt – UAG) is disrupting the 5'-donor element of an 162-nt long external intron (5' GUGA | GU – 137-nt – ACUAAC – 10-nt – UAG) between A_4_ and G_5_. The splicing scheme is shown in Fig. 5a. The experimentally determined sequence of the splicing intermediate of this [D4,5] stwintron, lacking the predicted internal intron and featuring the uninterrupted 5' donor of the predicted external intron, is shown in Supplementary Fig. S1. Determined sequences of the mature mRNA and the stwintron splicing intermediate were deposited at GenBank under accession numbers MN689083 and MN689084, respectively. This *L. lipofer* stwintron is the first donor-disrupted stwintron of the [D4,5] type as well as the first ever stwintron (*sensu stricto*) described outside the *Pezizomycotina* subphylum.

In two species, *L. suomiensis* and *L. japonicus*, we detected two potential canonical 5' donor sequences separated by one nt at the 5' extremity of the predicted CIS. Furthermore, a fully canonical 3'-acceptor and associated lariat branch point element could be identified within the CIS in both species. Sequence analysis of the mature messenger in *L. suomiensis* (GenBank MN689081 & Fig. 5b) showed that the most 5' of these donor elements (5'-GUGAGU) serves at the ultimate 5' splice site of the whole 140 nt-long CIS. Subsequent analysis of the splicing intermediate (GenBank MN689082 & Supplementary Fig. S2) confirmed that the 3' donor copy (5'-GUAGGU) is used to excise an 61-nt long internal U2 intron (5' GUAGGU –38-nt – UCUAAC – 8-nt – UAG) nested in a 79-nt long canonical external U2 intron (5' GUGAGUG | A – 45-nt – ACUGAC – 17-nt – UAG) between G_7_ and A_8_ (Fig. 5b illustrates the splicing scheme in *L. suomiensis*). Similarly, in *L. japonicus*, we predict that an 162-nt long canonical U2 intron (5' GUAAGU – 135-nt – ACUAAC – 12-nt – UAG) is disrupting an 124-nt long canonical U2 intron (5' GUAAGUG | A – 97-nt – AUUAAU – 10-nt – UAG) between G_7_ and A_8_. The whole CIS is 286 nt long and the two functional donor elements are identical in sequence (5'-GUAAGU) (see Fig. 4 for *L. japonicus*). A difference between the two species is that the constituting U2 introns in *L. suomiensis* are half the size of those in *L. japonicus*.

To excise these [D7,8] CIS, two standard splicing reactions are necessary. This intron nesting does not constitute a [D] stwintron in *sensu stricto* as previously defined^7^, since the internal intron does not interrupt the donor element of the external intron; the two canonical donor elements are actually one nt apart from each other. In the primary transcript of the gene for the reticulon-like protein in *L. suomiensis*, the primary selected 5'- and 3'-splice sites are clearly those that define the smallest possible internal intron (intron definition). We can therefore consider such CIS of nested introns examples of a new class of fungal stwintron, the “stwintron *sensu lato*”.

In *L. starkeyi* and the three closely allied species (Fig. 3a) we detected two abutting potential 5' donor elements at the 5' extremity of the predicted CIS in the gene for the reticulon-like protein, a second instance of stwintron *sensu lato*, [D6,7] (Fig. 5c and Supplementary Fig. S3). The most 3' of the donor elements has the ideal consensus sequence (5'-GUAAGU) while the one at 5' (5'-GUACGU) is somewhat less canonical. Given the position of the intervening sequence within the DNA/RNA coding for the poorly conserved N-terminal extension of the reticulon-like protein, we first tested in *L. starkeyi* (strain CCY 33-1-1: gene sequence, GenBank MN689085) whether the intervening sequence could be alternatively excised using either the one or the other donor element. Amongst the cDNA clones of matured mRNA generated, we indeed found two different ORFs, one of them containing six nt (GTACGT) more than the other (GenBank MN689086 & MN689087), demonstrating alternative splicing. Under our experimental conditions, fewer cDNA clones contained the GTACGT sequence. Subsequent inspection of the intervening sequence suggested the presence of a coupled lariat branch point element and 3'-acceptor roughly in its centre, of which the lariat branch point element could be the imperfect 5'-AUUCAU or the equally imperfect 5'-UUUAAU, associated with a 5'- CAG shortly downstream. If the internal splice signals were recognised by the spliceosome, one would expect a complex [D6,7] intervening sequence of 252 nt, where an 120-nt long internal U2 intron (5' GUAAGU – 75-nt – AUUCAU – 14-nt – UUUAAU – 10-nt – CAG) is disrupting an 132-nt long external intron (5' GUACGU | A – 109-nt – GCUGAC – 7-nt – UAG) between U_6_ and A_7_ (see Fig. 5c for the splicing scheme and Supplementary Fig. S3). We sequenced the expected stwintron splicing intermediates in the transcripts of the genes coding for the reticulon-like protein in *L. starkeyi* CCY 33-1-1 and *L. kononenkoae* (GenBank MN689088–MN689090). However, if the spliceosome would ignore the imperfect internal 3' splice site halfway the intervening sequence but not the fully canonical proximal donor, one would expect that a 246-nt long canonical U2 intron (5' GUAAGU – 224-nt – GCUGAC – 7-nt – UAG) is excised in one standard splicing reaction (Fig. 5c: Alternative splicing option), giving rise to the 6-nt longer mRNA species (i.e., GenBank MN689086).

In *L. starkeyi*, a [D6,7] stwintron *sensu lato*, characterised by abutting 5' donor elements, is functionally implicated in alternative splicing. The stwintron alternative employs both donor elements in subsequent splicing reactions, the first of which uses the proximal, ideal consensus 5'-donor paired with the imperfect 3'-splice site halfway the CIS instead of the canonical 3' splice site at its end. The U2 spliceosome thus does not “choose” between directly neighbouring functional 5'-donors but rather between available lariat branch point elements situated at much longer distance from one another, of which the more proximal one is, crucially, aberrant.

## Discussion

In the genes for the reticulon-like protein of genome-sequenced species of the *Lipomycetaceae*, the earliest divergent family of the *Saccharomycotina* subphylum, we identified and experimentally confirmed three different formats of intron nesting in the most 5' intervening sequence, and therewith three new types of stwintron. Like for the [D5,6] stwintron in *Pezizomycotina*^11^, the phase of the respective external introns is one and the [D4,5]-, [D6,7]- and [D7,8] stwintrons are located in the DNA/RNA encoding the intrinsically disordered N-terminal extension of the reticulon domain (PF02453), which is poorly conserved both in length and in sequence in ascomycete reticulon-like proteins.

The origin of spliceosomal introns is a vexing problem. Some commonly purported mechanisms involve mobile elements like transposons or group II introns of mitochondrial or bacterial origin, while other proposed mechanisms (tandem duplication, gene conversion or intronisation) are autonomous and have a strictly endogenous origin^22^. Some modes of autonomous intron gain could involve repair of double-strand breaks (DSB) by an end-joining mechanism^23–26^. The formation of a new stwintron from the sequences of an existing canonical intron can occur similar to the intronisation mechanism^17^, by the formation of new 5'- and 3'-splice sites within the parental intron at the chromosomal locus (see Fig. 2 for a schematic overview). The three position-conserved CIS in seven *Lipomyces* primary transcripts of the gene encoding the reticulon-like protein each constitute two differently nested canonical U2 introns. Crucially, in *L. starkeyi*, the CIS can also be removed in one standard splicing reaction, which suggests that the current *Lipomyces* [D4,5]-, [D6,7]-, and [D7,8] stwintrons found in other species have evolved by stwintronisation of one long primordial intron. An aligned representation (Fig. 4) suggests that the donor of the original intron has been duplicated – entirely or in part – to generate the 5' splice site of the new internal intron. Such small tandem duplications (4–7 nt) may well be the result of repair of asymmetric double-strand breaks with a limited 3' overhang by canonical nonhomologous end-joining (cNHEJ)^27^. However, we cannot exclude the possibility that formation of one or more of the three different stwintrons involved point mutations near the 5' end of the parental canonical intron to create the donor of the new internal intron, rather than a small duplication. Secondary mutations within the parental intron are still necessary to generate functional 3' splice elements for the internal intron of the new stwintron. Where stwintronisation appears completed in *L. lipofer*, *L. suomiensis* and *L. japonicus*, the situation in *L. starkeyi* ([D6,7]) could represent an intermediate stage in which the CIS can be removed by one- as well as by two subsequent U2 splicing reactions, as the internal imperfect 3' lariat branch point element is apparently functionally weak.

Natural sequence variation existent across a fungal phylum provides means to study intron structure and evolution. In this work, we describe stwintrons *sensu lato*, in which the internal intron is ***not*** nested within a conserved intronic sequence element essential for the excision of the external intron, but for which the constituent introns are still excised in “inside out” order by consecutive standard splicing reactions. The occurrence of three different stwintrons at a strictly conserved intron position in seven species of *Lipomyces*, one of which embodies an authentic alternative splice option, provides an insight into an evolutionary mechanism by which complex intervening sequences consistent of nested intron units may emerge. We have called this stepwise mechanism stwintronisation, where new functional 5' and 3' splice sites evolve within a pre-extant canonical intron.

## Methods

### Mining fungal genes for the reticulon-like protein

In an effort to probe the ancestry of the *Pezizomycotina* [D5,6] stwintron in the gene for the fungal reticulon-like protein, we searched the DNA databases at the National Center of Biotechnology Information (NCBI) for homologous genes in other fungi. Additional homologues were found in the *Saccharomycotina* and *Taphrinomycotina* subphyla of the *Ascomycota*, the *Blastocladiomycota*, the *Glomeromycota*, the *Mucoromycotina* and the *Mortierellomycotina* (Supplementary Table S1). Although most genome-sequenced ascomycete yeasts have intronless genes for the reticulon-like protein (Rtn), quite some taxa, including species from the *Cephaloascaceae*, *Alloascoideaceae*, *Pichiaceae* and *Debaryomycetaceae* families, retained one standard intron at the position corresponding to the fifth intron in the orthologous *Aspergillus nidulans rtnA* gene^11^. This position conservation suggests that the *Ascomycota* genes are phylogenetically related. Multiple genes were often encountered in *Mucoromycotina* and *Mortierellomycotina*, but we could not find [D5,6] stwintrons beyond the *Pezizomycotina* subphylum of the *Ascomycota*.

Species of *Lipomyces* are the only *Saccharomycotina* that harbour multiple introns in their orthologous genes for the reticulon-like protein. Of these, the second intron occurs at the position of the fifth *A. nidulans* intron. We used all Rtn proteins we deduced from the encoding genes in *Ascomycota* to infer maximum likelihood trees (not shown), to verify the status of the *Lipomycetaceae* as the earliest divergent family of *Saccharomycotina*^19,20^. Most of these analyses yielded monophyletic clades for the nine sequenced *Lipomyces*: A representative maximum likelihood subtree is shown in Fig. 3a. The used *Lipomyces* genome sequence resources are: *L. starkeyi* (GenBank Whole Genome Shotgun Master accession: LSGR00000000.1^20^), *L. mesembrius* (PPJS00000000.2^19^), *L. arxii* (PPJT00000000.2^19^), *L. kononenkoae* (PPJW00000000.1^19^), *L. lipofer* (PPJX00000000.1^19^), *L. doorenjongii* (PPJU00000000.1^19^), *L. suomiensis* (PPJQ00000000.2^19^), *L. oligophaga* (PPJR00000000.2^19^) and *L. japonicus* (PPJV00000000.1^19^).

### Maximum likelihood phylogenetic analysis of ascomycete reticulon-like proteins

The phylogenetic tree at the basis of the monophyletic *Lipomyces* clade shown in Fig. 3, was inferred from a trimmed multiple sequence alignment of 902 putative reticulon-like proteins. The proteins were first aligned with MAFFT (Multiple Alignment using Fast Fourier Transform; version 7)^28^, applying the L-INS-i iterative refinement method trained on recognising one conserved domain with long extensions – appropriate for the PF02453 domain – with the BLOSUM80 scoring matrix. This MAFFT alignment was trimmed to 136 informative residues with Block Mapping and Gathering with Entropy software (BMGE version 1.12)^29^, using the BLOSUM40 similarity matrix with the block size set at 4. A maximum likelihood tree was then calculated from the trimmed alignment by PhyML (version 3.0) with automatic substitution model selection^30^ in default mode. The Smart Model Selection (SMS) software selected LG + G + I + F as the substitution model^31^ using the Akaike Information Criterion. Branch support was estimated by approximate likelihood-ratio tests^32^ implemented by the PhyML webserver. The inferred tree was drawn from the PhyML Newick output with FigTree (version 1.4.3: http://tree.bio.ed.ac.uk/software/figtree). The *Lipomyces* subtree was selected in FigTree (select clade option) and saved in a separate file, then exported as an interactive pdf. Final annotation was done in Adobe Illustrator.

### *Lipomyces* strains, maintenance and cultivation conditions

*Lipomyces lipofer* CBS 944 (National Collection of Agricultural and Industrial Microorganisms, Budapest, Hungary: NCAIM Y.00351), *Lipomyces suomiensis* CBS 7251 (Westerdijk Fungal Biodiversity Institute, Royal Netherlands Academy of Sciences, Utrecht, The Netherlands), and *Lipomyces starkeyi* CCY 33-1-1 (Dept. of Microbiology and Biotechnology, Corvinus University of Budapest, Hungary) were used to verify the existence of a particular CIS in the primary transcript of the orthologous genes encoding a reticulon-like protein. *Lipomyces kononenkoae* CBS 2514 was also assessed in support of results obtained for *L. starkeyi*. All yeasts were maintained and cultivated on rich growth media listed in Supplementary Table S2 at 26 °C, as recommended by the respective stock centers supplying the reference materials.

For isolation of nucleic acids, yeast cultures were grown in 500-mL Erlenmeyer flasks with 100 mL medium in an orbital shaker (Infors HT Multitron) at 200 revolutions min^–1^ (rpm) for 16 h. The biomass was harvested by centrifugation at 10,000 rpm for 5 min then transferred to tubes with ceramic beads (MagNA Lyser Green Beads; Roche) and homogenised at 4000 rpm for 30 s with the help of a MagNA Lyser Instrument (Roche). Macherey-Nagel NucleoSpin kits were used for the extraction of genomic DNA (NucleoSpin Plant II) and total RNA (NucleoSpin RNA Plant) from the homogenate. Nucleic acids were quantified using NanoDrop technology (Thermo Scientific).

### Reverse transcription and polymerase chain reaction (RT-PCR) verification of the intron-exon structures, mature RNAs and stwintron splicing intermediates

Reverse transcription was primed off 1 μg of total RNA with Oligo(dT) as the primer using the First Strand cDNA Synthesis Kit (Thermo Scientific). PCR reactions were performed in a 25 μL volume containing 4 μL of single strand cDNA, using gene-specific oligonucleotides (Supplementary Table S3) as primers and DreamTaq DNA Polymerase (Thermo Scientific). Cycling conditions after initial denaturation at 95 °C (2 min) were: 40 cycles of 95 °C for 30 s, 56 °C for 1 min, and 72 °C for 1 min, followed by one post-cyclic elongation at 72 °C for 5 min. Amplified fragments were resolved in native agarose gels. All RT-PCR experiments were done in triplicate, starting with biomass from independent cultures.

Double-strand cDNA was gel-purified (NucleoSpin Gel & PCR Clean-up, Macherey-Nagel) and cloned (pGEM-T Easy Vector System I, Promega). Plasmid DNA was isolated using the NucleoSpin Plasmid EasyPure kit (Macherey-Nagel). Independent clones were sequenced over both strands using universal primers hybridising to the vector (Eurofins Genomics, Ebersberg, Germany). All sequences determined were deposited at GenBank. For convenience, determined sequences of RNA stwintron splicing intermediates are also given in the Supplementary Information.

### Accession numbers

Accession numbers for sequences determined during this study: MN689081–MN689090.

## Supplementary information

Supplementary Information.

## Data Availability

All datasets generated are included in the manuscript and/or the Supplementary Information. All sequences determined in the course of this study were deposited at GenBank.
