# Supplementary material for: Complex intron generation in the yeast genus Lipomyces
Source: Sci Rep. 2020 Apr 7;10:6022. doi: 10.1038/s41598-020-63239-6 (PMC7138796; doi:10.1038/s41598-020-63239-6)
Supplement: Supplementary file 1 — Supplementary Information. [file 41598_2020_63239_MOESM1_ESM.pdf]

Supplementary information

(Manuscript number: SREP-20-00236; Scientific Reports 2020)

Complex intron generation in the yeast genus *Lipomyces*

Norbert Ág [1], Napsugár Kavalecz [1,2], Fruzsina Pénzes [1,2], Levente Karaffa [1], Claudio Scazzocchio [3,4], Michel Flipphi [1] & Erzsébet Fekete [1,*]

[1] Dept. of Biochemical Engineering, Faculty of Science, University of Debrecen, 4032, Hungary.
[2] Juhász-Nagy Pál Doctoral School of Biology and Environmental Sciences, University of Debrecen, 4032, Hungary.
[3] Dept. of Microbiology, Imperial College London, SW7 2AZ, UK.
[4] Institut de Biologie Intégrative de la Cellule, Centre National de la Recherche Scientifique – Unité Mixte de Recherche UMR 9198, Gif-sur-Yvette, 91190, France.

* To whom correspondence should be addressed. Email: kicsizsoka@yahoo.com

Supplementary Table S1
Supplementary Table S2
Supplementary Table S3
Supplementary Figure S1
Supplementary Figure S2
Supplementary Figure S3
Supplementary Text File 1
Supplementary Text File 2

Supplementary Table S1. Identification of additional orthologous fungal *rtnA* genes (accession; coordinates AUG-STOP) and the stwintron(s) (where appropriate).

	Name	Taxon	Accession number	Coordinates of the gene	Coordinates of the stwintron
1	[Candida] aaseri	Debaryomycetaceae	LKAN01000048	411433-412554	
2	[Candida] ascalaphidarum	Debaryomycetaceae	PPPA02000002	374497-375348	
3	[Candida] athensensis	Debaryomycetaceae	PPIU01000002	1326674-1327616	
4	[Candida] blattae	Metschnikowiaceae	PPMS02000001	849531-850394	
5	[Candida] duobushaemulonis	Metschnikowiaceae	PKFP01000005	202491-203534	
6	[Candida] fragi	Debaryomycetaceae	PPMP01000006	246803-247720	
7	[Candida] glabrata	Saccharomycetaceae	AYJS02000028	234835-235719	
8	[Candida] golubevii	Metschnikowiaceae	PPPB01000029	80967-81821	
9	[Candida] gorgasii	Debaryomycetaceae	PPPC02000004	397059-398104	
10	[Candida] gotoi	Debaryomycetaceae	PPNP01000008	136867-138325	
11	[Candida] haemulonis	Metschnikowiaceae	PKFO01000008	631902-632879	
12	[Candida] hawaiiana	Metschnikowiaceae	NXGY01000010	86756-87661	
13	[Candida] heveicola	Metschnikowiaceae	PPOB01000006	631239-632171	
14	[Candida] intermedia	Metschnikowiaceae	BCGD01000005	735213-736139	
15	[Candida] ipomoeae	Metschnikowiaceae	NLAI01000697	142058-142987	
16	[Candida] oregonensis	Metschnikowiaceae	PPLJ02000006	793085-793894	
17	[Candida] ponderosae	Phaffomycetaceae	PPMG02000021	73000-73815	
18	[Candida] restingae	Metschnikowiaceae	PPJA02000004	594647-595818	
19	[Candida] rhagii	Debaryomycetaceae	PPNO02000002	324038-325003	
20	[Candida] schatavii	Debaryomycetaceae	PPIY01000035	7629-8940	
21	[Candida] stellimalicola	Phaffomycetaceae	PPIC01000026	136718-137653	
22	[Candida] tammaniensis	Debaryomycetaceae	PPOY01000003	1073939-1074796	
23	[Candida] torresii	Metschnikowiaceae	NXHD01000012	284807-285610	
24	[Candida] wancherniae	Metschnikowiaceae	PPOZ02000012	82917-83762	
25	[Kloeckera] hatyaiensis	Saccharomycodaceae	PPNQ01000046	48958-49653	
26	Aciculoconidium aculeatum	Metschnikowiaceae	PPJB01000018	271790-272821	
27	Alloascoidea hylecoeti	Alloascoideaceae	BCKZ01000009	381001-381917	
28	Ambrosiozyma ambrosiae	Pichiaceae	PPHU01000017	315028-316027	
29	Ambrosiozyma kashinagacola	Pichiaceae	BCGA01000005	84250-85264	
30	Ambrosiozyma kashinagacola	Pichiaceae	BCGA01000005	84250-85264	
31	Ambrosiozyma maleeae_1	Pichiaceae	PPLV01000001	498634-499583	
32	Ambrosiozyma maleeae_2	Pichiaceae	PPLV01000001	435605-436462	
33	Ambrosiozyma monospora	Pichiaceae	BCIP01000017	495576-496665	
34	Ambrosiozyma oregonensis	Pichiaceae	PPKY02000004	490167-491263	
35	Ambrosiozyma philentoma	Pichiaceae	NHAR01000352	625915-626964	
36	Ambrosiozyma vanderkliftii	Pichiaceae	PPHW01000018	9002-10152	
37	Ascoidea asiatica	Ascoideaceae	BCKQ01000003	314881-315807	
38	Ascoidea rubescens	Ascoideaceae	LYBR01000032	72831-73649	
39	Ashbya aceri	Saccharomycetaceae	CP006021	210544-211377	
40	Barnettozyma californica	Phaffomycetaceae	PPLX02000013	228932-229846	
41	Barnettozyma hawaiiensis	Phaffomycetaceae	PPLY01000052	5559-6497	
42	Barnettozyma populi	Phaffomycetaceae	PPLZ02000002	389326-390234	
43	Barnettozyma pratensis	Phaffomycetaceae	PPMA01000044	564-1529	
44	Barnettozyma salicaria	Phaffomycetaceae	PPMB01000025	87006-87953	
45	Blastobotrys adeninivorans	Trichomonascaceae	CBZY010000005	1056282-1057472	
46	Blastobotrys americana	Trichomonascaceae	PPJN02000010	132192-133436	
47	Blastobotrys mokoenaii	Trichomonascaceae	PPJM02000008	163174-164157	
48	Blastobotrys muscicola	Trichomonascaceae	PPJL02000030	59544-60536	
49	Blastobotrys peoriensis	Trichomonascaceae	PPJJ02000058	42965-44170	
50	Blastobotrys proliferans	Trichomonascaceae	PPJI02000078	57082-58161	
51	Blastobotrys raffinosifermentans	Trichomonascaceae	PPJH01000004	82736-83980	
52	Blastobotrys serpentis	Trichomonascaceae	PPJG01000055	1537-2574	
53	Brettanomyces anomalus	Pichiaceae	LCTY01000002	1157580-1158419	
54	Brettanomyces bruxellensis	Pichiaceae	AHMD01000276	291476-292315	
55	Brettanomyces custersianus	Pichiaceae	MDVK01000035	19719-20621	
56	Brettanomyces naardenensis	Pichiaceae	MDSA01000008	27799-28629	
57	Candida africana_TSA	Debaryomycetaceae	GEVV02003803	158-1258	
58	Candida albicans	Debaryomycetaceae	AJJF01000018	229690-230790	
59	Candida apicola	Dipodascaceae/Trichomonascaceae	LBNK01000001	1505348-1506475	
60	Candida arabinofermentans	Pichiaceae	LWUO01000151	27020-28223	
61	Candida auris	Metschnikowiaceae	CVRJ01000290	11398-12333	
62	Candida boidinii_GF002	Pichiaceae	LMZO01000245	6317-7306	
63	Candida bracarensis	Saccharomycetaceae	CAPU01000232	5644-6522	
64	Candida corydali	Debaryomycetaceae	PPLL02000002	706690-707736	
65	Candida dubliniensis	Debaryomycetaceae	JFFX01000123	30304-31368	
66	Candida freyschussii	Phaffomycetaceae	PPLE01000012	250311-251270	
67	Candida galacta	Trichomonascaceae	PPSZ01002098	33940-35226	
68	Candida hispaniensis	Dipodascaceae/Trichomonascaceae	PPSY01000001	2014702-2015667	
69	Candida incommunis	Dipodascaceae/Trichomonascaceae	PPME02000011	302668-303819	
70	Candida magnoliae	Dipodascaceae/Trichomonascaceae	PEOD01000865	7819-8988	
71	Candida nivariensis	Saccharomycetaceae	CAPV01000060	243480-244358	
72	Candida orthopsilosis	Debaryomycetaceae	AMDC01000279	20734-22014	
73	Candida parapsilosis	Debaryomycetaceae	CABE01000009	580707-582047	
74	Candida psychrophila	Debaryomycetaceae	FYBW01000003	559568-560506	
75	Candida sorboxylosa	Pichiaceae	BCGC01000001	1931449-1932381	
76	Candida succiphila	Pichiaceae	BCGL01000007	214156-215560	
77	Candida tropicalis	Debaryomycetaceae	AAFN02000008	6074-7081	
78	Cephaloascus albidus	Cephaloascaceae	PPLM01000032	26357-27445	
79	Cephaloascus fragrans	Cephaloascaceae	PPLN02000001	474285-475430	
80	Citeromyces hawaiiensis	Saccharomycetaceae	PPLA01002746	25616-26530	
81	Citeromyces matritensis	Saccharomycetaceae	PPHV01001941	6068-5157	
82	Citeromyces siamensis	Saccharomycetaceae	PPLB01004164	14612-13641	
83	Clavispora fructus	Metschnikowiaceae	PPLK01000005	236811-238006	
84	Clavispora lusitaniae	Metschnikowiaceae	APWW01000058	60484-61302	
85	Cyberlindnera americana	Phaffomycetaceae	PPOA02000017	122024-122875	
86	Cyberlindnera fabianii	Phaffomycetaceae	BCGI01000003	429751-430779	
87	Cyberlindnera fabianii_Corr	Phaffomycetaceae	MPUK01000011	151165-152162	
88	Cyberlindnera jadinii	Phaffomycetaceae	BAEL01000048	410766-411686	
89	Cyberlindnera maclurae	Phaffomycetaceae	PPNZ02000024	21194-22087	
90	Cyberlindnera mrakii	Phaffomycetaceae	PPLH02000004	452175-453074	
91	Cyberlindnera petersonii	Phaffomycetaceae	PPMR02000039	5814-6770	
92	Cyberlindnera saturnus	Phaffomycetaceae	PPNR02000007	347315-348208	
93	Cyberlindnera suaveolens	Phaffomycetaceae	PPNS02000008	248817-249710	
94	Cyberlindnera xylosilytica	Phaffomycetaceae	PPNT01000024	67076-67981	
95	Cyphellophora europaea	Phaffomycetaceae	AOBU01000020	454768-456100	
96	Danielozyma ontarioensis	Debaryomycetaceae	PPLF01000015	69279-70336	
97	Deakozyma indianensis	Dipodascaceae/Trichomonascaceae	PPLG02000012	11519-12811	
98	Debaryomyces fabryi	Debaryomycetaceae	LMYN01000061	4962-5885	
99	Debaryomyces hansenii	Debaryomycetaceae	AHBE01000163	11011-11935	
100	Debaryomyces maramus	Debaryomycetaceae	PPOS02000008	389367-390319	
101	Debaryomyces nepalensis	Debaryomycetaceae	PPOR01000011	88606-87654	
102	Diddensiella caesifluorescens	Trichomonascaceae	PPJD01000001	2144865-2145920	
103	Dipodascus albidus	Dipodascaceae	PPJE01000002	112286-113479	
104	Dipodascus geniculatus	Dipodascaceae	PPOV01000008	258856-257669	
105	Eremothecium coryli	Saccharomycetaceae	AZAH01000009	78524-79351	
106	Eremothecium cymbalariae	Saccharomycetaceae	CP002501	265471-266319	
107	Eremothecium gossypii	Saccharomycetaceae	AE016815	201739-202575	
108	Eremothecium sinecaudum	Saccharomycetaceae	CP014245	111325-112119	
109	Fungal sp_EF0021	Leotiomycetes (Pezizomycotina)	AIET01000648	59794-61092	
110	Galactomyces candidum	Dipodascaceae	CCBN010000016	488135-489208	
111	Groenewaldozyma salmanticensis	Trichomonascaceae	PPNW02000006	110858-111763	
112	Hanseniaspora guilliermondii	Saccharomycodaceae	FQNF01000113	20097-20789	
113	Hanseniaspora opuntiae	Saccharomycodaceae	LPNL01000009	481128-481820	
114	Hanseniaspora osmophila	Saccharomycodaceae	LPNM01000009	200747-201511	
115	Hanseniaspora singularis	Saccharomycodaceae	PPNY01000005	39220-39924	
116	Hanseniaspora uvarum	Saccharomycodaceae	APLS01000001	478111-478806	
117	Hanseniaspora vineae	Saccharomycodaceae	JFAV03000033	231363-232148	
118	Hyphopichia burtonii	Debaryomycetaceae	LYBQ01000222	83367-84886	
119	Hyphopichia heimii	Debaryomycetaceae	PPMQ02000009	132646-133601	
120	Hypocrella siamensis	Debaryomycetaceae	JMQE01001689	50057-51433	
121	Kazachstania aerobia	Saccharomycetaceae	PPOI01000517	112112-112927	
122	Kazachstania intestinalis	Saccharomycetaceae	PPOJ02000005	44715-45446	
123	Kazachstania kunashirensis	Saccharomycetaceae	PPOH01000313	56835-57650	
124	Kazachstania martiniae	Saccharomycetaceae	PPOM02000553	177694-178551	
125	Kazachstania rosinii	Saccharomycetaceae	PPOD02000366	24003-24845	
126	Kazachstania saulgeensis	Saccharomycetaceae	FXLY01000006	679493-680326	
127	Kazachstania siamensis	Saccharomycetaceae	PPOL02000057	61141-61953	
128	Kazachstania solicola	Saccharomycetaceae	PPOG02001482	99860-100681	
129	Kazachstania spencerorum	Saccharomycetaceae	PPOE02001980	181905-182690	
130	Kazachstania taianensis	Saccharomycetaceae	PPOK01000003	36821-37681	
131	Kazachstania transvaalensis	Saccharomycetaceae	PPXN02000055	19321-20106	
132	Kazachstania turicensis	Saccharomycetaceae	PPOO01000954	482062-482889	
133	Kazachstania unispora	Saccharomycetaceae	PPON02000007	83447-84235	
134	Kluyveromyces aestuarii	Saccharomycetaceae	AEAS01000135	52331-53158	
135	Kluyveromyces dobzhanskii	Saccharomycetaceae	CCBQ010000013	77639-78496	
136	Kluyveromyces lactis	Saccharomycetaceae	CP021241	1295894-1296751	
137	Kluyveromyces marxianus	Saccharomycetaceae	AKFM02000034	1103780-1104643	
138	Kluyveromyces nonfermentans	Saccharomycetaceae	QYLQ01000007	29947-30762	
139	Kluyveromyces wickerhamii	Saccharomycetaceae	AEAV01000249	16233-17072	
140	Kodamaea laetipori	Metschnikowiaceae	PPJF01000004	759858-760983	
141	Kodamaea ohmeri	Metschnikowiaceae	PPNN01000003	312284-313462	
142	Komagataella pastoris	Phaffomycetaceae	CP014584	2900621-2901517	
143	Komagataella phaffii	Phaffomycetaceae	OCZY01000003	973562-974458	
144	Komagataella populi	Phaffomycetaceae	PPIA02000002	398283-399167	
145	Komagataella pseudopastoris	Phaffomycetaceae	PPKX02000005	400344-401228	
146	Kregervanrija delftensis	Pichiaceae	PPNL02000076	11571-12506	
147	Kregervanrija fluxuum	Pichiaceae	PPNM02000085	10760-11695	
148	Kuraishia capsulata	Pichiaceae	CBUD020000032	28837-29843	
149	Kuraishia molischiana	Pichiaceae	PPKW02000015	285069-285999	
150	Kuraishia ogatae	Pichiaceae	PPKV02000002	553809-554853	
151	Kurtzmaniella cleridarum	Debaryomycetaceae	PPNG01000210	405-1301	
152	Lachancea cidri	Saccharomycetaceae	FJUT01000002	154703-155518	
153	Lachancea dasiensis	Saccharomycetaceae	FJUP01000003	590095-590916	
154	Lachancea fermentati	Saccharomycetaceae	FJUO01000028	152140-152952	
155	Lachancea kluyveri	Saccharomycetaceae	AACE03000006	1052455-1053306	
156	Lachancea lanzarotensis	Saccharomycetaceae	CDLU01000031	517182-517994	
157	Lachancea meyersii	Saccharomycetaceae	FJUM01000019	316356-317162	
158	Lachancea nothofagi	Saccharomycetaceae	FJUQ01000024	596590-597519	
159	Lachancea quebecensis	Saccharomycetaceae	CZLH01000089	110370-111299	
160	Lachancea waltii	Saccharomycetaceae	AADM01000056	115915-116820	
161	PPJT02000000 Lipomyces arxii (dixit)	Lipomycetaceae	PPJT02000025	148881-150108	149830-150053
162	PPJU01000000 Lipomyces doorenjongii (dixit)	Lipomycetaceae	PPJU01000019	46837-47680	
163	Lipomyces japonicus	Lipomycetaceae	PPJV01000031	132264-133557	133235-133520
164	Lipomyces kononenkoae	Lipomycetaceae	PPJW01000022	109847-111134	109902-110168
165	Lipomyces lipofer	Lipomycetaceae	PPJX01000048	100457-101794	101438-101757
166	Lipomyces mesembrius	Lipomycetaceae	PPJS02000050	91321-91544	91321-91544
167	Lipomyces oligophaga	Lipomycetaceae	PPJR02000005	931312-932229	
168	Lipomyces starkeyi	Lipomycetaceae	LSGR01000110	255558-256819	256513-56764
169	Lipomyces suomiensis	Lipomycetaceae	PPJQ02000021	223901-224934	223935-224074
170	Lodderomyces elongisporus	Debaryomycetaceae	AAPO01000126	181096-182277	
171	Lomentospora prolificans	Debaryomycetaceae	NLAX01000010	4455673-4457176	
172	Magnusiomyces capitatus	Dipodascaceae	UIDF01000003	1271530-1272585	
173	Magnusiomyces ingens	Dipodascaceae	UIDE01000004	561266-562387	
174	Magnusiomyces tetraspermus	Dipodascaceae	PPOU02000045	25236-26579	
175	Martiniozyma abiesophila	Pichiaceae	PPIT02001147	38-934	
176	Metschnikowia aberdeeniae	Metschnikowiaceae	NKZB01000142	79998-80921	
177	Metschnikowia arizonensis	Metschnikowiaceae	NLAT01000007	236153-237049	
178	Metschnikowia australis	Metschnikowiaceae	MVNQ01000014	321380-322234	
179	Metschnikowia bicuspidata	Metschnikowiaceae	LXTC01000002	1791044-1791862	
180	Metschnikowia bowlesiae	Metschnikowiaceae	NLAJ01001636	86329-87294	
181	Metschnikowia colocasiae	Metschnikowiaceae	NKZQ01000869	115320-116249	
182	Metschnikowia dekortorum	Metschnikowiaceae	NKZU01000834	1017342-1018283	
183	Metschnikowia drakensbergensis	Metschnikowiaceae	NKYW01000006	82345-83274	
184	Metschnikowia drosophilae	Metschnikowiaceae	NXHB01000040	102778-103581	
185	Metschnikowia fructicola	Metschnikowiaceae	MTJM01000056	173487-174347	
186	Metschnikowia hamakuensis	Metschnikowiaceae	NLAB01001507	84072-84953	
187	Metschnikowia hawaiiensis	Metschnikowiaceae	NLAM01001311	182756-183664	
188	Metschnikowia hibisci	Metschnikowiaceae	NLAP01000009	77088-78011	
189	Metschnikowia kamakouana	Metschnikowiaceae	NKZX01000603	523966-524862	
190	Metschnikowia kipukae	Metschnikowiaceae	NKZJ01000144	412624-413553	
191	Metschnikowia lochheadii	Metschnikowiaceae	NKZT01000993	91620-92561	
192	Metschnikowia orientalis	Metschnikowiaceae	NXHA01000007	79423-80319	
193	Metschnikowia proteae	Metschnikowiaceae	NKYU01000006	497045-497974	
194	Metschnikowia reukaufii	Metschnikowiaceae	MDYR01000004	2301665-2302528	
195	Metschnikowia santaceciliae	Metschnikowiaceae	NKZL01001456	236382-237335	
196	Metschnikowia shivogae	Metschnikowiaceae	NLAF01000291	76028-76948	
197	Metschnikowia similis	Metschnikowiaceae	NKZP01001096	208162-209127	
198	Meyerozyma guilliermondii	Debaryomycetaceae	AAFM01000023	201131-202106	
199	Middelhovenomyces tepae	Dipodascaceae/Trichomonascaceae	PPNF02000011	438807-439718	
200	Millerozyma acaciae	Debaryomycetaceae	BCKO01000007	334018-335018	
201	Millerozyma farinosa	Debaryomycetaceae	NBND01000001	1931520-1932472	
202	Nadsonia fulvescens_elongata	Dipodascaceae/Trichomonascaceae	LXPB01000011	85644-86744	
203	Nadsonia fulvescens_fulvescens	Dipodascaceae/Trichomonascaceae	PPIV01000007	133234-134334	
204	Nadsonia starkeyi-henricii	Dipodascaceae/Trichomonascaceae	QBLK01000023	7384-8433	
205	Nakaseomyces bacillisporus_A	Saccharomycetaceae	CAPX01000015	83641-84396	
206	Nakaseomyces bacillisporus_B	Saccharomycetaceae	CAPX01000067	63347-63994	
207	Nakaseomyces delphensis	Saccharomycetaceae	CAPT01000006	83618-84499	
208	Nakazawaea holstii	CUG-Ala clade (formerly Pichiaceae)	PPKU01000035	101153-102028	
209	Nakazawaea peltata	CUG-Ala clade (formerly Pichiaceae)	BCGQ01000006	77047-77895	
210	Nakazawaea wickerhamii	CUG-Ala clade (formerly Pichiaceae)	NHAO01000004	259162-259974	
211	Naumovozyma castellii	Saccharomycetaceae	AACF01000001	144896-145807	
212	Naumovozyma dairenensis_A	Saccharomycetaceae	HE580269	1167181-1168128	
213	Naumovozyma dairenensis_B	Saccharomycetaceae	HE580271	710585-711532	
214	Neolecta irregularis	Taphrinomycotina	LXFE01000241	15283-16428	
215	Ogataea glucozyma	Pichiaceae	PPKO01000008	65354-66245	
216	Ogataea henricii	Pichiaceae	PPHT01000022	50858-51740	
217	Ogataea kodamae	Pichiaceae	PPKP02000013	38976-39825	
218	Ogataea methanolica	Pichiaceae	BCKW01000001	1310480-1311511	
219	Ogataea methylivora	Pichiaceae	PPKQ01000030	53345-54560	
220	Ogataea minuta	Pichiaceae	PPKR01000007	53665-54607	
221	Ogataea naganishii	Pichiaceae	PPKS01000017	221497-222721	
222	Ogataea nitratoaversa	Pichiaceae	PPIW01000012	207492-208694	
223	Ogataea nonfermentans	Pichiaceae	PPKT01000001	1663535-1664480	
224	Ogataea philodendri	Pichiaceae	PPKN02000002	340008-340930	
225	Ogataea pilisensis	Pichiaceae	PPKM01000027	8651-9906	
226	Ogataea pini	Pichiaceae	PPKL01000005	516068-516955	
227	Ogataea polymorpha	Pichiaceae	AECK01000005	960014-960915	
228	Ogataea populi-albae	Pichiaceae	PPIX02000001	1083760-1084683	
229	Ogataea ramenticola	Pichiaceae	PPKK02000008	209056-210243	
230	Ogataea trehaloabstinens	Pichiaceae	PPKJ01000001	1628206-1629084	
231	Ogataea trehalophila	Pichiaceae	PPKI01000006	58915-59946	
232	Ogataea zsoltii	Pichiaceae	PPKH02000046	11585-12519	
233	Pachysolen tannophilus	CUG-Ala clade (formerly Saccharomycetaceae)	CAHV01000045	9974-10876	
234	Peterozyma toletana	CUG-Ala clade (formerly Debaryomycetaceae)	PPKG01000010	30631-31452	
235	Peterozyma xylosa	CUG-Ala clade (formerly Debaryomycetaceae)	NHAN01000001	623513-624340	
236	Phaffomyces antillensis	Phaffomycetaceae	PPNI01000010	138601-139566	
237	Phaffomyces opuntiae	Phaffomycetaceae	PPNH01000003	128425-129405	
238	Phaffomyces thermotolerans	Phaffomycetaceae	PPNJ01000019	141207-142160	
239	Pichia exigua	Pichiaceae	PPIS01000005	15825-16712	
240	Pichia heedii	Pichiaceae	PPIR02000013	159633-160562	
241	Pichia kudriavzevii	Pichiaceae	BBOI01000433	33402-34355	
242	Pichia membranifaciens	Pichiaceae	AEHA01000085	13445-14410	
243	Pichia nakasei	Pichiaceae	PPIP01000024	12140-13048	
244	Pichia norvegensis	Pichiaceae	QAWC01000133	8183-9100	
245	Pichia occidentalis	Pichiaceae	PPIN02000006	13194-14102	
246	Pichia terricola	Pichiaceae	PPMO02000002	318949-319878	
247	Pneumocystis carinii	Taphrinomycotina	LFVZ01000006	398285-399432	
248	Pneumocystis jirovecii	Taphrinomycotina	LFWA01000004	236757-237921	
249	Pneumocystis murina	Taphrinomycotina	AFWA02000002	160375-161520	
250	Priceomyces carsonii	Debaryomycetaceae	PPMM02000015	144035-145011	
251	Priceomyces castillae	Debaryomycetaceae	PPMN02000014	209817-210775	
252	Priceomyces haplophilus	Debaryomycetaceae	BCIF01000002	122613-123557	
253	Priceomyces medius	Debaryomycetaceae	PPIF01000001	1406502-1407459	
254	Protomyces gravidus	Taphrinomycotina	QXDP01000107	177507-178717	
255	Protomyces inouyei	Taphrinomycotina	QXDQ01000097	64850-66143	
256	Protomyces inundatus	Taphrinomycotina	QXDR01000440	30979-32233	
257	Protomyces lactucaedebilis	Taphrinomycotina	MCFI01000019	266180-267474	
258	Protomyces macrosporus	Taphrinomycotina	QXDT01000037	27815-29087	
259	Protomyces pachydermus	Taphrinomycotina	QXDU01000264	115232-116529	
260	Saccharomyces arboricola_1	Saccharomycetaceae	ALIE01000153	37021-37917	
261	Saccharomyces arboricola_2	Saccharomycetaceae	ALIE01000033	49396-50577	
262	Saccharomyces cerevisiae_YDL204W_Rtn2	Saccharomycetaceae	BK006938	94605-95786	
263	Saccharomyces cerevisiae_YDR233C_Rtn1	Saccharomycetaceae	BK006938	929470-930357	
264	Saccharomyces eubayanus_1	Saccharomycetaceae	CP030946	715218-716099	
265	Saccharomyces eubayanus_2	Saccharomycetaceae	CP030948	88483-89637	
266	Saccharomyces kudriavzevii_1	Saccharomycetaceae	CP030965	896337-897227	
267	Saccharomyces kudriavzevii_2	Saccharomycetaceae	CP030965	97718-98902	
268	Saccharomyces mikatae_1	Saccharomycetaceae	AACH01000831	1661-2545	
269	Saccharomyces mikatae_2	Saccharomycetaceae	AACH01000071	1789-2973	
270	Saccharomyces paradoxus_1	Saccharomycetaceae	CP020245	898075-898974	
271	Saccharomyces paradoxus_2	Saccharomycetaceae	CP020245	97158-98342	
272	Saccharomyces pastorianus_1	Saccharomycetaceae	AYZZ01000025	50972-51859	
273	Saccharomyces pastorianus_2	Saccharomycetaceae	AYZZ01000061	11334-12515	
274	Saccharomyces uvarum_1	Saccharomycetaceae	AACA01000334	8041-8934	
275	Saccharomyces uvarum_2	Saccharomycetaceae	AACA01000298	8224-9411	
276	Saccharomycodes ludwigii	Saccharomycetaceae	UFAJ01000250	1123-2058	
277	Saccharomycopsis capsularis	Saccharomycopsidaceae	NHAM01000005	125405-126418	
278	Saccharomycopsis fermentans	Saccharomycopsidaceae	JNFW01000001	2830518-2831438	
279	Saccharomycopsis fodiens	Saccharomycopsidaceae	JNFV01000001	1669294-1670469	
280	Saccharomycopsis malanga	Saccharomycopsidaceae	BCGJ01000011	125270-126166	
281	Saitoella complicata	Taphrinomycotina	AEUO01000027	33520-34893	
282	Saprochaete clavata	Dipodascaceae	CBXB010000021	81861-82856	
283	Saturnispora dispora	Pichiaceae	NHAL01000429	19665-20573	
284	Saturnispora hagleri	Pichiaceae	PPIH01000023	21118-22026	
285	Saturnispora mendoncae	Pichiaceae	PPII01000023	75000-75896	
286	Saturnispora saitoi	Pichiaceae	PPIJ01000013	240558-241451	
287	Saturnispora serradocipensis	Pichiaceae	PPIK01000007	249884-250792	
288	Saturnispora silvae	Pichiaceae	PPIL02000017	37360-38208	
289	Saturnispora zaruensis	Pichiaceae	PPIM01000009	100582-101490	
290	Scheffersomyces lignosus	Debaryomycetaceae	BCGS01000003	794366-795522	
291	Scheffersomyces shehatae	Debaryomycetaceae	BDMO01000006	825042-826205	
292	Scheffersomyces stambukii	Debaryomycetaceae	NBZC01000049	33112-32207	
293	Schizosaccharomyces cryophilus	Taphrinomycotina	ACQJ02000142	85160-86062	
294	Schizosaccharomyces japonicus	Taphrinomycotina	AATM02000030	1491763-1493040	
295	Schizosaccharomyces octosporus	Taphrinomycotina	ABHY03000003	616125-617225	
296	Schizosaccharomyces pombe	Taphrinomycotina	CU329671	1262678-1263604	
297	Spathaspora arborariae	Debaryomycetaceae	AYLH01000139	51986-52855	
298	Spathaspora boniae	Debaryomycetaceae	MXQC01000114	2725-3699	
299	Spathaspora girioi	Debaryomycetaceae	LQMS01002195	12749-13621	
300	Spathaspora gorwiae	Debaryomycetaceae	LQMZ01002136	18240-19091	
301	Spathaspora hagerdaliae	Debaryomycetaceae	LQHL01001838	21028-21894	
302	Spathaspora passalidarum	Debaryomycetaceae	AEIK01000002	2009696-2010574	
303	Spathaspora xylofermentans	Debaryomycetaceae	NDXA01000135	23750-24721	
304	Spencermartinsiella europaea	Trichomonascaceae	PPIE02000002	744593-745729	
305	Sporopachydermia lactativora	Sporopachydermia clade	PPID01000021	89155-89910	
306	Sporopachydermia quercuum	Sporopachydermia clade	BCGN01000005	512558-513307	
307	Starmera amethionina	Phaffomycetaceae	PPNC01000002	157269-158222	
308	Starmera quercuum	Phaffomycetaceae	PPIB01000001	815542-816588	
309	Starmerella bacillaris	Dipodascaceae/Trichomonascaceae	MWPI01000003	58697-59686	
310	Starmerella bombicola	Dipodascaceae/Trichomonascaceae	BBSW01000051	58647-59774	
311	Sugiyamaella xylanicola	Trichomonascaceae	MQSX02000010	169323-170516	
312	Suhomyces canberraensis	Debaryomycetaceae	PPLP01000002	721030-721960	
313	Suhomyces emberorum	Debaryomycetaceae	PPLQ02000006	728183-729106	
314	Suhomyces pyralidae	Debaryomycetaceae	PPLO02000001	154699-155650	
315	Suhomyces tanzawaensis	Debaryomycetaceae	LYME01000197	173732-174662	
316	Taphrina deformans	Taphrinomycotina	CAHR02000056	31701-33048	
317	Taphrina flavorubra	Taphrinomycotina	BAVW01000029	29406-30762	
318	Taphrina populina	Taphrinomycotina	BAVX01000074	32080-33446	
319	Taphrina wiesneri	Taphrinomycotina	BAVU01000030	87060-88410	
320	Tetrapisispora blattae	Saccharomycetaceae	HE806316	338377-339114	
321	Tetrapisispora fleetii	Saccharomycetaceae	PPKB01000002	400225-401052	
322	Tetrapisispora iriomotensis	Saccharomycetaceae	PPKC01000002	330681-331511	
323	Tetrapisispora phaffii	Saccharomycetaceae	HE612858	442370-443197	
324	Teunomyces cretensis	Debaryomycetaceae	PPLT01000003	503084-504024	
325	Teunomyces gatunensis	Debaryomycetaceae	PPLS02000002	133094-134025	
326	Teunomyces kruisii	Debaryomycetaceae	PPLR01000017	98905-99846	
327	Tortispora caseinolytica	Trigonopsidaceae	LSKT01000001	422229-422954	
328	Tortispora ganteri	Trigonopsidaceae	PPNB01000008	142162-142887	
329	Tortispora starmeri	Trigonopsidaceae	PPMJ02000038	35356-36195	
330	Torulaspora delbrueckii	Saccharomycetaceae	NJII01000002	1045413-1046294	
331	Torulaspora franciscae	Saccharomycetaceae	PPHX02000002	307835-308716	
332	Torulaspora maleeae	Saccharomycetaceae	PPNA02000003	341305-342180	
333	Torulaspora microellipsoides	Saccharomycetaceae	FYBL01000009	460048-460935	
334	Torulaspora pretoriensis	Saccharomycetaceae	PPKE01000008	156057-156938	
335	Trigonopsis variabilis	Trigonopsidaceae	PPXM02000004	297440-298616	
336	Trigonopsis vinaria	Trigonopsidaceae	PPMK02000022	88269-89332	
337	Wickerhamia fluorescens	Debaryomycetaceae	BCGE01000001	2766439-2767432	
338	Wickerhamiella cacticola	Trichomonascaceae	PPIZ01000012	44447-45664	
339	Wickerhamiella domercqiae	Trichomonascaceae	BCGM01000001	2193381-2194364	
340	Wickerhamiella sorbophila	Trichomonascaceae	NDIQ01000021	1517070-1517927	
341	Wickerhamiella versatilis	Trichomonascaceae	BCJV01000003	1736531-1737541	
342	Wickerhamomyces alni	Phaffomycetaceae	PPMI02000037	96534-97475	
343	Wickerhamomyces anomalus	Phaffomycetaceae	AEGI02000858	2076-3155	
344	Wickerhamomyces bovis	Phaffomycetaceae	PPMH02000086	602-1513	
345	Wickerhamomyces canadensis	Phaffomycetaceae	PPMU02000010	117133-118050	
346	Wickerhamomyces ciferrii	Phaffomycetaceae	CAIF01000047	90698-91798	
347	Wickerhamomyces hampshirensis	Phaffomycetaceae	PPXL02000007	440777-441748	
348	Yamadazyma laniorum	Debaryomycetaceae	MVNT01000004	837205-838256	
349	Yamadazyma nakazawae	Debaryomycetaceae	PPMF01000013	247691-248845	
350	Yamadazyma philogaea	Debaryomycetaceae	PPMV02000023	29262-30400	
351	Yamadazyma scolyti	Debaryomycetaceae	PPMW01000001	31290-32126	
352	Yamadazyma tenuis	Debaryomycetaceae	AEIM01000039	205622-206678	
353	Yarrowia alimentaria	Dipodascaceae	ULGN01000003	651416-652510	
354	Yarrowia bubula	Dipodascaceae	PPMX01000022	165635-166714	
355	Yarrowia deformans	Dipodascaceae	BCIW01000003	214043-215155	
356	Yarrowia divulgata	Dipodascaceae	PPMY01000034	40574-41674	
357	Yarrowia keelungensis	Dipodascaceae	BCJD01000003	1037220-1038326	
358	Yarrowia lipolytica	Dipodascaceae	JAFI02000071	74476-75606	
359	Yarrowia porcina	Dipodascaceae	ULGT01000003	1980205-1981356	
360	Yarrowia yakushimensis	Dipodascaceae	ULGW01000004	2036793-2037854	
361	Zygoascus meyerae	Trichomonascaceae	PPMD02000002	550990-552066	
362	Zygoascus ofunaensis	Trichomonascaceae	PPMC02000006	227517-226417	
363	Zygosaccharomyces bailii	Saccharomycetaceae	CBTC010000018	156661-157881	
364	Zygosaccharomyces bisporus	Saccharomycetaceae	PPKA01000023	153744-154895	
365	Zygosaccharomyces kombuchaensis	Saccharomycetaceae	PPJZ01000080	18650-19807	
366	Zygosaccharomyces rouxii	Saccharomycetaceae	BBQV01000166	51953-53254	
367	Zygotorulaspora florentina	Saccharomycetaceae	PPJY02000008	125840-126715	
368	Zygotorulaspora mrakii	Saccharomycetaceae	PPHZ01000011	61430-62338	
1	Cyanodermella asteris	Lecanoromycetes (Pezizomycotina)	UYJD01000002	807552-809109	807712-807828
					807922-808026
2	Oidium heveae	Leotiomycetes (Pezizomycotina)	QVIK01000462	4531-3261	4353-4461
1	Lobosporangium transversale_1	Mortierellomycotina	MCFF01000001	541602-542760	
2	Lobosporangium transversale_2	Mortierellomycotina	MCFF01000014	80272-82047	
3	Mortierella alpina ATCC 32222_1	Mortierellomycotina	ADAG01000981	106500-107637	
4	Mortierella alpina ATCC 32222_2	Mortierellomycotina	ADAG01000948	45993-48082	
5	Mortierella alpina B6842_1	Mortierellomycotina	AZCI01000995	293387-294539	
6	Mortierella alpina B6842_2	Mortierellomycotina	AZCI01001087	76309-78382	
7	Mortierella elongata_1	Mortierellomycotina	LYLZ01000347	46217-47647	
8	Mortierella elongata_2	Mortierellomycotina	LYLZ01000294	92509-95098	
9	Mortierella verticillata	Mortierellomycotina	AEVJ01000267	21358-23983	
1	Ambispora leptoticha_TSA	Glomeromycotina	GGIK01042352	138-1124	
2	Claroideoglomus claroideum_TSA	Glomeromycotina	GGIJ01017484	32-958	
3	Funneliformis mosseae_TSA	Glomeromycotina	GGII01096376	279-1151	
4	Gigaspora margarita_TSA	Glomeromycotina	GBYF01037975	143-1147	
5	Paraglomus brasilianum_TSA	Glomeromycotina	GGIL01011681	27-1013	
6	Rhizophagus irregularis	Glomeromycotina	JARB01004825	6588-7977	
1	Allomyces macrogynus	Blastocladiomycota	ACDU01000293	2121-3244	
2	Catenaria anguillulae	Blastocladiomycota	MCFL01000293	6805-7759	
1	Absidia caerulea_TSA	Mucoromycotina	GFAW01017460	61-948	
2	Absidia glauca_1	Mucoromycotina	FKIZ01006360	15087-16234	
3	Absidia glauca_2	Mucoromycotina	FKIZ01006732	3059-4326	
4	Absidia repens_1	Mucoromycotina	MCGE01000019	335756-336981	
5	Absidia repens_2	Mucoromycotina	MCGE01000010	1332590-1334079	
6	Absidia repens_3	Mucoromycotina	MCGE01000016	918316-919516	
7	Actinomucor elegans_1	Mucoromycotina	BCHK01000002	1349456-1350694	
8	Actinomucor elegans_2	Mucoromycotina	BCHK01000006	1140669-1141855	
9	Actinomucor elegans_3A	Mucoromycotina	BCHK01000025	434753-435943	
10	Actinomucor elegans_3B	Mucoromycotina	BCHK01000007	538397-539305	
11	Apophysomyces elegans_1	Mucoromycotina	JNDQ01001456	79799-81179	
12	Apophysomyces elegans_2	Mucoromycotina	JNDQ01001463	208550-209447	
13	Apophysomyces trapeziformis_1	Mucoromycotina	JNDP01001304	319349-320248	
14	Apophysomyces trapeziformis_2	Mucoromycotina	JNDP01001350	135096-136896	
15	Apophysomyces trapeziformis_3	Mucoromycotina	JNDP01001350	135944-136896	
16	Apophysomyces variabilis_1	Mucoromycotina	AZYI01000194	74765-75852	
17	Apophysomyces variabilis_2	Mucoromycotina	MZZL01000029	74396-75777	
18	Apophysomyces variabilis_3	Mucoromycotina	MZZL01000134	61371-62268	
19	Bifiguratus adelaidae	Mucoromycotina	MVBO01000005	36226-37582	
20	Choanephora cucurbitarum_1	Mucoromycotina	LUGH01000231	16057-17013	
21	Choanephora cucurbitarum_2	Mucoromycotina	LUGH01000335	1256-2602	
22	Cokeromyces recurvatus	Mucoromycotina	JNEH01001254	24665-25754	
23	Cokeromyces recurvatus_1A	Mucoromycotina	JNEH01001741	11730-12819	
24	Cokeromyces recurvatus_2	Mucoromycotina	JNEH01002627	24582-25766	
25	Cunninghamella bertholletiae_1	Mucoromycotina	JNEL01000739	88494-90123	
26	Cunninghamella bertholletiae_2	Mucoromycotina	JNEL01000825	21490-23050	
27	Cunninghamella elegans_1	Mucoromycotina	JNDR01001372	19503-21132	
28	Cunninghamella elegans_2	Mucoromycotina	JNDR01001311	61067-62627	
29	Cunninghamella elegans_EST	Mucoromycotina	DY885649	60-788	
			DY887670	3-944	
30	Hesseltinella vesiculosa_1	Mucoromycotina	MCGT01000004	875379-876605	
31	Hesseltinella vesiculosa_2	Mucoromycotina	MCGT01000010	496312-497290	
32	Jimgerdemannia flammicorona	Mucoromycotina	RBNJ01010746	1595-2972	
33	Jimgerdemannia lactiflua	Mucoromycotina	RQIL01043335	2855-4317	
34	Lichtheimia corymbifera_1	Mucoromycotina	JNEU01001107	96465-98169	
35	Lichtheimia corymbifera_2	Mucoromycotina	JNEU01000764	36127-37826	
36	Lichtheimia corymbifera_3	Mucoromycotina	JNEU01001166	58538-59650	
37	Lichtheimia ramosa_1	Mucoromycotina	JNEP01001514	1695-2831	
38	Lobosporangium transversale_A	Mortierellomycotina	MCFF01000001	541602-542760	
39	Lobosporangium transversale_B	Mortierellomycotina	MCFF01000014	80272-82047	
40	Mortierella alpina_ATCC_32222_A	Mortierellomycotina	ADAG01000981	106500-107637	
41	Mortierella alpina_ATCC_32222_B	Mortierellomycotina	ADAG01000948	45993-48082	
42	Mortierella alpina_B6842_A	Mortierellomycotina	AZCI01000995	293387-294539	
43	Mortierella alpina_B6842_B	Mortierellomycotina	AZCI01001087	76309-78382	
44	Mortierella elongata_A	Mortierellomycotina	LYLZ01000347	46217-47647	
45	Mortierella elongata_B	Mortierellomycotina	LYLZ01000294	92509-95098	
46	Mortierella verticillata	Mortierellomycotina	AEVJ01000267	21358-23983	
47	Mucor circinelloides_1	Mucoromycotina	AMYB01000004	1741387-1742521	
48	Mucor circinelloides_2	Mucoromycotina	AMYB01000006	343906-345033	
49	Mucor circinelloides_3A	Mucoromycotina	AMYB01000012	557596-558834	
50	Mucor circinelloides_3B	Mucoromycotina	AMYB01000007	97483-98461	
51	Mucor irregularis	Mucoromycotina	AZYI01000194	74765-75852	
52	Mucor irregularis_TSA_1	Mucoromycotina	GFBC01007756	2941-3885	
53	Mucor irregularis_TSA_2	Mucoromycotina	GFBC01006458	4-1017	
54	Phycomyces blakesleeanus_1	Mucoromycotina	AMYC01000124	528827-530344	
55	Phycomyces blakesleeanus_2	Mucoromycotina	AMYC01000101	1483094-1483879	
56	Phycomyces blakesleeanus_3	Mucoromycotina	AMYC01000147	86592-87352	
57	Rhizomucor miehei_2	Mucoromycotina	AGBC01002287	8332-9504	
58	Rhizomucor pusillus_1	Mucoromycotina	FWWN02000583	4498-5696	
59	Rhizomucor pusillus_2	Mucoromycotina	FWWN02000637	61636-62796	
60	Rhizomucor variabilis_2	Mucoromycotina	JNES01000474	4316-5403	
61	Rhizopus delemar_1	Mucoromycotina	AACW02000151	188248-189255	
62	Rhizopus delemar_2	Mucoromycotina	AACW02000217	9165-10009	
63	Rhizopus delemar_3	Mucoromycotina	AACW02000250	588665-589594	
64	Rhizopus delemar_4	Mucoromycotina	AACW02000289	80548-81828	
65	Rhizopus oryzae_TSA	Mucoromycotina	GDUK01019782	214-1119	
66	Saksenaea oblongispora_1	Mucoromycotina	JNEV01001563	36932-38188	
67	Saksenaea oblongispora_2	Mucoromycotina	JNEV01001568	221382-222589	
68	Saksenaea vasiformis_1	Mucoromycotina	JNDT01002364	19602-20874	
69	Saksenaea vasiformis_2	Mucoromycotina	JNDT01002389	42325-43236	
70	Syncephalastrum monosporum	Mucoromycotina	JNEN01001167	19449-20422	
71	Syncephalastrum racemosum	Mucoromycotina	JNDN01000807	67042-68026	
72	Thermomucor indicae-seudaticae_1	Mucoromycotina	JSYX01000031	36469-37572	
73	Thermomucor indicae-seudaticae_2	Mucoromycotina	JSYX01001292	2542-3754	
74	Umbelopsis isabellina_1	Mucoromycotina	JNEQ01000049	328889-329954	
75	Umbelopsis isabellina_2	Mucoromycotina	JNEQ01000043	502680-504017	
76	Umbelopsis isabellina_3	Mucoromycotina	JNEQ01000033	51589-52686	

Supplementary Table S2. Recommended Media for Maintenance and Growth of *Lipomyces* yeasts.

**Species**	**Reference Collection Number**	**Recommended medium**	**Composition of Recommended Medium**
Lipomyces suomiensis	CBS 7251	GPYA	4% glucose<br>0.5% peptone<br>0.5% yeast extract<br>(1.5% agar)
Lipomyces kononenkoae	CBS 2514	MEX	3% malt extract<br>(1.5% agar)
Lipomyces lipofer	CBS 944<br>(NCAIM Y.00351)	MEX	3% malt extract<br>(1.5% agar)
Lipomyces starkeyi	CCY 33-1-1	YEPD	1 % yeast extract<br>2 % peptone<br>2% dextrose<br>(1,5% agar)

Supplementary Table S3. Oligonucleotide primers used in this study.

Oligonucleotide primers for RT-PCR verification of stwintron splicing intermediates (see Supplementary Figures).

**Strain**	**Primer**	**Sequence (5'-)**
Lipomyces lipofer NCAIM Y.00351	L.lipofer_SplintF	ATGTCGCAAGCCGCTACTACC
Lipomyces lipofer NCAIM Y.00351	L.lipofer_SplintR2	CAGTTAGTATTAGAAGTTAC
Lipomyces suomiensis CBS 7251	L.suomi_SplintF3	ATGTCTACTAGTGCTGCGC
Lipomyces suomiensis CBS 7251	L.suomi_SplintR	TGTACATCGATCAGTCTTTC
Lipomyces starkeyi CCY 33-1-1	L.starkeyi_SplintF	TCTTCAGCGGTGTCTCGTACGT
Lipomyces starkeyi CCY 33-1-1	L.starkeyi_SplintR	ATGATCCAGTCAGCCCTCATC

Oligonucleotide primers for cDNA and/or gDNA sequencing.

**Strain**	**Primer**	**Sequence (5'-)**
Lipomyces lipofer NCAIM Y.00351	L.lipofer_F2	CTGCCGATTGATCCAACGTAC
Lipomyces lipofer NCAIM Y.00351	L.lipofer_R3	CACCAGATACGTCAATCATCC
Lipomyces lipofer NCAIM Y.00351	L.lipofer_R2	ACAGTTAGTATTAGAAGTTAC
Lipomyces suomiensis CBS 7251	L.suomi_F1	TTCCACGGTCTGATCCTACT
Lipomyces suomiensis CBS 7251	L.suomi_R1	ATCAGACGGAGCTATAGCAT
Lipomyces suomiensis CBS 7251	L.suomi_R3	GTGTATTTCATGTCGTTACGC
Lipomyces starkeyi CCY 33-1-1	L.starkeyi_seq_F1	CACAACACGAACCCAAAACATC
Lipomyces starkeyi CCY 33-1-1	L.starkeyi_seq_R1	ATACCGCAACGACACCATCATC
Lipomyces starkeyi CCY 33-1-1	L.starkeyi_R1	ATGATCCAGTCAGCCCTCATC
Lipomyces kononenkoae CBS 2514	L.kono_F1	GTCTGCCTGTAGATCGTATC
Lipomyces kononenkoae CBS 2514	L.kono_R1	GGTTAGCATTAGGTATGGCT
Lipomyces kononenkoae CBS 2514	L.kono_R2	CGTCAGCTCCTTATTCGGAT

Supplementary Figure S1

Lipomyces lipofer

a

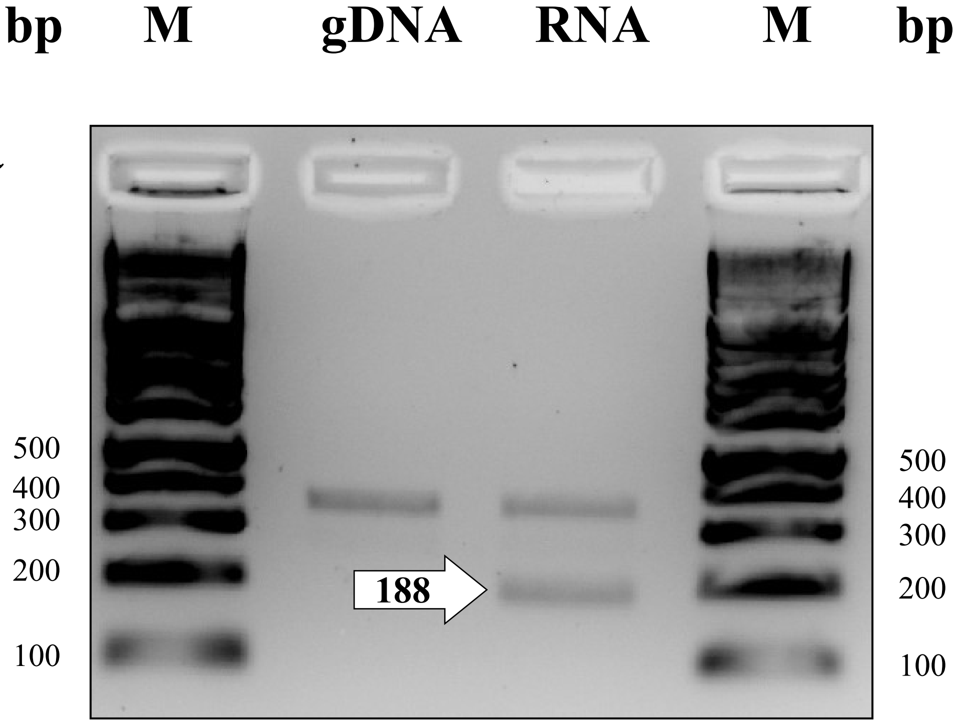

b

```
ATGTCGCAAGCCGCTACTACCTCTTCGTCGGTGC
CTCgtgagttcagatttgttatcttgttttgtt
caaggatgatacatgtgatcttgatcgcgctgca
attgatccgctctccaaacaagtttccatttgcg
cgcatctgaacattcatggaataaataatttggt
aacttctaatactaactg
```

Figure S1. The splicing intermediate of the [D4,5] stwintron in the *L. lipofer* gene for the reticulon-like protein. See Fig. 5a in the main manuscript for the corresponding stwintron splicing scheme. **(a)** The PCR amplication products corresponding to the primary transcript of the *L. lipofer* gene (predicted size: 346 bp) and the [D4,5] stwintron splicing intermediate (predicted size: 188 bp), separated in a native agarose gel, stained with ethidium bromide and photographed under UV illumination. With genomic DNA (gDNA) as template, only the longer fragment is amplified. The gene-specific oligonucleotide primer pair (Table S3) does not amplify off mature mRNA template. The uncropped image displays the relevant part of the original gel including standard molecular size markers. **(b)** The sequence of the *L. lipofer* stwintron splicing intermediate cloned cDNA (188 bp) from which the predicted internal intron is absent but with the external intron retained. Intronic sequences are printed in lower case letters: The reconstituted donor element of the external intron (5'-gtga|gt) is underlined.

Supplementary Figure S2

Lipomyces suomiensis

a

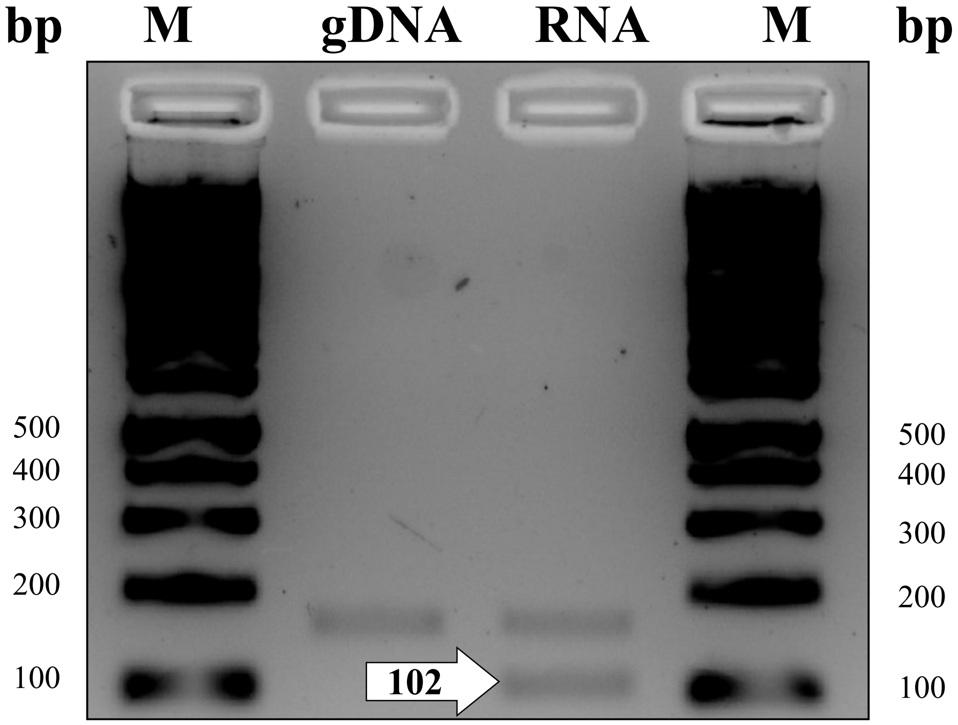

b

```
ATGTCTACTAGTGCTGCGCCCGTGAGGATTGATC
gtgagtgaatgccattgaatatgatgatttattg
attgagaaaatcatgaaagactgatcgatgtaca
```

Figure S2. The splicing intermediate of the [D7,8] complex intervening sequence (stwintron "*sensu lato*") in the *L. suomiensis* gene for the reticulon-like protein. See Fig. 5b in the main manuscript for the corresponding stwintron splicing scheme. **(a)** RT-PCR analysis of the *L. suomiensis* RNAs: The predicted sizes of the amplified cDNAs are 163 bp (primary transcript) and 102 bp ([D7,8] splicing intermediate). With genomic DNA (gDNA) as template, only the longer fragment is amplified. The gene-specific oligonucleotide primer pair (Table S3) does not amplify off mature mRNA template. The uncropped image displays the relevant part of the original native agarose gel including standard molecular size markers. **(b)** Sequence of the *L. suomiensis* stwintron splicing intermediate cloned cDNA (102 bp) from which the predicted internal intron is absent but with the external intron retained. Intronic sequences are printed in lower case letters: The functional donor element of the external intron (5'-gtgagt) is underlined.

Supplementary Figure S3

Lipomyces starkeyi

a

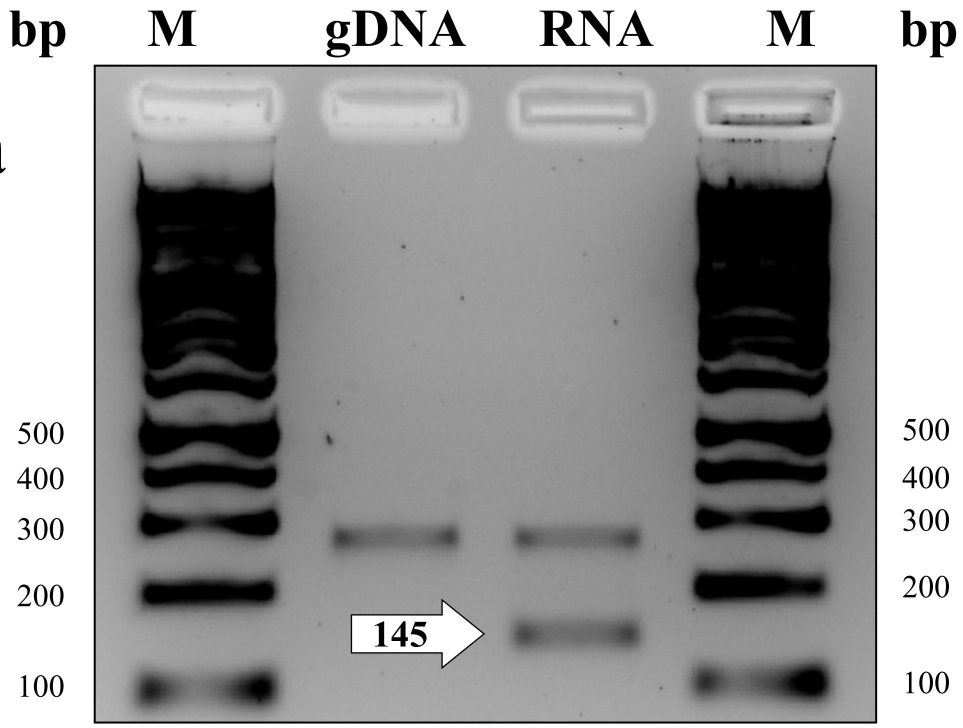

b

```
CTTCAGCAGTGTCTCgtacgtattgatttatttt
tgtcatattgttctcgcgttttcgaagctgcgat
cacatggactcgctcttctacttcgacttgtgaa
gagttggactttcgataatccagatgaggactga
ctcgatcat
```

Figure S3. The splicing intermediate of the [D6,7] complex intervening sequence (stwintron "sensu lato") in the *L. starkeyi* gene for the reticulon-like protein. See Fig. 5c in the main manuscript for the corresponding stwintron splicing scheme. **(a)** RT-PCR analysis of the *L. starkeyi* RNAs: The predicted sizes of the amplified cDNAs are 265 bp (primary transcript) and 145 bp ([D6,7] splicing intermediate). With genomic DNA (gDNA) as the template, only the longer fragment is amplified. The gene-specific oligonucleotide primer pair (Table S3) does not amplify off mature mRNA template. The uncropped image displays the relevant part of the original native agarose gel including standard molecular size markers. **(b)** Sequence of the *L. starkeyi* stwintron splicing intermediate cloned cDNA (145 bp) from which the predicted internal intron is absent but with the external intron retained. Intronic sequences are printed in lower case letters: The functional donor element of the external intron (5'-gtacgt) is underlined.

Supplementary Text File 1. Trimmed alignment (136 informative residues) of 902 reticulon-like proteins from Ascomycota in PHYLIP format. See Methods section of the main manuscript for details.

```
902 136
Trigonopsis_vinaria                        DLLTWKKPYFTGSVFAFLVTFAIAIKTVDFFGLALKSIYTILAIFITIEYTARVAGNGFLNSIAHGAIVYFQHILFIEDISGSAFALIFVLYYVIRLLSLWTIGFIGLILAFSLPPIYLRFQPEIDAQVEPYIAKV
Trigonopsis_variabilis                     DLLTWKNPYVTAPVFIGLLGLFIVIKKVDFLRFSLGLYSSTLAFTLAADYVSRFIGGSIVQSISHGGLLAVQSIIFVDDPSTAAFLTTFVLYYVTRLLSLWTIGLLTLILAFTAPPVYLRFQAPIDAKIEPYIAKV
Aspergillus_nidulans                       SLLSWEQPRATAASFASVVAFIFAARYLPLLRWFFKFLYLVLGVTAAAEIGGRVVLQGLTSSLEDFLFIEFQRILFAENATTAAAAAAFSAYWLIKILPFWGLSLLAVTIAYLGPFVYLNNQEIIDAQIGDYSNKA
Aspergillus_mulundensis                    SLLSWEQPRATAASFASVVAFIFAARYLPLLRWFFKFLYFVLGITAAAEIGGRLVLQGLTSSLEDFLFIEFQRVLFAENATSAAAAAAFSAYWLIKILPFWGLSLLAVTIAYFGPFIYINNREVIDAQIGDYSNKA
Aspergillus_unguis                         SLLSWEQPRATAISFASVVAFIFAARYLPLLRWVFKFLYVVLGFTALFEIVGKLVFQGIASSLEDFVLIEFQRILFAENITVAAFSASFSAYWLIKILPFWGLSLLAVTIAYLGPLVYINNRELIDAQIGEYSNKA
Aspergillus_ustus                          SLLSWEQPRATAASFASVVAFIFAARYLPLLRWFFKFLYVALAFTAALELGGRLVLQGLASSLEDFVLIEFQRILFAENVTVAAFSAAFSAYWLIKILPFWGLSLLAVSIAYLGPLIYINNRELIDAQIGDYSNKA
Aspergillus_calidoustus                    SLLSWEQPRATAASFASVVAFIFAARYLPLLRWFFKFLYVALGFTAALELGGRLILQGLASSVEDFVLIEFQRILFAENVTVAAFSAAFSAYWLIKILPFWGLSLLAVSIAYLGPLVYITNREIIDAQIGDYSNKA
Aspergillus_sydowii                        SLLSWEQPRATAASFASVVVFIFAARYLPLLRWLFKFLYLALGSMAALEIGGRLVLQGLASSLEDFVLIEFQRILFAENATVAGFSAAFSAYWLIKILPFWGLSLLAVTIAYLGPLVYMNNRELIDAQIGDYSNRA
Aspergillus_sp_MA_6037                     SLLSWEQPRATAASFASVVVFIFAARYLPLLRWFFKFLYLALGSMAALEIGGRLLLQGLASSLEDFVLIEFQRILFAENATVAGFSAAFSAYWLIKILPFWGLSLLAVTIAYLGPLVYINNRELIDAQIGDYSNKA
Aspergillus_sp_MA_6041_A                   SLLSWEQPRATAASFASVVVFIFAARYLPLLRWFFKFLYLALGSMAALEIGGRLLLQGLASSLEDFVLIEFQRILFAENATVAGFSAAFSAYWLIKILPFWGLSLLAVTIAYLGPLVYINNRELIDAQIGDYSNKA
Aspergillus_versicolor                     SLLSWEQPRATAASFASVVVFIFAARYLPLLRWFFKFLYLALGSMAALEIGGRLVLQGLASSLEDFVLIEFQRILFAENATVAGFSAAFSAYWLIKILPFWGLSLLAVTIAYLGPLVYINNRELIDAQIGDYSNKA
Aspergillus_rambellii                      SLLSWEEPRATAASFASVVAFIFAARYLPLLRWFFKFLYVALGFTAALEIGGRIVLQGLASSLEDFVLIEFQRILFAENVTVAAFSAAFTAYWLIKFLSFWGLSLLAVTIAYLGPLVYINNREIIDAQIGGYSSKA
Aspergillus_ochraceoroseus                 SLLSWEEPRATAASFASVVAFIFAARYLPLLRWFFKFLYVALGFTAALEIGGRIVLQGLASSLEDFVLIEFQRILFAENVTVAAFSAAFTAYWLIKFLSFWGLSLLAVTIAYLGPLVYINNREIIDAQIGGYSSKA
Aspergillus_thermomutatus                  RLLSWEQPRATAVSFASVVAFIFAARYLPLLRWFFKFMYLALGFTAAAEIGGRLVLQGLASSLEDLLLLEFQRILFAENVTVAAFSAAFTAYWLIKFLPFWGLSLIGVTIAYLGPLVYINNREVIDAQIGDYSAKA
Neosartorya_fischeri                       RLLSWEQPRATAVSFVSVIAFIFAARYLPLLRWFFKFTYLALGFTAAAEIGGRLVLQGLASSLEDLLLLEFQRILFAENVTVAAFSAAFTAYWLIKFLPFWGLSLIAVTIAYLGPLVYINNREVIDAQIGDYSAKA
Aspergillus_fumigatus                      RLLSWEQPRATAVSFVSVIAFIFAARYLPLLRWFFKFTYLALGFTAAAEIGGRLVLQGLASSLEDLLLLEFQRILFAENVTVAAFSAAFTAYWLIKFLPFWGLSLIAVTIAYLGPLVYINNREVIDAQIGDYSAKA
Aspergillus_neoellipticus                  RLLSWEQPRATAVSFVSVIAFIFAARYLPLLRWFFKFTYLALGFTAAAEIGGRLVLQGLASSLEDLLLLEFQRILFAENVTVAAFSAAFTAYWLIKFLPFWGLSLIAVTIAYLGPLVYINNREVIDAQIGDYSAKA
Aspergillus_lentulus                       RLLSWEQPRATAVSFVSVIAFIFAARYLPLLRWFFKFTYLALGFTAAAEIGGRLVLQGLASSLEDLLLLEFQRILFAENVTVAAFSAAFTAYWLIKFLPFWGLSLIAVTIAYLGPLVYINNREVIDAQIGDYSAKA
Aspergillus_novofumigatus                  RLLSWEQPRATAVSFVSVIAFIFAARYLPLLRWFFKFTYLALGFTAAAEIGGRLVLQGLASSLEDLLLLEFQRILFAENVTVAAFSAAFTAYWLIKFLPFWGLSLIAVTIAYLGPLVYINNREVIDDQIGDYSAKA
Neosartorya_udagawae                       RLLSWEQPRATAVSFVSVIAFIFAARYLPLLRWFFKFTYLALGFTAAAEIGGRLVLQGLASSLEDLLLLEFQRILFAENVTVAAFSAAFTAYWLIKFLPFWGLSLIAVTIAYLGPLVYINNREVIDAQIGDYSAKA
Aspergillus_clavatus                       RLLSWEQPRATAVSFASVVTFIFAARYLPLLRWFFKFVYLALGFTAAAELGGRLVLQGLASSLEDLLLLEFQRILFAENVTVAAFFAAFTAYWLIKFLPFWGLSLIAVTIAYLGPLVYLSNREVIDAHIEDYSSKA
Aspergillus_bombycis                       SLLSWEQPRATAASFATVVTFIFAARYLPLLRWFFKFIYVALGFTAAAELGGRLVLQGLASSYEDLVLLEFQRILFAENITVAAFAAAFSAYWLIKWLPFWGLSLIAVTIAYIGPLVYMNNREVIDAQIDDYSAKA
Aspergillus_parasiticus                    SLLSWEQPRATAASFATVVTFIFAARYLPLLRWFFKFIYVALGFTAAAELGGRLVLQGLASSYEDLVLLEFQRILFAENITVAAFAAAFSAYWLIKWLPFWGLSLIAVTIAYIGPLVYMNNREVIDAQIDDYSAKA
Aspergillus_arachidicola                   SLLSWEQPRATAVSFATVVTFIFAARYLPLLRWFFKFIYVALGFTAAAELGGRLVLQGLASSYEDLVLLEFQRILFAENITVAAFAAAFSAYWLIKWLPFWGLSLIAVTIAYIGPLVYMNNREVIDAQIDDYSAKA
Aspergillus_flavus                         SLLSWEQPRATAVSFATVVTFIFAARYLPLLRWFFKFIYVALGFTAAAELGGRLVLQGLASSYEDLVLLEFQRILFAENITVAAFAAAFSAYWLIKWLPFWGLSLIAVTIAYIGPLVYMNNREVIDAQIDDYSAKA
Aspergillus_nomius                         SLLSWEQPRATAASFATVVTFIFAARYLPLLRWFFKFVYVALGFTAAAELGGRLVLQGLASSYEDLVLLEFQRILFAENITVAAFAAAFSAYWLIKWLPFWGLSLIAVTIAYIGPLVYMNNREVIDAQIDGYSAKA
Aspergillus_hancockii                      SLLSWEQPRATAASFATVVTFIFAARYLPLLRWFFKFIYVALGFTAAAELGGRLVLQGLASSYEDLVLLEFQRILFAENITVAAFTAAFSAYWLIKWLPFWGLSLIAVTIAYIGPLVYINNREVIDAQIDDYSAKA
Aspergillus_terreus                        NLLSWEQPRATAISYATVVTFIFAARYLPLLRWFFKFLYVTLGFTAAAELGGRLVFQGLASSLADLLLLEFQRILFAENITVAAFISAFSAYWLIKWLPLWGLSLIAVNIAYFGPLVYMNNQEVIDAHIDDYSAKA
Aspergillus_pseudoterreus                  NLLSWEQPRATAISYATVVTFIFAARYLPLLRWFFKFLYVTLGFTAAAELGGRLVFQGLASSLADLLLLEFQRILFAENITVAAFVSAFSAYWLIKWLPLWGLSLIAVNIAYFGPLVYMNNQEVIDAHIDDYSAKA
Aspergillus_wentii                         SLLSWEQPRATAASFASVVFFIFAARYLPLLRWAFKLVYVLLGFTAAAEIGGRLILQSIASSVEDLVLLEFQRVLFAENVTVAAFFSAFTAYWLIKFLPLWGLSLITVTIAYIGPLVYINNREVIDAQISDYSAKA
Aspergillus_campestris                     SLLSWEQPRATATSFASVVVFIFAARYLPLLRWFFKFIYVALGFMAALEIGGKVALYGLASSLEDLVLIEFQRVLFVENITLASFFAAYTAYWLIKFLPFWGLSLFAVSVAYLGPLVYINNREVIDAQIDDYSAKA
Aspergillus_candidus                       SLLSWEQPRATATSFASVVVFIFAARYLPLLRWFFKFIYVALGFMAALEIGGKVALYGLASSLEDLALIEFQRVLFVENITLASFFAAFTAYWLIKFLPFWGLSLFAASVAYLGPLVYINNREVIDAQIDDYSAKA
Aspergillus_taichungensis                  SLLSWEQPRATATSFASVVVFIFAARYLPLLRWFFKFIYVALGFMAALEIGGKVALYGLASSLEDLALIEFQRVLFVENITLASFFAAFTAYWLIKFLPFWGLSLFAVSVAYLGPLVYINNREVIDAQIDDYSAKA
Aspergillus_persii_Corr                    SLLSWEQPRATATSFATVVTLIFAARYLPLLRWVFKFMYLALGFTAAAEIGGRILFQGLVKNMEDIVLLEFQRILFAENITVAAFFAAFSAYWLIMFLPFWGLSLLAATIAYIGPLVYMNNREVIDAQINDYSAKA
Aspergillus_sclerotiorum                   SLLSWEQPRATAASFATVVTLIFAARYLPLLRWVFKFMYLALGFTAAAEIGGRILFQGLVKNMEDIVLLEFQRILFAENITVAAFFAAFSAYWLIMFLPFWGLCLLAATIAYIGPLVYMNNREVIDAQINDYSAKA
Aspergillus_westerdijkiae                  SLLSWEQPRATAASFATVVTFIFAARYLPLLRWIFKFTYVALGFTTAAEIGGRVLFQGLVKNMEDIVLLEFQRILFAENITVAAFFAAFSAYWLIKFLPFWGLSLLAATIAYIGPLVYMNNREVIDAQITDCSVKA
Aspergillus_steynii                        SLLSWEQPRATAASYATVVTFILAARYLPLLRWVFKFSYVALGFTAAAEIGGRILFQGLVKNMEDIVLLEFQRILFAENITVAAFFAAFSAYWLIRFLPFWGLSLLAATIAYFGPLVYMDNREVIDAQINDYSAKA
Aspergillus_sp_MA_6041_B                   SLLSWDQPRATVTSYISTIIFIFAARYLPLLRWAFKLAYVTLGFTAAAELGGRLVLQGVADSLEDLGFLEFQRVLFVENITVFAFFAALNAYWLIKFLPFWGLSVIAVTVAYFGPLVYMNNYEVIDAHVDDYSNLA
Aspergillus_glaucus                        SLLSWEQPRATATSYISVITFIFAARYLPLLRWVFKLAYVVLGFTAAFEIGGRLVFQGLSSSLEDLGLLEFQRVLFVENITVAAFFAALNAYWLIKFLPFWGLSFIVVTIAYFGPLTYMNHSEVIDAHIDDYSNKA
Aspergillus_ruber                          SLLSWEQPRATATSYISVITFIFAARYLPLLRWVFKLAYVVLGFTAAFEIGGRLVFQGLSSSLEDLGLLEFQRVLFVENITVAAFFAALNAYWLIKFLPFWGLSFIVVTVAYFGPLTYMNHSEVIDARIDDYSNKA
Aspergillus_cristatus                      SLLSWEQPRATATSYISVITFIFAARYLPLLRWVFKLAYVALGFTAAFEIGGRLAFQGLSSSLEDLGLLEFQRVLFVENITVAAFFGALNAYWLIKFLPFWGLSLIVVTIAYFGPLTYMNHSEVIDAHIDDYSNKA
Aspergillus_chevalieri                     SLLSWEQPRATATSYISVITFIFAARYLPLLRWVFKLAYVALGFTAAFEIGGRLAFQGLSSSLEDLGLLEFQRVLFVENITVAAFFGALNAYWLIKFLPFWGLSLIVVTIAYFGPLTYMNHSEVIDAHIDDYSNKA
Talaromyces_purpureogenus                  SLLSWEQPRATAVSYATVIGFIFAARYLPLLRWAFKFLYMSLGLTAAVEIAGQLILQGIASSLEDLFLIEFQRILFAENVTVAAFTAAFTGYWLVRFVPLWGLAVISVTVAYLGPLVYISNREIIDEQIGEYSHKA
Mycosphaerella_populi                      SLLSWEQPRATAVSYATVIGFIFAARYLPLLRWAFKFLYMSLGLTATVEVAGQVLFQGFASSLEDLFLIEFQRILFAENVTVAAFFAAFTGYWLIRFVPLWGLALIAVTTAYIGPLIYISNQEIIDQQIGDYSNKA
Talaromyces_leycettanus                    SILSWEQPRATAVSFVSIVAFIFAARYLPLLRWAFKFLYLSLGFTAAVEIAGRLVLQGLASSQEDFFLLEFQRILFAENVTVAAFTAAFSAYWLIKWVPFWGLSLIAVTIAYFAPLIYISNREIIDSQIGEYSAKA
Byssochlamys_spectabilis                   SLLSWEHPRATALSFASVVTFIFAARYLPLIRWAFKFLYLALGFTATLEIAGKLVLQGLASSLEDLFLIEFQRILFAENITVAAFTAAFLSYWLIKIVPLWGLSLIGVTIAYLGPLIYINNRELIDAHIGTYSSKA
Cladosporium_cladosporioides_IMV_00236     SLLSWEHPRATAVSFASVVTFIFAARYLPLLRWAFKFLYVALGFTAALELAGKLVLQGLASSLEDLFLIEFQRILFVENITVAAFTAAFFSYWLIKILPLWGLSLIGVTVAYLGPLIYISNREIIDGYIGTYSSKA
Byssochlamys_sp__AF001                     SLLSWENPRATAVSFASVVTFIFAARYLPLLRWAFKFLYVALGFTATLEIAGRLVLQGLVTSLEDLFLIEFQRVLFAENITVAAFTAAFLSYWLIKILPLWGLSLIAVTVAYLGPLVYINNREIIDAQIGSYRSKA
Byssochlamys_nivea                         SLLSWENPRATAVSFASVVTFIFAARYLPLLRWAFKFLYVALGFTATLEIAGRVVLQGLASSLEDLFLIEFQRILFAENITVAAFTAAFLSYWLIKILPLWGLSLIAVTVAYLGPLVYINNREIIDAQIGTYSSKA
Thermoascus_crustaceus                     SLLSWERPRATAVSYATVVSFIIAARFFPLLRWFFKFLYVALGFTAALEIAGKTVMQGLASSLEDMFLIEFQRILFAENITVAAFFAAFTSYWLIKILPLWGLSLIAVTIAYMGPLVYINNREEIDAQIDEYSNKA
Elaphomyces_granulatus                     SLLSWERPRATAISFASVIAFILAARFLPLLRWVFKFLYLSLGLMAAIEITGRILLQGLASSLEDFFLIEFQRILFAENITVVAFSAAFFSYWLIKILPLWAFSLLAVTVAYLGPLAYISNQEIIDEQIGDYSAKA
Rasamsonia_emersonii                       SLLSWERPRTTAISFVSVVSFILAARYLPLLRWVFKFLYVSLGTMAALEVAGKLVFQGLASSLDDLFLLEFQRTLFAENVTIAAFSAAFVSYWLSKILPLWGLALIAVTIAYLGPLVYISNREIIDEQISDYSAKA
Talaromyces_islandicus                     SLLSWERPRATAISFASVVTFILAARYLPLLRWVFKFLYVTLGFTAAAEGLGKLVLHGLTSSLEDLFLIEFQRVLFAENVTVAAFVAAFLSYWLIKIVPLWGLSLIGVSVAYLGPLVYISNREIIDEQINDYTSKA
Talaromyces_wortmannii                     SLLSWERPRATAISFASVVAFILAARYLPLLRWVFKFLYVTLGFTAAAEGLGKLVLQGLASSLEDLFLIEFQRVLFAENVTAAAFIAAFLSYWLIKVVPVWGLALIGVNVAYLGPLIYINNREIIDEQINDYTSKA
Talaromyces_piceae                         SLLSWERPRATAISFVSVVSFILAARYLPLLRWIFKFLYVTLGCTAAAEGLGKLVFQGLASSLEDLFLIEFQRVLYAENVTAAAFVAAFLSYWLIKIVPFWGLSLIGISVAYLAPLIYVSNRELIDEQISNYSNKA
Talaromyces_verruculosus                   SLLSWERPRATAISFASVVFFILAARFLPLLRWVFKFLYISLGVTAAAEGAGKLILQGLTSSLEDLFLIEFQRILFAENVTAAAFIAAFLSYWLIKIVPVWGLALLGTAVAYLGPLIYISNREIIDEQINDYSSKA
Talaromyces_cellulolyticus                 SLLSWERPRATAISFASVVFFILAARFLPLLRWVFKFLYISLGVTAAAEGAGKLILQGLTSSLEDLFLIEFQRILFAENVTAAAFIAAFLSYWLIKIVPVWGLALLGTAVAYLGPLIYISNREIIDEQINDYSSKA
Talaromyces_pinophilus                     SLLSWERPRATAISFASVVFFILAARFLPLLRWVFKFLYISLGVTAAAEGAGKLILQGLTSSLEDLFLIEFQRILFAENVTAAAFIAAFLSYWLIKIVPVWGLALLGTAVAYLGPLIYISNREIIDEQINDYSSKA
Talaromyces_amestolkiae                    SLLSWERPRATAISFASVVFFILAARFLPLLRWVFKFLYISLGVTAAAEGAGKLIFQGLTSSLEDLFLIEFQRILFAENVTAAAFIAAFLSYWLIKVVPVWGLALLGTTVAYLGPLIYISNREFIDEQINDYSSKA
Talaromyces_marneffei                      SLLSWERPRATAISFASVVFFILAARFLPLLRWVFKFLYISLGVTAAAEGAGKLILRGLTSSLEDLFMIEFQRILFAENVTAAAFVAAFLSYWLIKIVPVWGLALLGTTIAYLGPLIYLSNREIIDDQINDYSSKA
Talaromyces_stipitatus                     SLLSWERPRATAISFASVVFFILAARFLPLLRWVFKFLYISLGVTAAAEGAGRLVLQGLTSSLEDLFLIEFQRILFAENVTSAAFVAAFLSYWLIKIVPVWGLALLGTSIAYLGPLFYISNREIIDEQVNDYSSKA
Talaromyces_borbonicus                     SLLSWERPRATAISFASVVFFILAARFLPLLRWVFKFLYISLGVTAAAEGAGKLILQGLTSSLEDLFLIEFQRILFAENVTAAAFIAAFGSYWLIKVVPVWGLALIGTTIAYLGPLIYISNRELIDEQINDYSSKA
```

Talaromyces_atroroseus	SLLSWERPRATAISFASVVFFILAARFLPLLRWIFKFLYISLGVTAAAEGAGKLILQGLTSSLEDLFLIEFQRILFAENVTAAAFVAAFLSYWLIKIMPVWGLSLLGVTIAYLGPLVYISNREIIDEQINNYSTKA
Phialosimplex_sp_HF37	SLLSWEQPRATAVSFATVVFSIFAARYLPLLRWVFKFLYLSLGFTVAAEVAGRLVLQGLAGSLEDLFLIEFQRVLFAENITVAAFSGAFTAYWLIKILPFWGLSLIAVTIAYLGPFFYMHNREVVDSHLGDYSAKA
Xeromyces_bisporus	SLLSWEQPRATAISYASVVTFILAARFLPLLRWALKFSYLALGFTAAAEIAGRVVLQSIAKSLEDVFLLEFQRILFAENVTVAAFSAAFVSYGLVRILPFWGLSLIGATAIYIGPLVYISNREIIDAQVGDYSNKA
Monascus_ruber	SLLSWEQPRATAVSYASVVTFILAARFLPLLRWVFKLLYLALGFTAAAEIAGRVVLQSIASSLEDVFLLEFQRILFAENVTVAAFSAAFIAYFLVKFLPLWGLSLLSATIIYIGPLIYISNRELIDAQIGDYSTKA
Monascus_purpureus	SLLSWEQPRATAVSYASVVAFILAARFLPLLRWVFKLLYLALGFTAAAEIAGRVVLQSIASSLEDVFLLEFQRILFAENVTVAAFSAAFIAYFLVKFLPLWGLSLLSATIIYIGPLIYISNRELIDAQIGDYSTKA
Byssochlamys_sp_BYSS01	SLLSWEQPRATAISYASVVAFILVARFLPLLRWVFKILYLALGFTAAAEIAGQFVLQSILSSLEDVFLLEFQRILFAENVTVAAFSAAFVSYWLVKFLPLWGLALISATVIYIGPLIYISNRELIDAQIGDYSTKA
Thermomyces_lanuginosus	SLLSWEQPRATAISFAGVVTFILAARYLPLLRWFFKFLYLTLGSTAALEIAGRAVFQGFTSKLEDILLIEFQRTLFAENITVSMFTAALLSYWLIKILPLWGLTLLAVFVAYLGPLIYISNREIIDEQINDYTSKA
Aspergillus_zonatus	SLLSWEQPRATAVSYFSIVAFIVAARFLPLLRWVFKFLYLALGFTASIELAGRLVFQGLASSQEDVFLLEFQRVLFVENATVAAFSAAFVAYWLIKWIPFWGLSLIAVTIIYFVPLFYISNHELVDSQIGGYSAKA
Amauroascus_niger	SLLSWERPQTTAASYASVVAMIVAGRYLPLLRWALKLLYLSLGFTAVVEVSGRAIFRGLMSNLEDLFLIEFQRILFVENLTIATFTAALMSYWLVRFLPLWGLALIGVTVAYLGPLIYLSNKEFIDEQIGEYRAMA
Amauroascus_mutatus	SLLSWERPQTTAASYASVVAMIIAGRYLPLLRWALKLLYLSLGFTAVLEVSGRAIFRGLMSNLEDLFLIEFQRILFVENLTIATFTAALVSYWLVRWLPLWGLALLGVTVAYLGPLIYLSNKDFIDEQIGEYRAKA
Chrysosporium_queenslandicum	SLLSWERPQATAVSYASVVAMIIAGRYLPLLRWALKLLYLSLGFMAIVEVSGRAIFRGLVSNLEDLFLIEFQRILFVENLTLTTFTAALTSYWLVRFVPLWGLALMGVTIAYLGPLVYISNREFIDEQVGEYRAKA
Coccidioides_immitis	SLLSWERPQTTAASYASVVAMIIAGRYLPLLRWALKLLYISLGFTAIVEVSGRAIFRGLMSNLEDLFLIEFQRILFVENLTLATFSAALTSYWLVRFLPLWVLALIGVTVAYLGPLFYLSNKEFIDEQIGEYRAKA
Coccidioides_posadasii	SLLSWERPQTTAASYASVVAMIIAGRYLPLLRWALKLLYISLGFTAIVEVSGRAIFRGLMSNLEDLFLIEFQRILFVENLTLATFSAALTSYWLVRFLPLWVLALIGVTVAYLGPLFYLSNKEFIDEQIGEYRAKA
Byssoonygena_ceratinophila	SLLSWERPQTTAISYASIVTMIIAGRYLPLLRWALKLLYLSLGFTAIVEVTGRTVFRGLMSSLEDLFLIEFQRILFVENLTLATFTAALTSYWLVRFLPLWGLALLGVTVAYLGPLIYLSNKEFIDEQVGEYRAKA
Uncinocarpus_reesii	SLLSWERPQTTAISYASVAGMIVAGRYLPLLRWALKLLYVSLGFTAIVEVIGRTVFRGLMSGLEDLFLIEFQRILFVENLTLATFTAALVSYWLVRFLPLWGLALIGVNIAYLGPLLYLSNKEFIDEQIGEYRAKA
Ophidiomyces_ophiodiicola	SLLSWERPQTTAISYVTVVAMIVAGRYLPLLRWALKLLYLSLGFTAIVEISGRVVLHGFMSTLEDLFLIEFQRILFVENLTLATFIAALTSYWLVRFLPLWGLALISVTIAYLGPLVYLSNKEFIDEQAGEYRAKA
Paracoccidioides_brasiliensis	SLLSWERPRATAFSFASIISLIIAARYLPLLRWIFKFTYLSLGFTVALEIVGKVLLRGLASSLEDLFLIEFQRILFAENLTIAAFAAALTSYWLVRFLPLWGLALTMVSTAYLGPLVYINNKELIDTQIGDYRAKA
Paracoccidioides_lutzii	SLLSWERPRATAFSFATIISLIIAARYLPLLRWIFKFTYLSLGFTVALEIVGKVLLRGLASSLEDLFLIEFQRILFAENLTIAAFTAALTSYWLVRFLPLWGLALTMVSTAYLGPLVYINNKELIDTQIGDYRAKA
Histoplasma_capsulatum	SLLSWERPRTTALSFVSIVSLIVAARYLPLLRWLFKFSYLALGFTIALEIAGKVLLRGLASSLEDLFLIEIQRILFAENLTLAAFSAALTSYWLVRFLPTWGLSLILVSVAYLGPLIYVNNKELIDSQIGDYRAKA
Blastomyces_percursus	SLLSWERPRTTAVSFATIVSLIIAARYLPLLRWLFKFSYLSLGFTIALEVAGKVMFRGLASSLEDLFLIEFQRILFAENLTLASFAAALTSYWLVRFLPLWGLALILVSVAYLGPLVYINNREIIDTQIGDYTAKA
Blastomyces_gilchristii	SLLSWERPRTTAVSFATIVSLIIAARYLPLLRWLFKFSYLSLGFTISLEVAGKVMFRGLASSLEDLFLIEFQRILFAENLTLAAFTAALTSYWLVRFLPLWGLALILVSVAYLGPLVYINNREIIDTQIGDYTAKA
Emmonsia_parva	SLLSWERPRTTAVSFATIVSLIIAARYLPLLRWLFKFSYLSLGFTIALEVAGKVMFRGLASSLEDLFLIEFQRILFAENLTLAAFTAALTSYWLVRFLPIWGLALTLVSVAYLGPLIYINNREIIDTQIGDYTAKA
Emergomyces_orientalis	SLLSWERPRTTAVSFATIVGLIVAARYLPLLRWIFKFAYLSLGFTVALEVAGKVLLRGLASSLEDLFLIEFQRILFAENLTLAAFTAALTSYWLVRFLPLWGLSLILVSVTYLGPLIYINNRELIDTQIGDYRAKA
Emmonsia_crescens	SLLSWERPRTTAVSFATIVSLIVAARYLPLLRWIFKFAYLSLGFTVTLEVAGKVLLRGLASSLEDLFLIEFQRILFAENLTLAAFTAALTSYWLVRFLPLWGLSLILVSVAYLGPLVYVNNRELIDTQIGDYRAKA
Emergomyces_pasteuriana	SLLSWERPRTTAVSFATIVSLIVTARYLPLLRWIFKFAYLSLGFTVALEVAGKVLLRGLASSLEDLFLIEFQRILFAENLTLAAFSAALTSYWLVRFLPLWGLSLILVSVTYLGPLVYFNNREFIDTQIGDYRAKA
Helicocarpus_griseus	SLLSWERPRATAVSFATIVSLIIAARYLPLLRWAFKLVYISLGFTVALEIAGKVLLRGLASSLEDLFLIEVQRILFVENLTLAAFTAALTSYWLVRFLPLWGLSLVLVSAAYLGPLVYINNRELIDTQIGEYSAKA
Polytolypa_hystricis	SLLSWERPRATALSFATVVSVIIAARYLPLLRWAFKFLYVSLGFTVIIEVGSKLVLRGIASSLEDLFLIEFQRILFAENLTLFAFTAALSAYWLVRFLPLWGLALIAVTIAYLGPLIYINNKELIDSQIEDYRHKA
Malbranchea_cinnamomea	SLLSWQRPRHTAISYVTVVFTILAARFLPLLRWALKFVYLSLGIAASMEVLGRAVFRGITTDLEDMFLIEFQRILFVENLTIGAFFAALVTYSLVRFLPLWGLALLGVTVAYMGPLIYINNKEFIDEQINDYRARA
Cladonia_metacorallifera	NLLSWENPRATTLSFISIILFIFAARYLPALRWSFKALYMVLGVTAAAEVAGKLVLRGLTSSLEDVFVIEFQRVLFAENVTIACFLASVVSYWLIKILPFWGLSLISVCCIYLGPLIYMTNQELIDHHLGDYTAKA
Cladonia_macilenta	NLLSWENPRATTLSFISIIFFIFAARYLPALRWSFKALYMVLGVTAAAEVAGKLVLRGLTSSLEDVFVIEFQRVLFAENVTIACFLASVVSYWLIKILPFWGLSLISVCCIYLGPLIYMTNQELIDHHLGDYTAKA
Cladonia_uncialis	NLLSWENPRATTLSFISIILFIFAARYLPALRWSFKALYMVLGVTAAAEVAGKLVLQGLTSSLEDVFVIEFQRVLFAENVTIACFLASVVSYWLIKILPFWGLSLIGVCCIYLGPLIYMTNQELIDHHVGDYTAKA
Cetradonia_linearis	NLLSWENPRATTLSFVSIILFIFAARYLPALRWSFKALYMLLGVTAAAEVAGKLVIQGLTSSLEDVFIIEFQRVLFAENVTIAAFLAAVVSYWLIKIVPFWGLTLIGVSCIYLGPLVYMTNQDLIDHHVGDYTAKA
Evernia_prunastri	NLLSWDNPRATTLSFVSIVLFIFAARYLPALRWSFKALWMVLGITASAEIAGKLVLQGLASSLEDVFVIEFQRVLFAENVTVAGFVAALLSYWLIKVVPFWGLSLIAVSCVYLGPLIYMTNKELIDHHVGDYSAKA
Pseudevernia_furfuracea	NLLSWENPRATTLSFVSIVLFIFAARYLPALRWSFKALWMVLGITASAEIAGKLVLQGLASSLEDIFVIEFQRVLFAENVTIAGFVAALLSYWLIKVVPFWGLSLIAISCIYIGPLIYMTNKELIDHHLGDYSAKA
Gyalolechia_flavorubescens	NLLSWENPRATTLSFLTTILIIFGARYLPALRWTFKALYVVLGITATAELGGKLVLQGLASSLEDVFVIEFQRVLFAEKPTVAAFLASFLSYWLIKFTPLWGLSVIAVSLVYLGPLIYLSNKELIDHHVGDYTSKA
Lasallia_pustulata	SLLSWEHPRASALSYLAIVAFIFIARYLPALRLLFKLLSYVIGVTTVLEIVGKTVFRGLASSVEDVFVIEVQRVVFAENVSGAAFVATFLSYWLIKFLPLWGLSLIAVSIAYLAPLIYINNKAIIDEQLGDYTSKA
Lasallia_hispanica	SLLSWEHPRASALSYLAIVAFIFIARYLPALRLLFKLLSYVIGVTTVLEIVGKTVFRGLASSIEDVFVIEVQRVVFAENVSGAAFVATFLSYWLIKFLPLWGLSLIAVSIAYLAPLIYINNKAIIDEQLGDYTSKA
Umbilicaria_muehlenbergii	SLLSWEHPRASALSYLAIVAFIFAARYLPALRLLFKALSYAIGFTTVAEVAGKLVFQGLATSIEDVFVIEFQRVIFAENVSGAAFVASFLSYWLIKFLPLWGLSLIAVSIAYLAPLIYINNKELIDEQFGDYTSKA
Ramalina_peruviana_Corr	NLLSWEHPRATAISFVASVLFIFACRYLPALRWAFKVLYLTLGATALAEVAGKLALQGLASSLEDVFVIEFQRVLFAENVTIAACTAAFLSYWLIKIVPFWGLSLIGTSLAYLGPLVYINNKELIDIHLGDYTSKA
Ramalina_intermedia	NLLSWEHPRATAISFVASVLFIFACRYLPALRWAFKVLYLTLGATALAEVAGKLALQGLASSLEDVFVIEFQRVLFAENVTIAACTAAFLSYWLIKIVPFWGLSLIGTSLAYLGPLVYINNKELIDTHLGDYTSKA
Endocarpon_pusillum_KoLRI	TLLSWKHPRATAISFATSLIFIFASRYLPLIRWFFKIAYLAFGVTALAEILGKFVVTGVTTSLDDVFIIEFQRILFAENPTVAAFLSAFISYYLIKIVPLWGLTLLSTFVIYLAPLIYIKNKDVIDSQLGEYAAKA
Phaeomoniella_chlamydospora	SLLSWEHKRATAIAFAATVAFIFAARYLKVLRFIFKTTYMVLGITAAAEVAGKFLLTGLTSSLDDVFVIEGQRLLFAENPTIGAFFAALVSYFLIRFVPLWGLSLLATLVIYLGPLIYLENKELIDGHLGEYAAKA
Coniosporium_apollinis	RLLSWRNPRATAISFLVSVLLIFATRYLPILRWFFKGAYIVTGITAAAEILGQAAFNGFATQTDDIFVIEFQRIIFAENVTLAAFASALISYFLIKVVPLWGLSLIATSIIYLAPLIYVKNKDVIDEQLDEYSHKA
Helminthosporium_solani	RLLSWKNPRATAISFAAAVIFIFAARYLNVIRYIFKALYMTLGITATAELAGSMTIKGLTSQLDDVFVIESQRIVFAENVTVAAFFASLISYFLIKFVPLWGLSLIATVIAYLGPLVYIKNKEVIDAQLQQYTAKA
Periconia_macrospinosa	RLLSWKNPRATAISFAASIIFIISARYLNVIRYIFKALYITLGITASAEAAGHLTLKGLTSQLDDVFVIESQRIIFAENVTGAAFFASLISYFLIKFVPLWGLALIATTIAYLGPLVYIKNKEVIDAQLQQYTAKA
Nigrograna_mackinnonii	RLLSWKNPRATAISFVVSVAFIFAARYLNVIRYLFKAIYLTLGATATAEVAGKLTFQGFTSQLDDVFVIESQRIVFAENVTVAAFFASLISYFLIKFVPLWGLALIATTVAYIAPLVYIKNKEVIDAQLGEYTSKA
Clohesyomyces_aquaticus	RLLSWKNPRATAISFAVSVLFIFAARYLNVIRYIFKAVYMTLGATATAEIAGKLAFRGFTSQLDDVFVIESQRILFAENVTSAAFLASLVSYFLIKFVPLWGLTLIATTVAYLGPLVYIQNKEVIDTHLGEYTHKA
Beverwykella_pulmonaria	RLLSWKNPRATAIAFAVSVLFIFAARYLNVIRYIFKIVYLTLGVTASAELGGKLAFRGLTSQLDDVFVIESQRIVFAENVTGAAFFASLISYFLIKFVPLWGLTLIATTVAYLGPLIYVKNKEVIDAQLKEYTHKA
Corynespora_cassiicola	RLLSWRNPRATAISFAVSIAFIFAARYLNVIRYIFKAIYITLGVTASAELVGQVALRGLTSQLDDVFVIESQRIVYAENVTIAAFFASLFSYFLIKFVPLWGLTLIATVIAYLGPLIYIKNKEVIDAQLKEYTAKA
Paraphaeosphaeria_sporulosa	RLLSWRNPRATGLTFAAVVAFIFAARYLNVFRYILKAVWVTLGITAAAEVGGQLTIKGLTSQTEDIFVIESQRIVFAENVTIAAFFASLTTYFLIKFVPLWGLALIATNVAFLGPLIYIQNKEVIDAQLQQYTNKA
Glonium_stellatum	RLLSWKNPRATAISFAISVAFIFAARYLNILRYIFKGLYVLLGLTASAEIAGKAVFNGFTSQLDDVFVIEFQRVVFAENVTIAAFMSAFISYYLIKFVPLWGLSLISISIIYLAPLVYLQNKELIDAQIGEYTSKA
Cenococcum_geophilum	RLLSWKNPRATAISFAISVAFIFAVRYLNILRYIFKGLYVLLGLTASAEIAGKAVFNGFTSQLDDVFVIEFQRVVFAENVTTVAFLSAFISYYLIKFLPLWGLSLISTTIIYLTPLVYIKNKEIIDAQIGEYTSKA
Lepidopterella_palustris	RLLSWKKPRATAISFAACVAFIFAARFLNILRYVFKALYILLGLTASAEIAGKAIFNGFSSQLDDLFVIEFQRIVFAENVTIAAFFSAFISYYLIKVVPLWGLSLISTSVIYLAPLVYLKNKEIIDSQLGEYTHKA
Hysterium_pulicare	RLLSWKNPRATAISFAISVLFIFAARYLNVIRYMFKILYIVLGLTASAEVAGKLVFNGLTSQLDDVFIIEFQRVVFAENVTIGAFVASFLAYYLIKVLPFWGLSLLATTVLYISPLIYLKNKEVIDAQLGEYSNKA
Alternaria_alternata	RLLSWKNPRATGIAFGATVVFIFAARYLNLIRYIFKTSYMVLGLTAAAEVVGQVAIRGLTSQTDDFFVIESQRIMFAENVTVAAFFASLISYFLIKFVPLWGLALISTFITFLGPLVYIQNKEVIDAQLQQATAKA
Alternaria_arborescens	RLLSWKNPRATGIAFGATVVFIFAARYLNLIRYIFKTSYMVLGLTAAAEVVGQVAIRGLTSQTDDFFVIESQRIMFAENVTVAAFFASLISYFLIKFVPLWGLALISTFITFLGPLVYIQNKEVIDAQLQQATAKA
Alternaria_brassicicola	RLLSWKNPRATGIAFGATVVFIFAARYLNLIRYIFKTSYMVLGATAAAEVVGQVAIRGLTSQTDDFFVIESQRIMFAENVTVAAFFASLISYFLIKFVPLWGLALISTFVTFLGPLVYIQNKEVIDAQLQQATAKA
Alternaria_solani	RLLSWKNPRATSIAFGATVIFIFAARYLNLIRYIFKTSYMVLGATAAAEVVGQMAIRGLTSQTDDFFVIESQRIMFAENVTVAAFFASLISYFLIKFVPLWGLALISTFVTFLGPLVYIQNKEVIDAQLQQATAKA
Stemphylium_lycopersici	RLLSWKNPRATGIAFAGTVVLIFAARYLNLIRYIFKTSYMVLGATAAAEVVGQLTIRGLTSQTDDLFVIESQRIVFAENVTVAAFFASLLSYFLIKFVPLWGLALVSTFITFLGPLVYIKNKEVIDAQLQQATAKA
Cochliobolus_miyabeanus	RLLSWKNPRATAIAFASTVVFIFAARYLNLIRYIFKSSYLVLGATATAEIAGQLTIRGITSQTDDFFVIESQRIVFAENVTVAAFFASLISYFLIKWVPLWGLGLIATHIAFLGPLIYIKNKDVIDAQLQQATAKA
Cochliobolus_heterostrophus	RLLSWKNPRATGIAFASTVAFIFAARYLNLIRYIFKTSYLVLGATATAEIAGQLTIRGLTSQTDDFFVIESQRIVFAENVTVAAFFASLISYFLIKWVPLWGLGLIATHIAFLGPLIYIKNKDVIDAQLQQATAKA
Cochliobolus_sativus	RLLSWKNPRATGIAFASTVVFIFAARYLNLIRYIFKTSYLVLGATATAEIAGQLTIRGLTSQTDDFFVIESQRIVFAENVTVAAFFASLISYFLIKWVPLWGLGLIATHIAFLGPLIYIKNKDAIDAQLQQATAKA
Bipolaris_cookei	RLLSWKNPRATGIAFASTVVFIFAARYLNLIRYIFKTSYLVLGATATAEIAGQLTIRGLTSQTDDFFVIESQRIVFAENVTVAAFFASLLSYFLIKFVPLWGLALIATHITFLGPLIYIKNKDVIDAQLQQATAKA
Cochliobolus_carbonum	RLLSWKNPRATAIAFASTVVFIFASRYLNLIRYMFKTSYMVLGATATAEIAGQLTIRGLTSQTDDFFVIESQRIVFAENVTIGAFFAFLISYFLIKWVPLWGLALIATHITYLGPLIYIKNKDVIDAQLQQATAKA
Curvularia_sp_IFB_Z10	RLLSWKNPRATSIAFAGTVVFIFAARYLNLIRYMFKMSYMVLGATAAAEIAGQMTIRGLTSQTDDFFVIESQRIVFAENVTVAAFVASLLSYFLIKFVPLWGLTLISTVITFLGPLIYIKNKDVIDAQLQQATAKA
Curvularia_lunata	RLLSWKNPRATSIAFASIIVFIFAARYLNLIRYIFKTSYMVLGATATAEIAGQLTIRGLTSQMDDFFVIESQRIVFAENVTVAAFFASLLSYFLIKFVPLWGLALISTVITFLGPLIYIKNKDVIDAQLQQATAKA

Curvularia_geniculata	RLLSWKNPRATSIAFASIIVFIFAARYLNLIRYIFKTSYMVLGATATAEIAGQLTIRGLTSQMDDFFVIESQRIVFAENVTVAAFFASLLSYFLIKFVPLWGLALISTVITFLGPLIYIKNKDVIDAQLQQATAKA
Setosphaeria_turcica	RLLSWKNPRATAIAFASSIVFIFASRYLNLIRYIFKTSYMVLGATAAAEVAGQLTIRGLTSQTDDFFVIESQRVVFAENVTIAAFFASLFSYFLIKFVPLWGLALIATHITFLGPLIYIKNKDVIDAQLQQATAKA
Pyrenophora_tritici_repentis	RLLSWKNPRATGIAFAGTVVFIFAARYLNLIRYIFKASYMILGLTAAAEVAGQATIRGLTSQTDDFFVIESQRIVFAENVTVTAFFASLLSYFLIKFVPLWGLALISTVITFLGPLIYIKNKDVIDAQLQQATAKA
Pyrenophora_teres	RLLSWKNPRATGIAFAGTVVFIFAARYLNLIRYIFKASYMILGLTAAAEVAGQATIRGLTSQTDDFFVIESQRIVFAENVTVTAFFASLLSYFLIKFVPLWGLTLISTVITFLGPLIYIKNKDLIDAQLQQATAKA
Pyrenophora_seminiperda	RLLSWKNPRATGIAFASTVVFIFATRYLNLIRYIFKASYMILGLTAAAEVAGQATIRGLTSQTDDLFVIESQRIVFAENVTVTAFFTSLLSYFLIKFVPLWGLALISTVITFLGPLVYIKNKDVIDAQLQQATAKA
Leptosphaeria_maculans	RLLSWKNPRATAIAFATTVILIFASRYLNLIRYVFKTSYLVLGATATAEVVGQLAIRGLTSQTDDVFVIESQRVVFAENVTIAAFFASLTSYFLIKFVPLWGLSLIGTVITFLGPLVYIKNKEFIDAQLQQATAKA
Leptosphaeria_biglobosa	RLLSWKNPRATGIAFATTVIIIFASRYLNLIRYIFKASYLVLGATATAEVVGQLAIRGLTSQTDDVFVIESQRVVFAENVTIAAFFTSLISYFLIKFVPLWGLTLIGTVITFLGPLVYIKNKEVIDAQLQQATAKA
Pyrenochaeta_sp_UM_256	RLLSWKNPRATGIAFAITVIFIFAARYLNLVRYIFKASYVTLGATATAEIVGQLAFRGLTSQTDDVFVIESQRIVFAENVTVAAFFASLISYFLIKFVPLWGLTLIATFVTFLGPLVYIQNKELIDAQLQQATAKA
Pseudopyrenochaeta_lycopersici	RLLSWKNPRATGIAFASTIIFIFAARYLNVIRYFFKAVYVTLGATATAEVAGQLAFRGLTSQTDDLFVIESQRIVFAENVTIAAFFASLISYFLIKFVPLWGLGLIATLVAYLGPLIYIKNKEVIDAQIQQATAKA
Pyrenochaeta_sp_DS3sAY3a	RLLSWKNPRATGIAFAVTVAFIFAARYLNLIRYIFKASYMTLGLTAAAEVAGQLTIRGLTSQTDDVFVIESQRIVFAENVTVAAFFASLISYFLIKFVPLWGLTLIATLITFLGPLIYIQNKEAIDAQLQQATAKA
Parastagonospora_avenaria	RLLSWKNPRATGIAFASTVAFIFAARYLNVIRYALKLLYLTLGATVTAEVAGQLAFRGLTSQLDDVVVIESQRIVFAENVSGAAFFASLISYFLIKFVPLWGLTLIATVVTYLGPLVYIQNKEVIDAQLQQYTNKA
Parastagonospora_avenae	RLLSWKNPRATGIAFASTVAFIFAARYLNVIRYALKLLYLTLGATVTAEVAGQLAFRGLTSQLDDVVVIESQRIVFAENVSGAAFFASLISYFLIKFVPLWGLTLIATVVTYLGPLVYIQNKEVIDAQLQQYTNKA
Parastagonospora_nodorum	RLLSWKNPRATGIAFASTVVFIFAARYLNVIRYALKLLYLTLGATVTAEVAGQLAFRGLTSQLDDVVVIESQRIVFAENVSGAAFFASLISYFLIKFVPLWGLTLIATVVTYLGPLVYIQNKEVIDAQLQQYTNKA
Stagonospora_sp_SRC1lsM3a	RLLSWKNPRATGIAFAATVIFIFAARYLNIIRYSLKLLYVVLGTTATAEVAGHLAFRGLTSQLDDVFVIESQRIVFAENVTGAAFFASLISYYLIKFVPLWGLTLIATLVTYLGPLIYIQNKEVIDAQLQQYTAKA
Paraphoma_sp_B47_9	RLLSWKNPRATGIAFAATILFIFAARYLNIIRYGLKLLYLTLGATATAEIAGQAAFRGLTSQLDDVFVIESQRIVFAENVTVAAFFASLISYFLIKFVPLWGLTLIATLVTYLTPLIYIQNKEAIDAQLQQYTAKA
Shiraia_sp_Slf14	RLLSWKNPRATGIAFASVILFIFAARYLNIIRYGLKLLYITLGTTAAAELGGSLVFRGLTSQLDDVFVIESQRIVFAENVTIAAFFASLISYFLIKFVPLWGLTLIATLVTFLGPLIYIQNKETIDAQLQQYTAKA
Epicoccum_sorghinum	RLLSWKNPRATGISFAAAVLFIFAARYLNVIRWIFKLSWIVLGTTAAAEVAGQLALKGLTSQLDDVFVIESQRIVFAENVTIAAFFASLISYFLIKFVPMWGLALIATVITYLGPLVYIQNKEVIDAQLQQYTTKA
Epicoccum_nigrum	RLLSWKNPRATGISFAAAVIFIFATRYLNVIRYIFKATWIVLGCTAAAEVAGQAALKGITSQLDDVFVIESQRIVFAENVTVAAFFASLISYFLIKFVPMWGLALIATIITYLGPLVYIQNKEVIDAQLQQYTTKA
Stagonosporopsis_tanaceti	RLLSWKNPRATGISFAAAVLFIFATRYLNVIRYIFKLTWVVLGTTAAAEVAGQAALKGLTSQLDDVFVIESQRIVFAENVTIAAFFASLISYFLIKFVPMWGLALIATIITYLGPLVYIQNKEVIDAQLQQYTTKA
Phoma_herbarum	RLLSWKNPRATGISFVAAVLFIFAARYLNVIRYIIKLTWIVLGTTAAAEVAGQAALKGLTSQLDDVFVIESQRIVFAENITIAAFFASLISYFLIKFVPMWGLALIATVTAYIGPLVYIQNKEVIDAQLQQYTTKA
Ascochyta_rabiei	RLLSWKNPRATGISFAAAIVFIFAARYLNVIRYIFKLTWVVLGTTAAAEVAGQLALKGLTSQLDDVFVIESQRIVFAENVTIAAFFASLISYFLIKFVPMWGLALIATLVTYLGPLVYLQNKEVIDAQLQQYTTKA
Rhytidhysteron_rufulum	RLLSWKSPRATAISFILSVLFIFAARYLNIVRYIFKALYITLGLTTSAEVLGKVTFTGLTTQFDDAFIIEFQRIIFAENVTIAAFVTTFISYFLIKWLPLWGLTLLGTILVYTLPLIYIQNKEFIDAQLGEYTHKA
Venturia_inaequalis	RLLSWKNPRATAITFALNIALIFAGRYLNVVRYAFKALYLVFGITASAELLGKAVLDGLATRLDDVFVIEFQRVLFAENITVLAFVSAFGSYFLIKFVPLWGLTLISTLAVYLGPLIYIQNKEIIDAQLGEYTKKA
Venturia_aucupariae	RLLSWKNPRATAITFALNIAFIFAGRYLNVVRYAFKALYMVFGITAGAEILGKAVLDGLATRLDDVFVIEFQRVLFAENITVLAFVSAFVSYFLIKFVPLWGLSLITTLAVYLGPLIYIQNKEVIDAQLGEYTKKA
Venturia_effusa	RLLSWKNPRATAITFALNIAFIFAARYLNVVRYAFKALYMVFGITAGAEILGKAVLDGLATRLDDVFVIEFQRVLFAENITVLAFVSAFGSYFLIKFVPLWGLALISTLALYLGPLVYIQNKELIDAQLGEYTKKA
Venturia_carpophila	RLLSWKNPRATAITFAINIAFIFAARYLNVVRYAFKALYMVFGITAGAEILGKAVLDGLATRLDDVFVIEFQRVLFAENITVLAFVSAFVSYFLIKFVPLWGLALISTLAVYLVPLIYIRNKEVIDAQLGEYTKKA
Venturia_asperata	RLLSWKNPRATAITFAINIAFIFAARYLNVVRYAFKALYMVFGITAGAEILGKVALDGLATRLDDVFVIEFQRVLFAENITVLAFVSAFVSYFLIKFVPLWGLTLISTLAVYLVPLIYIQNKEVIDAQLGEYTKKA
Venturia_pyrina	RLLSWKNPRATAITFALNIAFIFAGRYLNVVRYAFKALYMIFGITAGAEILGKAVLDGLATRLDDVFVIEFQRVLFAENITGLAFISAFVSYFLIKFVPLWGLTLISTLALYLGPLIYVQNKEVIDAQLGEYTKKA
Verruconis_gallopava	RLLSWKNPRATTITFLATVAFIFAARYLNVIRYTFKALYLVLGVTAASEVAGRLVLEGIATKLDDVFVIEFQRVVFAENITFLAFISSFISYWLIKWLPLWGLALIFDVFLFIGPLIYIKNQAIIDQHLSEYTKKA
Ochroconis_constricta	RLLSWKNPRATGITFAATILFIFAARYLNIVRYAFKVLYMVLGITAAAEIAGKTVLDGIATKLDDAFVIEFQRVIFVENLTLLAFTSSFIAYWLIKFVPFWGLALIFNTFLFLGPLVYLQNKEFIDAHLNEYTKKA
Cryomyces_antarcticus	SLLSWENPRATAVSFLTTILCIFAARYVPVLRYAFKGLYIVLGAAAAAEVAGKAILRGLVSQLEDVFVIEFQRILFAENVTIAAFTVTLVSYFLVKFVPKWGLALIGTSVLYLAPLVYLQNRETINAQLQQYTEKA
Neoscytalidium_dimidiatum	RLLSWKNPRATSISFACSVLFIFAARYVNIVRYIFKLLWVTLGCTAFAEVAGKAALHGLATQTDDLFVIEFQRVVFVENISAACFVASFISYWLIKWLPLWGLSLIATLIIYLGPLIYIQNKELIDEQLDEYSHKA
Botryosphaeria_dothidea	RLLSWKNPRATSISFACSILLIFAGRYVNIVRYIFKLLWVTLGCTAIAEVAGKAALHGLATQTDDLFVIEFQRVVFVENISATCFLASFISYWLVKWLPLWGLSLIATLVVYLGPLIYIQNKELIDEQLDEYSHKA
Neofusicoccum_parvum	RLLSWKNPRATAISFACSILFIFFARYINIVRYIFKLLWVTLGCTALAEVAGKAALHGLATQTDDLFVIEFQRVVFVENLSGACFIASFISYWLIKWLPLWGLSLIATIVVYMGPLIYIQNKEIIDEQLDEYSHKA
Macrophomina_phaseolina	RLLSWKNPRATGISFACSVLFIFAGRYVNIVRYIFKLLWVTLGCTALAEVAGKATLHGLATQTDDLFVIEFQRVVFVENISSACFLASFISYFLIKWLPLWGLSLIATIVVYMGPLIYIQNKEEIDEYLDEYSSKA
Diplodia_seriata	RLLSWRNPRATAISFACSVLFIFTARYVNIVRYIFKLLWVTLGCTAFAEVAGQAALHGLATQTDDLFVIEFQRVVFVENISAACFVASFISYFLIKWLPLWGLGLIATILIYMGPLVYIQNKEYIDEQLNEYSSKA
Diplodia_scrobiculata	RLLSWRNPRATAISFACSVLFIFTARYVNIVRYIFKLLWVTLGCTAFAEVAGQAALHGLATQTDDLFVIEFQRVVFVENISATCFVASFISYFLIKWLPLWGLGLIATILIYMGPLVYIQNKEYIDEQLNEYSSKA
Diplodia_sapinea	RLLSWRNPRATAISFACSVLFIFTARYVNIVRYIFKLLWVTLGCTAFAEVAGQAALHGLATQTDDLFVIEFQRVVFVENISAACFVASFISYFLIKWLPLWGLGLIATIVIYMGPLVYIQNKEYIDEQLNEYSSKA
Diplodia_corticola	RLLSWRNPRATAISFACSVLFIFTARYVNIVRYIFKLLWVTLGCTAFAEVAGQAALHGLATQTDDLFVIEFQRVVFVENISAACFVASFISYFLIKWLPLWGLGLIATVLVYMGPLVYIQNKEYIDEQLNEYSNKA
Lasiodiplodia_theobromae	RLLSWRNPRATSISFACSVLFIFAARYVNIVRYIFKLLWVTLGCTAFAEVAGQAALHGLATQTDDLFVIEFQRVVFVENLSGACFVASFISYWLIKWLPLWGLSLIATILLYMGPLVYIQNKEYIDEQLDEYSHKA
Phyllosticta_capitalensis	RLLSWKNPRATGISFALSILFIFAARYLNIVRYIFKLLWVILGTTAIAEVAGKSVLNGLASQTDDLFIIEFQRIVFAENITVSACVASFISYFLIKWLPLWGLSLIATTFVYLGPLVYIQNKEYIDEQLQEYSVKA
Phyllosticta_citricarpa	RLLSWKNPRATGISFGLSVLFIFAARYLNIVRYIFKLLWVILGTTAIAEVAGKSLLNGLASQTDDLFIIEFQRIVFAENITVSACVASFISYWLIKWLPLWGLSLIATTLVYLGPLVYLQNKEYIDEQLQEYSVKA
Microsporum_canis	SLLSWERPSATASSFATVVLFIVASRYLPLIRWALKFLYLSLGVTASAEVGGRLLFRGLTSSLEDLFLIEFQRILFVENLTVLAFVTALTSYWLVMFLPLWGLALIATCVAYLAPLVYITNKELIDSQLGDYRAKA
Nannizzia_gypsea	SLLSWERPSATASSFVTVVLFIVASRYLPLLRWTLKFLYLALGVTASAEIGGRFIFRGLTSSLEDLFIIEFQRILFVENLTVSAFITALVSYWLVMFLPLWGLALIATCAAYLAPLVYITNKEFIDNQLGDYRAKA
Trichophyton_rubrum	SLLSWERPSATASSFATVVLFIVASRYLPLIRWTLKFLYLALGVTASAEIGGHFLFRGLTSSLEDLFLIEFQRILFVENLTVSAFVTALVSYWLVMFLPLWGLALIATCVAYLAPLVYITNKEFIDSQLGEYRAKA
Trichophyton_verrucosum	SLLSWERPSATASSFATVVLFIVASRYLPLIRWTLKFLYLALGVTASAEIGGHFLFRGLTSSLEDLFLIEFQRILFVENLTVSAFVTALVSYWLVMFLPLWGLALIATCVAYLAPLVYITNKEFIDSQLGEYRAKA
Trichophyton_benhamiae	SLLSWERPSATASSFATVVLFIVASRYLPLIRWTLKFLYLALGVTASAEIGGHFLFRGLTSSLEDLFLIEFQRILFVENLTISAFVTALVSYWLVMFLPLWGLALIATCVVYLAPLVYITNKEFIDSQLGEYRAKA
Trichophyton_mentagrophytes	SLLSWERPSATASSFATVVLFIVASRYLPLIRWTLKFLYLALGVTASAEIGGHFLFRGLTSSLEDLFLIEFQRILFVENLTVSAFVTALVSYWLVMFLPLWGLALIATCVAYLAPLVYITNKEFIDSQLGEYRAKA
Trichophyton_tonsurans	SLLSWERPSATASSFATVVLFIVASRYLPLIRWTLKFLYLALGVTASAEIGGHFLFRGLTSSLEDLFLIEFQRILFVENLTVSAFVTALVSYWLVMFLPLWGLALIATCVAYLAPLVYITNKEFIDSQLGEYRAKA
Onygena_corvina	SLLSWERPAATASSFISVVLFIVASRYLPLIRWALKFLYLALGVTASAEIGSRVVFRGLNRSLEDICLIEFQRILFVENLTIAAFVTALASYWLVMFLPLWGLALLATCVVYLAPLIYITNKEFIDTQLGDYRAKA
Basipetospora_chlamydospora	ELLSWNNPRASAIAYAGIVSMILAARYLDILRWGLKLAWMALGTTVLAEVAGQTVLNGIASQIGDVFVIEAQRILFAENISAAAFVSAFISYYLVKIVPYWGLAIIGTTVAFLVPLIYTSNQELIDYQIGDYSTKA
Corollospora_maritima_TSA	ELLAWNNPRASAVAYATIISFIFAVRYLDVIRWGFKLTWMVLGTTIAAEVAGKLVMYGLATQIGDVFVIEAQRIVFAENVSIAVFIGAFISYFLVKVVPYWGLAVIGTTAAFFVPLIYTSNQELIDSQIGDYSAKA
Chaetomium_cochliodes	ELLSWRNPRASAIAYASLVSLIFSVRYLDVLRYTFKLTWMVLAITVAAEIAGKALINGFATQIGDVFVIESQRVLFAENVSVAAAFAAFISYYLVKIVPYWGLAVIATTVVFFTPLIYTTNQELIDTQLGDYTAKA
Chaetomium_globosum	ELLSWRNPRASAIAYASLVSLIFSVRYLDVLRYTFKLTWMVLAITVAAEIAGKALINGFATQIGDVFVIESQRVLFAENVSVAAAFAAFISYYLVKIVPYWGLAVIATTVIFFTPLIYTTNQELIDTQLGDYTAKA
Madurella_mycetomatis	ELLSWRNPRASAIAYATIVSIIFSVRYLDVFRWAFKLTWMVLAITIAAEVAGKGLMSGFTTQIGDVFVIESQRVLFAENVSAAAALAAFISYYLVKIVPYWGLAVIATTVVFFAPLIYMTNQELIDDQLGDYTAKA
Myceliophthora_thermophila	ELLSWRNPRASAIAYASIITLIFSVRYLDIIRWAFKITWMVLGITIAAEVTGKVLLNGFATQIGDVFVIESQRVLFAENVSAAAALAAFISYYLVKIVPYWGLAVIATTVIFFTPLIYTTNQELIDNQLGDYTAKA
Thielavia_terrestris	ELLSWRNPRASAIAYAAVVSLIFSVRYLDILRWGFKLTWMVLGITIAAEVVGKALLHGFTTQIGDVFVIESQRVLFAENVSAAAALAAFISYYLVRIVPYWGLAVIATTVVFFAPLVYTTNQEFIDSQLGDYTAKA
Podospora_anserina	ELLSWKNPRASGIAYLTIVSFIFSVRYLDVLRWGLKLTWMALGVTIAAEIAGKAILNGFATQIGDVGVIESQRILFAENISAAIALGAFISYYLVKVVPYWGLALIATSVIFFAPLIYTTNQELIDSQIGDYTAKA
Podospora_comata	ELLSWKNPRASGIAYLTIVSFIFSVRYLDVLRWGLKLTWMALGVTIAAEIAGKAILNGFATQIGDVGVIESQRILFAENISAAVALGAFISYYLVKVVPYWGLALIATSVIFFAPLIYTTNQELIDSQIGDYTAKA
Magnaporthe_oryzae	ELLSWNNPRASGIAYASIVTFIFAVRYLDVIRWSLKLSGYVLATTVAAEVVGKAILTGFASQTGDVFCIESQRILFAENVSAVACVGALISYWLVKIVPYWGLALMTTTLAFVAPLIYKTNKEMIDEHLGDCTAKA
Magnaporthe_grisea_DS0505	ELLSWNNPRASGIAYASIVTLIFAVRYLDVIRWSLKLSGYVLATTVAAEVVGKAILTGFASQTGDVFCIESQRILFAENVSAVACVGALISYWLVKIVPYWGLALMTTTLAFVAPLIYKTNKEMIDEHLGDYTAKA
Pyricularia_pennisetigena	ELLSWNNPRASGIAYASIVTFIFAVRYLDVIRWSLKLSGYVLATTVAAEVVGKAILTGFASQTGDVFCIESQRILFAENVSAVACVGALISYWLVKIVPYWGLALMTTTLAFVAPLIYKTNKEMIDEHLGDYTAKA
Pseudohalonectria_lignicola	ELLSWNNPRASAIAYASIVTVIFAVRYLDVLRWGLKLTWMILATTVAAEVVGKTLLTGFASQIGDAFIIESQRILFAENVSAAACVGAFLSYYLVKIVPYWGLTLIFTTIAFIAPLVYKSNKELIDSQLGDYTAKA
Ophioceras_dolichostomum	ELLSWNNPRASGIAYASIVTLIFAVRYLDVLRWGLKLTWMVLATTVAAEVVGKTILTGFASQLGDVFVIEAQRIVFAENVSAAACVAAFLSYYLVKIVPYWGLALMATTIAFIAPLIYKSNKELIDHYLGDYTAKA
Cryphonectria_radicalis	ELLSWKNPRASAIAYLSTVSLIFAVRYLDVLRWGFRLTWMVLGITIAAEVAGKLVMTGLASQIGDVIVIESQRILFAENISTTVCISAFLSYWLVKIVPYWGLALLTTTLLFTVPLIYTTNQELIDHHLGEYTSKA
Chrysoporthe_austroafricana	ELLSWNNPRASAIAYVSIVTFIFAVRYLDVLRWGFRLTWMVLGTTIAAEVAGKLVMTGLASQIGDVIVIESQRILFAENISTTVCVSAFLSYWLVKIVPYWGLALLATTLLFTVPLVYTTNQELIDHHLGEHISKA

```
Coniella_lustricola                          ELLSWNNPRASALAYLSTVAFIFAVRYLDVLRWGFRLTWMVLGVTIAAEVAGKLVMAGLASQIGDVVVIESQRILFAENISTTVAVSAFLSYWLVKIVPYWGLGLLATTLLFTVPLIYTTNQELIDHHLGDYASKA
Ophiognomonia_clavigignenti_juglandacearum ELLSWKNPRASAIAYFSVVATIFSVRYLDTLRYGFKATWMALGIVIAAEVAGKLVMTSIASQFGDVLVIESQRILFAENISATVALSAFLSYWLVKVVPYWGLALLSTTLLFTVPLIYTSNQELIDHHIGDYTSKA
Juglanconis_oblonga                          ELLSWKNPRVSAIAYFSVVATIVAVRYLDVLRYGFKATWMALGIVIAAEVAGKLAMTSIATQFGDVIVIESQRILFAENISTTVAVSAFFSYWLVKIVPYWGLALLSTTLLFTVPLVYTTNQELIDHHLGDYTSKA
Juglanconis_juglandina                       ELLSWKNPRVSAIAYFSVVATIVAVRYLDVLRYGFKATWMALGIVIAAEVAGKLAMTSIATQFGDVIVIESQRILFAENISTTVAVSAFFSYWLVKIVPYWGLALLSTTLLFTVPLVYTTNQELIDHHLGDYTSKA
Juglanconis_sp_DMW523                        ELLSWKNPRVSAIAYFSVVATIVAVRYLDVLRYGFKATWMALGIVIAAEVAGKLAMTSIAAQFGDVIVIESQRILFAENISTTVALSAFFSYWLVKIVPYWGLALLTTTLLFTVPLVYTSNQELIDHHLGDYTSKA
Valsa_mali                                   ELLSWNNPRASAIAYASTVALIFAVRYLDLLRWGFKLTWMVLGITVAAEVAGKTIMTGIASQIGDVLVIEAQRIVFAENISLTVCLSAFISYWLVKIVPYWGLALIATTLLFTAPLIYTSNQELIDHHLGDYTSKA
Valsa_malicola                               ELLSWNNPRASAIAYASTIAFIFAVRYLDLLRYGFKLTWMVLGVTVAAEVAGKTIMTGLASQIGDVLVIETQRIVFAENISLTVCLSAFISYWLVKIVPYWGLALIATTLLFTAPLIYTSNQELIDHHLGDYTSKA
Valsa_sordida                                ELLSWNNPRASAIAYASTIAFIFAVRYLDLLRWGFKLTWMVLGVTVAAEVAGKTVMTGLASQIGDVLVIEAQRIVFAENISVTVCLSAFLSYWLVKIVPYWGLALLATTLIFTVPLVYISNQELIDHHLGDYTSKA
Cytospora_leucostoma                         ELLSWNNPRASAIAYASTVALIFAVRYLDLLRYAFKLTWIVLGVTVAAEIAGKTIVYGLASQIGDVLVIEAQRIVFAENISATVGVSAFLSYFLVKIVPYWGLALIASTLLFSVPLIYTSNQELIDHHIGDYTNKA
Diaporthe_aspalathi                          ELLSWNNPRASAIAYASIITFIFAVRYLDVIRYGFKLTWMVLGITIAAEAAGKAVLKGIASQLGDVLVIEAQRIVFAENISLAVCVSAFVSYWLAKVVPYWGLALLGTTVLFTAPLIYTSNQELIDHHLGDYTSKA
Stenocarpella_maydis                         ELLSWNNPRASAIAYASIIAFIFSVRYLDVIRWGFKLTWMILGITIAAEAAGKTVMTGIASQIGDVLVIEAQRIVFAENISLAVCVSAFISYWLVKVVPYWGLALIGTTVLFTAPLIYTSNQELIDHHLGDYTHKA
Diaporthe_longicolla                         ELLSWRNPRASAIAYASIIAFIFSVRYLDVIRWGFKLTWMILGITIAAEAAGKAVMTGIASQIGDVLVIESQRIVFAENISLAVCVSAFISYWLVKVVPYWGLALIGTTVLFTAPLIYTSNQELIDHHLGDYTSKA
Diaporthe_ampelina                           ELLSWRNPRASAIAYASIIAFIFSVRYLDVIRWGFKLTWMILGITIAAEAAGKAVMTGIASQIGDVLVIESQRIVFAENISLAVCVSAFISYWLVKVVPYWGLALIGTTVLFTAPLIYTSNQELIDHHLGDYTSKA
Diaporthe_helianthi                          ELLSWRNPRASAIAYVSIVAFIFSVRYLDVIRWGFKLTWMILGTTIAAEATGKAVMTGIASHIGDVLVIESQRIVFAENISLAVCVSAFISYWLVKVVPYWGLALIGTTVLFTAPLIYTSNQELIDHHLGDYTSKA
Phaeoacremonium_minimum                      ELLSWNNPRASAIAYAAIVSFIFAVRYLDVLRWGFKLTWMVLGVTIAAELAGKTVMTGIASQIGDVAVIESQRILFAENISVSVCVSAFLAYWLVKIVPYWGLALIAATTLLFTVPLIYTSNQELIDQQLGDYTAKA
Didymobotryum_rigidum                        ELLSWNNPRASAIAYASIVSFIFAARYLDVIRYTFKLTWMALAVTVAAEVVGKMILNGLTTQIGDVFVIEAQRILFAENVSAAAAVAAFLSYYLVKVVPYWGLAVIATTVTFFAPLIYATNQELIDSQVGDYTAKA
Hypoxylon_sp_CI_4A                           ELLSWNNPRASSIAYASVVSLIFVARYLDVVRYAFKLTWISLGITVLAEVVGKTLLNGLVTQIGDVFVIESQRVLFAENVSAIAAIGAFLSYYLVKIVPYWGLALIATTLLFFVPLVYATNQELIDHYLGDYTAKA
Hypoxylon_sp_EC38                            ELLSWNNPRASAIAYVTIVSLIFVARYLDVVRYAFKLTWMTLGITVLAEGAGKAILNGLATQIGDVFVIESQRILFAENVSAVAAVGSFLSYYLVKIVPYWGLALIATTLLFFVPLIYTTNQELIDHYIGDYTAKA
Hypoxylon_pulicicidum                        ELLSWNNPRASAIAYVTVVSLIFVARYLDVVRYAFKLTWMTLGITVLAEGAGKAILNGLTTQIGDVFVIESQRILFAENVSAVAAIGSFLSYYLVKVVPYWGLALIATTLLFFVPLIYTTNQELIDQQLGDYTAKA
Annulohypoxylon_stygium                      ELLSWNNPRASAIAYASVVSLIFVARYLDVIRYTFKLTYLALGVTVLAEAAGKATITGLATQIGDVFVIESQRVFFAENVSAVAAFGAFLSYYLVKIVPYWGLALIATTILFFAPLIYTTNQELIDHYLGDYTAKA
Xylaria_sp_JS573                             ELLSWNNPRASAIAYASVVSLIFVVRYLDVVRYAFKLTWMALGITVLAEAAGKALLNGLTSQIGDVFIIEAQRVLFAENVSAAAAVGAFLSYYLVKIVPYWGLALLATTVLFFAPLVYTTNQELIDHHLGDYTAKA
Daldinia_eschscholzii                        ELLSWNNPRASAIAYASIVSAIFVARYLDVIRYTFKLSYMALAVTILAEVAGKAVLNGLTTQIGDAFVIESQRILFAENVSAVAAVGAFLSYYLVKIVPYWGLALISTTLLFFVPLIYSTNQELIDHYLGDYTAKA
Pestalotiopsis_fici                          ELLSWNNPRASGIAFASVVSFIFAVRYLDVIRYAFKLTWMALGVTILAEVAGKTILNGLASQIGDVFVIEAQRVFFAENVSTAVALSAFLSYYLVKIVPYWGLALIATTITFFAPLIYTSNQELIDHHLGDYTAKA
Pestalotiopsis_sp_JCM_9685                   ELLSWNNPRASGIAFASVVGLIFAVRYLDVLRYSFKLTWMALGVTILAEVAGKTILNGLASQIGDVFVIEAQRIFFAENVSTAVAAAAFLSYYLVKIVPYWGLALIATTITFFAPLIYTSNQELIDHHLGDYTTKA
Eutypa_lata                                  ELLSWNNPRASAIAYAAIVSLIFAARYLDVVRYTFKLTWMVLGVTVLSEVAGKAILNGLTTQIGDVVIMEVQRILFAENVSAAAAIGAFISYYLVKIVPYWGLALISTTVVFFAPLIYATNQELIDHYIGDYTAKA
Pseudomassariella_vexata                     ELLSWNNPRASGIAYASIVSLIFAARYLDVLRYTLRLTWMALGVTVLAEVAGKTILNGLATQIGDVFVIEAQRIFFAENVSTAAAIGAFLSYYLVKVVPYWGLALIATTVTFFTPLVYKSNQELIDHHLGDYTSKA
Arthrinium_malaysianum_TSA                   ELLSWNNPRASAIAYASVVSVIFVARYLDLLRYSFKLTWMALGVTVLAEVAGKAILQGLATQIGDVFVIEAQRIFFAENVSGAAAVGAFLSYYLVKIVPYWGLALIATTLVFFVPLIYATNQELIDHYLGDYTAKA
Kretzschmaria_deusta                         ELLSWNNPRASAIAYASVVTLIFAVRYLDIIRYFFKLTYMALAITVLAEVTGKAILHGLASQIGDIFVIEAQRILFAENVSVAAAASAFLSYFLAKIVPYWGLALIGTTVLFLAPLIYTTNQELIDQYVGDYTAKA
Xylaria_polymorpha                           ELLSWNNPRASAIAYASVVTLIIATRYLDITRYVFKLTWMALGTTVLAEATGKAILHGLASQIGDIFVIEAQRILFAENISTAAAVGAFISYFLVKIVPVWGLALIGTTVLFLAPLIYTNNQELIDHYVGDYTHKA
Xylaria_longipes                             ELLSWNNPRASAIAYASVVALIFATRYLDITRYVFKLTWMALGTTVLAEATGKAILSGLATQIGDVFVIEAQRILFAENISTAAAVGAFLSYFLVKIVPFWGLALIGATVLFFVPLIYTTNQELIDHYVGDYTHKA
Xylaria_sp_MSU_SB201401                      ELLSWNNPRASGIAYASIVAVIFAVRYLDIVRYFLKFTWMILGTTVLAEVAGKTILNGLASQIGDVFVIEAQRILFAENVSVTACIGALISYYLVKIVPYWGLALIGTTTLFLAPLLYASNQELIDHYVGDYTAKA
Rosellinia_necatrix                          ELLSWNNPRASAIAYTSVVALILAVRYLDIIRYFFKLTWMTLAVTVLAEGTGKAILHGLASQIGDIFVIEAQRIVFAENVSSAAALSAFFSYFLAKIVPYWGLALLGITVLFMAPLIYATNQELIDHYVGDYTAKA
Magnaporthiopsis_poae                        ELLSWNNPRASAIAYVSIVTLVFSVRYLDVFRYALKLTWITLLTTVAAEVAGKTVLTGVASQIGDVFIIESQRILFVENVSALAFVGSLLAYYLVKFVPYWGLTLIFTTVAFIAPLVYKSNKELIDEQLGDYTAKA
Magnaporthiopsis_incrustans                  ELLSWNNPRASAIAYVSIVTLVFSVRYLDVFRYALKLTWITLLTTVAAEVAGKAVLTGVASQIGDVFIIESQRILFVENISALAFVGSLLAYYLVKFVPYWGLTLIFTTVAFIAPLVYKSNKELIDEQLGDYTAKA
Falciphora_oryzae                            ELLSWNNPRASAIAYVSIVTLVFSIRYLDVVRYGLKLTWITLLTTVAAEVAGKTILTGVASQIGDVFIIESQRILFVENISALAFVGSLLAYYLVKFVPYWGLTLIFTTVAFIAPLVYKSNKEFIDEQLGDYTAKA
Gaeumannomyces_tritici                       ELLSWNNPRASAIAYVSIVTLVFSVRYLDVFRYALKLTWITLFTTVAAEVAGKAVLTGVASQIGDVFIIESQRILFVENISAMAFVGSLLAYYLVKFVPYWGLTLIFTTVAFIAPLVYKSNKEFIDEQLGDYTAKA
Gaeumannomyces_sp_JS_464                     ELLSWNNPRASAIAYVSIVTLVFSIRYLDVVRYALKLTWITLLTTVAAEVAGKTILTGVASQIGDVFVIESQRILFVENISALAFVGSLLAYYLVKFVPYWGLTLIFTTVAFIAPLVYKSNKEFIDEQLGDYTAKA
Magnaporthe_salvinii                         ELLSWNNPRASAIAYASIVTTVFAVRYLDVVRYALKLTWITLLTTVAAEVAGKTVLTGVASQIGDVFIIESQRILFVENVSALAFVGSLLAYYLVKFVPYWGLTLIFTTVAFIAPLIYKSNKELIDEQLGDYTAKA
Microdochium_bolleyi                         ELLSWNNPRASGIAYATVVTLIFAARYLDILRYAFKLTWMTLGVTVLAEVVGKLVLNGLATQIGDVFVIESQRILFAENLSAAAAIGAFISYYLINIVPYWGMALIAATAVFFVPLIYTSNQELIDSQLGDYTAKA
Coniochaeta_hoffmannii                       ELLSWNNPRASAIAYASIVTLIFSIRYLDVFRWAFKLTWMVLGITVAAEVLGKTILSGLASQIGDVFVIEAQRILFAENVSAAAAVTAFISYYLVKIVPYWGLSLIATTTLFFAPLVYKSNQEFIDEHLGDYTAKA
Coniochaeta_ligniaria                        ELLSWNNPRASAIAYATIVTLIFSIRYLDIFRWAFKLTWMVLGITVAAEVAGKTILNGLASQIGDVFVIEAQRILFAENVSAAAATTAFISYYLVKIVPYWGLALIGTTVLFFAPLVYKSNQEFIDEHLGDYTAKA
Verticillium_dahliae                         ELLSWKNPRASAIAYASIVSVIFAVRYLDVLRYAFKLTWMVLGTTVLAELTGKLVLNGLATQIGDVFVIEAQRILFAENVSAAAFLGAFISYYLVKVMPLWGLALAATTTLFVVPLAYTSNQELIDHHLGDYTTKA
Verticillium_longisporum                     ELLSWKNPRASAIAYASIVSVIFAVRYLDVLRYAFKLTWMVLGTTVLAELTGKLVLNGLATQIGDVFVIEAQRILFAENVSAAAFLGAFISYYLVKVMPLWGLALAATTTLFVVPLAYTSNQELIDHHLGDYTTKA
Verticillium_nonalfalfae                     ELLSWKNPRASAIAYASIVSVIFAVRYLDVLRYAFKLTWMVLGTTVLAELTGKLVLNGLATQIGDVFVIEAQRILFAENVSAAAFLGAFISYYLVKVMPLWGLALAATTTLFVVPLAYTSNQELIDHHLGDYTTKA
Verticillium_isaacii                         ELLSWKNPRASAIAYATIVSAIFAVRYLDVLRYAFKLTWMVLGTTVLAELTGKLVLNGLATQIGDVFVIEAQRILFAENVSAAAFLGAFLSYYLVKVMPLWGLALAATTTLFVVPLIYTSNQELIDHHLGDYTTKA
Verticillium_tricorpus                       ELLSWKNPRASAIAYATIVSAIFAVRYLDVLRYAFKLTWMVLGTTVLAELTGKLVLNGLATQIGDVFVIEAQRILFAENVSAAAFLGAFLSYYLVKVMPLWGLALAATTTLFVVPLIYTSNQELIDHHLGDYTTKA
Verticillium_zaregamsianum                   ELLSWKNPRASAIAYATIVSTIFAVRYLDVLRYAFKLTWMVLGTTVLAELTGKLVLNGLATQIGDVFVIEAQRILFAENVSAAAFLGAFLSYYLVKVMPLWGLALAATTTLFVVPLIYTSNQELIDHHLGDYTTKA
Acremonium_furcatum                          ELLSWKNPRASAIAYASIVSFILAARYLDILRYALKLTYMALGTTVLAEITGKLVLRGLATQIGDVFVIETQRILFAENVSAVAFIGAFLAYYLVKVMPLWGLALTATSALFFVPLVYSSNQELIDSQIGDYTTKA
Reticulascus_tulasneorum                     ELLSWNNPRASAIAYVGIVSLIFSARYLDVIRWGFKTTWMVLGVTVLAELAGKLVLNGIATQIGDVFVIEAQRILFAENVSVAAFVAAFISYYLVKIVPYWGLALLGTTLAFMVPLAYTSNQELIDHHLGDYSAKA
Coniochaeta_pulveracea                       ELLSWNNPRASAIAYGAIATFILAVRYLDIIRWGFKLTWMVLGITLAAEIVGKTLLSGLATQIGDVFVIEAQRILFAENISAVTAFAAFLAYYLVKIVPYWGLALIGTTITFFTPLVYKNNQEVIDYQLGDYTAKA
Knoxdaviesia_capensis                        HLVAWENPRDSAIAYVSIVSFIVLVRYLDVIRYGLKLSWMVLGTTVAAEALGQLTLRGLTTQIGDVFAIESQRILFAENVSAAAFVAAFISYYLVKVVPYWALAILATTVAFFVPLIYTSNQELIDSQIGDYTAKA
Knoxdaviesia_proteae                         HLIAWENPRDSAIAYFSIVSFIVLVRYLDVIRYGLKLSWMILGTTVAAEALGKLTLRGLTTQIGDVFAIESQRILFAENVSAAAFVAAFISYYLVKVVPYWALAILATTVAFFVPLIYTSNQELIDSQIGDYTAKA
Albophoma_yamanashiensis                     ELLSWKNPRASAIAYASTVSLILAARYLDVIRWAFKLSWMALAVTVSAEAAGKLLLHGLATQIGDVFVIESQRILFAENLSAAACIAAFISYYLVKLMPYWGLAIVATTVAFFVPLVYTSNQELIDYHLGDYAGKV
Tolypocladium_ophioglossoides                ELLSWKNPRASAIAYASIVSVIFAARYLDVIRWALRLSWMALAVTVSAESAGKLVLHGLATQIGDFFVIESQRILFAENISAAACVAAFISYFLVKLVPYWGLAVIGITVAFFAPLVYTSNKELIDHHLGDYTGKV
Tolypocladium_sp_Sup5_PDA_1                  ELLSWKNPRASAIAYASIVSLIFAARYLDVIRWAFKLSWMALAVTVSAEAAGKLVLHGLATQIGDVFVIESQRILFAENVSAAACVAAFISYYLVKLVPYWGLAIIGTTVAFFVPLVYASNKELIDHHLGDYTGKV
Tolypocladium_paradoxum                      ELLSWKNPRASAIAYASIVSLIFAARYLDVIRWAFKLSWMTLAVTVSAEAAGKLVLHGLATQIGDVFVIESQRILFAENVSAAACVAAFISYYLVKLVPYWGLAIIGTTVAFFVPLIYTTNQELIDHHLGDYTGKV
Tolypocladium_inflatum                       ELLSWKNPRASAIAYVSIVSLIFAARYLDVIRGAFKLSWMALAVTVSAEAAGKLVLHGLATQIGDVFVIESQRILFAENVSAAACVAAFISYYLVKLVPYWGLAIIGTSVAFFVPLIYASNQELIDHHLGDYTGKV
Purpureocillium_lilacinum                    ELLSWKNPRASAIAYASIVSLIFAARYLDVIRWAFKLSWMALAVTVSAEAAGKLVLHGLATQIGDVFVIEGQRILFAENLSAAACVAAFISYYLVKLVPYWGLAVIGTTVAFFVPLIYTTNQELIDHHLGDYTGKV
Tolypocladium_capitatum                      DLLSWKNPRASAIAYASIVSLIFAARFLDVIRWAFKLSWMALAVTVSAEAAGKLLLHGLATQIGDVFVIESQRILFAENVSAAACVAAFISYYLVKLVPYWGLAIIGTTVAFFAPLIYTSNQELIDHHLGEYTGKV
Hirsutella_thompsonii                        ELLSWKNPRASAIAYASVISFIFAVRYLDVIRYAFKMAWMALAVTVSAEAAGKLVLHGLATQIGDVFVIETQRILFAENVSALVGVAAFLSYWLAKLVPYWGLAIIATTVAFFVPLIYTTNQELIDHHLGEYTGKV
Hirsutella_minnesotensis                     ELLSWKNPRASAIAYASIVTSIFVARYFDVIRYAFKLSWMALAVTVSAEAAGKLVLHGLATQIGDVFVIETQRILFAENVSALVGVAAFLSYWLVKLVPYWGLAVIATTVAFLVPLVYTTNQELIDHHLGEYTGKV
Ophiocordyceps_sinensis                      ELLSWDNPRASAIAYVTTVSLIFAARYLDVTRYVFKLSWMALAVTVSAEAAGKLVLHGLATQIGDVFVIEAQRILFAENISAAACVAAFLSYHLAKLVPYWGLAVIATTVAFIVPLVYTSNQELIDHHIGDYTGKV
Cordyceps_sp_RAO_2017                        ELLSWNNPRASAIAYASAITFIFAARYLDVVRWAFKLCWMALAVTVSAEAAGKVVLHGLATQIGDVFVIEAQRILFAENVSAAACVAAFLSYHLAKLVPYWGLAIIATTIAFFAPLVYTSNQELIDHHIGDYTGKV
Ophiocordyceps_polyrhachis_furcata           ELLSWSNPRASAIAYVTIVSFILAARYLDVIRWAFKLSWMALAVTVSAEVAGKLVLHGLATQIGDVFVIEAQRILFAENVSTAACVATFIAYFLLKLVPYWGLAVIATTIAFAVPLTYVSHKEVIDHHLGDYSGKV
Ophiocordyceps_unilateralis                  ELLSWKNPRASAIAYVTIVSFIFAARYLDVIRWAFKLSWMALAVTVSSEVAGKLVLHGLATQIGDVFVIEAQRILFAENVSAAACFATFIAYYLLKLVPYWGLAVIATTIAFAAPLIYVNNQELIDHHLDDYSGKV
Ophiocordyceps_camponoti_rufipedis           ELLSWKNPRASAIAYVTIVSSIFAARYLDVVRWAFKLSWMALAVTVSAEVAGKMALHGLATQIGDVFVIEGQRILFAENPSAAACVAAFIAYHLSKLVPYWGLAVIATTIAFAAPLIYINNQELIDHHLGDYTGKV
```

Pochonia_chlamydosporia	ELLSWKNPRASAIAYASIVSFIFAARYLDVIRWAFKLSFMALAVTVAAEVTGKVVLHGLATQIGDVFVIEAQRVLFVESVSTAACIAAFISYHLVKLVPYWGLAIIATTVAFFVPLVYTSNQELIDQHLGDYTGKV
Metarhizium_majus	ELLSWKNPRASAIAYASIVSFIFAVRYLDVIRWGLKLSFMALAITVAAEVTGKVVLHGLATQIGDVFVIEAQRILFVENVSAAGIAAFISYHLVKLVPYWGLAIIATTVAFFVPLVYTSNQELIDQHLGDYTGKV
Metarhizium_robertsii	ELLSWKNPRASAIAYASIVSFIFAVRYLDVIRWGLKLSFMALAITVAAEVTGKVVLHGLATQIGDVFVIEAQRILFVENVSAAGIAAFISYHLVKLVPYWGLAIIATTVAFFVPLVYTSNQELIDQHLGDYTGKV
Metarhizium_anisopliae	ELLSWKNPRASAIAYASIVSFIFAVRYLDVIRWGLKLSFMALAITVAAEVTGKVVLHGLATQIGDVFVIEAQRILFVENVSAAGIAAFISYHLVKLVPYWGLAIIATTVAFFVPLVYTSNQELIDQHLGDYTGKV
Metarhizium_acridum	ELLSWKNPRASAIAYASIVSFIFALRYLDVIRWGLKLSFMALAITVAAEVIGKVVLHGLATQIGDVFVIEAQRILFVENVSAAGIAAFISYHLVKLVPYWGLAIIATTVAFFVPLVYTSNQELIDQHLGDYTGKV
Metarhizium_album	ELLSWKNPRASAIAYASIVSFIFVVRYLDVIRWGFKLSFMALAITVAAEVTGKVVLHGLATQIADFFVIEAQRILFVENVSVAAGIAAFVSYHLIKLVPYWGLAVIATTVAFFVPLVYTCNQELIDQHIGDYTGKV
Atkinsonella_texensis	ELLSWKNPRASAIAYTSIVSLIVAVRYLNVIRWTFKLSFMALAATVGAEVAGKVVFHGIVTQIGDVFVIEAQRILFVENISAAACLASFLSYHLIKLVPYWGLAIIATTIGFFVPLVYTSNQELIDHHLGDYTGKV
Balansia_obtecta	ELLSWKNPRASAIAYVSIVSLIVALRYLDVIRWTFKLSSMALAATVGAEVAGKVVFHGIATQIGDVFVIEAQRILFVENISAAACLASFLSYHLIKLVPYWGLAIIATTIGFFVPLIYTSNQELIDHHLGDYTGKV
Atkinsonella_hypoxylon	ELLSWKNPRASAIAYVSIVSLIFAVRYLDVIRWTFKLSSMALAATVGAEVAGKVVFHGITTQIGDIFVIEAQRILFVENISAAACLASFLSYHLIKLVPYWGLAIIATTIGFFVPLVYTSNQELIDHHLGDYTGKV
Claviceps_fusiformis	ELLSWKNPRASAIAYASIVSFIFVARYLDVLRWALKLSSMALSVTVAAEVAGKAVLHGLATQIGDVFVIEAQRILFVENISAAACVTAFLSYHLVKLVPYWGLAIIATTVAFFVPLVYTTNQELIDHHLGDYTGKV
Epichloe_typhina	ELLSWKNPRASTIAYASIVSFIFVARYLDVIRWALKLSSMALSVTVGAEIAGKAVLHGLATQIGDVFVIEAQRILFVENVSAAACVTAFLSYHLVKLVPYWGLAIIATTVAFFVPLVYASNQELIDHHLGDYTGKV
Epichloe_amarillans	ELLSWKNPRASTIAYASIVSFIFVARYLDVIRWALKVSSMALSVTVAAEIAGKAVLHGLATQIGDVFVIEAQRILFVENVSAAACVTAFLSYHLVKLVPYWGLAIIATTVAFFVPLVYASNKELIDHHLGDYTGKV
Epichloe_festucae	ELLSWKNPRASTIAYASIVSFIFVARYLDVIRWALKLSSMALSVTVAAEIAGKAVLHGLATQIGDVFVIEAQRILFVENVSAAACVTAFLSYHLVKLVPYWGLAIIATTVAFFVPLVYASNKELIDHHLGDYTGKV
Epichloe_glyceriae	ELLSWKNPRASTIAYASIVSFIFVARYLDVIRWALKLSSMALSVTVAAEIAGKAVLHGLATQIGDVFVIEAQRILFVENVSAAACVTAFLSYHLVKLVPYWGLAIIATTVAFFVPLVYASNQELIDHHLGDYTGKV
Epichloe_elymi	ELLSWKNPRASTIAYASIVSFIFVARYLDVIRWALKLSSMALSVTVAAEIAGKAVLHGLATQIGDVFVIEAQRILFVENVSAAACVTAFLSYHLVKLVPYWGLAIIATTVAFFVPLVYASNQELIDHHLGDYTGKV
Epichloe_brachyelytri	ELLSWKNPRASTIAYASIVSFIFVARYLDVIRWALKLSSMALSVTVAAEIAGKAVLHGLATQIGDVFVIEAQRILFVENVSAAACVTAFLSYHLVKLVPYWGLAIIATTVAFFVPLVYASNQELIDHHLGDYTGKV
Epichloe_gansuensis	ELLSWKNPRASTIAYASIVSFIFVFRYLDVIRWALKLSSMALSVTVAAEIAGKAVLHGLATQIGDVFVIEAQRILFVENVSAAACVTAFLSYHLVKLVPYWGLAIIATTVAFFVPLVYASNQELIDHHLGDYTGKV
Periglandula_ipomoeae	ELLSWKNPRASAIAYASIVSFIFVARYLDVIRWAFKLSFMALSVTVTAEVAGKVVLHGLATQIGDLFVIEAQRILFVENISAAACIAAFISYHLVKLVPYWGLAIIATTVAFFVPLIYTSNQELIDHHLGDYTGKV
Aciculosporium_take	ELLSWKNPRASAIAYASIVSFIFVARYLDVLRWGLKLCSMALSVTVAAEVAGKAVLHGLATQIGDVFVIEAQRILFVENISAATCLTAFLSYHLIKLVPCWGLAIIGTTVAFFVPLIYTTNKELIDHHLGDYTGKV
Claviceps_purpurea	ELLSWKNPRASAIAYATIVSCIFVARYLDVIRWAFKLSSMALSVTVGAELVGKAVLHGLATQIGDVFVIEAQRILFVENTSAAALVAAFLSYHLVKLVPYWGLAILAITVAFFGPLIYATNKELIDHHLGDYTGKV
Moelleriella_libera	ELLSWNNPRASAIAYSSIVSFIFAIRYFDVIRWAFKLSSMALAITVAAEVAGKAVLHGLATQIGDVFVIEAQRILFVENTSVAACIAAFISYHLVKLVPYWGLAVIATTVAFFVPLVYKSNQELIDHHIGDYTGKV
Hypocrella_siamensis	ELLSWKNPRASAIAYSSIVSFIFVARYLDVIRWAFKFSSMALTVTVAAEIAGSTLLHGLASQIGDVFVIEAQRILFVENLSIAACVAAFISYHLVKLVPYWGLSIIATTVAFFVPLVYTSNQELIDHHIGDYTGKV
Ustilaginoidea_virens	ELLSWKNPRVSAIAYASIVSFTFAVRYLDLIRWALKLSSVVLAVTVAAEFAGKAVLHGLATQIGDVFVIEAQRILFVENVSALACIAAFISYHLVKLVPFWGLSIIATTVAFFVPLIYKSNKELIDHHIGDYTGKV
Claviceps_paspali	ELLSWKNPRASAIAYASIVSVIFVFRYLDVIRWAFKLSSMALAVTVAAEVAGKTVLRGLATQIGDAFVIEAQRILFVETISAVTCLAAFLSYHLTKLVPYWGLAILATTVAFFVPLIYTTNQELIDHHLGDYTEKV
Lecanicillium_sp_LEC01	ELLSWNNPRASAIAYTSAVSFILLARYLDVIRWAFKLSWMALAVTVAAEAAGKLILNGLATQIGDFIIEAQRILFAENISGAACVAAFLSYYLVKLVPYWGLAIIGTTVAFFVPLVYTTNQELIDSHIGDYTGKV
Drechmeria_coniospora	ELLSWKNPRASAIAYASIVSLIFAARYLDVIRWALKLSWMTLAVTISAEAAGKLVLHGLATQIGDAFIIEGQRILFAENISSAAAMAAFISYFLVKLVPYWALALIGTTVAFLAPLIYSSNKELIDHHLGDYTGKV
Ophiocordyceps_australis	ELLSWNNPRASALAYVSIVSFILAARYLDVIRWALKMSWMALAVTSSAEAAGKVVLHGIASQIGDVFIIEAQRILFAENISVAAFVAAFVLYHLAKLVPYWGLAIMATTVTFFVPLVYKSNQEVIDSQIGDYTGKV
Torrubiella_hemipterigena	ELLSWKNPRATGIAYASIVSLIIAARYLDVIRWAFKLSWISLAVTVAAEAAGKLVLKGLASQIGDVFVIEAQRILFAENISAAACAAAFFSYFLVKLVPYWGLAIIATTIAFFVPLVYTTNQELIDAHIGDYSEKV
Paramyrothecium_roridum	ELLSWKNPRASGIAYAAIVSFIFAARYLDLVRWGFKLTWVALGVTVAAEATGKLILNGLASQIGDVFVIEAQRILFAENLSAAGISAFISYYLIKIVPYWGLALLGTTVLFFVPLIYTTNQELIDHHLGDATAKV
Dactylonectria_macrodidyma	ELLSWNNPRASALAYVTIIALIFSARYLDILRWGFKLSWMVLGVTVLAEVVGKTVLHGLATHIGDVFVIEAQRILFAENISCAAWVAAFVSYFLVKLVPYWGLAVIGTTVVFLVPLVYASNQELIDAQIGDYTGKV
Ilyonectria_destructans	ELLSWNNPRASAIAYVTIVALIFSARYLDILRWGFKLSWMVLGVTVLAEVVGKTVLHGLATHIGDVFVIEAQRILFAENISCAAWVAAFISYFLVKLVPYWGLAVIGTTVVFFVPLVYTSNQELIDAQIGDYTGKV
Ilyonectria_mors_panacis	ELLSWNNPRASAIAYVTIVALIFSARYLDILRWGFKLSWMVLGVTVLAEVVGKTVLHGLATHIGDVFVIEAQRILFAENISCAAWVAAFISYFLVKLVPYWGLAVIGTTVVFFVPLVYTSNQELIDAQIGDYTSKV
Nectria_sp_B_13	ELLSWNNPRASAIAYVTIVALIFSARYLDILRWGFKFSWMILGVTVLAEVVGKAVFNGLATQIGDVFVIEAQRIIFAENISCAAWVAAFISYFLVKLVPYWGLAVISASVIFFVPLVYTSNQELIDTQIGDYTGKV
Neonectria_punicea	ELLSWNNPRASAIAYVTIVALIFSARYLDILRWGFKFSWMVLGVTILAEVVGKAVFNGLATQIGDVFVIEAQRIIFAENISCAAWVAAFISYFLVKLVPYWGLAVISASVIFFVPLVYTSNQEMIDTQIGDYTGKV
Neonectria_hederae	ELLSWNNPRASAIAYVTIVALIFSARYLDILRWGFKFSWMVLGVTVLAEVVGKAVFNGLATQIGDVFVIEAQRIIFAENISCAAWVAAFISYFLVKLVPYWGLAVISTSVIFFVPLVYTSNQEIIDTQIGDYTGKV
Neonectria_ditissima	ELLSWNNPRASAIAYATIVALIFSARYLDILRWGFKFSWMVLGVTVLAEVVGKAVFNGLATQIGDVFVIEAQRIIFAENISCAAWVAAFASYFLVKLVPYWGLAVISASVVFFVPLVYTSNQELIDTQIGDYTGKV
Corinectria_fuckeliana	ELLSWNNPRASAIAYVTIVALIFSARYLDILRWGFKFSWMVLGVTVLAEVIGKAVFNGLATQIGDVFVIEAQRIIFAENISCAAWVAAFASYFLVKLVPYWGLAVISATVVFFVPLIYTTNQEIIDAQVGDYTGKV
Nectria_haematococca	ELLSWNNPRASGIAYATTVSLIFAARYLDVIRWAFKVSWMVLGVTILAEVVGKVVLNGLATQIGDAFVIEAQRIIFAENVSAAAFVAAFISYYLVKLVPYWGLAVIGTTVVFFVPLIYTSNQELIDEQLGDYSGKV
Pseudonectria_foliicola	ELLSWNNPRASAVAIISAVIAFIFSARYLDVIRWGFKLSWMVLGVTIGVEAVGKAVLRGFATQIGDVFVIEAQRIVYAENISAAACVAAFISYYLVKLVPYWGLAVIGTTIAFLTPLVYKNNKEFIDEHLGEYTGKV
Stachybotrys_chlorohalonata	ELLSWNNPRATGLAYGSIVALIFATRYLDIIGWALRLSYLTLGVTVAAEIAGKLVLQGLTSQIGDVFVIEAQRILFAENVSAVTAVAAFLSYHLVSIVPYWGLALIAATMVFFVPLVYTSNQEIIDQQLGDYTKKA
Stachybotrys_chartarum	ELLSWNNPRATGLAYGSIVALIFATRYLDIIGWALRLSYLTLGVTVAAEIAGKLVLQGLTSQIGDVFVIEAQRILFAENVSAVTAVAAFLSYHLVSIVPYWGLALIAATMVFFVPLVYTSNQEIIDQQLGDYTKKA
Stachybotrys_echinata	ELLSWNNPRATGIAYGSIIAFIFAARYLDIISWALRLSYLTLGVTVAAELAGKLVLKGLASQIGDVFVIEAQRILFAENVSAVTALSAFLSYYLIRIVPYWGLAVITTTVVFFVPLVYTSNQELIDQQLGDYSTKV
Sarocladium_oryzae	ELLSWKNPRASAIAYVGIVSLIFAVRYLDILRWGFKLSSWTLFAAVAAEATGKFVLTGLASQIGDVFVIEAQRIVFVENISATACLTAFLSYHLVKLVPYWGLTLIFTTMAFFVPLIYTSNQELIDQHLGDYTGKV
Sarocladium_strictum	ELLSWKNPRASAIAYFGIVTLIFAARYLDILRWGFKLSSWTLFAAVAAEATGKFVLSGLASQIGDVFVIEAQRILFVENISAAACVTAYISYHLVKLVPYWGLALVATTLAFFLPLVYTTNQELIDEHLGDYTGKV
Clonostachys_rosea	ELLSWNNPRASAIALVSTVLAILAFRYVDVARHGLRLAWMALGITVTSEAIGKFLFKGIATQVGDAVVIEAQRILFVESISIATAILAFLSYHLTKIVPYWGLAIIGTTVIAFVAPLIYVSNKEVIDEQLGDYSHKV
Acremonium_chrysogenum	ELLSWNNPRASAIALVSTITLILVARYVDVTRYALKLSWMALGVTVAAEVAGKLLFSGFVTQVGDVVVIEAQRILYVENVSVAAAVFSFIGYHLTKLVPYWGLAVIGTVLAFVLPLIYVTNKELIDHHLGDYTGKV
Gliomastix_tumulicola	ELLSWNNPRASGIALVSIIAFIFTTRYVDLTRYALWLTSAALGITVSLEVAGKFLLKGIATSVGDVFIIESQRILFVENVSLGAAVFAFLSYHLTKLIPYWGLAVIGTVVAFVAPLVYVTNKELIDHHLGDYTGKV
Geosmithia_putterillii	ELLSWKNPRASAISFASILAFIFAARYLDATRWFLRLSWMALGVTVAAEVAGKFVLNGLATQVGDLVVIEAQRILFVESVSAAAAVASFIGYHLTKIVPYWGLAVIGTTILAFTLPLIYVTNQELIDEHLGDYTSKI
Geosmithia_flava	ELLSWKNPRASAVSFVSIITFILAARYLDATRWILRLTWMALGVTVSAELAGKLVLNGLASQVGDLVVIEAQRILYVENVSVVAAISAFIGYHLTKLVPYWGLALIGTILAFTAPLIYVTNQEVIDQQLGDYTSKV
Geosmithia_morbida	ELLSWKNPRASAVSFISIVTFILAARYLDATRWVLRLTWMTLGVTVAAELAGKLVLNGLASQVGDFVVIEAQRILYVENVSVAAAFSAFIGYHLTKLVPYWGLALISTVLAFTLPLVYITNQELIDGQLGDSISKV
Trichothecium_roseum	ELLSWKNPRASAIALGSIISAIFVGRYVDIYRRILQLTWMALGITVSAEAAGKFLLKGLATSMGDLVVIESQRILFVENISFGVAFLSFLTYHLTKIVPYWGLAIMGTLLAFIAPLVYVTNQELIDHHLGDYTGKV
Trichothecium_ovalisporum	ELLSWKNPRASAIALVSIVSAIFIGRYVDIYRRLLQLTWMALGITVSAEAAGKFLLKGIATSVEDIVVIESQRILFVENISLGVAVLSFLTYHLTKIVPYWGLAIMGTLLAFLTPLVYVTNKELIDHHLGDYTGKV
Neurospora_tetrasperma	ELLSWNNPRASAIAYLSIVAAIFSVRYLDMLRLGFKLTWMVLGVTVTAEVAGKTILQGLATQIGDVFVIEAQRILFAENVSGLVAIAAFFAYYLVKIVPYWGLALIATTLAFLGPLVYKTNKEFIDAQLGEYTAKA
Neurospora_crassa	ELLSWNNPRASAIAYLSIVAAIFSVRYLDMLRLGFKLTWMVLGVTVTAEVAGKTVLQGLATQIGDVFVIEAQRILFAENVSGLVAIAAFFAYYLVKIVPYWGLALIATTLAFLGPLVYKTNKEFIDAQLGEYTAKA
Neurospora_africana	ELLSWNNPRASVIAYLSIVAAIFSVRYLDLLRLGFKLTWMVLGVTVTAEVAGKTILQGLATQIGDVFVIEAQRILFAENVSGLVALAAFFAYYLVKIVPYWGLALIGTTLAFLGPLVYKTNKEVIDAQLGEYTAKA
Neurospora_pannonica	ELLSWNNPRASAIAYLSIVAAILSVRYFDVLRLGFKLTWMVLGVTVAAELAGKTILQGLATQIGDVFVIEAQRVLFAENISGLVALAAFFAYYLVKIVPYWGLALIGTTLAFLGPLVYKTNKEVIDAQLGEYTAKA
Neurospora_sublineolata	ELLSWNNPRASAIAYLSIVATILSVRYFDVLRLAFKLTWMVLGVSVAAELAGKTILQGLATQIGDVFVIEAQRVIFAENVSGFVALAAFFAYYLVKIVPYWGLALIGTTLAFLGPLVYKTNKEVIDAQLGEYTAKA
Sordaria_macrospora	ELLSWNNPRASAIAYLSVVAGIFFVRYFDVLRLGFKLTWMVLGATVAAEVAGRTVLQGLTTQIGDVFVIEAQRVLFAENVSGLVALSAFFAYYLVKIVPYWGLALVSTTLAFLGPLVYKTNKEVIDAQLGEYTAKA
Ophiostoma_novo_ulmi	DLLSWNNPRASGIAYASIVTFIFAVRYLDLIRWGLKLTWISLAITVTAEAAGKLVLKGLTSQFGDVFVIESQRIVFAENVSTAACVAAFLSYFLVKIVPYWGLALISTTVVFLAPLIYKTNKELIDSQVGDYTAKA
Ophiostoma_piceae	DLLSWNNPRASGIAYASIVTFIFAVRYLDLIRWGLKLTWITLAITVAAEAAGKLVLKGLSSQFGDVFVIEAQRIVFAENISAAACFAAFLSYFLVKIVPYWGLALISTTVLFLAPLIYKTNKELIDTQVGDYTAKA
Ophiostoma_ips	KLLSWNNPRASGIAYASIVTFIFAVRYLDLIRWGLKLTWIVLGITVAAEAAGKLVLKGLASQFGDVFVIESQRILYVENLSAAACIAAFLSYFLVKVVPYWGLALIGTNVLFLAPLVYKTNQEFIDNQVGDYTTKA
Sporothrix_brasiliensis	ELLSWNNPRASGIAYASIVAFIFAVRYLDVIRWGLRLTWMILGVTVAAETAGKLVLKGLASQLGDVFVIEAQRIVYAENVSAAACVAAFLSYYLVKIVPYWGLALIGTSILFIAPLIYKTNKELIDAQVGDYTAKA
Sporothrix_schenckii	ELLSWNNPRASAIAYLSIVAFIFAVRYLDVIRWGLRLTWMILGVTVAAETAGKTVLKGLASQLGDVFVIEAQRIVYAENVSAAACVAAFLSYYLVKIVPYWGLALIGTSIAFIAPLIYKTNKELIDAQVGDYTAKA
Sporothrix_pallida	ELLSWNNPRASAIAYTSIVAFIFAVRYLDVIRWGLRLTWMLLGVTVAAEIAGKLVLKGLASQLGDVFVIEAQRIVYAENVSAAACIAAFLSYYLVKIVPYWGLALISTTILFIAPLVYKTNKELIDDQVGDYSAKA
Leptographium_lundbergii	NLLSWDNPRSSAIAYTSIVTFIVAGRYLDVVRWAFKLTWIVLGVAVAAEVAGKAVLKGLASQLGDVFVIEAQRVAFAENVTAAAFVGALIAYFLVKFVPYWGLALIFNTVVFFAPLVYKTNKELIDEQLGDYTSKA
Grosmannia_clavigera	NLLSWDNPRSSAIAYASIVTFIVAGRYLDVVRWAFKLTWIVLGVAVAAEVAGKAVLKGLASQLGDMFVIEAQRIAFAENVTAAAFVGALIAYILIKFVPYWGLALIFNTVVFFAPLVYKTNKELIDEQLGDYTTKA

```
Leptographium_procerum              SLLSWDNPRSSGIAYASIVAFIVAGRYLDVIRWAFKLTWIVLGVSVAAEVAGKAVLKGLASQLGDVFVIEAQRVVFAENVTAAAFVGALISYFLVKVVPYWGLALLTTTVVFFTPLIYKTNKELIDEQLGDYTAKA
Leptographium_penicillatum          ELVSWNNPRSSAIAYASIVSLIFAVRYFDVIRWAFKLTWIVLGVTVAAESAGKLVLNGIASQLGDFFVIEAQRIAFAENVTSAAFVAAFLSYYLVKIVPYWGLALIGTTVVFFAPLTYKANKEVIDAQVGDYTAKA
Grosmannia_penicillata              ELVSWNNPRSSAIAYASIVSLIFAVRYFDVIRWAFKLTWIVLGVTVAAESAGKLVLNGIASQLGDFFVIEAQRIAFAENVTSAAFVAAFLSYYLVKIVPYWGLALIGTTVVFFAPLTYKANKEVIDAQVGDYTAKA
Esteya_vermicola                    DLLSWDSPRSSAIAYASIVSFIVAARYLDVIRWAFKLTWITLGVTVAAEIAGKAALNGLASQLGDAFVIEAQRIAFAENVTGAAFVAALLSYYLIKVVPYWGLALIGTTVVFFAPLIYKTNKELIDSQVGDYTAKA
Raffaelea_quercus_mongolicae        NLLSWNDPKSSAIAYTSIVALIVAGRYLDVIRWTFKLTYIALGITVAAEVAGKAVLKGLASQFGDAFIIEAQRILFVENVTAVSFVASLLSYFLVKFVPIWGLALMGTTVVFFAPLIYKTNKELIDAQVGDYTAKA
Raffaelea_quercivora                NLLSWNDPKSSAIAYTSIVALIVAGRYLDVIRWTFKLTYIALGITVAAEVAGKAVLKGLASQFGDAFIIEAQRILFVENVTAVSFVASLLSYFLVKFVPIWGLALMGTTVVFFAPLIYKTNKELIDAQVGDYTAKA
Hawksworthiomyces_lignivorus        SLLSWNNPKMSAIAYASIVSLIVAVRYLDVIRWGLKLSYMLLGITVAAEIAGKLVLNGLASQVGDIFVIEAQRILYVENVSAACVAAFLSYYLVKVVPYWGLTLIGTTILFFAPLVYKTNQELIDQQLGDYTAKA
Fragosphaeria_purpurea              ELLSWNSPRASAIAYASSIVVTILAVKYLDVIRWGFKLTYLLLGVTVAAEIVGKLTINGLASQISDVFVVEAQRILYAENVSAGACVAAFLSYYLVKVVPYWGLALIGTTVAFFAPLIYKTNKELIDEQLGDYTAKA
Sporothrix_insectorum               ELLSWNSPRASAIAYSSIVVTILAVKYLDVIRWGFKLTYLLLGVTVAAEIVGKLTINGLASQISDVFVVEAQRILYAENVSAGACVAAFLSYYLVKVVPYWGLALIGTTVAFFAPLIYKTNKELIDEQLGDYTAKA
Ceratocystiopsis_minuta             ELLSWKNPRATGITYASIVAFILAVRYLDVIRWGLKLTYLLLGATVAAEVAGKTVLKGLASQLSDVFVIETQRIVFAENVSAVAAVAAFLSYYLVKIVPYWGLLLIGTTTLFVGPLVYKTNKEVIDRQLGDYSTKA
Ceratocystiopsis_brevicomis         ELLSWNNPRASGITYVSIVAFILAVRYLDVIRYGLKLTYLLLGATVAAEVAGKTVLKGLTSQLSDVFVIEGQRILFAENVSAAACVAAFLSYYLIKVVPYWGLLLTGTTILFVGPLVYKTNKEVIDHLLGDYSAKA
Graphilbum_fragrans                 DLLSWNNPRASGIAYGSIVSLIIAIRYLDVLRWGLKLTYLSLALTVAAETAGKLVLNGLVSQINDVFIIEGQRILYVENISAAACVAAFLSYFLVKFVPIWGLTLIATTIAFFAPLVYSTNQELIDAQIGDYTTKA
Endocalyx_cinctus                   ELLSWNNPRASAIAYSSIITLIVAGRYLDILRYTFKITWMTLGVTVLAEMVGKMVLNGLASQLGDVFIIESQRVLFAENLTAAAAVGAFLSYYLIKIVPYWGLAVMATTITFFAPLIYATNKEVIDHYVGEYTNKA
Raffaelea_sp_RL272                  SLLAWENPRASAIAYASIVTFILAVRYLDVLRWGFKLTWVLLGISLGAEIAGKLVLNGLASQIGDVLFIESQRIFFAENISAAACIAAFVSYFLVKVVPYWGLALIATSVMFITPLIYASNQELIDAQIGDYTARA
Raffaelea_lauricola                 SLVSWENPRSSAIAYASIVTFILAIRYLDVLRWTFKLTWVALGFTVAAEIAGKLVLNGLASQIGDVFVIEGQRIFFAENVSAAIAVAAFVSYFLVKVVPYWGLALIGTTVAFATPPLYTSNQELIDQQIGNYTARA
Trichoderma_reesei                  HLFSWENPRDSAVAYVSVVASIAAVRYLSLVRWALGLSWMVLATTVAAEVAGKAVLNGLASQVSEAFLVEAQRIVFVENVSSAAFVATLLSSLLIKVLPYWVLAIVATTIAFFAPLIYSRNQEFIDQQLGDYTEKV
Trichoderma_longibrachiatum         HLFSWENPRDSAVAYASVVASIVAVRYLSLLRWALGLSWMVLATTVAAEVAGKAVLNGLASQVSEAFLVEAQRIVFVENVSSAAFVATLLSSLLIKVLPYWVLAIVATTIAFFAPLIYSKNQEFIDEQLGDYTEKV
Trichoderma_citrinoviride           HLFSWENPRDSAIAYTSVVASIVAVRYLNLVNWALGLSWMVLATTVAAEVAGKAVLNGLASQVSEAFLVEAQRIVFVENVSSAAFVATLLSSLLIKVLPYWVLAIVATTIAFFAPLIYSRNQEFIDEQLGDYTEKV
Trichoderma_koningiopsis            HLFSWEKPRDSAIAYVSVVTTIVAVRYLNLLSWTLGLSWMVLATTVAAEISGKLVLNGLASQVSDAFLIEAQRIVFVENVSAAACVAAFISGFLIKVAPYWVLAIIATTIAFFAPLVYSTNQEFIDEQLGDYSGKV
Trichoderma_atroviride              HLFSWEKPRDSAIAYASVVTTIIAVRYLNLLSWTLGLSWMVLATTVAAEISGKLVLNGLASQVSDAFLIEAQRIVFVENISVAACVAAFISGFLIKVAPYWVLTIIATTIAFFAPLVYSSNQEFIDEQLGDYTGKV
Trichoderma_hamatum                 HLFSWEKPRDSAIAYVSVVTTIIAVRYLNLLSWTLGLSWMVLATTVAAEVSGKLILNGLASQVSDAFLIETQRIVFVENVSAAACVAAFISGFLIKVAPYWVLAIIATTIAFFAPLVYASNQEFIDEQLEDYTGKV
Trichoderma_virens                  QLLSWKNPRNSAIAYVSVISTIIAVRYLNLFSWILSLSWMILATTVAAEVTGKLVLNGLASQVSDAFLIETQRIIFVENVSAATCVAAFISGFLIKVLPYWVLAIIGTSIAFLAPLIYSKNQEFIDEQLGDYTGKV
Trichoderma_harzianum               QLLSWKNPRNSAIAYVSVVATIVAVRYLNLFSWTLSLSWMILATTVAAEVAGKLVLNGLASQVSDAFLIESQRIIFVENVSAAACVAAFISGLLIKVLSYWVLAIIGTSIAFFAPLIYSTNQEFIDEQLGDYTEKV
Trichoderma_brevicompactum          HLFSWDNPRNSAIAYVSVVSTIIAVRYLNLFSWALGLSWMVLATTVAAELAGKLVLTGLASQVSDAFLIEAQRIVFVENVSAAACVVAFISGLLIKVVPYWALAIVATTIAFFAPLIYSSNQEFIDEQLGDYTGKI
Escovopsis_weberi                   DLVSWKNPRESAIAYASIVTAIIATHYLPLFYVRWAIKLSWMSLALTIAAEVAGKTIINGLASQTTDVFAIEAQRIFYVESVSSAACAAAFVTYYLIKIVPYWGLAVFATTLAFFAPLIYQSNQELIDQQLGDYTGKV
Escovopsis_sp_AC                    DLVSWKNPRESAIAYASIVTAIIATHYLPLVRWAIKLSWMSLALTIAAEVAGKTIINGLASQTTDVFAIEAQRIFYVESVSSAACAAAFVTYYLIKIVPYWGLAVFATTLAFFAPLIYQSNQELIDQQLGDYTGKV
Escovopsis_sp_TC                    NLVSWKNPRESAIAYASIVTAIIATHYLPIVRWAIKLSWMTLALTIAAEVTGKTVLNGLASQTADLFAIEAQRIFYIENVSSAACVAAFITYYLIKFVPYWGLAILATTLTFFAPLIYKSNQELIDQQLGEYTVKV
Cordyceps_confragosa                ELLSWKNPRNSAIAYGSIVTFILAARYFDIIRWAFKLSWMTLAVTISAEVAGKLVLNGITSQIGDVFIIEGQRILFAENISAIAGLAAFISYFLVKLVPYWGLAILGTTLAFFVPLVYAANKELIDEHLGDYTGKV
Cordyceps_farinosa                  ELLSWKNPRNSAIAYGSILTFILATRYFDIIRWAFKLSWMTLAVTITAEVAGKLVLNGITSQIGDVFVIEGQRILFAENISAVAGLAAFVSYFLVKLVPYWGLAILGTTLAFFVPLVYAANKELIDEHLGDYTGKV
Paecilomyces_hepiali                ELLSWKNPRNSAIAYGSILTFILATRYFDIIRWAFKLSWMTLAVTITAEVAGKLVLNGITSQIGDVFVIEGQRILFAENISAVAGLAAFVSYFLVKLVPYWGLAILGTTLAFFVPLVYAANKELIDEHLGDYTGKV
Cordyceps_militaris                 ELLSWKNPRNSAIAYGSIVTFILAARYLDIIRWAFKLSWMTLAVTISAETAGKALVLSGITSQIGEVFIIEGQRILFAENISAVACVAAFITYFLVKLVPYWGLAILGTTLAFFVPLVYAANKELIDENIGDYTGKV
Cordyceps_brongniartii              ELLSWKNPRNSAIAYGSIITFILATRFFDIIRWAIKLSWMTLAVTITAEAVGKLVLSGITSQIGDVFIIEGQRILYTENISAAACFAAFVSYFLVKLLPYWGLAILGTTLAFFVPLVYAANKELIDEQLGDYTGKV
Beauveria_bassiana                  ELLSWKNPRNSAIAYGSIVTFILATRFFDIIRWAIKLSWMTLAVTITAEAVGKLVLSGITSQVGDVFIIEGQRILYTENISAAACFAAFVSYFLVKLLPYWGLAILGTTLAFFVPLIYAANKELIDEQLGDYTGKV
Beauveria_pseudobassiana            ELLSWKNPRNSAIAYGSIVTFILATRFFDIIRWAFKLSWMTLAVTITAEAVGKLVLSGITSQIGDVFIIEGQRILYTENISAAACFAAFVSYFLVKLVPYWGLAILGTTLAFFVPLVYAANKELIDEQLGDYTGKV
Cordyceps_cicadae                   DLLSWKNPRNSAIAYGSIVTFILATRYFDILRWAIKLSWMTLAVTVTAEAAGKLLLSGIASQVSDAFIIEAQRILYVENISAAACFAAFISYFLVKLVPYWGLAILSTTLAFFVPLVYAANKELIDEHLGDYTGKV
Cordyceps_tenuipes                  DLLSWKNPRNSAIAYGSIVTFILATRYFDILRWAIKLSWMTLAVTVTAEAAGKLVLSGIASQVSDAFIIEAQRILYVENISAAACVAAFISYFLVKLVPYWGLAILSTTLAFFVPLVYAANKELIDEQLGDYTGKV
Isaria_fumosorosea                  DLLSWKNPRNSAIAYGSIVTFILATRYFDILRWAIKLSWMTLAVTITAEAAGKLVLSGIASQVSDAFIIEAQRILFVENISAAACVAAFISYFLVKLVPYWGLAILGTTLAFFVPLVYAANKELIDEHLGDYTGKV
Isaria_farinosa                     ELLSWKNPRNSAIAYGSIVTFILATRYLDIIRWAFKLSWMTLAVTVTAEAAGKLVLSGIASQIGDVFVIEGQRILFAENISAAAFVAAFISYFLVKLVPYWGLAILGTTLAFFVPLVYAANKEVIDEHLGDYTGKV
Lecanicillium_psalliotae            DLLSWKNPRNSAIAYGSIVTLILATRYLDIIRWAFKLSWMTLAVTITAEIAGKLVLSGLASQIGDVFVIEGQRILFAENISAAACVAAFISYFLVKLVPYWGLAIMATTLAFFVPLVYAANKELIDENLGDYTGKV
Lecanicillium_fungicola             DLLSWKNPRNSGIAYGSIVTLILATRYFDIIRWAFKLSWMTLAVTITAEIAGKLVISGLASQVGDVFIIEGQRILFAENVSAAACVAAFISYFLVKLVPYWALAILSTTLAFFVPLVYAANKELIDENLGDYTGKV
Scedosporium_aurantiacum            DLLSWKNPRASGIVYISVISLILAARYLDVIRYFFKLTWVALGTTVALEIVGKVLLQGITSQIGDVFVIEAQRILFAENISAAAFIAAFVSYYLVKVVPYWGLAIIGTTAAFMIPLVYTSNQELIDAQLGDYSAKA
Scedosporium_dehoogii               DLLSWKNPRASAIVYASIITLIFAARYLDVIRYFFKLTWVALGTTVALEVAGRVLLQGITSQIGDVFVIEAQRILFAENISAVAFIAAFVSYYLVKVVPYWGLAIIGTTAAFMIPLVYTSNQELIDAQLGDYSAKA
Scedosporium_apiospermum            DLLSWKNPRASAIVYASIISLIFAARYLDVIRYFFKLTWVALGTTVALEVAGKVLLQGITSQIGDVFVIEAQRILFAENISAGAFIAAFVSYYLVKVVPYWGLAIIGTTAVFMIPLVYTSNQELIDAQLGDYSAKA
Scedosporium_boydii                 DLLSWKNPRASAIVYASIISLIFAARYLDVIRYFFKLTWVALGTTVALEVAGKVLLQGITSQIGDVFVIEAQRILFAENISAGAFIAAFVSYYLVKVVPYWGLAIISTTAVFMIPLVYTSNQELIDAQLGDYSAKA
Lomentospora_prolificans            DLLSWKNPRASAVVYASLVSLIFAARYLDVIRYFFKLTWLALGTTVALEIAGQTVLQGITSQIGDVFVIEAQRILFAENISAAAFVAAFVSYYLVKVVPYWGLAIIGTTAAFMIPLLYTSNQELIDAQLGDYSAKA
Amorphotheca_resinae                SLLSWENPRASGIAFVSVVLFIFAARYLDILRYVFKITWMTLGITVFAEAVGKAILTGLTSQIGDLFVIETQRVVFAENVSAAAFLGAFFSYFLIKVLPFWGLSLLSTFVIFLTPLIYKNNKEVIDHHLGDYSAKA
Oidiodendron_maius                  SLLSWQNPRATGIAFASVVAFIFAVRYLDILRYAFKFTWMTLGITVLAEAAGKTILSGLTSQISDLFVIESQRVVFAENLSAAAFLGAFFSYFLIKVVPFWGLALISTSALFLGPLVYKTNKELIDHNLGDYSTKA
Fungal_sp_EF0021                    SLLSWENPRASGIAYAVTVLFIFAARYLDIIRYTFKLSYIVLFITVLAEGVGKLVFTGLTSQIGDIFIIEAQQIVFAENLSIAAFVSAFLSYHLIKVVPYWGLSLISASVLFLAPLIYKTNQELIDHHLGDYSAKA
Phialocephala_subalpina             SLLSWKNPRASGIAYATVVLFIFAARYLDIIRYAFKLSYMILGITVLAEGVGKALFTGLASQIGDVFIIEAQQIIYAENLSSAAFLGAYLSYYLIKVVPFWGLSLISTSILFLAPLIYKTNQELIDHHVSDYSAKA
Phialocephala_scopiformis           SLLSWENPRASGIAYASVVLFIFAARYLDIIRYTFKISYMVLGITVFAEAVGKALLTGLTSQIGDVFVIEAQQIIFAENLSFAAFLGAFLSYYLIKIVPFWGLSLISTSILFLAPLIYKTNQELIDHHVGDYSAKA
Cadophora_malorum                   SLLSWEHPRASGIAYLATVLFIFAARYLDILRYSFKVTYMILGITVLAEAVGKGLFTGFTSQIGDVFVIEAQQIVFAENLSGAAFIGAFLSYYLIKVVPFWGLSLIGTSVLFLAPLIYKTNKELIDANLGDYSAKA
Cadophora_sp_DSE1049                SLLSWEHPRASGIAYLATVLFIFAARYLDIIRYSFKFTYMVLGITVLAEAVGKGLFTGFTSQIGDVFVIEAQQIVFAENLSGAAFIGAFLSYYLIKVVPFWGLSLIGTSVLFLAPLIYKTNKELIDANLGDYSAKA
Helotiales_sp_F229                  SLLSWEHPRASGIAYLAIVLFIFAARYFDIIRYSFKLTYMVLGITVLAEAVGKALFTGFTSQIGDVFVIEAQQIVFAENLSGAAFIGAFLSYYLIKVVPFWGLSLIGTSVLFLAPLIYKTNKELIDANLGDYSAKA
Rhynchosporium_secalis              SLLSWEHPRASGIAYLAIVSFIFAARYLDIIRYSFKLTYMVLGVTVLAEAVGQAAFTGFTSQIGDVFVIEAQQIVFAENLSGAAFVAAFISYYLIKIVPFWGMSLIATSVLFLTPLIYKTNKQLIDENLGDYSAKA
Rhynchosporium_agropyri             SLLSWEHPRASGIAYLAIVSFIFAARYLDIIRYSFKLTYMVLGVTVLAEAVGQAAFTGFTSQIGDVFVIEAQQIVFAENLSGAAFVAAFISYYLIKIVPFWGMSLIATSVLFLTPLIYKSNKQLIDENLGDYSAKA
Rhynchosporium_commune              SLLSWEHPRASGIAYLAIVSFIFAARYLDIIRYSFKLTYMVLGVTVLAEAVGQAAFTGFTSQIGDVFVIEAQQIVFAENLSGAAFVAAFISYYLIKIVPFWGMSLIATSVLFLTPLIYKTNKQLIDENLGDYSAKA
Marssonina_coronariae               SLLSWEHPRASGIAYLATVVFIFCARYFDIIRYIFKATYMTLGITLLAEVLGKALFNGLTTSQIGDVFVIEAQQIVYAENISAAAFLGAFLSYYLIKVVPFWGLSLIGTSVLFLGPLIYKTNQELIDENLGDCSAKA
Marssonina_brunnea                  SLLSWENPRASGIAYLATVLFIFAARYLDIIRYSFKLTYMTLGITVLAEVLGKALINGLTSQIGDVFVIEAQQIVFAENISGAAFIAAFISYYLIKVVPFWGLSLIATSVLFLVPLIYTTNKEFIDENLGDYSAKA
Cairneyella_variabilis              SLLSWNNPRASGIAYLSVVLFIVATRYLDILRYSFKFTYMVLFVTVLAEIVGKALFTGFTSQLGDVFVIESQRIIFAENVSGAAFVGALLSYYLIKIVPYWGLSLIATSVLFLAPLIYKTNQELIDHHLGEYSIKA
Pezoloma_ericae                     SLLSWNNPRASGIAYLSIVLFIFAARYLDILRYAMKLTWVTLAITVAAEAAGKALFTGFTSQMGDVFVIESQRIVFAENLSGAAFVGAFLSYYLIKIVPFWGLTLISTSVIFLTPLIYKANQELIDHHIGEYAAKA
Meliniomyces_variabilis             SLLSWNNPRASGIAYLSIVLFIFAARYLDVLRYAMKLTWVTLAVTVAAEVTGKALFVGFTSQMGDVFVIESQRIVFAENVSAAAFVGAFLSYYLIKIVPFWGLTLISTSVLFLSPLIYKTNQELIDHHLGEYAAKA
Meliniomyces_bicolor                SLLSWNNPRASAIAYLSVVLFIFAARYLDVLRYAMKLTWVTLAITVAAEVTGKALFTGFTSQMGDLFVIESQRIVFAENVSAAAFVGAFLSYYLIKIVPFWGLTLISTSVLFLSPLIYKTNQELIDHHLGEYSAKA
Calycina_herbarum                   SLLSWNNPRASGIAFASIVSFIFAARYLDILRYTFKVTYMTLAVTVLAEIAGKSILTGLTSQTADVFVIESQRILFVENVSAAAFLGSFISYFLIKFVPFWGLTLLTTTIAFIAPLVYKTNKEVIDHYLGDYSHKA
Hymenoscyphus_fructigenus           SLLSWNNPRASGIAFASIVSFIFAARYLDVLRYTFKVTYMTLAVTVLAEIAGKSILTGLTSQTADVFVIESQRILFVENVSAAAFLGSFISYFLIKFVPIWGLTLLATTITFIAPLVYKTNKEVIDHYLGDYSHKA
Hymenoscyphus_scutula               SLLSWNNPRASGIAFASIVAFIFAARYLDVLRYTFKVTYMTLAVTVLAEIAGKSILTGLTSQTADVFVIESQRILFVENVSAAAFLTTFISYFLIKFVPFWGLTLLATTIAFVAPLVYKTNKEVIDHYLGDYSHKA
Hymenoscyphus_fraxineus             SLLSWDNPRASGIAYSSIVLFIFAARYLDILRYTFKVTYMTLAVTLLAEVAGKSILHGLTSQTSDVFVIESQRIVFVENVSLAAFFGSFISYFLIKFVPIWGLTLLATTIAFAGPLIYKTNKEVIDHYLGDYSHKA
```

```
Hymenoscyphus_repandus            SLLSWNNPRASGIAYASIVAFIFAARYLDILRYAFKVTYLTLSVTVLAEVIGKLVIQGLTSQIGDVFVIESQRIVFAENVSAAALIGSFISYYLIKVVPFWGLSLIASTVLFIAPLIYKTNKELIDHYLGDYSHKA
Phialophora_cf_hyalina            SLLSWNNPRASAIAYGSVVLFIFAARYLDILRYTFKVTWMTLGVTVLAEIAGKAVLTGLTSQLGDVFVIESQRILFAENVSAAAFVAAFISYFLIKVVPIWGLSLIATSVLFLAPLIYKTNKELIDEHLGEYSSKA
Gamarada_debralockiae             SLLSWKNPRASGIAFASVVLFIFAARYFNILRYGFKLTWMTLGVTIAAEVAGKMVLQGLSSQTGDLFVIESQRIVFAENVSTAAFLGAFFSYYLIKIVPLWGLALIFTSILFLAPLIYKTNKELIDHHIGDYSAKA
Sclerotinia_homoeocarpa           SLLRWDNPRASGIAYASIVTFIFAARYLDILRYSFKVTYMTLFVTVAAEGLGKLIFRGLTSQLGDVFVIESQRILFAENVSLAAFLGSFISYYLIKVVPFWGLTLIATSVLFLAPLIYKTNKEFIDHHLGEYASKA
Rutstroemia_sp_NJR_2017a          SLLAWDNPRASGIAYVSIITFIFAARYLDILRYSFKVTYMTLFVTVAAEGLGKLLFRGLTSQLGDVFVIESQRILFAENVSLAAFFGSFLSYYLIKVVPFWGLTLIATSVLFISPLVYKTNKEFIDHHLGEYASKA
Rutstroemia_echinophila           SLLSWNNPRASAIAYVSVVTFIFAARYLDVLRYGFKVTYMALFVTVLAEGLGKMLFHGLASQLGDVFVIEFQRVLFAENVSLAAFFGAFLSYHLIKIVPFWGLSLIGTSVLFVSPLVYKTNKEFIDHHVGEYASKA
Rutstroemia_sydowiana             SLLSWDNPRASAIAYASAVTFIFAARYLDVLRYSMKVTYMALFVTVLAEGTGKMLFHGLASQLGDVFVIEFQRILFAENVSLAAFFGAFLSYHLIKIVPFWGLSLIATSVLFVSPLVYKTNKELIDHHVGEYASKA
Botrytis_cinerea                  NLLSWENPRASGIAYLSTVAFIFAARYLDILRYSFKITYMTLFVTVLAEGLGKLAFHGLTSQLGDVFVIESQRVLFAENVSLTALIGSFISYYLIKLIPFWGLSLIATSVLFISPLVYKTNKELIDNTLGEYAHKA
Botrytis_paeoniae                 NLLSWENPRASGIAYLSTVAFIFAARYLDILRYLFKVTYMTLFVTVLAEGLGKLAFHGLTSQLGDVFVIESQRVLFAENVSLTALIGSFISYYLIKLIPFWGLTLIATSVLFISPLVYKTNKELIDNTLGEYAHKA
Sclerotinia_glacialis             NLLSWNNPRASGLAYLSTVSFIFAARYLDILRYSFKVTYMALFVTVLAEALGKLAFHGLTSQLGDVFVIESQRILFAENLSITALIGSFISYYLIKLIPFWGLSLVFTSVIFICPLIYKTNKELIDNLLGEYAHKA
Sclerotinia_sclerotiorum          NLLSWNNPRASGIAYLSTVAFIFAARYLDILRYSFKVTYMALFVTVLAEALGKLAFHGLTSQLGDVFVIESQRILFAENLSLTALIGSFISYYLIKLIPFWGLSLIFTSVIFICPLIYKTNKELIDNLLGEYAHKA
Ciborinia_camelliae               SLLSWDNPRASGIAYLSTVAFIFAARYLDILRYSFKVTYMALFVTVLAEGLGKMVFHGLTSQLGDVFVIESQRILFAENVSLAAFLGAFVSYYLIKLIPFWGLSLIATSVLFISPLIYKTNKEFINHHLGEYAHKA
Myriosclerotinia_duriaeana        SLLSWDNPRASGIAYLSTVAFILAARYLDILRYSFKLTYMSLFVTVLAEAFGKLLFHGLASQLGDVFVIESQRILFAENVSLAAFLGSFLSYYLIKLMPFWGLSLIATSVLFVSPLIYKTNKEFIDYHLGEYAHKA
Myriosclerotinia_curreyana        SLLSWDNPRASGIAYLSTVAFILAARYLDILRYSFKLTYMSLFVTVLAEAFGKLLFHGLASQLGDVFVIESQRVLFAENVSLAAFLGSFLSYYLIKLMPFWGLSLIATSVLFVSPLIYKTNKEFIDYHLGEYAHKA
Sclerotinia_borealis              SLLSWDNPRASGIAYLSTVAFILASRYLDILRYSFKVTYMALFVTVLAEGLGKVLFHGLASQLGDVFVIESQRILFAENMSLAAFFGSFLSYYLIKLMPFWGLSLIATSVLFVCPLVYKTNKEFIDYHIGEYAHKA
Monilinia_fructicola              SLLSWDNPRASGIAYLSIVGFIFAARYLDILRYSFKVTYMTLFVTVLAEGLGKLLFHGLTSQLGDVFVIESQRILFAENVSLAAFFGSFISYYLIKLIPFWGLTLIATSVLFVSPLIYKTNKEVIDNLLGEYAHKA
Monilinia_laxa                    SLLSWDNPRASGIAYLSIVGFIFAARYLDILRYSFKVTYMALFVTVLAESLGKLLFHGLTSQLGDVFVIESQRILFAENVSLAAFFGSFTSYYLIKLIPFWGLTLIATSVLFVSPLIYKTNQQFIDNLLGEYAHKA
Monilinia_fructigena              SLLSWDNPRASGIAYLSIVGFIFAARYLDILRYSFKVTYMSLFVTVLAEGLGKLLFHGLTSQLGDVFVIESQRILFAENVSLAAFFGSFISYYLIKLIPFWGLTLIATSVLFVSPLIYKTNKEFIDNLLGEYAHKA
Pezicula_radicicola               SLLSWNNPRASGIAYLSIVTFIFAARYFSILRYGFKLTWMTLGITVLAEIAGKALFHGFTSQLGDVFVIESQRIVFAENVSIAAFFGAFFSYYLIKIVPLWGMTLIATSILFLGPLVYKTNKELIDHHVGDYSAKA
Coleophoma_cylindrospora          TLLSWNNPRASGIAYLSTVLFIFAARYLNILRYAFKLTWMTLGVTVLAEVAGKTLFAGFTSQLGDVFVIESQRIVFAENVSVAAFFGSFLSYYLIKIVPLWGMALIGTSVLFLSPLIYKTNKELIDHHVGDYSAKA
Coleophoma_crateriformis          TLLSWNNPRASGIAYLSTVLFIFAARYLNILRYAFKLTWMTLGVTVLAEVAGKTLFAGFTSQLGDVFVIESQRIVFAENVSVAAFFGSFLSYYLIKVVPLWGMALISTSVLFLGPLVYKTNKELIDHHVGDYSAKA
Glarea_lozoyensis                 SLLSWNNPRASGIAYASIVTFIFAARYLDILRYAFKLTWMTLGVTVLAEVAGKAVLHGLASQTGDFFVIESQRIVFAENLTVASFLGAFISYYLIKFVPFWGLTLISTTVIFISPLIYKTNKETIDHYVGDYSHKA
Articulospora_tetracladia         SLLSWEHPRASGIAYLSTVVFIFAARYLDIIRYAFKATYMILAITVLAEAVGKAVLHGLTSQIGDLFVIEAQQIIFAENLSTAALFAAFLSYYLIKVVPFWGLCLISDTVLFGAPLIYTTNKQLIDTQVGDYSAKA
Chlorociboria_aeruginascens       SLLSWEHPRASGIAYIATVVFIFFARYVDVLRYTFKAAWVVLGITAAAELAGQAVFQGFVSSIGDVFVIEIQRIVFAENVTVAAFLGAFFSYYLIKIVPVWGLALISTSVLFLAPLIYKTNKEFIDHHLGDYSAKA
Ascocoryne_sarcoides              SLLSWNNPRASAIAYTSIVALIFAIRYLDILRYGLKITYVTLGITVAAEATGRILFHGFATQIGDIFVIEAQRILFAENVSVAAFISAFISYYLIKIVPIWGLSLLATTVLFVTPLIYKTNKEIIDTQIGDYSAKA
Raffaelea_arxii                   SLIAWENPRSSAIAYASIIIFILAVRYLDVLRWGFKLTWMLLGISLAGEVIGKLVLNGLTSQIGDVFVIEAQRVFFAENVSAAACFAAFVSYFLVKIVPYWGLALIGTTVIFIVPLIYTSNQELIDEQIADYTARA
Raffaelea_ambrosiae               SLVAWENPRNSAIAYLSIVGFILAVRYLDVLRWGFKLTWIVLGISLAAEVAGKVVLNGLTSQIGDVFVIEAQRIFFAENVSAAVAIAAFVSYFLVKIVPYWGLALIATTVAFIFPLIYTSNQELIDQQLANYSARA
Raffaelea_albimanens              SLVAWENPRNSAIAYVSIITFILAVRYLDVLRWGFKLTWMVLGISLAAEVVGKLVLNGLTSQVGDVFVIEAQRIFFAENVSAGAAVAAFLSYFLVKVVPYWGLALIATTVAFIFPLIYTSNQELIDAQLASYSARA
Podosphaera_xanthii_TSA           SLLSWEHPRASGIAYLFTVFLIFAARYLNVLRYSLKVTYMVLGVTVLAEILGKVLFHGFTSQIRDFFIIEAQQIIFAENLSIAAFLSAFASYFLVKFIPLWGLSLLGTSTLFLGTLVYRVNQDVIDHHLGDYSSKA
Blumeria_graminis_hordei          SLLSWENPRASGIAYLASIFFIFAARYLNFVRYLFKITYVVLGITIMAEVSGKVLLHGFTSQIADLFVIEAQQIIFAENLSIGAFVAALFSYFLVRFVPLWALCLLGTSSLFLGSLFYVSNQEMIDQHLEDYSIKA
Blumeria_graminis_tritici         SLLSWENPRASGIAYLASVFFIFAARYLNFVRYLFKITYVVLGITIMAEVSGKVLLHGFTSQIADLFVIEAQQIIFAENLSIGAFVAALFSYFLVRFVPLWALCLLGTSSLFLGSLFYVSNQEMIDQHLEDYSIKA
Erysiphe_pulchra                  SLLSWEHPRASGIAYITSVLIIFAARYLNIFRYSLKVTYVVLGITLLSEIVGKLLFHGFTSQIGDLFVIQAQQIIFTENLSLAAFISAFISYFLVKFVPIWGLSLLGTSALFLGTLAYKSNQEFIDHNLGDYSVRA
Oidium_neolycopersici             SLLSWEHPRASGIAYITSILIIFAARYLNIFRYSLKVTYVVLGITVLSEIAGKLLFHGFTSQIGDLFVIQAQQIIFTENLSLAAFTSAFISYFLVKFVPIWGLSLLGTSALFLGSLAYKSNQEFIDDNLGDYSVRA
Erysiphe_pisi_Corr                SLLSWEHPRASGIAYITSVLIIFAARYLNIFRYSLKVTYVVLGITVLSEIAGKLLFHGFTSQIGDLFVIQAQQIIFTENLSLAAFIFAFISYFLVKFVPIWGLSLLGTSALFLGTLGYKSNQEFIDQNLGDYSVRA
Oidium_heveae                     SLLSWEHPRASGIAYITSVLIIFAARYLNIFRYSLKVTYVILGITVLSEIAGRLLFHGFTSQIGDLFVIQAQQIIFTENLSLAAFIFAFISYFLVKFVPIWGLSLLGTSALFLGTLAYKSNQEFIDHNIGDYSVRA
Erysiphe_necator                  SLLSWEHPRASGIAYLTSVLIIFATRYLNIFRYSLKVTYVVLGITLLSEITGKLLFHGFTSQIGDVFVIQAQQIIFAENLSLAAFVSAFISYFLVKFVPIWGLSLLGSSALFLGTLAYKSNQEFIDNNIGDYSVRA
Golovinomyces_cichoracearum       SLFSWEHPRASGIAYIVSVLCIFAARYLNIVRYGLKVTYVVLGITVLAEVAGKIILHGFTSQTGDFFVIQAQQIIFAENLSIAAFISAFISYFLVKFVPLWGLSLLGTSTLFMGTLVYKTNQELIDHNVGDYSVKA
Chalaropsis_thielavioides         YLLSWNNPRDSGIAYGSIVAGIILIRCIDVLRYAFKLTYLTLAVTVAAEVLGKLVINGLTSQASDLISTESQKIIYVENVSGAALIGAFLSYHMVKIVPYWALAILTVSAIFFGPLVYHENQEAIDAQIGNFSTKA
Thielaviopsis_basicola            YLLSWNNPRDSGIAYGSIVTAIILFRCIDVLRYAFKLTYLTLAITVTAEVLGKLAINGLTSQASDLISTESQKIIYVENISGAALIGAFLSYHLVKVLPYWALGIMTVSAIFFGPLVYRENQEVIDAQIGNLSTKA
Ambrosiella_xylebori              YLLSWNNPRDSGIAYGSIVASIILIRCLDVLRYAFKLTYLTLAVTVAAEILGKLTINGLTSQASDLIATEFQKVVYVENVSSAALVAAFLSYYLVKVVPYWALAIVATTVTFFGPLVYHQNQEIIDEQLGDYSAKA
Bretziella_fagacearum             YLLSWNNPRDSGIAYGSIVAGIILIRCLDVLRYAFKLTYLTLAVTVAAEVLGKLVINGLTSQAGDLVVTEFQKVIYVENVSGAAFIAAFLSYYLVKVVPYWALAIVATTLTFFGPLIYHQNQEVIDEQLGDYSAKA
Huntiella_omanensis               YLLSWNNPRDSGIAYASIVAGIILIRCLDVLRYTLKLTYLTLAITIAAEVAGKLVINGLTSQASDLVTTEFQRVIYVENVSTAAFVAAFISYYLVKIVPLWALAIVATTIGFFAPLVYHQNQELIDEQLGDYSAKA
Huntiella_savannae                YLLSWNNPRDSGIAYASIVAGIILIRCLDVLRYTLKLTYLTLAITIAAEVAGKLVINGLTSQASDLVTTEFQRVIYVENVSTAAFVAAFISYYLVKIVPLWALAIVATTIGFFAPLVYHQNQELIDEQLGDYSAKA
Huntiella_moniliformis            YLLSWNNPRDSGIAYASIVAGIILIRCLDVLRYTLKLTYLTLAITIAAEVAGKLVINGLTSQASDLVTTEFQRVIYVENVSTAAFVAAFLSYYLVKVVPLWALAIVATTIGFFAPLVYHQNQELIDEQLGDYSAKA
Davidsoniella_virescens           YLLSWVSPRDTGIAYATIIFSIILMRCVDVLRYAFKLTYYTLTITVAAEVLGKLVINGLTSQAADFIAIESQKLVYAENVSGAALVAAFLSYHLVKIVPYWALAIVATTLTFFGPLVYHQNQELIDAQIGDYSAKA
Endoconidiophora_polonica         YLLSWASPRDSGIAYASIVFGIILVRCLDVLRYAFKLTYLTLAITVTAEVLGKLAINGLTSQTTDIIAIESQKLVYAENISGIALITAFLSYHLVKIVPYWALAIVATTVTFFGPLVYRQNQELIDAQIGDYSAKA
Endoconidiophora_laricicola       YLLSWASPRDSGIAYASIVFGIILVRCLDVLRYAFKLTYLTLAITVTAEVLGKLAINGLTSQTTDIIAIESQKLVYAENISGIALVTAFLSYHLVKIVPYWALAIVATTVTFFGPLVYRQNQELIDAQIGDYSAKA
Thielaviopsis_euricoi             YLLSWRNPRDSAIAYLSIVAGIIIINCLDVFRYTLKFTYLALAVTVSGEILGKAVLNGLTSQSNDFASIEAQKILYVENVSAGALIAAFLSYHLVKIVPYWALAIFTATAIFFGPLVYNDNKEFIDAQIGTYSAKA
Thielaviopsis_musarum             YLLSWRNPRDSAIAYLSIVASIVMINCLDVFRYTLKFTYLALAVTVSGEILGKVVLNGLTSQSNDFASIEAQKILYVENVSAGALIAAFLSYYLVKIVPYWALAIFTATAVFFGPLVYNDNKEFIDAQIGTYSAKA
Thielaviopsis_paradoxa            YLLSWRNPRDSAIAYLSIVASIIIINCLDVFRYTLKFTYLALAVTVSGEILGKMVLNGLTSQANDFASIEAQKILYVENVSAGALVAAFLSYHLVKIVPYWALAIFTATAVFFGPLVYNDNKEFIDAQIGTYSAKA
Thielaviopsis_punctulata          YLLSWRNPRDSAIAYLSIVASIIMINCLDVFHYTLKFTYLALAVTVSGEILGKMVLDGLTSRANDFASIEAQKILYVENISAGALTAAFLSYHLVKILPYWALAIFTATAVFFGPLVYNDNKEVIDAQICAYSSKA
Pseudogymnoascus_verrucosus       SLFSWENPRASALAYAATVVFIFAARYLDLLRYGLKLTWMALGVTVAAEVAGRALFAGFTAQTGDLFVIESQRLLFAENVSGAALLSAFISYHLVKIVPLWGLALIGTSVLFLGPLVYTTNKALIDGQIGDYSAKA
Pseudogymnoascus_destructans      SLFSWENPRASALAYATTVVFIFAARYLDLLRYGLKLTWMALGVTVAAEVAGRALFAGFTAQTGDLFVIESQRLLFAENVSGAALLSAFISYHLVKIVPLWGLALIGTSVLFLGPLVYTTNKALIDGQIGDYSAKA
Pseudogymnoascus_pannorum         SLFSWENPRASALAYAATVIFIFAARYLDLLRYGLKLTWMALGVTVAAEVAGRALFAGFTSQTGDLFVIESQRLLFAENVSGAALFSAFISYHLVKIVPLWGLALIGTSVLFLGPLVYTTNRELIDGQIGDYSAKA
Phialophora_americana             TLLSWKNKRATGITFVSAITFIFAVRYLPIVRYVIKLTWMSLGVVTIAEASSKALMSSVASSLDDLFVIEIQRIVFAENITAAAFLTAFITYYLIKIVPFWGMALIATSVMFLAPLIYLENKEFIDAQLGTAAAKA
Phialophora_verrucosa             TLLSWKNKRATGITFVSAIAFIFAVRYLPIIRYVIKLTWMSLGVVTIAEASSKALMSSVAGSLDDLFVIEIQRIVFAENITAAAFLTAFITYYLIKIVPFWGMALIATSVGFLAPLIYLENKEFIDAQLGTAAAKA
Cladophialophora_carrionii        TLLSWKNKRATGITFASAIAFIFAVRYLPIIRYVIKLTWMSLGVVTIAEASSKALMSSVASSLDDLFVIEIQRIVFAENITAVAFLTAFITYYLIKVVPFWGMALISTSVIFLAPLIYLENKEFIDAQLGTAAAKA
Cladophialophora_yegresii         TLLSWKNKRATGITFASAIAFIFAVRYLPIIRYVIKITWMSLGVVTIAEASSKALMSSVASSLDDLFVIEIQRIVFAENITAVAFLTAFITYYLIKIVPFWGMALISTSFIFLAPLIYLENKEFIDAQLGTAAAKA
Capronia_coronata                 TLLSWKNKRATGVTFVSLVAFIFACRYLPIFRYLLKISWMTLGVVTLAEASSKALMSSVAGALEDLFVIEGQRIVFAENITATAFLSSFVTYYLIRLLPFWGVALVGTVVAFLGPLVYLENKELIDSQLGTYAAKA
Capronia_epimyces                 TLLSWKNKRATGITFVSVLALIFAFRYLPILRYLLKISWMTLGVVTLAEASSKALVSSVAGALDDLFIIEGQRIVFAENVTAAAFLSAFVTYYLIKLLPLWGVSLVATVIAFLGPLIYLENKELIDSQLGTYAAKA
Exophiala_dermatitidis            TLLSWKNKRATGITYLGLVAFIFACRYLPILRYFLKITWMTLGVVTLAEASSKLVMSSVAGALDDLFVIEGQRIVFAENVTAAAFLSAFVTYYLIKLLPFWGVALVAANVAFLGPLIYLENRELIDAQLGTAAAKA
Rhinocladiella_mackenziei         TLLSWKNKRSTGITFASVVAFIFACRYLPITRYLLKISWMTLAVVTIAEASSQALLSSVVGALDGLFVIEGQRLVFAENITAAAFLSTFLTYYLIKLLPLWGLALLFTCVIFLGPLIYIENQELIDSQLGTYAAKA
Cladophialophora_immunda          TLLSWKNKRATGITFASVVAFIFACRYLPIIRYVIKITWMTLGVVTLAEASSKALMSSLASSLDDLFVIEIQRIVFAENVTAAAFLTAFLTYYLIKIVPFWGMALIASCVVFLGPLIYIENKEFIDSQLGTYAAKA
Fonsecaea_pedrosoi                TLLSWKNKRATGITFASVVAFIFACRYLPIIRYVIKITWMTLGVVTLAEGSSKALMSSVAGTLDDLFVIEIQRIVFAENVTAAAFLTAFLTYYLIKIVPFWGMALIASCALFLGPLIYIENKEFIDSQLGTYAAKA
Fonsecaea_monophora               TLLSWKNKRATGITFASVVAFIFACRYLPIIRYVIKITWMTLGVVTLAEGSSKALMSSVAGTLDDLFVIEIQRIVFAENVTAAAFLTAFLTYYLIKIVPFWGMALIASCALFLGPLIYIENKEFIDSQLGTYAAKA
Fonsecaea_multimorphosa           TLLSWKNKRATGITFASVVAFIFACRYLPIIRYVIKITWMTLGVVTLAEASSKALMSSVASSLDDLFVIEIQRIVFAENVTAAAFLSAFLTYYLIKIVPFWGMALIATCVLFLGPLIYIENKEFIDAHLGTYAAKA
```

Cladophialophora_psammophila	TLLSWKNKRATGITFASVVAFIFACRYLPIIRYVIKVTWMTLGVVTLAEASSKALMSSVASSLDDLFVIEMQRIIFAENVTAAAFLTAFMTYYLIKIVPFWGMALIANCVVFLGPLIYIENKEFIDSQLGTYAAKA
Cladophialophora_bantiana	TLLSWKNKRATGITFASVVALIFACRYLPIIRYVIKITWMTLGVVTLAEASSKALMSSVASSLDDLFVIEMQRIIFAENVTAAAFLTAFITYYLIKIVPFWGMALIANCVVFLGPLIYIENKEFIDSQLGTYAAKA
Fonsecaea_erecta_Corr	TLLSWKNKRATGITFACVIAFIFACRYLPIIRYVIKITWMTLGVVTLAEASSKALMSSVASSLDDLFVIEIQRLVFAENVTAAAFLTAFLTYYLIKIVPFWGMALITSCVVFLGPLTYIQNKEFIDSQLGTYATKA
Exophiala_spinifera	TLLSWKNKRATGVTFLSTVLFIFACRYLPILRYFLKITWMTLGVVTLAEASGKALMSSVAGALDDLFVIEGQRIVFAENITATAFFSAFVTYYLIKLLPFWGLSLFFTCVIFLGPLIYIENKEAIDAQLGTATAKA
Exophiala_oligosperma	TLLSWKNKRATGLTFLSAVAFIFACRYLPILRYVLKITWMTLGVVTLAEASSKALMSGVAGALDDLFVIEGQRIVFAENITATAFFSAFLTYYLIKLLPFWGLSLFFTCVVFLGPLIYVENKEVIDAQLGTATAKA
Exophiala_calicioides	TLLSWKNKRATGITFLSLVAFIFACRYLPILRYVLKVSWMTLGVVTLAEASSKALMSGLASTLDDLFVIEVQRIIFAENVTAAAFLTTFTTYYLIKLLPFWGLSLLGTCVAFLGPLVYLENQELIDAQLGTYAAKA
Exophiala_alcalophila	SLLSWKNKRATGITFAAVVAFIFACRYLPITRYLIKCTWMTLGVVTLAEAAGKLLMSSVASSLDDLFVIEFQRIVFAENITAAAFLTTFISYYLIKLLPFWGLALLGTCVVFLGPLVYIENKELIDSHLGTYAAQA
Exophiala_sideris	TLLSWKNKRATGVTFASVIVFIFACRYLPILRYLLKITWMTLGVVTLAEASSTALMSSVAGTLDDLFVIEGQRIVFAENITAAAFVSAFLTYYLIKLLPFWGMSLLSTCVIFLGPLIYLENKQLIDSQLGTATAKA
Exophiala_mesophila	TLLSWKNKRATGITFLSVVALIFACRYLPVLRYSLKLTWSVLGVVTLAEVASKTVTSSLTSALDDLFVIEAQRIVFAENTTGATFFAALVSYYLIKLLPLWGFILLSTVVLFTAPLIYIENKEFVDAQLGAYTAKA
Exophiala_aquamarina	TLLTWKNKRATGITFLAAVALIFAFRYLPVLRYSLKVTWTTLGVVTAAEVASKLVTSSVTNALDDLFVIEAQRIVFAENVTAGAFVASLVSYYLIKLLPLWGFALFSTIVTFIAPLIYIENKELIDSQLGAYTAKA
Exophiala_xenobiotica	SLLSWKNKRATGVTFLAAVTFIFACRYLPIVRYMLKITWMTLGVVTLAEASSKALMSSVAGTLDDLFVIEAQRIVFAENVTAVAFLSAFLTYYLIKLLPFWGMSLLFTCVIFLGPLIYIENKEVIDAQLGTATAKL
Phialophora_attae	TLLSWKNPRATGATFASVLVFIFACRYLPIIRYALKLTWMTLGVVTLAETATKLAAGSLTSSLDDVFVIESQRVVFAENITGAIFLATFLSYYLVKLLPFWGMALLGASVIFLGPLIYVMNQELIDSNIGEYAGAA
Cyphellophora_europaea	TLLSWKNPRATGATFAATLVFIFAARYLPVTRYALKLTWMTLGVVTLAEALSKLATGSLTSSLDDVFVIESQRVVFAENVTAAVFLATFLSYYLVKLLPMWGMALLATSVVFLGPLIYVMNKEVIDSNIGEYAGAA
Knufia_petricola	NLLSWKNPRATGVTFASFIVLIFACRYLPIVRLTLKGTWTILGVVTLAEAAAKFTISSVAGQLDDVFVIEAQRIVFAENITALAFTAAFLSYYLVKLLPAWGFALFATCVTFLVPLVYTQNKELIDSQYGEYANVA
Herpotrichiellaceae_sp_UM238	NLLSWKNPRATSISFATIVIFIFACRYLPIVRLTLKATWTILGVVTLAEAAAKFTISSVANSLDDVFVIEIQRIVFAENITAVAFTASFLSYYLVKLLPAWGFALFSTCVVFLVPLIYTQNKELIDAQLGEYANVA
Scytalidium_lignicola	SLLSWDNPRASGIAFASTVLFILAVRYLDILRYAFKLTWMTLGVTVLAEIVGQTLIQGLTSQVGDVFIIEAQRIIFAENVSGAAFLASFISYFLVKIVPYWGLALIGSSVLFLAPLIYKSNKEVIDLYLSEYSAKA
Scytalidium_sp_3C_Corr	SLLSWRDPRASGIAYASIVVFILAARYLDILRYAFKLTWMTLGVTVVAEIIGQSFLQGLSSQVGDVFVIESQRIVFAENVSGAAFLSAFISYYLVKVVPYWGLALIGTTVLFFAPLIYTTNQELIDHHLGEYSAKA
Acidomyces_richmondensis	SLLSWENPRATAISYASLVLIIFACRYVPIVRYALRATWIVLGVTAAAELAGRMVLQGLTSKLEDAFVIEFQRILFAENLTIGAFFTTLLTYFLIKITPAWGLALLFSTLIYFVPLIYITNKDLIDEHLKDYSAKA
Baudoinia_panamericana	TLLSWENPRATAISYLAIVLFIFACRYVPIMRYALRLTWITLGITAAAEVVGRVAMSGVVSKLEDVFVIEFQRVLFAENVTIAVFFTTLLTYFLIQITPAWGLALLFTTLTYFTPLVYITNKDFIDGHVKDASSKA
Pallidocercospora_crystallina	NLLSWENPRATAITYVFIVLSILTARYVPVSRYALKALYIVLGVTAALEIAGQTLLQGVSTKMQDGFVIEFQRIIFAENVTIAAFASTFLTYWLIKIMPTWGLSLFFTTVIFFAPLVYITNKELIDEHLKEYTGKA
Nothophaeocryptopus_gaeumannii	NLLSWENPRATAITYVFIVLSILTARYVPVSRYALKALYIILGVTAAAEVAGQTLLQGVSTKMQDGFVIEFQRIIFAENVTIAAFVSTFLTYWLIKIMPTWGLFLFFTTVIFFAPLVYITNKELIDEHVKEYTGKA
Mycosphaerella_musivoides	NLLSWENPRATAVSYATIVLSILVFRYAPVAKYLLKALYIVLGITAAAEMAGKTLLQGVATKIVDVFVIEFQRVVFAENVTIAAFASAFLSYWLIKVMPSWGLFLFFTTVIYFAPLVYITNQELIDEQLREYSAKA
Sphaerulina_musiva	NLLSWENPRATAVSYATIVLSILVFRYAPVAKYLFKALYIVLGITAAAEMAGKTLLQGVATKIVDVFVIEFQRVVFAENVTIAAFASAFLSYWLIKVMPSWGLFLFFTTVIYFAPLVYITNQELIDEQLREYSAKA
Sphaerulina_populicola	NLLSWENPRATAVSYATIVLSILVFRYAPVAKYLLKALYIVLGITAAAEIAGKTLLQGVATKIVDVFVIEFQRVAFAENVTIAAFASAFLSYWLIKVMPSWGLFLFFTTVIYFAPLVYITNQELIDEQLREYSAKA
Mycosphaerella_sp_Ston1	NLLSWENPRATAISYATIVLSILVVRYVPVAKYLLKALYIVLGLTAAAEIAGKTLLQGVATKIVDVFVIEFQRVVFAENVTVAAFASSFLSYWLIKVMPSWGLFLFFTTVIYFAPLVYINNQELIDEQLKEYSAKA
Cercospora_sojina	NLLSWENPRATAISYATIVLSILAFRYVPVAKYAFKALYIVLGITAAAEITGKLVLQGVATKIVDAFVIEFQRIVFAENVTVAAFSSSFLSYYLIKVMPTWGLLLFFTTLVYFAPLIYVTNKELIDEQLKEYTAKA
Cercospora_nicotianae	NLLSWENPRATAISYATIVLSILAFRYVPVAKYAFKALYIVLGITAAAEITGKLVLQGVATKIVDAFVIEFQRIVFAENVTVAAFSSSFLSYYLIKVMPTWGLFLFFTTLVYFAPLVYVTNKELIDEQLKEYTAKA
Cercospora_beticola	NLLSWENPRATAISYATIVLSILAFRYVPVAKYAFKALYIVLGITAAAEITGKLVLQGVATKIVDAFVIEFQRIVFAENVTVAAFSSSFLSYYLIKVMPTWGLLLFFTTLVYFAPLVYVTNKELIDEQLKEYTAKA
Cercospora_berteroae	NLLSWENPRATAISYATIVLSILAFRYVPVAKYAFKALYIVLGITAAAEITGKLVLQGVATKIVDAFVIEFQRVVFAENVTVAAFSSSFLSYYLIKVMPTWGLFLFFTTLVYFAPLVYITNKELIDEQLKEYTAKA
Cercospora_canescens	NLLSWENPRATAISYATIVLSILAFRYVPVAKYAFKALYIVLGITAAAEITGKLVLQGVATKIVDAFVIEFQRIVFAENVTVAAFSSSFLSYYLIKVMPTWGLFLFFTTLIYFAPLIYVTNKELIDEQLKEYTAKA
Cercospora_zeina	NLLSWEHPRATAISYATIVLSILAFRYVPVAKYAFKALYIVLGITAAAEITGKLLLQGVATKIVDGFVIEFQRIVFAENVTVAAFSSSFLSYYLIKVVPTWGLLLFFTTVLYFAPLIYVTNKELIDEQLKEYTAKA
Dothistroma_septosporum	SLLSWDNPRATAISYAVIVAFVFTTRYVPITRYALRVLYITLGVTAALEIAGKVVLEGLASKLSDVFVIEFQRIVFAENVTVAAFLTAFLGYFLVKIMPAWGLVLLSTTVIYFAPLAYLSNKEFIDHHIKDYTGKA
Dothistroma_pini	NLLSWENPRATAISYAVIVAFVFTTRYVPVTRYALRLLYITLGVTAALEIAGKVVLEGLASKLSDVFVIEFQRIVFAENVTVAAFLTAFLGYFLVKIMPAWGLVLLSATVIYFAPLAYISNKEFIDHHIKDYTGKA
Passalora_fulva	NLLSWENPRATAISYAMIVAFVFTTRYVPVTRYALRVLYITLGVTAALEIAGKVILEGLASKLSDVFVIEFQRIVFAENVTVAAFVTAFLGYYLVKIMPAWGLVLLSTTVIYFAPLVYISNKELIDYHIKDYTGKA
Mycosphaerella_arachidis	SLLSWENPRATAISYALIVAFVFTTRYVPVTRYALRVLYLTLGVTVALEIAGKVVLEGLASKLSDIFVIEFQRIVFAENVTVAAFLSAFLGYFLVKIMPAWGLLLLGTTVIYFAPLTYITNKEVIDHHIKNYTGKA
Exutisphaerella_laricina	NLLSWENPRATAISYGSIVALVFTTRYVPVTAYLLRALYITLGITAALEVAGKVVLEGLASKLGDVFVIEFQRVVFAENVTIGAFLTAFVGYFLVKIMPAWGLVLLGTTVVYFAPLTYISNKEFIDHHLKDYTGKA
Microcyclosporella_mali	SLLSWENPRATAISYGTIATLIILGRYMPLSRYALKAVYSVLGLTAALEIVGKLAMDGIMAKLGDVFVIEFQRVLFAENVTAAAFVTAFLSYFLVKVMPNWGLALFFTTVVYFTPLVYISNKELIDQTIKDYSAKA
Mycosphaerelloides_madeirae	SLLSWENPRATAISYATIVLSILIGRYMPLSRYALKAAYTVLGITAAFEIVGKMALDGLTAKLGDVFVIEFQRILFAENVTVAAFVTSFLSYFLVKLMPTWGLALFFTTIVYFTPLVYISNKELIDQQLKDYSAKA
Lecanosticta_acicola	SLLSWENPRATGVSYATIVALLFIARYVPVAKFGLKVLYTVLGVTAAAEIAGKLALEGISSKTGDIFVVEFQRIVFAENITVAAFFTAFVSYFLIKITPSWGLALLFTTVLYFAPLAYITNKEFIDEHLKDYAAQA
Zasmidium_angulare	NLLSWENPRATAISYVTIVALIFATRYLPVSRFVLKGLYVLLGCTAAAEAVGKLVLEGIASKLGDIFVIEFQRIVFAENITILAFVTSFLSYFLIKIMPSWGLALFFTTAAYFIPLGYISNKEFIDEHLKEYTAKA
Zasmidium_citri_griseum	NLLSWENPRATAISYVTIVALILATRYLPVTRLALKGLWVLLGCTAAAETVGKLVLNGIASKLGDLFVIEFQRIVFAENVTILAFFTTLLSYVLIKIMPSWGLALFFTTLIYFVPLAYISNKEIIDEHLKEYTAKA
Ramularia_collo_cygni	NLLSWENPRATAISYASIVILVFATRYLPITRYTMKALYMLLGLTAAAEIIGKVALQGVASKLADTFVIEFQRIVFAENVTGAAFISAFVTYFLVKIMPKWGLFLFFTTVVYFAPLAYISNKEFIDEHLKDYSSQA
Ramularia_endophylla	NLLSWENPRATAISYASIVVLVFAARYVPVTRYTMKALYMLLGVTAGAEIVGKVALQGFSSKLADTFVIEFQRIVFAENVTVAAFVSALVTYFLVKIMPKWGLFLFFTTAIYFAPLAYISNKEFIDEHLSGYSSQA
Zymoseptoria_brevis	NLLSWENPRATAISYASIVFFVFAARYVPLTRYAMKALYTVLGITAAAELAGRAALEGLSSKLADAFVIEFQRVIFAENITIGAFLSAFLTYFLVKIMPKWGLFLFFTTVVYFAPLAYISNKEFIDEHLSQYSAQA
Zymoseptoria_tritici	NLLSWENPRATAISYASIVFFVFAARYIPLTRYAMKALYTVLGITAAAELAGRAALEGLSSKLADAFVIEFQRVIFAENITIGAFLSAFLTYFLVKVMPKWGLFLFFTTVVYFAPLAYISNKEFIDEHLSQYSAQA
Zymoseptoria_ardabiliae	NLLSWENPRATAISYASIVFFVFAARYVPLTRYAMKALYTVLGITAAAELAGRAALEGLSSKLADAFVIEFQRVIFAENITIGAFLSAFLTYFLVKIMPKWGLFLFFTTLVYFAPLAYISNKEFIDEHLSQYSTQA
Zymoseptoria_passerinii	NLLSWENPRATAISYATIVFAVFAARYIPLTRYAMKALYTVLGITAAAEIAGRVALEGFSSKLADAFVIELQRVIFAENITIAAFLSAFLTYFLVKVMPKWGLFLFFTTVVYFAPLAYISNKEFIDEHLSQYSAQA
Pseudocercospora_fijiensis	NLLSWENPRATAVTYATFVLSILIARYVPVVRYLLKASYIVLGITAALEATGRLALQGVASKLSDLFVIEFQRIVFAENITILAFLSTFTTYWLVKIMPAWGFILLSTTVLFFTPLVYITNKELIDEYLKNVSAQA
Pseudocercospora_pini_densiflorae	NLLSWENPRATAITYATFVLSILIARYVPVVRYLLKASYIVLGITAALEGVGRLALQGVASKLSDLFVIEFQRIVFAENITILAFLSAFTTYWLVKIMPAWGFILLSTTVLFFTPLVYITNKELIDEHLKNVSAQA
Mycosphaerella_eumusae	NLLSWENPRATAITYTTFVLSILIARYVPVIRYLLKASYIVLGITAALEGVGRLALQGVASKLSDLFVIEFQRIVFAENITILAFLSAFTTYWLVKIMPAWGFILLSTTVLFFTPLVYITNKELIDEYLKNVSAQA
Ramichloridium_luteum	NLLSWENPRATSVSYAAIVSLIFTLRYVPVFRYVLRVLYLLLGLTATAELIGKFAVDGFASKLADVFVIEFQRIVFAENVTVGAFSTAFVTYWLVRFMPTWGIALFFTTVIFFTPLTYITHRELIDQHIQEYSAKA
Leptoxyphium_fumago	SLLSWENPRVTAISFASIITAIFIGRYVPIARYTLKATWTILGGIAAVEVAAKAVTAGVVSKLEDVFVIEVQRIVFAENITIAAFAASLVTYFLIKVTPTWGLALLSTFILYLAPLIYIKNRELIDGHLGSYATKA
Preussia_sp_BSL10	SLLSWENPRATAISFASIVSAILIVRFIPVERFVLKAIFTVLGVTSAAELVGRFILEGIATKTGDVFVIEFQRILFAENVTIAATFTALIGYFLVKVTPKWGLLLISTCVLYLAPLVYITNKELIDTHLSQYVSLA
Cladosporium_sp_SL_16	SLLSWENPRATAISFASIVSTILIVRFIPVERFVLKSIFTVLGVTSAAELVGRFVLEGIATKTGDIFVIEFQRILFAENVTIAATFAAIIGYFLVKVTPKWGLLLISTSVIYLAPLIYITNKELIDTHLSQYVSLA
Cladosporium_cladosporioides_Corr	SLLSWENPRATAISFATIVTSILLFRFVPIERYVLKAIFTVLGVTAAAELVGHFVLEGLSTKTGDLFVIEFQRILFAENVTIAAFFASLISYFLVKVTPKWGLLLIATSVVYLAPLIYITNKELIDTHLSQYVSLA
Cladosporium_sphaerospermum	SLLSWENPRATAISFATIVTGILLARYVPLERYVLKAAFTVLGVTAAAELVGHFILEGFATKTGDLFVIEFQRILFAENVTIAAFFTTLIGYFLVKVTPKWGLLLLSTCVVYLTPLIYIKNKELIDRHLSQYVSMA
Cladosporium_phlei	SLLSWENPRATAISFATIVTGILLTRYVPIERYVLKAAFTVLGITAAAELAGQFIFQGFATTTGDVFVIEFQRILFAENVTILAFFTTLIGYFLVKVTPKWGLLLLSTFAVYLTPLVYIKNKDFIDQHLSQYVSVA
Rachicladosporium_antarcticum	SLLSWENPRATAISFVAIVTTIIVARYVPVDRYVLKALFTVLGITAAAEIAGQMAFQGFATKLGDVFVIEFQRIIFAENVTLAAFLAALISYFLVKVTPKWGLLLLSTSVVYLAPLIYIKNKDFIDSHLSQYVSKA
Rachicladosporium_sp_CCFEE_5018	SLLSWENPRATAISFVAIVTTIIVARYVPVDRYVLKALFTVLGITAAAEIAGQMAFQGFATKLGDVFVIEFQRIIFAENVTIAAFLAALISYFLVKVTPKWGLLLLSTSVVYLAPLIYIKNKDFIDSHLSQYVSKA
Microcyclospora_tardicrescens	NLLSWENPRATAISYVSVVVAIIIARYVPIVSLALRGLWIVLGIVAAVETTAQLALQSMTQHLEDVFVIEVQRVLFAENVTIAAFFVTLLSYFLIKITPAWGLALIFTTAVYFLPLIYLQNKELIDSQLKDASVKA
Microcyclospora_pomicola	NLLSWQNPRATAISYVSVVIAIIIARYVPLVSLALRGLWIVLGIIAAVETTAQLALQSVTQHLEDVFVIEVQRVLFAENVTVAAFFVTLLSYFLIKITPAWGLALIFTTAVYFLPLVYIQNKELIDSQLKDASAKA
Sphaceloma_murrayae	NILSWENPRVSAIAYATIVTTIFFTRYVPILKYFFKASYVILGIIAAAEIAGKLVFKGLATQLEDVFVIEFQRIVFAENVTVSAFSAALISYFLVKFVPKWGIALIATSVVFFVPLIYIYQNRELIDGQLSQYSAKA
Elsinoe_sp_CQ_2017a	NILSWENPRVSAIAYATIVTTIFFTRYVPILKYFFKASYVILGIIAAAEIAGKLVFKGLATQLEDVFVIEFQRIVFAENVTVSAFSAALISYFLVKFVPKWGIALIATSVVFFVPLIYIQNRELIDGQLSQYSAKA
Elsinoe_australis	NILSWENPRVSGIAYASIVTFIFFTRYVPVLKYFFKASYMILGIVAAAEIAGKLIFRGLASQLEDVFVIEFQRIVFAENVTVAAFTSALIAYFLVKFVPKWGIALIATSVVFFVPLVYIQNRELIDGHLSQYSAKA
Cyanodermella_asteris	SLLAWEHPRVTAISYATIITSIFILRYVPVLRLTFKVLAYSLGSAALLEGASQALLQSILAALEDVFVIESQRLLFAEDATFVAFLASFLAYWLIKFTPVWGLSLIAVTTIYFVPLIYIYITNKEVIDEQLGDLSAQA
Tuber_melanosporum	DLFTWKFPRATGFFFFAAISLIFAFRFVNVLRYVFKATYILFFLVTFLEIASKPFGRGFISSFGELFALEFQRVLFAENITVVASFVSFIGYFLIKYIPLWALSLLTTITAFTAPLIYLQNQEVIDEQISELTQKV

Tuber_magnatum	DLFTWKFPRATAFFFSSAIFLIFAFRFVNVLRYVFKATYLLFFGVAFLEIAAKPFGRGFISSFGELFVLEFQRVLFVENITVVAFFASFIGYFLIKYIPFWALLLLTTITAFTAPLIYLQNQEVIDEQITELTQKV
Tuber_umbilicatum	DLFTWKFPRATGFFFFSAISLIFAFRFVNVLRYVFKATYLLFFGVAFLEIAAKPFGRGFISSFGELFVLEFQRVLFAENITVVAFFASFIGYFLIKYIPFWALLLLTTITAFTAPLVYLQNQEVVDEQITELTQKV
Tuber_calosporum	DLFTWKFPRATGFFFFSAISLIFAFRFVNVLRYVFKATYLLFFGVAFLEIAAKPFGRGVISSFGELFVLEFQRVLFAENITVVAFFASFIGYFLIKYIPFWALLLLTTLTAFTAPLIYLQNQEVIDEQIVELTQKV
Choiromyces_venosus	DLFTWKFPRATGFFFFSAISLISAFRFVNVLRYVFKATYLLLFGVAFLEIAAKPFGRGIISSFGELFVLEFQRVLFAENITVVAFFASFIGYFLIKYIPFWALLLLTTVTAFTAPLIYLQNQEVIDEQITELTQKV
Tuber_aestivum	DLFTWKFPRATGFFFSFAISLILAFRFVNVLRYVFKATYLLFFGVAFLEIAARPFGRGFISSFGELFVLEFQRVLFVENITVVAFFASFIGYFLIKYIPFWALLLLTTITAFTAPLIYLQNQEVIDEQITELTQKV
Tuber_borchii	DLFTWKFPRATGFFFFSAISLIFAFRFVNVLRYVFKATYLLFFGVAFLEIAAKPFGRGFISSFGELFALEFQRVLFAENITVVAFFASFIGYFLIKYIPFWALLLLTTVTAFTAPLTYLQNQEAIDEQLVELTQKV
Tuber_microsphaerosporum	DLFTWKFPRATGFFFFSAISLIFAFRFVNVLRYVFKATYLLFFGVAFLEIAAKPFGRGFISSFGELFAVEFQRVLFAENITIVAFFASFIGYFLIKYIPFWALLLLTTVTAFTAPLTYLQNQEVIDEQIVEFTQKV
Morchella_septimelata	DLFTWKYPRATGFFFFSVISMITAFRFINVLRWVFKGAYLLFASAALLEVAGKPFGKGLVSSFNEAFVVEFQRVIFVENITIVAFVTTFAAYFLIKYMPFWSLLLTATITAFTAPLIYLQNQELIDEKIVDLSQKA
Morchella_eximia	DLFTWKYPRATGFFFFSVISMITAFRFINVLRWVFKGAYLLFASAALLEVAGKPFGKGLVSSFNEAFVVEFQRVIFVENITIVAFVTTFAAYFLIKYMPFWSLLLTATITAFTAPLIYLQNQEFIDEKIVDLSQKA
Morchella_importuna	DLFTWKYPRATGFFFFSVISMITAFRFINVLRWVFKGAYLLFASAALLEVAGKPFGKGLVSSFNEAFVVEFQRVIFVENITIVAFVTTFAAYFLIKYMPFWSLLLTATITAFTAPLIYLQNQEFIDEKIVDLSQKA
Morchella_conica	DLFTWKYPRATGFFFFSVISMITAFRFINVLRWVFKGAYLLFASAALLEVAGKPFGKGLVSSFNEAFVVEFQRVIFVENITIVAFVTTFAAYFLIKYMPFWSLLLTATITAFTAPLIYLQNQEFIDEKIVDLSQKA
Pyronema_omphalodes	DLFTWKFPTATGFFFLSAILTIVAFRYVNVLRYVFKALYMSFGAVTLLEIAGKPLGSGIVSSFQQIFLLEFQRVIYVENVSLSAFVASFFGYFLIKYIPFWALLLLATVTAFTAPLIYLNNKEVIDDQIADLQSKV
Terfezia_boudieri	DLVTWKYPRASGLIFISLLSTILAFRFVNVMRYVFKAAYLLLGSVAALEYAGKHLGKGVVSQFEEIVVVEFQRVVFVENLTVAAFVASFFGYFLIKYLPFWSLAFLGTILGFTGPYVYINNQEQIDAQISQLSEKV
Arthrobotrys_oligospora	DTFTWKYPRTTGLLFASSLAFFLLGHYVNILRYAFKAAYITFAATAAIEYLGRPTTTGFMTQFAEIIVLEFQRVIFVENLTIASFVFSLIGYFLVKFMPIWSLLILADVLLFTVPLLYLQNQELIDSHISDITGKL
Duddingtonia_flagrans	DTFTWKYPRTTGLLFASSLAFFLLGHYVNILRYAFKAAYITFAATAAVEYLGRPTTTGFMTQFAEIVVLEFQRVIFVENLTIASFVFSLIGYFLVKFMPIWSLLIFADVLLFTVPLLYLQNQELIDSHINDITGKL
Dactylellina_haptotyla	DTFTWKYPRTTGLLFASSLAFLLLGHYVNILRYALKGAYITFAATAAFEYLGRPTTTGFMTQFAEIVVLEFQRIIFVENLTVASFVLSLIGYFLVKFMPIWSLLILADILIFTAPLLYLQNQDLIDHHINDLTGKL
Protomyces_lactucaedebilis	DMLTWKYPRASGLILATLLTTLIVSKSVNLPKLAFRALWVVLASTTSLELAGQFVFEGLVAKFDELFLTEAQRLLFVKNATTVAFVLSFVAYNLIKFLPLSALIGIAILLAFSMPPVYLRHRQVIDAQLIDLGNKV
Protomyces_inouyei	DMLTWKYPRASGLILATLLTTLIVSKSVNLPKLAFRALWVVLASTTSLELAGQFVFEGLVAKFDELFLTEAQRLLFVKNVTTVAFVLSFVAYNLIKFLPLSALIGIAILLAFSMPPVYLRHRQVIDAQLIDLGNKV
Protomyces_pachydermus	DMLTWKYPRASGLILATLLTTLIVSKSVNLPKLAFRALWVVLASTTSLELAGQFVFEGLVAKFDELFLTEAQRLLFVKNVTTVAFVLSFVAYNLIKFLPLSALVGIAILLAFSMPPVYLRHRQVIDAQLIDLGNQV
Protomyces_gravidus	ELLTWKHPRASGLILFTLLGSLIFSQSVNVPKFAFRILWVVLGSATTLEVATQFAFEGFVTKVDELFLAEAQRLLFVRDPTSVAFVLSFVAYNLIKFLSLTALVGVGIVLAFALPPIYLRHRQVIDAQLIDLGNKV
Protomyces_inundatus	DMLTWEHPRASGLILTGLLGALIFSQTIYLPRFIFRILWVTLAATTSMEVAGQFVFEGFVAKFDELFLSEAQRLLFVKNVTAVAFALSFVAYNLIKFLPLSALIGLGIILAFTAPPIYLRHKQVIDEQWIGLGNKV
Protomyces_macrosporus	DMLTWEHPRASGLILAGLLGALIFSQTIYLPRFIFRILWITLAATTSMEVAGRFVFEGFVAKFDELFLTEAQRLLFVKDVTAVAFALSFVAYNLIKFLPLSALIGLGIILAFTAPPIYLRHKQVIDEQWIGLGNKV
Taphrina_flavorubra	ELLTWKHPRASGLILSTLLSTIIFTQFVNVPKFAFRVLWITLASSTALEVAGQFVFEGLVAQFSEVAVVEIQRLLFVRNVTFVAFVLSFVAYNLIKFLPLWALTMIGLILAFTLPPVYIQNQEVIDAQIIDLGNKA
Taphrina_wiesneri	ELLTWKHPRASGLILITLLSTIIFTQFVNVPKFAFRILWITLASSTVLEVAGQFVFEGLVAQFNEVALVEAQRLLFVRNVTSVAFVLSFVAYNLIKFLPLWALTMIGLVLAFTVPPVYIQNQAAIDAQIIDLGNKA
Taphrina_deformans	ELLTWKHPRASGLILTTLLSTIIFTQFVNVPRFAFRILWITLASSTALEVAGQFVFEGLVAQFSEVAVVEAQRLLFARNVTSVAFVLSFVAYNLIKFLPLWALMLIGLVLAFTLPPVYIQNQAAIDAQIIDLGNKA
Taphrina_populina	ELLTWKHPRASGVILTTLLSSIIFTQFVNVPKFVFRMVWIGLASTTALEVAGRFIFEGLVAQFNEIAVTEAQRLFFVRNVTSAAFVLSFVAYNLIKFLPLWALAMIGIVLAFVLPPVYIQNQDVIDAQLIDLGNKA
Lipomyces_starkeyi	DLLTWKNLYVSGGSFAGALIALYLFKYVNVVKLFFNFAYIALGTAIAVEFAGRTIKPGFVSSFNEFLLVEFQQIVFVENFTVFAFVVSYFTYFLVHYVSLWSLAVVGVTVAFAAPPVYLKFQEEIDAQVDQALDKV
Lipomyces_mesembrius	DLLTWKNLYVSGGSFAGALIALYLFKYVNVVKLFFNFAYVALGTAIAVEFAGRTIKPGFVSSFNEFLLVEFQQIVFVENFTVFAFVVSYFTYFLVHYVSLWSLAVVGVTVAFAAPPVYLKFQEEIDAQVDQALDKV
Lipomyces_arxii	ELLTWKNFYVSGGSFAGALIALYLFKYVNVVKLFFNFAYVALGTAIAVEFAGRTIKPGFVSSFNEFLLVEFQQIVFVENFTVFAFVVSYFTYFLVHYVSLWSLAVVGVTVAFAAPPVYLKFQEEIDAQVDQALDKV
Lipomyces_kononenkoae	DLLTWKNLYFSGGAFAGTLVALYLFKYVNVVKLLFNFAYIALGTAIAVEFAGRTIKPGFVSSFNEFLLIEFQQIVFVENFTVFAFVVSYFTYILVHYVSLWSLTVFGVIVSFSAPPIYIKFQKEIDAQVNEALDKV
Lipomyces_lipofer	DLLTWKNLYVSGGSFVGAIVLLYLVKYVNVVQFFFNAAYITLGSAIALEFAGRSIKPGFVSSFSEFVLIEIQQIVFVESVTVLAFVVSYFTYILVHYVSLWALAFLGTILAFTAPPVYLRFQTQIDEQVDQGLDKV
Lipomyces_doorenjongii	NLFTWKSPYVSGAVFAASIVVLYLVKYVNVVKLVFNAAYVALGTAIIVEFAGRTIKSGFVSSFAETGLVELQQIVFVEHVTIIAFVVSYFVYILVNYVSLYALAFTAIIAAFTGPIVYRTFQTEIDQQIDLGLDKI
Lipomyces_japonicus	NLLTWKNPIYTGSVFAGSIVALVLVKYVDVLKLVLNAAYLAFGTGVLIELSGRSIKPGFVSSIAEGVLVEIQRIAFVENVTISAFIISYFTYFLVRYISLWTLVSAAIIFAFSLPPLYLQFQTEIDAQVNDALEKV
Lipomyces_suomiensis	DLVTWKNVLNSAIAFSGLLVSLIAVKYINVVNLLFNTAYRALGTVVVIEFVGRALGPGFVSSFDEILLVEAQKLVFVESVTLLAFVGSYFAYVMVKFVSIWTLVFFGVTVAFTAPPIYFTFQKQIDAQISKGLDKV
Lipomyces_oligophaga	DLFTWKKPICTGKVFGATLAVLFVLKYISLLKLVLNTASIAFATAVAVEFAGRYVKPGFLSSFEELVLVELQKIVYVEKTTVSAVFGSYFAYVLVHHISVWTLVLMTVLGAFAVPPIYLKYQTEIDAQIGQALSKV
Neolecta_irregularis	ELFTWKYPRASGVVFGTILTTLFVTKYFNILRFVFKVLWMAFLASASLEYGGRLMTSGLMSQLSELLLLEFQKLAYATNPTLGAFLAAWVSCQLVTYLSVLSLLIIATLTAFTTPAVYVRHQKVVDEHLQQASEKI
Pneumocystis_murina	DLFTWKYPRTSGSVLGGVTLLLLLSNTVDIPRLILRTAWIVFASSTVIEITGRSLFSGFISQIDKTALYGIQELLFARKITAIAFIVSWISFVLVQFLSFIHTLLLGVLLAFTVPLFYIKYQKTIDKYFIKLSTKI
Pneumocystis_carinii	DLFTWKYPRTSGSVLGGVTLLLLLSNTVDIPRLILRTAWIVFASSTVIEITGRSLFNGFISQIDKTALYGFQELLFARKITATAFIVSWISFVLVQFLSFVHTLLLGVLLAFTVPLFYIKYQKTIDKYFIKLGTKI
Pneumocystis_jirovecii	DLFTWKYPRNSGTILGGIVALLLFSSIVNIPHLIFRTAWIVFASSTIIEITGRSVFNGFISQTDKLILYGIQELLFVKKITAIAFAVSWISFILVQFFSFVHIILLATLLAFTIPSFYIKYQKIIDKNFIKLGTKI
Saitoella_complicata	DLCSWNYPTASAFFLALGLSGLVLIKFVNIPKLVFRGAWIVFLISAIIEYAGKIVTTGFVSTFDECILSEFQRLVFAENVTLVAFAFSFIFYQLFKIVSLWTLSLLTLIAAFSIPPFYARNKTHIDAQLTVVGEKV
Candida_hispaniensis	SVLTWEDPVDSAKVLVSILAGLYLVKSGNIIKLALQALTYAVGLGLAAEFVTGYVTQGLVSFAKSITLNKLKAIVDVENLSAQAFGVFFVLYQLTKVISAWSLILTTTVVAFTVGPIYHQFHNEIDEAINSVIEKL
Yarrowia_lipolytica	RVLTWIDPVASARSLVTILGLLYLAKYGNLFRLALRFSYWAIGLGVATEFITGKATHGLVSYAQALAFDHVKGYLDADNLSFHAALFTWVLYHVTKFVSVWSLALTATILAFAIPPVYLQFQEPIDQTISKVTDQL
Yarrowia_keelungensis	RVLTWIDPVASARSLVTILGLLYLAKYGNLFRLALRLSYWAIGLGVATEFVTGKATHGLVSYAQALAFDHVKGYLDADNLSFHAALFAWVLYHVTKFVSVWSLALTATVLAFAIPPVYLQFQEPIDQTISKITDQL
Yarrowia_divulgata	RVLTWIDPVASARSLVTILGLLYLAKFGNLFRLGLRLSYWAIGLGVATEFVTGKATHGLVSYAQALAFDHVKGYLDADNLSFHAALGAWVLYHVTKFVSVWSLALTATVLAFAIPPIYLQFQQPIDQTIAKITDQL
Yarrowia_deformans	RVLTWIDPVASARSLVTILGLLYLAKFGNLFRLGLRLSYWAIGLGVATEFVTGKATHGLVSYAQALAFDHVKGYLDADNLSFHAALGAWVLYHVTKFVSVWSLALTATVLAFAIPPIYLQFQQPIDQTIAKITDQL
Yarrowia_porcina	RVLTWIDPIASARSLVTILGLLYLAKFGNLFRLALRLSYWAIGLGVATEFVTGKATHGLVSYSQALAFDHVKGYLDADNLSFHAALFTWVLYHVTKFVSVWSLALTATILAFTIPPVYLQFQEPIDQTISKITDQL
Yarrowia_yakushimensis	RVLTWIDPVASARSLVTILGLLYVAKYGDLFRLALRLSYWAIGLGVATEFITGKATHGLVSYAQALAFDHVKGYLDADNLSFHAALFAWVLYHVTKVVSVWSLALTATVLAFAIPPVYLQFQEPIDQTISKVTDQL
Yarrowia_bubula	RVFTWIDPVASARSLVTILGLLYLAKYGNLFRLALRLSYWAIGLGVATEFITGKATQGLVSYAQALAFDHVKGYLDADNLSFHAALFAWVLYHVTKVVSIWSLALTATVLAFAVPPVYLQFQQPIDQTISKITDQL
Yarrowia_alimentaria	RVLTWIDPVASARSLVTLLGLLYLAKYGNLFRLLLRVSYYAIGLGVVTEFVTGKATHGLVSYSQALAFDHVKGYLDVESLSFHAALGAWLLFHVTKVVSLWSLALTATIIAFTVPPVYLQFQEPIDQTINKISEQL
Middelhovenomyces_tepae	ALVTWKEPIKTAKVFAALIAALVLFKSTCLIRLSVKLATFALGTGCAVEYATRVFYQGIISSAKNLTASEVEKIVNVQDLTSTVFAAGIFLYIFLGFFSLWAFFLTVVIAAFTVPVVYLKFQKEIDEALLSVAGSL
Candida_incommunis	SVLLWKNPAHSGTVLAEALGAVVVLRYGAIFRFLLRIALWAIFLSTSVEFVSRKFTAGLVSAAQHLGLDNLKALIDCRDVSAAAWVALFTLYVLSKFLTFTTLLLLTALAAFSLPLLAVTFEKEIDDVLFGLASKL
Deakozyma_indianensis	SVLLWHNPAHSGTVLAETLAAVVVLRYGGIFRFLLKLTLWAIFITTTVEYGSKQFQNGLVSSTQKLSVEEIKSIVDSRDVSAITWVGLFFVYVLSAFLSFTSLLFLLTITAFTVPLLTVKFEKEINEVLSGIASKV
Zygoascus_ofunaensis	SILTWQNPVKSGTVFIELLAILVVWRFGNIFRTALRLSYWAIGITGSVEYASRLLKPGFVSSTNKAAFSELKKLLDAEDLSATVFFFTLFLYFLTGVLSISTIAFVLVILGFTLPPVYLKFHKEIDQVVNTATSFV
Zygoascus_meyerae	PILTWQNPVKSGTLVAELLGALLLLRYGNLLRFGLRATYWAVAVSGSAEYASRLFSHGVVSSTNKATIHDVKRLFDAEDLSLTVFIGAFFAYLLTGVLSVSTLLIVSVVLAFTVPPVYLKFQKEIDHFAANVSSIV
Spencermartinsiella_europaea	QILTWQDPVKSGTVFGELLAVLLALRYGTLFRVSLRLIYWVVGGTALVEFATRKLQRGVVSSTSRISFADIKQVIDGEDLSLFVFLASFVGYILTGFLSVSALLLVTLVLSFTVPPVYLQFQHQIDEFVNNLAGTL
Diddensiella_caesifluorescens	EVLAWTNPIRSGTIFGGLLGLLLSLRYGGLLRLTLRLTYWFIGVSGAVELATRYFYTGLVSQTSRAGLSDVRKIFDAEDLSLVAFVSVYIAHCLTAFLSFSTLLFIVIISAFTVPPLYLQFQTEIDEAADSLAMAV
Blastobotrys_serpentis	KVLTWSDPVRSGTALGQILVTLFIVRYGNLLRFFLRITYWAIAITGVLEFLTRQIQRGVISSASRTCLNELNKIFDAEDLSLVAFFATIFMYILTGYVSVSALLFIATLFAFTVPRLYLQFQQEVDQVISTIASTL
Blastobotrys_proliferans	SVLTWSDPIRSGTILGEILATLFVIRYGNLLRLALKLTYYAIGATGLVELATRYFNQGVVSSTNRTGVAELKKLFDAEDLSFVAFIAVFAMYVLTGFVSVSTLTFIATIVAFTLPRLYLQFQPEIDEAVEGVASKV
Blastobotrys_mokoenaii	SVLTWNDPVRSGSVLGYVLAILFITRFFNLLRLALRLSYWAIGATALLEFVTRHLKSGVVSSTSRALANNAKKILDAEDLSLVAFVLVLAAYILTGCVSLSTLAFIATIGAFTIPRLYLQFEKEIDEVASGLAGSL
Blastobotrys_muscicola	SILTWNNPVKTGTVFGEILATLIVLKYGNLLRLALRLAYWSIGLTGAVELVSRQFYSGLVSQVSKTGLDDVKKLLDAEDLSLATYIIVVAMYILTGFVSVSTLLFIATVAAFTLPPLYLQFEEEINELA-------
Sugiyamaella_xylanicola	PVLTWENPVRSGTVLAEILGVLVVLRYANLLRLALRFSYIAIGATGVAEFATRHLYTGFVSSASHASVNDAKKLLDAEDLSLVSFVSVFVLYVLTGFLSISTLLIISTVAVFALPPIYLQFQTEIDDLLNNVASAV
Blastobotrys_raffinosifermentans	KVLTWQNPVQSGTLLAEILGVLLIIRYGNLLRTGLRALYWAIGLTGAAEFLTRHALRGAVSSTSRLALTEVKSLLDAECLSAIAFVAVVFLYILTAFVSVSAIFIFVTIAAFSLPKLYLTFQKEADEIIGGVADRF
Blastobotrys_adeninivorans	KVLTWENPVKSGSALVQTLGVLLIIRYGNLLRTGLRALYWAIGVTGAAEFLTRHALRGAVSSTSRLALSEVKSLLDAECLSAIAFFAVVFLYILTGFVSVSAIFIFLTIAAFTLPKLYLTFQKEADEIIGGVADRF
Blastobotrys_peoriensis	ALLKWQNPVRTGTVLAQVVAVLLVLRYGNLLRTALRVMYWATGATLLVEFVSRATLNGLVSQSSRLGLNEVKSLFDAEDLSALAFIGFVLLYILTGFVSVSTLLIVTTVAAFSLPKLYLTFQKEFDQVIGGIADRF
Blastobotrys_americana	LLLTWKKPVSTGIVFAEIITVLLVIRYGNLLRTSLRLMYWAVGATGVVEFLSRSVLHGIVSQSSRLGLNEVNSLFDAEDLSALAFVTFVFLYILTGFVSVSTLLFVTTLAAFSVPKLYLTFQKEFDEVIGSVTDRF
Groenewaldozyma_salmanticensis	LALTWKDPAHSAIVLGEVLAVLFVLQSNTLIRLGLRLLYLVIGGAGLVEFGTRYINKGILSTASKATFDQGKKLVDAEDLSFAAAAYFWLAYKFTAIWPVSYFLLLTTVAAFAVPPVYLRFQKEIDHYVESVTSSI

Sporopachydermia_lactativora	ALLTWADPVASGKVFVGGFTLLYVLKNVNLVATFYFVAFLALGLSAAIEFVSSKLNGGFVSQITKLSILSVQKLVYAENVTTKAAGVSYFLYKLTSLVPVYCLLTTAFVLAFVVPPVYLKFYPVVDESIPP-----
Sporopachydermia_quercuum	ELLTWKNPVATGXVFVGGFTLLYILKNVNLAATFFFVAFVALALSSSIEFVGSKLNGGFVTQVTKLSILSIQKLAYAEDITTTAAGISYFLHKLTSLLSISTLITTYFVLAFVVPPIYLKFYPVIDETI-------
Alloascoidea_hylecoeti	HILTWDNPIATGKTFSLIIFALYVVKYVNVLRVFLFLSYPVLFLSAAIEYLGRRIQTGFVSKFSFLVLSHGQKIFYSESCTFVAAISSWFLYQFTGLVSIWTLATIATIVAFSVPAIYIRFQKEIDQGVAPVRAKV
Peterozyma_xylosa	DLLSWKDPIQTGKIFGSIVVSLLLLKFVNLVNLVFRLSFLSLLAAAAAEYGGKLVTTGFVTRGGHIFEKSIQQLFYAADITFKAGIASYILFKITSWFSLFTLALISTLVAFTVPYVYETYQVEIDAAVGPLGNLI
Peterozyma_toletana	DLLSWKDPIQTGKIFGSIVVSLLLLKFVNLVNLVFRLSFLSLLAAAAAEYGGKLVTTGFVTRSGHIFEKSVQQLFYAADITFKAGIASYILFKITSWFSLFTLALLSTLVLFTVPYVYETYQVEIDAAVGPLGTFI
Nakazawaea_holstii	QLLTWKDPVETGKIFGSIILTLISLKFFNIINLIFRLSFYTLILSAIAEYSGKLILTGFITRGGHVAELQIQDLIYSKDITFKTALGAYFLYKLTSWFSIFTLLLTSTVLAFTVPLIYESNKEQIDAAIGPVGSFI
Nakazawaea_peltata	GLLTWKNPAETGKVFGGIVFSLISLKYFNIINLILRLSFYALILSAVAEYSGKLIFTGFVTKSGHIAEQEIQHLIYAKDITFKAALIAYFLYKLTSWISVFTLVLTSTIAVFTLPIVYENNKEQIDKAIGPVGAFI
Nakazawaea_wickerhamii	DLLTWKNPIETGKIFVSIIFTLISLKYFNIINLILRLTFYILILSSISEYIGKLIITGFITKATKLAERQFQDLIYSKDITFKFAIVTYFLYKITSWISIFNLLLISTILSFSLPVIYESNKEQIDEFIGPVGSFI
Pachysolen_tannophilus	SLLTWKNPVETGAIFGSIVVSLLVLKYVNVINLLFRALFVILLGSSIAEYSGKLITTGFVTRSAHFLETTIQELIYAKNITLKAGALSYILFKLTLWFSIFTLLLTSTIVAFTAPFIYENNKKQIDELLGPVGNFI
Citeromyces_hawaiiensis	QLLTWKDPIKSGQVFGGIIFALISIKTINLVNVLFRLGAVTLFVAAAAEYAGKLTTVGFVTRASHVLQLESQKILYAKDITLKSAGICYLMYKLTALISLWSLTLLSVITAFTVPALYEKYQKEIDSAVGPVGAFI
Citeromyces_matritensis	QLLTWKDPIKSGQVFGAIIFGLISLKTINLANVIFRLGAVTLFVAAAAEYAGKLTTVGFVTRANHVLQLESQKILYAKDITLKSAGICYLMYKLTALISLWSLTFLSVITAFTVPALYEKYQKEIDSAVGPVGAFF
Citeromyces_siamensis	NLLTWKDPIKTGQVFGSILLALVAVKTINLVNVVARLTFLTLSISAAAEYSGKLITVGFVTRASHVIQVQTQQLLYSKDVTLKGAGAAYVVYKLTSLLSLWTLSFVSVITAFTVPALYERYQKEIDQAVGPVGAFI
Kuraishia_molischiana	NLLTWSDPAKSGKVFGSIVTALLAIKTINILNIVFRLGFFVLIASALAEYAGKLALEGLVTRAEHIAEQTVQEIVYSKDITLKAAGALYILYLLTSWFSLWTLLFTSVVLAFSVPGVYLKYQTEIDAKVGPVGAFI
Kuraishia_capsulata	SLLTWSDPAKSAKVFGGIVLTLLTLKYINILNIVFRLGFFVLITSALAEYAGKLALEGLVTRAEHTFEQSAQETVYSKDITLKTAGGLYILYLLTSWFSLWTLLFTSVVLAFSVPGVYLKYQTEIDAKVGPVGAFI
Kuraishia_ogatae	KLLTWSDPVASGKVFGVTIASLLVLKYINLLNFFFRLGFFALIASAAAEYSGKLATTGFVTRASHFIELKGQEVVYAKDITLKVAGFSYIFYKLTSWFSLWTLLFTSVVLAFTVPVVYVKFQKEIDEAVGPVGKFI
Ogataea_trehaloabstinens	SLLTWKDPAKSAKLFSGIVGSLLLLKYINVLTILFYVGAFSLAAAAFAEYAGKLTTQGFVTRSSHVAEIKVQEILYAKDVTLKAAGISYILYKLTSWFSLWTLTFVSTVVSFIAPPIYLQNQKQIDDAVGPVGEFI
Ogataea_populi_albae	DLLTWKVPVRSARVFGTIVGSLLVFKYVNVLRIVFNFGAFALIATALAEYAGKFVTQGFVTRAPHIGEIKVQELLYAKDITLKAAGVSYILYKLTSWFSLWTLAFVSTIVSFTVPPVYLQNQKQIDDAVGPVGDFI
Ogataea_pini	ALLTWETPELSAKVFGAIVGTLLLVKFVNILSLFFYLGVLVLGVSAAAEYAGQLITQGFVTRASHALELQAQEIVYAKDITAKAAGVSYILYKVTSWFSLWTLLFVSTLAAFAVPPVYLKNQKQIDEAFGPVGSFI
Ogataea_glucozyma	SLLTWQTPEVSAKVFGAIVGTLLLVKLVNVLALGFYLGMLILGVSAVAEYAGKLTTQGFVTRASHAIELRAQEIVYAKDITAKAAGVSYIAYKLTSWFSLWTLLFVSTVAAFTVPPVYLKNQKQIDEAIGPVGSFI
Ogataea_henricii	ALLTWDNPEVSAKVFGAIVGTLLLVKLVNVLAVFFYLGVLVLGVSAAGEYVGKLTTQGFVTRASHAAELRAQEIVYAKDITAKAAGVSYILYKLTSWFSLWTLLFVSTVAAFTVPPVYLKNQKQIDEAVGPVGSFI
Ogataea_zsoltii	ELLTWKTPAVSAKVFGAIVGSLLLVKTVNVLAFVFYTGMLVLGVSALGEYAGKLATQGFVTRASHVAEVKAQEIVYAKDITAKAAGISYILYKLTSWFSLWTLLFVSTVVAFTVPPVYLKNQTQIDEAIGPVGSFI
Ogataea_nonfermentans	DLLTWKSPAASGKVFGTIIGSLLLFKFVNVLNIVFYISAFVLAVSALGEYTGRLATQGFVTRASHAAELKIQEVVYSKDITLKAAGIAYVMYKLTTWFSLWTLLFISTLASFAVPPVYLKNQTQIDDAVGPVGGFI
Ogataea_minuta	DLLTWKNPGASGRVFGSIIGSLLLIKFVNVLNIVFYVSAFVLAVSAMGEYTGKLATQGFITRAGHIAELKIQEVLYSKDITLKAAGTAYVLYKLTAWFSLWTLLFISTLVSFAVPPVYLKNQKQIDDAIGPVGSFI
Ogataea_polymorpha	DLLTWKDPSVSGKVFGSLVGSLLLLKSVNLLSIVFYISAFVLAVSALGEYAGKLATQGFVTRASHAAELKLQEIVYSKNITLKVAGVAYVLYKLTSWFTLWTLLFTATLTAFSVPPLYLKNQKQIDDAIGPVGSFI
Ogataea_kodamae	ALLTWKNPNVSGKVFGGIIGSLLLFKFANLISIVFYASALALAASALGEYAGKLATQGLVTRAVHVAEIKIQEVLYAKDITLKAAGVAYILYKLTAWFSIWTLSFISTLLAFSAPPIYLKNQKQIDDAVGPVGSFI
Ogataea_philodendri	DLLTWKDPAASGKVFGSIVGSLLLLKYVNLLNILFKLAALVLSVSALAEYTGKFATQGFVTRSGEAAEVKVQEVLYSKDITLKVAGIAFIFYELTSFFSLWTLLLTATLVSFTAPVIYLGNKKQIDDAVGPVGSFI
Candida_boidinii_GF002	SLLTWKDPIISGKIFGALIGSLLFIKYFNIFTVFFYTSAVALAVSAIAEYSGKVATTGFITRATHIFEIEFQKLIYSSNITLKAAGYSYILYKLTSWFSIWTLIFTSVLIAFTVPPVYLKYQTEIDDAVGPVGSFI
Ogataea_methylivora	NLLTWTDPAKSGKVFGGIVATLLTVKFFNVFTVVFYFSALALGVSAVAEYLGKTFTVGFVTRASHVFEVYVQELLYSKDITFKAGCVAYILYKFTSWFSLWTIAFVSTIIAFTVPVVYLKNQKEIDEAVGPLSKFV
Ogataea_ramenticola	SLLTWKDPVKSGKVFGGIIAFLLVVKFFTITALLSFFTAVILGGSAIAEYAGKTVTTGFVTKGAKVIETVFQDLIYSKDITFKTAGVFYILYKLTSWFSLWTLTFLTTVIAFTIPAAYLKNQKQVDEAVGPVGSFI
Ambrosiozyma_maleeae_1	SLLLWTNPVKSGKFFGGVVVFLLLVKYINVLSVTFYLSSAVLLLSAAAQFVGKKVTQGFVTKAHHAIEGIAQDLIYSVKITLKFAGITYLLYKLTSWFSLWTIAFASTIVSFTAPVVYLKNQKVIDEAVGPVGKFI
Ambrosiozyma_maleeae_2	DLLTWANPVMSAKVFGSIVAFLLLVKKVNLLSVVFYLATLALLLSAAAEFVGQKVTQGLVTKSAKLAESQLQALFYSANLTLKFSAVNYFLYKLTSWFSLWTICFTAVVLAFSVPPVYLKNQKVIDEAVGPVSKFI
Ambrosiozyma_vanderkliftii	NLLTWTDPVASGKVFGSIIAFLTLFKYVNVLSLALYLGALALGVSALVEYASKKVTQGLVTKASHFVEASAQSLLFSADSTAKLAGVSYILYKLTSWYSLWSLLLVSTVLAFAIPPIYLKNKEVIDAAVGPVSKFI
Ambrosiozyma_kashinagacola	SLLTWSDPVASGKVFVSIIATLTLFKYVNILSFVFYLGALALAGSALAEFVGQKTVQGFVTKASHFVEAEVQDLLFSKNLTAKSAGVFYILYKLTRWYSLWSLLVVTTVLAFTVPLIYLKNKETIDAAVGPVSSFI
Ambrosiozyma_kashinagacola_TSA	SLLTWSDPVASGKVFVSIIATLTLFKYVNILSFVFYLGALALAGSALAEFVGQKTVQGFVTKASHFVEAEVQDLLFSKNLTAKSAGVFYILYKLTRWYSLWSLLVVTTVLAFTVPLIYLKNKETIDAAVGPVSSFI
Ambrosiozyma_ambrosiae	KLLTWSNPVCSAKVFVGIIATLVLIKYVNVPSLLLYLSSIVLGISGLAEFVSKKVTQGIISKSPRLFELFVQDLVYSVDLTAKFAGISFVLYKFTSWFSLWSLMLSFTLISFTVPAIYLKYKEIIDEFAGPVSTFV
Ambrosiozyma_philentoma	KLLTWSNPVCSAKVFVGIIATLVLFKYVNVPSLLLYLGSIVLGVSALAEFVSKKVTQGIISKAPRLFELFVQDLVYSVDLTAKFAGISFVLYKFTSWFSLWSLILTFTLVSFTIPAIYLKYKEIIDECAGPVSTFI
Ambrosiozyma_oregonensis	KLLTWSSPVCSAKVFVGIIATLVLFKYVNVPSLLLYLSSIVLSVSALAEFVSKKVTQGIVSKSPRLFELFVQELVYSVDLTAKFAGIAFVLYKFTSWFSLWSLILTFTLVAFTVPAIYLKYKEIIDELAGPISTLI
Ambrosiozyma_monospora	GLLTWKNPIESAKVFFGIIITLTLFKYVNVVSLVLYASTLVLAASAFAEFVGRKVTQGLISKASHFFELFLQDVIYSRDVTAKFSALAFIFYKLTSWFSLWSLSVFFTVVLFTIPPLYLGNKTLIDDSAAPVTKFI
Ogataea_nitratoaversa	DLFTWKVPAQSAKVFGAITGTLLLVKFVDILHILFYIAFLGLIASAVAEYAGKLTTEGFVTKAPSIVELSVQDLVYSRDISTKYGFGSYLLFKLTSWFSLWTLLFISTIAAFTIPLAYLSNQQEIDDAVGPVGQFV
Ogataea_pilisensis	DLFTWKSPAKSAKVFGSITGTLLVFKFVDILNILLYVTFLGLIASGIAEYAGRLTTEGFVTKASCVVEASAQDIIFAKDITIKFAFGSYLLYKLTSWFSLWTLLFISTVAIFSVPAIYLQNQKEIDDAIGPVGKFI
Candida_arabinofermentans	ALLTWEDPAKSGKVFGGIIGSLLLFKYVNLVKVISWLGFWGLAISLITEYGSKKFTQGLVTKATHVYEIAVQDLIYSVDFTLKATATFFAIFQLSKLFSLWTALFISTLIAFTVPFAYLKNKTQVDKAVGPVSSFI
Ogataea_naganishii	QLLTWADPAKSGSIVAGLLAFKLFNVLPVLFYLTAVALGVSAVAEYLGKTFINGFVSRASRALELKVQKLVYAEKITFKTAGLAYIAYKLTSWFSLWTLSFLVTIISFTIPAVYLQNQKQIDDTVGPIGGFV
Candida_succiphila	GLLTWKDPTRSGKYFGTIISTLLALKFINVFTIIFYFSAVGLAVAGAAEYLGVFFTTGFVTRGSHVAEKNIQELFYAKSITLWAALLSYVLYKFTSWISLWTLAFVSTLAAFTLPVIYLTNQKEIDHAVGPVGDFI
Starmera_amethionina	SLLTWKDPVKTGKVFGSIIATLLVLKSVNVLSLIFHLISIALVSSAAAEFVGKKVTTGFVTRLESFLENEGQKLIYSFDVTLKAAGGFYILYKITAWLSIFKILLISTVLAFSCPLIYQTYEKEINEAVGPISKFI
_Candida__stellimalicola	ALLTWADPVKTGKVFGSIIATLLVLKSVNILSLVFRLISLSLFTSAVAEYAGKKVTTGFVTRLKTVFEEQVQKLFYSFNVTLKFAGITYVLYKITSILSVFTILVSATVLAFTFPKFYRTYEKEIDEAVGPVSKFV
Starmera_quercuum	AFFTWKDPLKTGKVFGALIFSLIVLKSVNLVSLFFRLGALALVTSAVAEYAGKLVTTGFITRLQSFFEEEGQRLLYSFDITLKAAGLFYVLYKITSWLSLYKLIFISTILAFTVPLIYENYQKEINEIAGPVSKFI
_Candida__ponderosae	DLLTWKDPIKTGKVFGSLVTSLVVLKSVNLLSLFFRLGSLALIASAAAEYAGKATTTGFVTRIETFYEKQVQDLLYSLNITLQAGGLFYVLYKITSWISLFTIILTSTILAFTVPAVYEKYEVEINEAVGPVSKFI
Wickerhamomyces_alni	PLLTWDDPVKTGKVFGASVVALIVLKSVNLLSLFFRLGAYSLIASAAAEYAGKLVTTGFVSRLEALLESEVQKLLYSFNVTLKAAGLFYILFKITSWVSLYTLIFTSVVLAFTIPAVYEKYQDEINAAVGPVSKFI
Wickerhamomyces_canadensis	PLLTWKDPVKTGKVFGALIFALIVLKSVNLLSLFFRLGAYALFTSAIAEYAGKLATTGFVTRLESFLEQEGQKLLYSFNITLKASALFYVLFKITSWIALYKLIFLSTVLGFSVPAVYEKYQDEINAAAGPVSKFI
Wickerhamomyces_ciferrii	PLLTWKDPVKTGKVFGSLVFSLIVLKSVNLLSLFFRLGSIALISSAIAEYAGKLVTTGFVTRLESFLEEEGQKLLYSFDITLKASGLFYILYKITSWLSLYKLIFTATVLTFTLPAIYENYQDEINEAVGPVSKFI
Wickerhamomyces_anomalus	PLLTWKDPIKTGKVFGTLIVSLIVLKSVNLLSLFFRLGSIALISSAIAEYAGKLITTGFVTRLQSFLEEEGQKLLYSFNITLKASGLFYILYKITSWLSLYKLIFISTVLTFTLPAVYENYQDEINQAVGPVSGFI
Wickerhamomyces_bovis	PLLTWDNPVETGKVFGAVIFALVVLKSVNLLSLFFRLGFYALLTSAAAEYAGKIVTTGFVTRLEQLLEEEGQRLLYSFNVTLKTSGLFYVLYKITSWISLYNLIFISTVAAFTLPAVYEKYQDEINAALGPVSKFI
Candida_freyschussii	PLLTWKDPVRTGKVFGSLVFALIVLKSVNLLSLFFRIVSIALISSGLAEYAGKTLTTGFVTRLESFLEEQVQKLFYAANITLRAGALSFILYKITSWLSLYKLIFISTIWGFSLPAIYENNQVAINNAVGPVSKFI
Cyberlindnera_saturnus	PLLTWKDPVKTGKVFGAIVAALVVLKSVNLLNLFFRLGSYVLISSAVAEYAGKFITTGLVTRLNALLEEAIQKLVYAANITLKAGALFYILFKITAWLSLYKLIFAATISSFSLPAIYEQNQVQINAALGPISSFI
Cyberlindnera_suaveolens	PLLTWKDPVKTGRVFGAIIAALVLLKSVNLLNLFFRLGSYVLISSAVAEYAGKFITTGLVTRLNALLEETIQKLVYAANITLKAGALFYILFKITSWLSLYKLIFAATISSFSVPAIYEQNQVQINSAVGPISSFI
Cyberlindnera_mrakii	PLLTWKDPAKTGKVFGAIIAALVVLKSVNLLNLFFRLGSYVLISSAVAEYAGKFITTGLVTRLNALLEEAIQKLIYAANITLKAGALFYILFKITSWLSLYNLIFAATISSFSIPAIYEQNQVQINAAVGPISNFI
Cyberlindnera_jadinii	PILTWKDPVKTGKVFGTIIAALIVLKSVNLLNLFFRIGAFTLVTSAVAEYAGKFVTSGLVTRLECLLEESVQKLIYSADITLKAGALFYILFKITSWLSLNTLIFTGAILGFSLPAIYEKNQVQINAAVGPVSKFI
Cyberlindnera_fabianii	SILTWKDPVKTGKVFGGVIFALIIILKSVNLLSLFFRITSLTLISSGIAEYAGKVITTGFVTRLESLLEQSAQKLLYSANITLKTGALSYVLYKITSWLSLYSLIFTATILGFTLPFVYETYQTEINAAVGPVSKFI
Cyberlindnera_fabianii_Corr	SILTWKDPVKTGKVFGGVIFALIIILKSVNLLSLFFRITSLTLISSGIAEYAGKVITTGFVTRLESLLEQSAQKLLYSANITLKTGALSYVLYKITSWLSLYSLIFTATILGFTLPFVYETYQTEINAAVGPVSKFI
Cyberlindnera_xylosilytica	ALLTWKNPVETGKIFGGVIFALIVLKSVNLLSLFFRLGSFALLTSAVAEYVGKMVTTGFVTRLESFLEEGAQKLLYSANITLKAGVLSYVLYKITSWFSLYTLIFATTVLAFSVPFVYETYQTEINAAVGPVSKFI
Cyberlindnera_americana	SLLTWKDPIATGKIFGGVVSALVVLKSVNLLSLLFRVGSLALITSAIAEFAGKKITTGFVTRLECFLEEDVQKLIYSADITLKAGALSYVLYKITSWFSLYTLIFTATIATFSVPYVYETYQTEINAVVGPVSKFI
Cyberlindnera_petersonii	PLLTWKDPIATGKVFGGVVCALIVLKSVNLLALFYRVAFVALLTSAAAEYVGKLVTTGFVTKLESLLEEESQKLLYSANVTFKAGVLSYILYKITSWFSLYTLIFTSTVLAFTLPFVYETYQTEINEAVGPVSKFI
Cyberlindnera_maclurae	PLLTWKDPVKTGKVFGAIVGALILFKSVNLLNLYFHIVSVALIAAAAAEYAGKLVTTGLVTRLESIIEDAVQKIVYAANITLKVGVLSYILFKITSWASLYTLIVTGTVLGFTVPAVYENNKKEIDAAVGPVSKFV
Barnettozyma_hawaiiensis	PLLTWNDPVKTGKVFGSVVAALIVLKSVNLLSLLFRITSMALLASAVAEYGGKLVLQGLVTKLEKLLESEGQKLLYAVSVTAKASGLFYILYKITSWISLYTLVFTAVVSAFTLPFIYEKYQVEINAAVGPVSSFI
Barnettozyma_californica	PLLTWNDPVKTGKVFGSLIAALVVFKSVNLLSLFFRIASVALFASAIAEYGGKLVLQGLVTKLEKILESEGQKLLYAANITAKSAGLFYILYKITSWVSLYTLVFTAVIAAFTAPFIYEKYQVEIDAAVGPVSSFI
Barnettozyma_populi	PLLTWNDPVKTGKVFGSLIAALVVLKSVNLLSLFFRIASVVLFASAIAEYGGKLVLQGLVTKLENLLESEGQKLLYAVNITAKSAGLFYTLYKITSWVSLYTLIFTTVIAAFTVPFIYEKYQVEINAAIGPVSSFI

Barnettozyma_salicaria	PLLTWKDPIKTGKVFGSLIAALIVLKSVNILSLFFRVAAMALFASAVAEYGGKLILQGLVTKLERFLETQGQELLYSANVTLKSSALFYILYKVTSWFSLHTLITTSVISSFTLPLAYEKYQDEINAALGPVSKFI
Barnettozyma_pratensis	PLLTWKDPVKTGKVFGGIVAALIVLKSVNLLSLFFRLSSLALFVSAVAEYGGKLILQGLVTKLESFLETEGQELLYSVNVTLKSAGLFYILYKITSWVSLYSLIFTSVVAAFSLPFVYEQYQDEINAGLGPVSKFI
Phaffomyces_thermotolerans	PLLTWKDPVQSGKVFGGIIAALVLLKSVNLLSLFFRLASLVLAISATAEYGGKLILQGLVTKVQSFLETEGQELLYSINITLKSAGLFYVLYKITSWISLYNLIFTSVVLSFTLPFIYEKYQTEINASLGPVSKFI
Phaffomyces_opuntiae	PLLTWKDPIQSGKVFGGIVFALIIILKSVNLLSLFFRLASLVLAISASAEYGGKLILQGLVTKLKTFLETEGQELLYSNNITLKSAGLFYILYKITSWVSLYNLVFTAVVSAFTLPFVYEQYHDEINAGLGPVSKFI
Phaffomyces_antillensis	PLLTWKDPIHSGKVFGGIITALVLLKSVNLLSLFFRLASLVLAISASAEYGGKLILQGLVSKLKSFLEAEGQELLYSFNITLKSAGLFYILYKITSWVSLYSLVFTAVVSAFTLPFIYELYQDEINAGLGPISKFI
Wickerhamomyces_hampshirensis	PLLTWKDPIATAKVAGGILFSLISLKVLNIVKLFFHLGYLALISSAIAEYAGKLVTTGFVTRLDGFLEDAAQKLIFAQNITLKTGVAFYILYKITSWLSLFNIILVSTLGVFTLPVIYETYQTEINALVGPVSKFI
Komagataella_pseudopastoris	SILTWKNPVKTGLVFGGIIVALLLIRFVNVFKLAFRLGTYALFATASAEFIGKIAVQGFISRGDHIFELKFQQLFYAENVTFKAAGISYILYILTGLISVWTLLFVSTLLVFTVPKLYEVYQKEIDQAVGPVGKYV
Komagataella_populi	SILTWKNPVKTGLVFGGIIVALLLIRFVNVFKLAFRLGTYALFATASAEFIGKIAVQGFISRGDHIFELKFQQLFYAENVTFKAAGISYILYILTGLISVWTLLFVSTLLVFTVPKLYEVYQKEIDQAVGPVGKYV
Komagataella_phaffii	SILTWKNPIKTGTVFGGIIVALLLIRYVNVFKLVFRLATYVLFATASAEFIGKIAVQGFISRADHVFELKFQQLLYAENVTFKAAGISYILYILTGLISVWWLLFVSTLVVFTVPKLYEIYQKEIDEAVGPVGKYV
Komagataella_pastoris	SILTWKNPIKTGTVFGGIIVALLLIRYVNVFKLVFRLATYVLFATASAEFIGKIAVQGFISRADHIFELKFQQLLYAENVTFKAAGISYILYILTGLISVWWLLFVSTLVVFTVPKLYEVYQKEIDEAVGPVGKYV
Ogataea_trehalophila	ALLTWKDPRKSANVFLATTTILLLIKYINVINVVSYIAFLALLASAAAEYAGKKLTEGFVTKAEHAAEIFLQDVIYSKNITFRFGLIAYVAYKLTSILSLWTLLFVTNVLAFSIPFIYLTYETEINQLYKPATSFI
Ogataea_methanolica	ALLTWKNPQKSAKVYLATTTVLLLVRYINVINVALHIACLGLLGSAAVEYTSKQIIEGLVTKAEHAVEIFLQDLVYSKNITFRFGVIAYIAYKLTSILSLWTLLFVTNILAFSVPFFYLTYETEINQLYKPVTSFI
Cephaloascus_albidus	ALLTWKNPIETGKVFGSIVVSLIVLKYVNLVNLFFRLAWVALLASAAAEYSGKLVTTGFVTKLPNLLEAEVQKIIFSADITLKAGGVSYILYKITSWLSFYTILVSSVVLAFTVPAIYSNFKTEIDQAIGPVGSFI
Cephaloascus_fragrans	ALLTWKNPIETAKVFGSIVVSLIVLKYVNLINLFFRFAWVALLASAAAEYSGKLVANGFVSKLPDLLETEVQKIVFSADVTLKAGGISYILYKITSWFTLYTILVTSIVLAFTVPAIYENFKVEIDNFVGPVGSFI
_Candida__fragi	SLLTWKDPIKSGKVFGGAVASLIIFKYVHLLNVFFRLAYIGLLGSAAVEYISKLVTEGLISKLPAIFFPEVQKLVFSTNVTLKAAGGAYILYKVTSWFSLYTLAFASIVIAFTVPAVYFANRKEIDAAVGPIGDFI
Kurtzmaniella_cleridarum	SLLTWKDPIKSGKVFGGAVASLIIFKYVNLLNVFFRLAYMGLLASATIEYASKLVTEGIVSKLPAIFFPQVQKLVFSTNVTLKAAGGAYILYKVTSWFSLYTLAFASIVIAFTVPAFYFANQKEIDAAVGPIGDFI
_Candida__athensensis	ALLTWEDPARTGKVFGAIVAVLVVFKTVNLFNVFFHVAYLGLLVAAAAEYAGKLVTQGFVTKLPEVLEEQIQKIIYAHDITLKAAGVSYVLYKVTSWFSLYTLLFAAVVLTFTLPAVYVHNKKEIDAAVGPLGSFV
Meyerozyma_guilliermondii	ALLTWQDPARTGKVFGGIVATLIVFKTVNLFNVFFHVAYLGLTVAAAAEYAGKLVTQGFVTKLPQVFEEQIQKVIYAHDITLKAAGLSYVLYKITSWFSLYTLVFAGVVLTFTLPAVYVHNKKEIDAAVGPLGSFI
Priceomyces_haplophilus	DLLTWKDPVKTGKIFGAIVFTLFVFKKVNLFNVFFHLAYLGLLLTAAAEYVGKLFTKGFVTKLPSVLEEEIQKIVYAHDITLKAAGFSYLLYQVTSWFSLFTLISIAVVGLFTVPAIYVRNKKEIDAAVGPVGSFI
Priceomyces_castillae	DLLTWKEPLKTGKVFGAIVLTLFVFKKVNLFNVFFHLAYLGLLLTAAAEYAGKVVTQGFVTKLPCVLEEEVQKIVYAHDITLKAAGLSYLLYKVTSWFSLFTLIAVAVVGVFTIPAIYVRNKKEIDAAVGPVGSFI
Priceomyces_medius	DLFTWKDPVRTGKVFGTFVVTLFVFKKVNLFNVFFHLAYIALLVTAAAEYAGKLVTQGFVTKLPNVLEEEIQKIVYAHDITLKAAGLSYLLYKVTSWFSLFTLITIGVFALFTIPAIYVRNKKEIDAAIGPVGSFI
Priceomyces_carsonii	DLFTWKDPVKTGKVFGSIVLALLVFKKVNLFNVFFHLAYIGLLVSAAAEYLGKLLTQGFITKLPCVAEDKIQEILYAHDITLKGAGISYILYKITSLFSLFTLITAGVVILFTIPAIYVRNKKEIDAAVGPVGSFI
_Candida__rhagii	DLLTWKDPIKTGKVFGGLVAVLVIFKKVNLFNVFFHLAYIGLLLSAAAEYSGKLVTQGFVTKLPQFLEQKFQKIIYAHDITLKAAGFSYILYKVTSWFSLYTLVFAAVVVAFTAPAVYSKNKKEIDAAVGPVGSFI
Hyphopichia_heimii	DLLTWKDPIKTGKVFGGLVAVLVIFKKVNLFNVFFHLAYIGLLLSAAVEYSGKLVTQGFVTKLPQFLEQQFQKIIYAHDITLKAAGLSYILYKVTSWFSLYTLVFAAVVIAFTAPAVYSKNKKEIDAAVGPVGSFI
_Candida__gotoi	DLLTWKDPIKTGKVFGGLVAVLVIFKKVNLFNVFFHLAYIGLLLSAAVEYAGKLVTEGFVTKLPHVLEQQFQKIIYAHDITLKAAGLSYVLYKITSWFSLYTIVFAVVVLVFTVPVIYSKNKKEIDAAVGPVGSFI
Hyphopichia_burtonii	ELLTWKDPVKTGKVFGSIVLALVVFKKVNLFNIFFHLAYIGLLASAAAEYAGKVVTQGFVTKLPHLIEGQFQKIVYAHDITLKAAGISYVLYKVTSWFSLYTLISIAVVLAFTGPAIYTRNKKEIDAAVGPVGAFI
Danielozyma_ontarioensis	SLLTWRDPVYTGKIFGAIVAGLVIFKKVNLFNVFFHIAYIGLLVSAAAEYAGKLVTQGFVTKLPLVLEGQFQKVVYAHDITLKAAGISYLLYKVTSWFSFFTLLSFTVVLAFTWPAIYTRYKTEIDGAVGPVGSFI
Debaryomyces_fabryi	DLLTWKDPVKTGKVFGTIILTLLVLKTVNLFNIFFHVAYIGLLVSAILEYVGKFVVEGFVSKLPLVFEAEIQKILYAHDITLKAAGISYILYKITSLFSLYTLITIVVVLAFTVPAVYVRNKKEIDAAIGPVGAII
Debaryomyces_hansenii	DLLTWKDPVKTGKVFGTIILTLLVLKTVNLFNVFFHVAYIGLLVSAILEYVGKFVVEGFVSKLPLVFEAEIQKILYAHDITLKAAGISYILYKITSLFSLYTLITIVVVLAFTVPAVYVRNKKEIDAAIGPVGAII
Candida_psychrophila	NLLTWKDPVKTGKVFGTIMFTLLVLKTVNLFNIFFHIAYIGLLVSAIFEYVGKFVVEGFVSKLPLVFEAEIQKILYAHDITLKGAGISYIMYKITSLFSLYTLITIVVVLAFTVPATYVRNKKEIDAAIGPVGALI
Debaryomyces_nepalensis	DLLTWKDPVKTGKVFGTIIVTLLVLKTVNLFNIFFHVAYVGLLISAILEYVGKFVVEGFVSKLPHVFEQEIQKILYSHDITLKGAGISYILYKITSLFSLYTLITIAVVLAFTVPAVYVRNKKEIDAAVGPVGNLI
Debaryomyces_maramus	NLLTWKDPVKTGKVFGTIVVTLLVLKTVNLFNVFFHVAYIGLLLSAVFEYAGKLVVQGFVSKLPHVLETEIQKILYAHDITLKGAGISYILYKVTSLFSLYTLIFVAVVLLFTVPAIYVRHKKEIDAAIGPVGAYI
Millerozyma_acaciae	DLLTWKDPVKTGKVFGTLLLTLIVFKTVNLFNIFFRLAYIGLLVSAAAEYAGRLITQGFVTKLPHIFEEEVQKIVYAHDITLKGAGVSYVLYKITSFLSLYSLLFTALVLTFTAPAVYVKNKKEIDAAVGPLGSYL
Millerozyma_farinosa	DLLTWKDPVKTGKVFGGLILILLIFKTVNLFNVFFHLAYLGLLVSAAAEYAGRLVTTGFVTKLPRLLEEEVKKILFAHDITLKGAALSYILYKVTSYFSLYALVTTVVVLTFTLPAVYVKNKKEIDAAVGPLGTFV
Suhomyces_emberorum	NLLTWKDPVKTAKVFGGVIGGLVIFKTINLFTIFFRLAYLGLLVSAAAEYAGKFVTQGFVTKLPLVAEQEYQKIVFSHDITLKAAGVSYVLFKLTSWFSLFTLFTASVVLVFTAPVFYLKNKKQIDALVGPVGAFI
Suhomyces_canberraensis	TLLTWKDPIKTGKVFGGVVVGLFVFKTINLFNIFFHLAYIGLLVSAAAEYAGKLITQGFVTKLPLIAEQEYRKIIFAHDITLKAAGTSYVLYKLTSWFSLFTLITASVVLLFTVPAIYVKNKKQIDALVGPVGNFI
Suhomyces_tanzawaensis	SLLTWKDPVKTGKVFGGVVAGLLIFKTINLFNIFFHLAYIGLLLSAAAEYAGKLVTQGFVTKLPLVAEQEYQKIVFAHDITLKAAGVSYVLFKVTSWFSLFTLITTAVVLLFTVPAFYVHNKKQIDALVGPVGNFI
Suhomyces_pyralidae	SLLTWEDPIKTGKVFGGILAGLLVFKTVNLFNIFFRLAYIGLLISAAAEYSGKFITQGFVTKLPAVAEEEYQKVVFSHDITLKAAGLSYVLYKVTSWFSLYTLVTSSVVLLFTVPAVYVQNKKQIDALVGPVGSFI
Aciculoconidium_aculeatum	DLLTWKDPVKTGKIFGSIIASLFVFKTINLFNVFFRLAYMGLLVSAAVEYVGKLVTQGFVTKLPLVLEQEFQKIVYAHDITLKAAGISYVLFKITSWFSLFTLIATGVILAFTLPAFYVKNKKQIDALVGPLGSFI
Kodamaea_laetipori	ELLTWKDPVKSGKVFGSIIVGLVVFKTVNLFNVFFHLAYLGLLLSAAVEYAGKFVTQGFVTKLPLILESEFQKIVYAHDITLKAAGVSYILFKITSWFSLFTLVATSVVLAFTVPAVYLKNKKQIDALVGPVGTFI
Yamadazyma_scolyti	DLLTWKDPIKTGKIFGSIVAVLISVKAINLLNIFFYFAFVGLILSAAAEYAGKLVTQGFVTKLPAILEADVHNIIYSHDITLKAAGLSYILYKLTSWFSLFTLIATSVVLLFTVPVIYTKNKKEIDAAVGPVGSFV
_Candida__tammaniensis	DLLTWKDPVQTGKVFGSLVLALVVFKKINLINIFFHLSYIALLFSAAGEYAGKLITQGFVTKLPSILEDEFHKIVYSHDITLKAAGLSYILYKITSWFSFFTLLTASIVLLFTVPVVYVKNKKEIDAAVGPVGSFL
_Candida__gorgasii	DLLTWKDPVQTGKVFGSIIAGLVIFKSVNLINIFFHLSYIALLFSAAGEYAGKLVTQGFVTKLPSILEEEFQKIVYSHDITLKAAGVSYILYKVTSWFSFFTLLTASVVLLFTVPVVYVKNKKEIDAAVGPVGAFV
Yamadazyma_tenuis	ELLTWQNPIKTGKVFGSLILGLVVFKKVNLINIVFHLTYIALLFSAAAEYAGKLVTEGFVTKLPAIAEDVTNKVIYSHDITLKAAGVSYVLYKLTSWFSVFTLLTTALVLAFTFPVVYSKNKKEIDAAVGPVGSFI
Yamadazyma_laniorum	DLLTWKDPIKTGKVVGSIIVGLVVFKKVNLLNIFFHLSYVALLFSAAAEYAGKLVTEGFVTKLPAILEENVHKIVYSHDITLKAAGVSYVFFKLTSWFSLFTLVTSALVVLFTFPVVYSKNKKQIDAAVGPVGAFV
_Candida__aaseri	DLLTWKDPVKTGKVFGITVGSLIVFKMVNLINIFFHLSYIALLFSAAAEYAGKLVTQGIVTKLPAIAEGEFYKIIYSHDITLKAAGCSYVLYKLTSWFSIFTLLATSVILLFTVPIVYIKYQKEIDAAVGPVGAFV
Yamadazyma_nakazawae	ELLTWKDPVKTGKVFGSVVAGLAVFKMVNLINIFFHLSYLALLLSAAAEYASKLVTQGFVTKLPAILEATGHKIIYSHDITLKAAGVSYVLYKLTSWLSFFTLLTTAVVLLFTVPAVYQKNKKEIDAAVGPVGAFF
Yamadazyma_philogaea	HLLTWQDPVYTGKVFGSIMLGLIAFKTTNFLNIFFHLAYLGLLVSAAAEYVGKLVTQGFITKLPAVLEDVSDKVIFARDITLKVAGVSYVLYKLTSFLSFFALLTIAVVLLFTGPAIYQKNKKEIDAAVGPPGAFV
Teunomyces_kruisii	SLLTWKDPVKTGKVFGSIVAALLVLKTVNLFNLFFRLAYIGLLVSAAAEYSGKLVTQGFVTKLPSLLESEFQKIIYAHDITLKAAGLSYVLYKITSWFSLYTLVFAAVIVAFTAPAVYSKNKKEIDAAVGPVGNFI
Teunomyces_cretensis	SLLTWKDPVKTGKVFGSIVAALLVLKTVNLFNLFFRLAYIGLLVSAAAEYSGKLVTQGFVTKLPALVESEFQKIIYAHDITLKAAGLSYVLYKITSWFSLYTLVFAAVIVAFTAPAVYSKNKKEIDAAVGPVGNFI
Teunomyces_gatunensis	SLLTWKDPVKTGKVFGSIIATLLVVKTVNLFNLFFRLAYIGLLVSAAAEYAGKLVTQGFVTKLPSILETTVQKIIYAHDITLKAAGVSYVLFKITSWFSLYTLVFAAVIVAFTAPVIYSKNKKEIDSVVGPVGQFI
Scheffersomyces_stambukii	DLLTWKDPVKTGKVFGGLVLGLIVFKKVNLFNVFFHLAYIGLLISAAAEYAGKLVTKGFVTALPHLAEEEFNKIVYAHDITLKAAGVSYVLYKLTSWFSFFTLLASSIVLLFTVPFIYTTFKKEIDAAVGPVGSFI
Scheffersomyces_lignosus	SLLTWKDPVLTGKVFGSIISSLVVLKYINLINLFFRGAYVGLLLSAAAEYAGKLVTQGFVTKLPQLVEEEFQKIVYSHDITLKAAGLSYIIFKLTSWFSLFTLVAASVVVLFTVPVIYQSNKKEIDAAVGPVGKFV
Scheffersomyces_shehatae	SLLTWKDPVLTGKVFGSIIVSLIIILKYINLINLFFRGAYVGLLLSAAAEYAGKLVTQGFVTKIPQLIEEEFQKIIYSHDITLKAAGLSYILYKLTSWFTLFTLIAASVVILFTVPVIYQTNKKEIDAAVGPVGKFV
Candida_dubliniensis	DFLTWKNPIKTGKVFGSLVFGLIILKTVNLFNIFFHLAYIGLLVSAAAEYSGKLITKGFLANLPELLEESFNKIVYSHDITLKAAGISYVLYKLTSWFSLYTLIFIFVVLIFTVPVIYKTYKKEIDAAVGPIGSFI
Candida_albicans	DFLTWKNPIKTGKVFGSLVFGLIVLKTVNLFNIFFHLAYIGLLISAAAEYSGKLITKGFLANLPELLEESFNKIVYSHDITLKAAGISYILYKLTSWFSLYTLIFIFVVLIFTVPVIYKTYKKEIDAAVGPIGSFI
Candida_africana_TSA	DFLTWKNPIKTGKVFGSLVFGLIVLKTVNLFNIFFHLAYIGLLISAAAEYSGKLITKGFLANLPELLEESFNKIVYSHDITLKAAGISYILYKLTSWFSLYTLIFIFVVLIFTVPVIYKTYKKEIDAAVGPIGSFI
Candida_tropicalis	DLLTWKNPIKTGKIFGAIIFGLIIFKTVNLFNIFFHLAYIGLLVSAAAEYSGKLITKGFLSNLPELFEESLNRIVYAHDITLKAAGLSYVLYKLTSWFSLYVLIFVSVILVFTVPFIYTTYKKEIDAAVGPVGAFV
_Candida__schatavii	SLLTWRDPASTGTVFGGILAVLVVLKYVNLLNLFFHLSYLGLLVAAAAEYAGKLVTQGFVSKLPAVLEAEAQKIVYAHDITLKAAGLSYILYKLTSWLSLYTLIVTAVVSIFTLPAAYSYNKKEIDAAVGPLGAFI
Kodamaea_ohmeri	QLLTWKDPAYSAKVFGSIIVGLFVFKTVNLINVFFHLAYLALLVSAAVEYAGKFVTQGFVTKLPLVLEEEFQKIVYAHDITLKAAGISYVIYKLTSLISFFNLIATAVVLAFTIPGIYLQNKTEIDNIIGPVGSFI
_Candida__restingae	QLLTWKNPAQTAKVFGSIVVGLFVFKTVNLVNVFFHLAYIGLLVSAAVEYAGKFVTQGFVTKLPLIAEVELQKIFYAHDITLKAAGISYIVYKITSWFSFFTILATAVVLAFSVPGIYLQNKTQIDQLVGPVGAFI
Candida_corydali	ALLTWKDPVHTGKVFGSILLTLTVFKTINLFNIFFHLAYIGLLISAAAEYSGKLIIKGFLENLPNLIESTLKKIVYSHDITLKAAGISYILYKLTSFVSLFTLITISVVLIFTGPFVYTTYKKEIDAFIGPVGQFV
Spathaspora_passalidarum	DLLTWKNPIKTGKVLASIVVALVVFKTVNLINLFFHLSYIGLLVSAAVEYVSKVVTKGLITALPHLLEERFRKIVYAQDITLKAAGVSYIAFKLTSWIPLFNLVVAAVILLFTAPFIYTTYKKEIDAAIGPLGDFV
Spathaspora_girioi	DLLTWKDPIKTGKVLVSIIAALVVFKTVNLLNLFFRLSYVGLLVSAAVEYVSKVVTKGLITTLPHLFEEKFRKIVYAQDITLKAAGVSYIAFKLTSWISIFTLISIAVGLLFTVPFVYTTYKKEIDSIVGPLGDFI
Spathaspora_arborariae	DLLTWKNPIKTGKVLVSIIVALAVFKTVNLINLFFHFSFVALLVSASVEYISKIVTKGLITTLPHLLEEQFRKIVYAQDITLKAAGVSYIGYKLTSWIPIFTLVSISVGLAFTVPFIYTTYKKEIDAVVGPLGDFI
Spathaspora_hagerdaliae	DLLTWKDPIKTGKVFGSIIISLIVFKTVNIFNVLFHVAYIGLLLTAAVEYISKVVTKGLITTLPHLVEQQFKKIIFAENPSLKAAGVSYILYKLTSWFSIFTLITASVVLLFTLPFVYTTFKKEIDAAVGPVGEFI

Spathaspora_gorwiae	DLLTWKDPIKTGRVFGSIIISLIVFKTINLFNVLFHVAYIGLLITAGVEYVSKMVTKGLITTLPHLVEQQFKKIIFAENPTLKAAGVSYILYKLTSWFSIFTLITTSVVLLFTLPFVYTNFKKEIDAAVGPVGEFI
Spathaspora_xylofermentans	DLLTWRYPIKTAKVFGSIIIALTVFKTVNIINLLFRIGFYGLIAAAIVEYSTKLITKGLVSSLPHLFEQRLKSIVFVENVTLKAAGISYIVYKLTSWFSIFTLITTITVLLFTLPVIYTTYKKEIDATIGPVGEFI
Spathaspora_boniae	TLLTWKNPVETAKVFGAILLTLAVVKYVNLFNIVFRFGYIGLLISAAAEYGSKLVTKGIVTSVPQLAEDEFTKIVYAHDVTLKAAGVSYVLYKLTSWFSVFTLVAVSVVLLFTVPFAYQSNKKEIDAAVGPLGKFI
_Candida__ascalaphidarum	DLLTWKDPIKTGKVFGGSFLGLLSLKYINLLNIVFHFAYIGLLLSAAAEYAGRLVVQGFVTKLPCLLEFHVQNVVYSHNLTLKAAGLSYILYKATSYLSLFTLVTVALFLTFTLPVIYISNKKEIDAAVGPVGDYI
Clavispora_fructus	QLLTWQNPTATAKVFGAIIATLLLVK-INVVNHLFHLAYIGLLVAAAAEYSGKLATQGFVTKLPSLLEAKLHKVVYAQDVTLKAAGVSYIFFKLTSWFSVYSLIFASVILAFSGPFVYQKNQKEIDAAVGPVGSFI
Clavispora_lusitaniae	SLLTWKDPVATGKVFGAIIGALLLVK-INVANHFFHLAYIGLLAAAAAEYAGKLVTQGFVTKLPALAEKRLHKIIFAQDITLKAAGAAYILFKLTSWFSVYSLIFTSVLLTFSVPLVYQSNKKEIDAAVGPVGNFI
_Candida__wancherniae	SLLTWKDPVATGKVFASILGALILLK-VNVFNHVFHFAYIGLLIAAAAEYSGRFLTQGFIKKLPALIESEVQRIVYGQDITLKAAGVSYILYKLTSWFSLYTLLVAAVLLAFSFPVFYQSNKKEIDAAVGPVGSFI
_Candida__golubevii	SLLTWKDPAATGKVFGAIIAGLILLK-VNIVNHVFHLAYLGLLAAAAAEYSGRVLTQGFVRKLPALVEAEVHRVVYAQNITLKAAACSYILYKLTSWFSLYSLVFAAVVLVFTLPALYESNKKQIDAAVGPVGDFI
Metschnikowia_reukaufii	ALLTWKDPVATGKVFGSIVGALVLTK-VNVFNHLFHVSYLALLAAAAAEYSGRVLTQGFVTKLPALIEAGVHKVVYAQNITLKAAGVSYILYKLTSWFSLYSLIFATVILAFTGPFVYQSNKKEVDAAVGPVGSFI
Metschnikowia_fructicola	SLLTWRDPAATGKVFGAIISALILTK-VNVFNHLFHLAYLALLAAAAAEYAGRVLTEGFVKKLPAVLETEVHRVVYAQNITLKAAGSSYILYKLTSWFSLYTLIFATVLLAFSVPYVXQSNKXEVDAAVGPVGSFI
Metschnikowia_bicuspidata	SLLTWKDPVATGKVFGAIVAALVLAK-VNVVNHVFHLSYLALFAAAGSEYAGRILTQGFVQKLPAVLEAEFHRVVYAQNITVKAALVSYMMYKLTSWFSVYALVFATVLLAFSVPYVYQTNKKNVDAAVGPVGAFV
Metschnikowia_australis	SLLTWKDPIVTGKVFGAIVATLVLTK-VNVVNHVFHLSYLALFAAAGSEYAGRILTEGFVHRLPAVLEAEFNRVVYAQNITVKAALVSYMMFKLTLWFSVYALVFVTVLLVFSVPYVYQTNKKNVDVAVGPVGAFV
_Candida__hawaiiana	DLLTWKDPIATGKVFGATLGSLILIK-INIFSHVFHLAYLALLASAAAEYAGRFLTQGFVSKLPALLEAESQKVIYAKDLTLKTAGASYVLYKLTSWFSLYTLLVVSVLLAFSVPFVYNNNKKEIDAAVGPVGSFV
Metschnikowia_orientalis	DLLTWKDPIASGKVLGATLGSLILVK-INIFSYVFHLAYIALLASAAAEYAGRFLTQGFVSKLPALIEVESQKIIYAKDLTLKTAGVSYILYKLTSWFSLYTLVFVSVILAFSVPFVYVSNKKEIDAAVGPVGSFI
Metschnikowia_lochheadii	ELLTWKDPVTTGKIFGATLGSLILVK-INILNHLFHLAYVALLVSAAAEYFGRFLTQGFVSKLPALIEAKAHQIIFAKDLTLKTAGVSYILYKLTSWFSLYSLIFVSVLLAFSVPLVYQNNKKEIDAAVGPVGTFV
_Candida__ipomoeae	ELLTWKDPVTTGKIFGATLGSLILVK-INILNHLFHLAYVALLVSAAAEYSGRFLTQGFVSKLPALIEAKAHQIVFAKDLTLKTAGVSYILYKLTSWFSLYSLIFVSVLLAFSVPLVYQNNKKEIDAAVGPVGAFV
Metschnikowia_santaceciliae	ELLTWKDPVTTGKIFGATLGSLILVK-INILNHLFHLAYVALLVSAAAEYSGRFLTQGFVSKLPALIEAKAHQIIFAKDLTLKTAGVSYILYKLTSWFSLYSLIFVSVLLAFSVPLVYQNNKKEIDAAVGPVGAFV
Metschnikowia_kamakouana	ELLTWKDPVTTGKIFGATLGSLILVK-INILNHLFHLAYVALLVSAAAEYSGRFLTQGFVSKLPALIEAKAHQIIFAKDLTLKTAGASYILYKLTSWFSLYSLIFVSVLLAFSVPLVYQNNKKEIDAAVGPVGAFV
Metschnikowia_colocasiae	ELLTWKDPVTTAKIFGATLGSLILVK-INILNHFFHLAYVALLVSAAAEYSGRFLTQGFVSKLPALIEAKSHQIIFAKDLTLKTAGASYILYKLTSWFSLYTLIFVTVLLAFSVPLVYQNNKKEIDAAVGPVGAFV
Metschnikowia_hamakuensis	ELLTWKNPVTTGKIFGATLGSLILVK-INILNHLFHLTYVALLVSAAAEYSGRFLTQGFVSKLPALIEGKAHQIIFAKDLTLKTAGASYILYKLTSWFSLYSLIFVSVLLAFSVPLVYQNNKKEIDAAVGPVGAFV
Metschnikowia_hawaiiensis	ELLTWKDPVTTGKIFGATLGSLILVK-INILNHLFHLGYVALLVSAAAEYSGRFLTQGFVSKLPALIEGKAHQIIFAKDLTLKTAGASYILYKLTSWFSLYSLIFVSVLLAFSVPLVYQNNKKEIDAAVGPVGAFV
Metschnikowia_drakensbergensis	QLLTWKDPVTTGKIFGATIGSLILVK-VNILNHVFHLAYVALLISAAAEYSGRFLTQGFVSKLPALIEAKFHQVVFAKDLTLKTAGVSYILYKLTSWFSLYSLIFVSVLLAFSVPLVYQNNKKEIDAAVGPVGAFV
Metschnikowia_proteae	QLLTWKDPVTTGKIFGATIGSLILVK-VNILNHVFHLAYVALLISAVAEYSGRFLTQGFVSKLPALIEAKFHQVVFAKDLTLKTAGVSYILYKLTSWFSLYSLIFVSVLLAFSVPLVYQNNKKEIDAAVGPVGAFV
Metschnikowia_hibisci	ELLTWKNPVTTGKIFGATLGSLILVK-INIFNHVFHLAYVALLISAAAEYSGRFLTQGFVSKLPALIESKFHQIIFAKDLTLKTAGVSYILYKLTSWFSLYSLLFVSVLLAFSVPLVYQNHKKEIDASVGPVGSFV
Metschnikowia_aberdeeniae	ELLTWKDPITTGKIFGATLGSLILVK-INILNHIFHLAYVALLISAAAEYSGRFLTQGFVSKLPALIEAKFHQIIFAKDLTLKTAGVSYILYKLTSWFSLYSLLFVSVLLAFSVPLVYQNNKKEIDAAVGPVGSFV
Metschnikowia_shivogae	ELLTWKDPVTTGKIFGATLGSLILVK-INILNHVFHLAYMALLISAAAEYAGRFLTQGFVSKLPALIETKSHQIIFAKDLTLKTAGVSYILYKLTSWFSLYSLLFVSVLLAFSVPLVYQSHKKEIDAAVGPVGSFV
Metschnikowia_kipukae	ELLTWKDPITTGKIFGATIGSLILVK-INIFNHIFHLAYLALLVSAAAEYSGRFLTQGFVSKLPALIESQFHKIIFAKDLTLKTAGVSYILYKLTSWFSLYSLAFVTVLLAFSVPLVYQNNKKEIDAAVGPVGAFV
Metschnikowia_similis	EVLTWKDPVATAKIFGAILGSLILVK-VNILNHVFHLAYVALLISAAAEYSGRFLTQGFVSKLPALIEVKAHQVIFAKDLTLKTAGVSYILYKLSSWFSLYSLIFGTVLLAFSAPPVYKQNKKEIDAAVGPVGSFV
Metschnikowia_dekortorum	EVLTWKDPVVTAKIFGAILGSLILVK-VNILNHLFHLAYVALLISAAAEYSGRFLTQGFVSKLPALIEVKAHQIIFAKDLTLKTAGASYILYKLSSWFSLYSLIFGTVLLAFSAPPVYKQNKKEIDAAVGPVGSFV
Metschnikowia_bowlesiae	EVLTWKDPVVTAKIFGAILGSLILVK-VNILNHLFHLAYVALLISAAAEYSGRFLTQGFVSKLPALIEVKAHQIIFAKDLTLKTAGASYILYKLSSWFSLYSLIFGTVLLAFSAPPVYKQNKKEIDAAVGPVGSFV
Metschnikowia_arizonensis	ELLTWKDPVTTGKIFGAIIGSLILVK-INILNHFFHLAYVALLVSAAFEYSSRFVTQGFVSKLPALIETKFHKIIFAKDLTLKTAGVSYILYKLTSWFSLYSLIFSAVLLAFSVPLVYENNKKEIDAAVGPVGAFV
Metschnikowia_drosophilae	SLLTWRDPVATGKVFGSIVLALILVK-VNIFSHLFHVAYLGLLASAAAEYAGRFLTQGFITKLPALIESEVHTLVYAKDMTLKAAGVSYLLYKLTSWFSLYSLVFAVVILAFSVPFVYQGNKKEIDAAVGPVGAFV
_Candida__torresii	MLLTWQDPVATGKVFGSTVLALILVK-INIFSHLFHVAYLGLLASAAAEYAGRFLTQGFITKLPALIESEVHTLVYAKNMTLKAAGVSYLLYKLTSWFSLYSLVFAVVLLAFSFPPVYQGNKKEIDAAVGPVGAFV
_Candida__intermedia	ALLTWQDPVATGKVFGALIGALILFK-VNVAPLVFHGLYIGLLVAAAAEYAGKLLTQGFVTKLPAVLEAYVHKVAFAQDITLKAAGVSYILYKLTSWFTVYSLLIVTVLLAFSFPPFYQANKKEIDAAVGPLGSFI
_Candida__blattae	YVLGWHDPVATGKLFFSIIAGLILVK-VNVVSIGFHALYIALLLSAAAEYIGKLVTQGFVTKLPAIAETEIYKVVFAQDITLKAAGVSYILYKLTSWFSVYTLVFATVLLAFSFPPFYQRNKKEIDAAVGPVGSFL
_Candida__oregonensis	ELLTWKNPAATGKVFGSIVAVLLLVK-INIVNYVFYVAYLALLVSAAAEYVGKLVVQGFVTKLPALVESHVQSIVYAQDITLKAAGASYILYKLTSFFSLYTLLFATVVLAFSFPPLYQANKKEIDAAVGPVGEFI
_Candida__heveicola	PLLTWKNPVATGKVFGGLVLGLVLLK-VNVINYFFYVAYLGLLVSAAAEYVGKLITQGFVTKLPAVFESQIQRIVYAQDITLKAAGVSYILYFVTSYLSLYTISIISVVLAFTLPYLYIQNKDEIDAAVGPVGDFI
_Candida__duobushaemulonis	SLLTWKDPVATGKVFGGLLLGLVLLK-VNFINYIFYAAYLGLLVSAAAEYVGKLVTQGFVTKLPAIAESKVQRIVYAQDITLKAAGVSYVLYFVTSYLSLYTLAIVSVVLAFTVPFLYVQNKDEIDAAVGPVGDFI
_Candida__haemulonis	ALLTWKDPVATGKVFGGIVLGLVLLK-VNVINYVFYVAYLSLLVSAAAEYIGKLVTQGFVTKLPAVFESKIQRIIYAQDITLKAAGVSYILYTVTSFFSLYTLAIVSVVLAFSLPYLYVQNKDEIDAAVGPVGDFI
Candida_auris	YVLTWQDPVLTGKIFGGIVAGLILLK-VNVISHVFYLLYLGLLVSAAAEYVGKLVTQGFVTKLPAVFEAKVQRIVYAQDITLKAAGVSYILYFITSFLSLYTLAFVGVLVAFSWPPFYIRNKDQIDALVGPVGDFI
Wickerhamia_fluorescens	SLLTWKNPAKTAKVFGSIVLVLVVFKTVNLVNLLFRLTFAGLLVAGSLEYVGRFFTQGLVTKLPAVAESLLLNIVFARDVTLKAAGVSYVFYKLTSWFSVFTLIFSFVVLAFTGPFIYTNNKKEIDAAVGPLGSFV
Lodderomyces_elongisporus	YLLTWKDPIHTGKVFGSIVFGLLIFKSVNLVNIFFHASYVALLLSAAAEYVGKLLTKGFVTNLPTIIEKQVKKVVFAQDITLKAAGISYILYKVTSFVSLYSLFIFAIGFFFTVPYIYTTYQKEIDAAVGPIGQAI
Brettanomyces_custersianus	DYLSWKDPVASGKAFGSILATLLIFKYTDVIGWFFYLSAIVLSLSALAEYVGRWATEGFVTKAQILAEIQLQDILTSVDITLKVALASYVLYKLTSFLSVWTILFTSTILAFTLPAIYTANKKLIDSTVAPITKFI
Brettanomyces_bruxellensis	DLLSWKDPIASAKVSGSILGGLLVYRYTDFVSWFFYLSAIVLAASALAEYVGKVATEGFISRAQIFAEIKLQNLFTSVNITLKAAIVSYVLYQLTSFLSVWTIAFTTTVLAFTLPAAYASNKELVDSSVAPLTDFI
Brettanomyces_anomalus	DLLSWKDPVASAKVAGGIIGGLLVYRYTDFVSWFFYISAYVLAVSALAEYIGKVATEGFVSRAQIFCEIQLQNLFTSVSITLKAAIVSYVLYQLTSFLSVWTIAFTSTILAFTLPVAYASNKEVVDSSVAPLTDFV
Brettanomyces_naardenensis	TLLTWDDPVESAKVFAGSISLLLLYKYTDFVSWSFYVTTLVLAFAALAEYSGRLLTEGFVTRAQILLEIELQNLFTAVNITLKAALASYFLYLLTSLFSVWTILFVSTVLSFTVPPVYVSNKEVIDSSVAPVTDWV
Martiniozyma_abiesophila	DLLSWKNPKKSAFALSSILTILFLGKYANIPSLIAYISTFALLGAAIAEYTGKLLTTGFITKAPHVLELKSINLYTAVDISVKAGIIAYIVYLVTKFLSLWTIAFYGTTLAFTIPFIYLSNKEVIDAQAAPVIKLV
Pichia_occidentalis	NLLTWSDPKKSGMALGSILSALLLVKYVNLLSLFFYLSTFALIASGLAEYSGRLVTTGFVTKAPHVIELKIQSLYTSVDVTLRASVLSFVLYKLTSSISLWTLLFVSTLLAFTVPPVYLANKELVDEHVEPVFKLI
Pichia_norvegensis	NLLTWTDPRKSAITLGSILSALLLIKYVNLTALFFRLSTFVLLGSALAEYAGKVVTTGFVTQAPHAVELQAQQLYTATSVTVRAGFASFFLYKLTSAFSLWTIAFVSTILAFTAPPIYLANKELIDANVDPLFKMI
Pichia_membranifaciens	SLLTWKDPKKSAITLGSILTFLVLIKWVNLVALFFRLSTFILLISGVAEYVGKFLTTGFVTKAPHVIELQTQSLYTAVDVTLRTGVLAFFLYKLTSAFSLWTLAFTSAVLAFTVPPVYLSNKEVIDKNIAPAWKLI
Pichia_exigua	SLLLWKDVKKSGIAFSSILSVLLLIKFVNLISLFFRISTFALLVSALAEYTGKTVTTGFITKAPHFLELKTYELFTSVSISIKAGVASFFLYKLTNHFSLWTLTFVLTILSFSVPKVYILNQKVIDENIDPVWKIV
Pichia_kudriavzevii	KLLTWANPKKSATVLGSILGGLLFVKYVNVVSLFFYFSTFALIISALAEYAGRVVTTGFVTKAPHVVEAEGKKLITSTDITFRAGVATFLLYKITSIFSLWSLLFASSIISFTAPPVYLANKEVIDANVGPIFKFI
Pichia_nakasei	GLLTWENPKKSAIVLSSILSSLLLIKYVNLLSLFFRLSTFALLFSGLAEYVGKSVTTGFITRAPHAVEITIQDLYTSVNITLRAGVASFFLYKITSVFSIWTLSFISTIIAFTAPKIYLLNKTVIDKAITPIVKLI
Pichia_heedii	GLLTWTNPKKSAIALTSILTLLILIKNVNLVSLFFRLSTFILLFSGLAEYIGKSITTGFITKAPHFIEINAKNLYTSVNISLKAGIISFVLYKLTIFFSIWSLIFNGTIIAFTAPKIYLLNKKAIDKLISPITKLI
Candida_sorboxylosa	SVLTWSNPQKSGIIFGSIISGLLLVKYVNLVSVLFYVSSIVLLAGSIVEFLSKSILKGLVTQAPHFVELKVQNLLTCVDVSIEAGFATAFLYKLTSIFSLWTIAFYSTIVAFAAPKIYLSNKTVIDANVEPVIKMI
Pichia_terricola	CLLTWEDPKKSSIVLASIISSLLLIKYVNIPALLFRVSTYALLLSVVAEYSTKTLTRGLVTSSVHFFEIKTQKLFASANTTLKAVFASYFLYKLTSLFSLWTIAFYLTIISFTAPKVYQLNKELIDANVSPVIKLV
Saturnispora_hagleri	SLIQWKDPKKSLIAFGSIISTLLLFKFVNVVSLIFYIGTLVFSIGSILEYGSKLVLKSIIANAPHFLETKLLDLVLIKNASFKAAIVSYVIYLITKWVSLWNLLFVGTLLAFSLPPLYTANKKVIDANVSPLFKLV
Saturnispora_serradocipensis	SLIQWKDPKKSLIAFGSIISTLLLFKFVNVVSLIFYIGTLVFSIGSILEYGSKLIIKSIIANAPHFLETKLLDLVLIKNASFKAAIVSFVIYLITKWVSLWNLLFVGTLLAFSLPPLYTANKKAIDANVSPLFKLV
Saturnispora_dispora	SLIQWKDPKKSLIAFGSIISTLLLFKFVNVVSLVFYVGTLVFSIGSILEYGSKLILKSIIANAPHFLESKLLDLVLIKNASFKAAIISYVIYLITKWVALWNLLFVGTLLVFSLPPLYTANKKVIDANVSPLFKLV
Saturnispora_zaruensis	WLIQWKDPKKSSIAFGSIISTLLLFKYFNVVSIVFYISTLVLSIGSIIEYGSKFILKSIIANAPHFLETKLLDLVLIKNASFKAAITSYVLYLITKWVSLWNLLFVSTILAFSLPPLYTANKKVIDANVSPLFKLL
Saturnispora_saitoi	ALVQWTDPKKSGIAFGSIITSLLLLKYINVTALIFYIGTLALALGSIVEYGSKFITKSIISSAPHFLETKFLDLIAIKDVSFKAAIGSYILYLITSWFSLWTLAFYGTILSFALPPLYLSNKKVIDSNLSPLLNLV
Saturnispora_mendoncae	NLIKWDNPKNSAIAFGSGLSALLLVKYVNIPSLIFYSLTLGLLLGSAVEFVGEKILQSLIGVAPHFLEGKFIDLLTIKDISFKAGVVSYVFYLLFKLVSLWNLSLIALILAFGIPPVYQSNKKLIDSNVSPVIKMV
Kregervanrija_delftensis	QLLTWKDPKLSGKVFGSILGFLLLIKFVNIISLFFRILSILLLGSGIAEYSTKTLTSGIISKSNHVLELQFQDILASVNITIKSGIVAFFLYKLTNCLSIWTISFFTTIFSFTLPKLYLSNKECFDKTYSPVIRLI
Kregervanrija_fluxuum	QLLTWKDPKLSAKVFGSILSFLLLIKSVNVISLFFRVLSILLLGSGIAEYSTKALTSGIVSKSNHVLELQFQDILASVNVTIKSGILAFFLYKLTNCLSIWTISFFTTIFSFTLPKLYLSNKECFDKTYSPVIKLI
Saccharomycopsis_fodiens	ALLTWKNPAETGKVFGAALAVLLALKHLNLLNLFFRVSFIGLFVAAAAEYAGKVVTSGFASRSPKLLEQKLVSIAYASDVTLKAAGLSYVLYKVTSWASLYTIALTSLVLAFTVPAVYATYREDIHAITAPFTSKV
Saccharomycopsis_capsularis	DLLTWKNPQETAKVFGVSLATLLALKYVNLVHLFFRFAFISLFVAGAGEYAGKLLTQGFASRLPRLLEAELQSVFFATDITLKVAALSYVFYKITSWVSFFSLLVISLVAAFTVPAAYVSYEKEIHEAKSPYTQKA

Saccharomycopsis_malanga	GLLTWKDPQRTAKVFGVSLASLLALRYVNLVNLFFRFAFISLFVAGAGEYAGKLVTSGFASRLPKVLEAQFQAVFFAADITLKFAALSYVLYKITAWFSVFSLLVSSLVLAFTVPVAYVTYEKEIHQAKSPYTSKV
Saccharomycopsis_fermentans	GLLTWKDPKQSAKVFGVSLAVLIALKYLNIVNLFFKLSSFVLAVSGVAEYLGRLVTTGFASKIPKYLEAHLLDIVYSKNITLKLSGLSFVLYKITSWFSLFQLLFVGLVSAFTLPVVYVTYEKEIKEYQSPYTAHI
Ascoidea_asiatica	SILKWDNPQKTGKIFASIILSLLSFKYLNLLNIFFHFSFIALFISAAAEYLGKLITNGFVSHLPHILELNIQNLFYSNDVTLKAAGLSYLLYKITSWFSLYTLVFSCICFTFTVPAIYSKFQTEIDNGVSPITNFY
Ascoidea_rubescens	KILKWENPQKTGKIFGLTIFSLLAVKYLNLLNIFFHFAFISLFVSAAAEYLGKLITTGFVTHLPHVIESESQKLFYSADVTLKAAGVSYLLYKITSWFSLYTLVFTSVIGAFSVPAIYSRYQNEIDQGIAPLTGFL
Saturnispora_silvae	SLLSWTNPRASAQGLGAILASLIALKYLDVSSLLLWGLAAWLVVASGAQFASQVVGPSVRKLAPHVYEQKALSLILVDDVSVKAGLAFYVAYLVVSTLGLWNVFFFGTIVAFAFQPVYQSNKKVIDESVLPFWKTL
Saccharomycodes_ludwigii	DLLLWKNPVETGKVFGGLLLLLIFIKKINFITGTLTIFYYVTFITSVVEIVTKKFMQGLITRIDRFYQAQFRELIFANKPSLKASVVAYVSCKILSLFTLWTFSLLAVFGTFTLPLVYAKNQEVIDEHVTPLGKYL
Hanseniaspora_vineae	SILKWENPVETGKIFGAILLSLIFIKKIQLIHV-LKFFYSVIFTFSIVEYASKTLLKGLITKIDQFWQYNARQILFASKPSLKIASLVYIFTKILKVISLYSMIVTLVFLSFSLPLVYSKNQTAIDAQITPLGKYF
Hanseniaspora_osmophila	SILKWENPVETGKIFGAILLFLILIKKIQLIHV-LKFFYSVVFTFSIVEYGSKTLLKGLITKVDQFWQYNLRQITFASKPSLKVASLVYIFTKVLKIISLYSMIVTLVFLSFSLPLIYSKNQAAIDAQITPLGKYF
Hanseniaspora_opuntiae	DILLWKNPVQSGKFLGAALLVIIIIKNLKFTHI-LSLIYKAAFTFALVNIISAKVVYQAITKIDNFYQLKFRTFLFAENPTIKVASALFIIVKLLNALSFTTILLIGVLAAFSLPLAYTKNQKVVDAHATPAKKSE
Hanseniaspora_guilliermondii	DILLWKNPVQSGKFLGAALLVIIIIKNLKFTHI-LSLVYKVAFTFALINIISLKVVYQAITKIDNFYQFKFRTFLFAEDPTIKISSALFIIVKLLKALSFTSILLIGVLAAFSLPLAYNKNQKVVDEQVTPAKKSD
Hanseniaspora_uvarum	DILLWKNPVHTGKILGAALLAIIIIKNLKFTHI-LSLTYKLAFTFALINLVSSKVVYQAITKIDNFYQYKFRTFLFAENPTIKVSSVLYIVVKLLNTLSFTTILLIGVLGAFSLPLAYNKNQKVVDEQVAPAKKSD
_Kloeckera__hatyaiensis	DILLWKDPVQSGKILGGALLFIIVINYLKFTHI-LSLIYKVALTFALVNLVSSKVVYQVITKIDNFYQAHFKTFLFAENPTAKVASILYIAVKLLNTLSFSTILLIAVLGAFTLPLAYQKNQKVVDEQVQAPKKTE
Hanseniaspora_singularis	DILLWKNPVESGKVLGAALLAIVLIKSLKFTHI-LSLIYKISFTFAVLNIISDKVVYQVITKIDNFYQKQFRTFLFAENPSIKIVSILFAVTKLLNLLSFTTILLIVVLAAFSLPLAYHKNQAVVDEQVTTAKKTD
Kluyveromyces_dobzhanskii	DILYWKNPVETGKVLGGALVALLVVKKVDLISLFLKLLYTTLFATASVEFLSKLVLKGLVTTIDEAQQSKVRKLVFASSPSFKAAGVVYIAHKLISFMSVWTIAFVSTLATFTLPIVYKTYQREIDAAVGPVSGFV
Kluyveromyces_lactis	DILYWKNPVETGKVLGGALVALLVIKKVNLISLFLKLLYTVLFATASVEFLSKLILKGLVTTIDEAYQAKFRKLVFASSPSFKAAGVVYIAHKLISFLSLWTIALVSTLATFSVPILYKTYQKEIDAAVGPVSGFV
Kluyveromyces_marxianus	DILLWKNPVETGKIFGGALIALLIVKKVDLVSLFLKLLYTVLFASASVEFLSKLVLKGLVTTVDEAYQAKFRQLLFASSPSFKAAGVVYASQKLISLMSLWTIALIATIGTFTVPIIYQTYQKEIDQAVAPVTGFV
Kluyveromyces_wickerhamii	DLLLWKNPIETGKVLGGILVALLVIKKVDLLSFVLKLLYTALFASASVEFLSKLILKGLITTVDEAYQAKFRKLLFAFSPTFKAAGVLYAFSTLISFISLWTLALISTLATFTLPVVYTTYQKEIDAAVSPVTGFV
Kluyveromyces_aestuarii	DLLLWKNPIRTGKVLGGILVALLVVKKVNLLTLLLRFIYTAMFTTASIEFVTKVALQGLVTKIDTAYQSKMRKLMFAYSPSYKAAGVVYIAHKLISTFSLWTIAFVSTIATFTLPVVYKTYQKEIDAAVGPVGGFV
Kluyveromyces_nonfermentans	DLLLWKNPIRTGKVLGGILVGLLIVKKVNLLSLVLRVVYTVMFATASIEFVTKVVSQGLVTKIDVFYQSKFKKLVFAASPSYKAAGVAYIAHKLICAFSVWTLALVATISTFTVPVVYKTYEKEINAAVGPVGDML
Lachancea_kluyveri	DLLLWRNPVETGKVFGGLLVSLLVLKKVNLITFFLKFLYMVFLTTGSIEFASKLFLQGLVTKIDQAHQAKLRKLVFAASPTLKAAGVTYVAHKLFSVLSLWTFLFVSVIFTFTLPLIYKTYQKEIDATAGPVSGFV
Lachancea_cidri	DLLLWKNPIETGKVFGGLLVGLLIVKKVNLITFALRVIYTVMLTTASVEFLSKLFLQGLVTKIDEFYQSKIRKLVMASSPTFKAAGVFYVLHKFFGIFSLWTVLFVGTIGTFTFPFVYQTYQTEIDATVGPVSKFV
Lachancea_fermentati	DLLLWRNPIESGKVLGGILVGLLIIKKVNLITFVLRVIYTVMLTTASVEFLSKLFLQGLVTKIDECLQTKARKLALASSPTFKAAGVFYVLHKFFGFFSLWTILFVGTIGAFTLPFVYQTYQKEIDATVGPVSKFV
Lachancea_quebecensis	ELLYWRNPVETGKVFGGVLVALLVLKKVNLITCVLKVMYTTMLTTASVEFLSKLFLQGMVSKIEAARQAQLRKLLMAQSPTFKAAGVLYVLHKLFSVVSLWTALFVGTILTFSLPLIYKSYQKEIDATVGPVSGFV
Lachancea_waltii	DLLYWRNPVETGKVFGGALVTLLVLKKVNLVTFVLKVLYTVMLSTASVEFLSKLFLQGMVSRVDEMRQEQLRKVLMAQSPTFKAAGVLYVLHKLFSVLSLWTTLFVGTILIFSLPLVYKTFQKEIDSTVGPVTGFV
Lachancea_nothofagi	ELLYWRNPVETGKVFGGALVALLVLKKVNLITFMLRAFYTAMLTTASVEFLSKLFLQGLVTRIDAFYQAEFRRVVMAQSPTFKAAGVFYVLHMVFSIMSLWTALFVGTIATFTLPVVYKTFQKEIDATVGPVSGFI
Lachancea_meyersii	--MYWRNPVETGKVFGGALVALLVLKKVNLITFVLRILYTTMFATASVEFLSKLFLQGLVTRIDTAAMAKFRTTLMAQSPTFKASGVLYVLCKLFSVMSLWTTLLVGTIAAFTLPVVYKTFQKEIDATVGPVSGFI
Lachancea_lanzarotensis	DLLYWKNPVETGKVFGGALVALLVLKKVNLVSFLLRVLYTSMFTTATVEFLSNLFLQGLVTRIDAFTMAKFRTTLMAQSPTYKASGALYVLSKLFSVLSVWSILFIGTIGVFTVPVVYKTFQKEIDATVGPVSGFI
Lachancea_dasiensis	ELLYWKNPVETGKVFGGALVALLVLKKVNLLTFLLRTLYTVLLTTASIEFLTNLFLQGLVSRVDAFYQTKLRKLAMAQSPTFKAAGVLYVLHKVFSVLSLWSTLFIGTVALFTAPVIYKTFQKEIDATVGPVSGFI
Eremothecium_coryli	DLLLWKNPIETGKVFGGLLVALLILKKVNLITFFLRVLYTTLFASSTIEFVSKLVLQGLVTKIDEMYQAKIRTLLFAASPSFKAAGVLYILHKLMSSISLWTLTFLGVIASFSLPLVYHIYQKEIDAAVGPVSHYL
Eremothecium_sinecaudum	DLLLWKNPLETGKVFGGLVVALLILKKVNLVTFFLRVLYTTLFASSTIEFVSKVVLQGLVTKMEEFYQSQMRQLLFAASPSFKAAGVLYILHKLISTVRLWPLLMIGVVSTFSLPLVYKTYQREIDSAVGPISQYL
Ashbya_aceri	DLLLWRNPVETGKVFGGLLVGLLVLKKVNLVTFFLRVLYTTLFLSSAVEFASKFVLQGLVTKLEAAYQTQVRRLVFAASPTFKAAGLLYVLHILVSFFSVWTLGLLSVVGAFTVPVVYHKYQREIDDAVGPVSHYL
Eremothecium_gossypii	DLLLWRNPVETGKVFGGLLVALLVLKKVNLVTFFLRVLYTTLFLSSAVEFASKFVLQGLVTKLEAAYQTQVRRLVFAASPTFKAAGLLYVLHILVSFFSVWTLALLSVVGAFTLPVVYHKYQREIDDAVGPVSHYF
Eremothecium_cymbalariae	DLLLWKNPVQTGKVFGGLLFLLLVLKKVNLITFFLKVGYSILFVNSTVEFVSKLVLQGLTTKIEEFYQAHVRRLVFAASPSFKAAGVLYVLHKLVSVLSLWTLVFIGVIATFTLPIVYKTYQKEIDGTVGPVSQYL
Candida_bracarensis	DLLLWRNPIDTGKYFGTSLLVLLILKKVNLITFFLRVGYTILLSTATLEFATKLFLQGLVTKIDDAKQAIMRQTVFAYSPTFRAAVVLFLLHRFFSWFSVWTILFVGDIVLFTFPLIYKTYQTEIDAVVGPLNKFI
Nakaseomyces_delphensis	DFLLWKNPVQTGKCFGSSLLVLLILKKVNLITFFLRVGYTILFSTALLEFVSKLFLQGLVTKIDDLKQAVMRQTVFAYSPTFRAAVVLFFLHRFFSWFSVWTILFVGDIVLFTFPLIYKTYQKEIDAVVGPLNKFV
Candida_nivariensis	DFLLWKNPVQTGKCFGTSLLVLLILKKVNLITFFLRVGYTVLFSTAALEFVSKLFLQGLVTKIDDLKQAVMRQTVFAYSPTFRAAVVLFFLHKFFSWFSVWTILFVGDIVLFTFPLIYKTYQQEIDAVVGPLNKFV
_Candida__glabrata	DLLLWRNPLETGKVFGTTLVALLILKKVNLLTFFLRVGYTILLSTALLEFSTKLVLQGLVTKIDNAKQAKMRQLVFANSPTFKAAVVLYLLHKFFSWFSVWTILFVGDIALFTLPLVYKTYQKEIDAVVGPISKMV
Naumovozyma_castellii	DILLWKNPIETGKIFGGALVALLILKKVNLITFFLRVFYTIFLTTGSIEFLSKLFLQGLITKIDCFKQAKMRKLVFAYSPTFHAAFTFYLLHKLFSWFSVWTLLFVGVIAAFTSPLTYCTYQKEIDAVVGPLCKFI
Naumovozyma_dairenensis_A	DILLWRNPIESGKLFGGLLVSLLVLKKVNLITFFLRAAYTILLTTGSIEFISKLFLQGLITKIDEIKQAKMRKLVFAYSPTFKAAIVLFLLHKLFSWFSVWTLLFVGVISAFTLPITYKIYQKEIDTILAPLCNLI
Kazachstania_rosinii	DLLLWKNPVETGKYFFGALLALFILKKVNLITFFLRVAYTVLLTTGSIEFVTKLFLQGIVTKVDELKQAKMRMLVFAYVPNFKAAFILFLLHRFFSWFSVWSTIFAGVIATFTLPIVYKTYQKEIDAFIGPLAKLV
Kazachstania_saulgeensis	DILLWKNPIETGKYFFGSIIALLILKKVNLLTFLLRVLYTVFLTTGTLEFATKLILQGIVTKVDEIQQAKMRMLVFAYVPTFKAAGIAFALHRFFSWFSVWTILFVADIMAFTVPIVYHTYQKEIDAQLGPLAKLC
Kazachstania_turicensis	DILLWKNPVETGKYFFGSIIALLILKKVNLITFLLRVFYTIFLTTGTLEFLTKIILQGIVTKIDEFKQAKMRMLVFAYVPTFKAAGIAYCLHKFFSWFSVWTILFVADLLAFTVPIVYQTYQKEIDAQLG-LAKYL
Kazachstania_kunashirensis	DILLWKKPVETGKYFFGSILVLLILKKVNLITFILRVLYTVFLTTGSIEFISKLVLQGLITKIDEIRQAKMRMLVFAYVPTFKAALIAYCLHKFFSWFSVWTILFTADIIIFTFPIIYKTYQKEIDTNIGPLSKLF
Kazachstania_martiniae	DLLLWKNPIETGKIFFGSLIALLILKKVNLITFFLRVGYTILLTTGTIEFVSKAFLQGLVTKIDEFKQAKVRMLVFASVPTFKAAVTLYLLHKLFSIFSLWTLLVLVDLAVFTLPIVYKTYQKEIDAAVGPLKSLV
Kazachstania_intestinalis	DLLLWRNPIRTGKVFFGALLALVILKKVNLITFFLRVAYTILLTTGTIEFASKAFLQGLVTKVDEFKQAKVRMIVMANVPTFKAALVLYLAHKFFSIFSLWTVLFLFTIGTFTCPIIYKTYQKEIDACLGPVSKLF
Kazachstania_taianensis	DLLLWKNPVETGKVFFGSLVALLILKKVNLITFFLRVFYTVFLTTGTVEFVSKAFLQGLVTKIDEFKQAKMRKLVFAAVPTFKAALCAYLLHKLFSWFSVWCIVFVGDLAVFTLPIVYKTYQKEIDQVLAPLSKLM
Kazachstania_siamensis	DLLLWKNPIQTGKIFFGSLLALLILKKVNLITFFLRVFYTIFITTGTIEFLSKAFLQGLVTKLDEFKQAKMRKLVFAQVPTFKAALITYLLHKLFSWFSLWTLLFVGDLFIFSLPIVYVTYKTEIDSFLGPLNTLL
Kazachstania_aerobia	DLLLWKNPVQTGKVFFGAILALLILKKVNLITFFLRVFYTILLTTGSIEFLSKAFLQGLVTKLDDFKQAKMRMLIMAQVPTFKAAFVTYLLHKLFSWFSVWTLLFVGVLATFTLPVVYTTYQKEIDQTVGPLNALV
Kazachstania_solicola	DLLLWKNPVKTGKVFFGAILALLVLKKVNLITFFLRVFYTIFLTTGTIEFLSKAFLQGLVTKFDAFKQAKMRMLVMAQVPTFKAAFVTYLLHKLFSWFSVWTLLFVGVLATFTLPVVYTTYQKEIDETVGPLNAIV
Kazachstania_unispora	DLLLWKNPIETGKYFFGSILTLLILKKVNLITFFLKLFYTIFLTTGTIEFLSKAFLQGLITKFDEFKQAKFRMLVFAQVPTFKAALIAFALHKLFSWFSVWTILFVSVLAMFTLPIVYVTYQKEIDSMLGPLNTLL
Kazachstania_spencerorum	DLLLWKNPIETAKYFIGSLTILLILKKINLITFFLRIFYTIFLTTGSIEFISKVFLQGLVTKIDMLKQAKMRMLVFAYVPTFKAAGIAFVLHKLFSFLSLWTILFISDIAMFTLPIFYITYQKQIDQSVG-VSKYI
Saccharomyces_cerevisiae_YDR233C_Rtn1	DLLLWRNPVQTGKYFGGSLLALLILKKVNLITFFLKVAYTILFTTGSIEFVSKLFLQGLITKIDEAFQAHIRKTVFAQVPTFKTAVALFLLHKFFSWFSIWTIVFVADIFTFTLPVIYHSYKHEIDATVGPISNLV
Saccharomyces_pastorianus_1	DLLLWRNPVQTGKYFGGSLLALLILKKVNLITFFLKVAYTILFTTGSIEFVSKLFLQGLITKIDEAFQAHIRKTVFAQVPTFKTAVALFLLHKFFSWFSIWTIVFVGDIFTFTLPVIYHSYKHEIDATVGPISNLV
Saccharomyces_paradoxus_1	DLLLWRNPVQTGKYFGGSLLALLILKKVNLITFFLKVAYTILFTTGSIEFVSKLFLQGLITKIDEAFQAHIRKTVFAQVPTFKTAVALFLLHKFFSWFSIWTIVFVGDIFTFTLPVIYHSYKHEIDATVGPISNLV
Saccharomyces_kudriavzevii_1	DLLLWRNPVQTGKYFGGSLLALLILKKVNLITFFLKVAYTILFTTGSIEFVSKLFLQGLITKIDEAFQAHIRKTVFAQVPTFKTAVALFLLHKFFSWFSIWTIVFVGDIFTFTLPVIYHSYKHEIDATVGPISNLV
Saccharomyces_mikatae_1	DLLLWRNPIQTGKYFGGSLLALLILKKVNLITFFLKVAYTILFTTGSIEFVSKLFLQGLITKIDEAFQAHIRKTVFAQVPTFKTAVALFLLHKFFSWFSIWTIVFVGDIFTFTLPVIYHSYKHEIDATVGPISNLV
Saccharomyces_arboricola_1	DLLLWRNPVQTGKYFGGSILALIILKKVNLITFFLKVAYTILFTTGSIEFVSKLFLQGLITKIDEAFQAHVRKTVFAQVPTFKTAVALFLLHKFFSWFSIWTIAFVGDIFIFTLPVIYHSYKHEIDATVGPISNLV
Saccharomyces_eubayanus_1	DLLLWRNPIQTGKYFGGSLLALLILKKVNLITFFLKVAYTVLFTTGSIEFVSKLFLQGLITKIDEAFQAHIRKTVFAQVPTFKSAVALFLLHKFFSWFSIWTIMFVGDIFVFTLPVIYHSYKHEIDATVGPISKLV
Saccharomyces_uvarum_1	DLLLWRNPVQTGKYFGGSLLALLILKKVNLITFFLRVAYTVLFTTGSIEFVSKLFLQGLITKIDEAFQAHIRKAVFAQVPTFKAAVALFLLHKFFSWFSIWTIMFVGDIFIFTLPVIYHSYKHQIDSTVGPISKLV
Kazachstania_transvaalensis	DLVLWKNPIETGKIFFGSIIALLVLKKVNLLTFFTRIIYTVLLTTGSIEFVSKVFLQGLVTKIDSFKQAKFRMLVFAQVPTFKAAFVFYLLNKLFSWFSVWTIIFGIDVALFTLPIFYKTYQKEIDSYVKPLEKYL
Torulaspora_maleeae	GLLLWKNPIETGKVFGGLLVGLLILKKVNLVTFFLKVLYTVVFITGSVEFVSKILLQGLITSIDEVQQAKFRKLVLAYQPNFKAAVVLYLLHKFFSWFSVWTIALVSVIGAFTVPLVYSIFQTEIDTAVGPVSKFV
Torulaspora_pretoriensis	NLLLWKNPVETGKVFGGLLVGLLVLKTVNLATFFLKVLYTVLFLTGSVEFVSKILLQGLVTSIDSLQLAKFRKLVFAYQPNFKAVAGLYVLHKFFSWFSLWTIAFVGVIGAFTVPLIYSIFQTEIDTAVGPVSKFV
Torulaspora_franciscae	KLLLWKNPVETGKVFGGLLVGLLVLKTVNLATFFLKVLYTVLFVTGSVEFVSKILLRGLVTSIDSLQISKFRKLVFAYQPNFRAAAGLYVLHKFFSWFSLWTIAFVGVIGAFTVPLVYSIFQTEIDTAVGPVSKFV
Torulaspora_delbrueckii	NLLLWRNPVETGKVFGGLLVGLLVLKTVNLATLFLKLLYTVVFITGSVEFVSKILLQGLISSVDYLQQAKLRKLVFACQPNFRAAAVLYVLHKFFSWFSLWTITFVGVIGAFTVPLVYSLFQTEIDTAVGPVSNFV
Torulaspora_microellipsoides	GLLLWRNPIETGKVFGGLLVGLLVLKTVNLFTFFLKILYTVVFITGSIEFVTKIILQGLVTKIDNLQQAKLRKLVFAYQPNFKAAAGFYVLHMLFSWFSVWTIALAGVISTFTLPLIYSLYQTEIDTAVGPVSKFV
Zygotorulaspora_mrakii	GLLLWKNPIETGKVFGGLLVGLLVLKTVNLITFFLKVFYTTLFISGSIEFVSKIILKGLVTKVDHMYQAKFRKLVFAYQPNFKAAVILFVLHRFFSWFSVWTIAFIGVISTFTVPLIYSMYKKEIDTAVGPVSGFV

```
Zygotorulaspora_florentina                 DLLLWKNPVETGKVFGGLLVSLLILKTVNLATFFLKIFYTVLFVTGSVEFLSKLVLQGLVTRIDHLYQAKVRKLVFAYQPNFKAAATLFVLHRCFSWFSVWTISLMSVISVFSLPLVYSIYKKEIDTAVGPVSSFV
Zygosaccharomyces_kombuchaensis            DLLLWKNPIETGKVFGLSLVVLLILKKVNLITFFLRVLYTVVFTTGAIEFVSKFVLQGLVSKVDSLFQSKLRELVFAYEPNLKAGVTFYFLHKFFSWFSVWTILFLGVISSFTLPLIYHTHQQEIDEAVGPVSKFV
Zygosaccharomyces_bisporus                 DLLLWKNPIETGKVFGLSLVVLLILKKVNLITFFLRVLYTVVLTTGAIEFASKFVLQGLVSKIESLVQTKLRQLVFAYQPNLQAGLTFYFLHKFFSWFSVWTILFLGVISAFTLPLVYHTHQAEIDSAVGPVSKFV
Zygosaccharomyces_bailii                   DLLLWRNPIETGKIFGLSLVVLLILKKVNLITFFLRVLYTVVLTTGAIEFVSKFVLQGLVSKIDSLIQTKLRQLVFAYEPNLQAGIAFYFLHKFFSWFSVWTIVFLGVISAFTLPLVYHTHQAEIDSAVGPVSKFV
Zygosaccharomyces_rouxii                   DLLLWKNPIETGKYFGLSLLVLLILKKVNLITFFLRVLYTVIFTTGAIEFASKVVIQGIVSKIEDVYQAKLRTLVFAEEPNLKAGVVLYFLHKFFSWFSVWTIVFLGVISAFTLPLVYYTHQEEIDSAVGPVSKFV
Tetrapisispora_blattae                     DLLLWKNPINSGKILGGALVSLLVLKKVNIITFFLRLFYTVFFISGSVEFLTKLFLQGLVTKIDALKQAKMRGLVFAHSPTFKAAGVVWVLHKLFSVLSIWTVLFISVIAAFTLPLIYHIYQVEIDTAV-------
Nakaseomyces_bacillisporus_A               DLLLWKNPIYTGKIFFSILTGLIILKRVNLITFLLKVLYIVIFTTGSIEFLTKLFLQGLVTKVDEGQQAKMRKLVFAYSPNFKTAVILFCLHKFFLIFSLWTVCFIGAIFTFTLPLIYSIKQKEIDQCLGPLTTYL
Tetrapisispora_fleetii                     DVLTWKNPIETGKLFGASLIVLLFIKKINILTFLTKTLYLVFFTTGTIEFATNLLLNGLVTKIDEFKQAAVRKLVFAASPTLKAGVLFWFLNKVFNLISFTNLLIIVDLLAFSVPIIYKNYQTEIDQIIGPVSKFI
Tetrapisispora_phaffii                     DVLTWKNPIETGKLFGASLLAILFIKNVNILTFLSKTLYLVFFTTGSIEFITNLLLNGLVTKVDEFKQAAMRKLVFAASPTLKAGALFWFLHKVFSLISFTNLLIIVDLIAFTLPIVYKSYQTEIDQVVGPVSKLI
Tetrapisispora_iriomotensis                DLLVWKNPIETGKIFGASLVALLFIKKVNILTFLTKTLYLVLFTTGTIEFVTNFVLNGLVTKVDEFKQAAMRKLVFAHSPTLKAAGCLWFLNKIFNLISFTNLLLVLDLVAFTFPIIYKNYKTEIDQIVGPVSTFI
Candida_orthopsilosis                      ELLTWKNPVYTGKVLGLTLGGLILFRTIDLVRIFFKISYITLLFSAGAEYIGKVFTQGFVSNLPLIIEHHLNYIVYARNVTLKFAGISYIFYQLTSVVSLFTLTIVSVILLFAIPPAYLANKKQVDAFVTPVGPYL
Candida_parapsilosis                       ELLTWKNPAYTGKVLGLTLGGLLLFKAIDLVRIFFKISYITLLFSAGAEYVGKVFTQGFVSNLPLIIEHHLNYIVYSRNVTLKFAGISYIFYQLTSVVSLFTLTVISVILLFTIPPAYLANKKQVDAFVTPVGPYL
Saccharomyces_cerevisiae_YDL204W_Rtn2      GLIYWTNPSKSGASFAATLVSLLILRNVNVISVLLKIGYMVLFTSFAVELSTKVLFKGVVSRIDRELEDRIRKLVFAHRTNFTIGVSLYFLHGLFAIFSMNTVLIMTTIFLYTVPLIYDRKQARIDRAITDEGSYS
Saccharomyces_pastorianus_2                GLIYWTNPSKSGASFAATLVSLLILRNVNVISVLLKIGYMVLFTSFAVELSTKVLFKGVVSRIDRELEDRIRKLVFAHRTNFTIGVSLYFLHGLFAIFSMNTVLIMTTIFLYTVPLIYDRKQARIDRAITDEGSYS
Saccharomyces_paradoxus_2                  GLIYWTNPSKSGASFVATLVSLLIFRNVNVISVLLKIGYMVLFTSFAVELSTKVLFKGVVSRIDRGLEDRIRKLVFAHRTNFTIGVSLYFLHGLFAIFSMNTVLIMTTIFLYTVPLIYDRKQVRIDRAITDENSYS
Saccharomyces_mikatae_2                    GLIYWTNSKKSGASFVATLVGLLIFRNVNVISVLLKIGYMVLFTSFALELSTKVLFQGVVSRIDRALEDKVRKLVFAHRTNFTIGVSLYFLHGLFAIFSMNTVLIMTTIFLYTVPLIYDRKQTRIDHAITDNGSYS
Saccharomyces_arboricola_2                 GLIYWSNPRKSGATFVATLVSLLIFRNVNVISVLLKIGYMILFTSFAVELSTKVLFRGIVSRIDRALEDRIRKLVFAHRTNVTIGISLYFLHGLFAIFSMNTVLIMTTIFLYTMPLMYDRKQTRIDHAITDEDSYS
Saccharomyces_kudriavzevii_2               GLVYWTNPKKSGATFVATLVSLLIFRNVNVFSVLFKIGYMVLFTSFAVELFTKVLFQGIVSRIDRLLEDRIRKLVFAHRTNFTVAVSLYFLHSLFAIFSMNTVLVMTTIFLYTVPLIYDRKQARIDSAITDKDSYS
Saccharomyces_eubayanus_2                  GLIYWTNPRKSGATFAATLVGLIVFRNVNIISVLLKIGYMVLFTSFAVELSTKILFQGIVSRIDRALQDKIRKLVFANRTNLTIGMSLYFLHGLFAIFSMNTVLMMTTMLLYTVPLIYDRKQERIDRAINDQDSYS
Saccharomyces_uvarum_2                     GLIYWTNPRKSGATFAATLVGLIIFRNVNVISVLLKIGYMVLFTSFAVELSTKILFQGIVSRIDRVAEDKIRKLVFAQRTNLTIGVSLYFLHGLFAIFSMNTVLMMTTILLYTVPLIYDRKQARIDRAINDQDSYS
Nakaseomyces_bacillisporus_B               DLLLWKRPFYSLKWFVSTLLVLVILEHVNIVSLFLKGIYTLLFISSLLEFGTKLSLQVILSTMDYVWQTKTRNIILASSPTFKVATSLFFLQKFIEIMPILCGLSTLVVLLFTVPYFYNRYENTIKQKQ-------
Wickerhamiella_cacticola                   PILSWRDPARTGALFGQAIIAWVVLSSSIVFRLFLRLSVYLLLLLAGLEASTRAINNGLIASSATVILYGIEYVLDTRNPNAKLAFGAWILYKVLGIFTLRTFALLVIIITFILPVAYLQFKPQVDQAIRDRGGYS
Candida_galacta                            PLLSWRNPHATGIAAARAVGLWVVLSSTLTLRLFLRLAAWSLVLLSGVEFLTRQLNPGLISQSSCIGVYGIEHLVDARDPGLKAAGISWVFYVLLGLFSLRFLTFLIIVTTFGAPPLYLHFKTQVDALIGTRGGFP
Wickerhamiella_versatilis                  PALSWRNPQETGLLLAESLGIFVVLSSPLTLRLFLRLSFWLVGLFSLIELVSKHLNQGFIAAANALVISGVENVLDAKNPGLKIAAVCRLVYSLLGIFSVKFLVLFSIVLVFGLPPLYLQFKTQVDDAIGNRGGFP
Wickerhamiella_domercqiae                  YILSWKNPQATGILLAETLGIFLVFSSTLSLRLFLRVGFWAVGILSLVELGTRALNNGIVSNAVAVALASIQHVVDARNPGLKLSALCWVGYVLLGFLSLKTLVLVSILSAFTLPPLYLQFKSHVDGTVGARGGFP
Candida_magnoliae                          YILSWKNPYETGVLLAEVLGLFVMFSSTLTLRWFLRAAFWIVGAASFVEWGSRTLSQGLVTAASYVAIFGIQHVLDARDPGFKLAGLFYVVYVFLGLFKLRTLLLTAIVLSFSLPPLYLQFKTEVDDAIGPRGGFP
Starmerella_bombicola                      SILTWKNPFETGVLLAETLGLFLVFGSTFTLRYFLKFASSVVGLLSVAEWASRSVSKGLVSTASYVLTFGIQHLLDARDPGLKLAGLLYGSYILLWIFSLKALLLSSIIIAFSVPPLYLQFKPEVDEAIGPRGGFD
Candida_apicola                            HILSWKNPYETGSLLAEALGIFIVLSSTFSLRFFLKIFSSVIGLFSIVEWGSRAISKGLILNTNYLVTHSIEGLVDAREPGLQLAALLYATYVLLGLFSLKTLVLLTIVFAFGLPPLYLQFKSQIDELIGSRGGFP
Starmerella_bacillaris                     YILTWKNPYETGVLLAKILVIFAI-GIKMTPRCFFKLFFYSIGSLSALEAISKAVTTGIVSSASHLLTVGFMHLIDARNPGIRLSAAAFVIYILLGIFSVRNLIFTIIISAFSLPPLYIANKEIIDQGIGPRGGFP
Wickerhamiella_sorbophila                  AILSWKTPRETGLLFVQLLLVWVVFSSTLTLRLFLRLSYWAVGLLSAVEYATRKLNSGLVANAQHVAYGGIEHVVDARNPGFGIAAAFWLTYKLLGAFSLKSLFLIAIIGAFAVPRLYLEFQQPIDNALRDRGGFP
Nadsonia_fulvescens_fulvescens             SILTWSDPVKSGGSFVSAIALLLLLKFGSILPFVLWIAYWFLGVSTFTEYFTRSLYKGLISSVQVLALKEFRRILDVQDPSMVACGLSYILCKAIGLVSLWTLLVSMTTLAFTVPALYLRFEKTIDANFDSVVSTV
Nadsonia_fulvescens_elongata               PILTWSDPVKSGGSFASAIVLLLLLKFGSILPFVLWIAYWLFGVSTFTEYFTRSLYKGLISSVQVLALKEFRRILDVQDPSMVACGLSYILCKAIGLVSLWTLLVLSTILAFTVPALYLKFEKTIDANFDSVVSTV
Nadsonia_starkeyi_henricii                 PLLSWSNPIKSGGVFASAIGFLLVIKFCGFLPLLLWATYWTIGISTFTEYFTKSLYKGLISSVQAVIIKQFKQILDVEDPSMMVAGVSYIIYQIISLISLWSFLILLTIAAFTVPALYLKFEETIDAKVDSIVSII
Naumovozyma_dairenensis_B                  NLVYWKNPKNTLKVFSSILFSLIIFKLFNFFTLSLRVIYSILFISSLAEFITKLTLRGLVTSHSENCQLYMTSIIFAKSPTFAFALSFYILHKIAKLVSIWWCIFISVWSIFTVPFIFSKFQREIDQTINELVQEN
Dipodascus_albidus                         SLLKWEDPGRTGLAFFGGLLILTVLRSPNLIRYFLKTCFTLVGFLLLIELVTRFLSRGLVSASDINSIYWLRRVIDARDASFISLILIVILYILTMIVSLPTLLSIIFVLTFTVPALYFKFRPELHHAYQTLGNVF
Dipodascus_geniculatus                     SLLKWEDPSRTGFAFFGGLIAISLFHSPKFIQYLLRTSFTIIGLTLVTELSTKFLSRGLISSSDIFSIFWFRRVLDCRDTSILSLVVITVLYILTMIASLTSVLSVIYVITFTLPAIYFKFQSELNHAYNTIGNVF
Galactomyces_candidum                      SIFNWEDPIRTATAFGECLVLLAIFQSADFLRLVLRLCYLFIGITALTEIGTKFLNAGLVSSANMFVLYWIRRVVDARDLTGVSVFCLWFTYCLTAVFPVSSILSLLVIGAFTVPAIYTRFRAEIDHARGTVFGAG
Saprochaete_clavata                        PLLLWENPTRTAIVFAEIVTVLLMAQNSEFLRIVLRVIYYSLGLTVGLEVITNFLSGGVISSSHVICSYWFKRVFDARDPTVMTVVGLWILHNISYGVSISQLLTIVVIGAFLIPPIAHRYNHNLSHAYATIASIF
Magnusiomyces_capitatus                    PLLLWENPTRTAIVFAEIMTVLFMAQNSEFLRMILRIIYYSLGVTVALEVVTNFLSGGIVSSSNAICSYWFKRVFDARDPTMMTIGGLWFLHNISYGVSLSQLFTVIVIGAFIIPPIVHRYHHNLNHAYEKFANIF
Magnusiomyces_tetraspermus                 PLLLWENPVRTGLTLFELLLVLLIFQNSNLLRMILRIVYFTLGATLFIEFVTKFLNSGLVSSSSTIIVYWVKRAFDARDMTLTAFFSVLGLYFLSFAVPVSLLLTVSLVGLFILPALYLRCEHHVKAAQGGIRNRF
Magnusiomyces_ingens                       PLLLWENPVRTSVTLAETLAVLLMVQSTDFLRLFLRIIWLSIGGTLLLELITKFLNEGLISSSLIIILYWFKRALDARDTTILAFIFVWGIYLVTFMASISSVLILIVIGAFTLPVLGLRFEPQLARARNTLLSFL
Tortispora_starmeri                        DIILWKEPAVSAGVLATILLGFALSSYIKLTPLILRALFFYLTGAVIVEVATAKFRTGLVTAATCAALAGAQHILFVQNPTGLAALLAFFGYILSRALSLGCLIETAVVSAFIVPPVYVKYEAEIQKGL-------
Tortispora_ganteri                         QVLLWKNPVVTGGILAFVVIGMIISTKLDMPRLIIRLTAFYLCSVAVVEFASAKAKKGLVTSAEKIAVSGIYSIVYADNPTAVVGFSMLLLMGLLRTFSLLAIVRMAILLAFAVPPLYVKYEKQIHQYL-------
Tortispora_caseinolytica                   QVLLWKNPVVTGGILAFIIIGMVISTKLDMPRLLVRLTAFYLCGVAAVEFGSAKAKKGFVTSAEKIAVSGLYSIVYAENPTALLGFSVLLLMGLLRTFSLLTIFRMTILLAFAVPPLYVKYEKQVHHYF-------
Schizosaccharomyces_pombe                  SVLTWKNTSCSFSTLMSILALVYVPSWINLPRLFFRTFRYVFLITSIIEFGGLFASRGVLSHVNSIMLIQFQRILFAESPTFTASVAAFIEFFLSGFLSYKSLFVWNVLFAFILPRLYVCNERSIKHLV-------
Schizosaccharomyces_octosporus             HVLTWKNVSCSFSTLLSILAVFFIPAWINIPRLFFRTIRYVFLITSIIEFGGLFASRGVLSPVNSLLLIQFQRVLFAECPTFAVAIGAFIEYFLSGFLSYRGLCIWNVLFAFTLPKLYEMNETAIKRLV-------
Schizosaccharomyces_cryophilus             RVLTWKNTSCSLSSLLSILALFFVPSWINIPRLFFRTIRYVLLITSIIEFGGQFASRGVLSPVNCTLLIQFQRVLFAECPTFAVAIGSFIEYFLSGFLSYRGLFIWNIIFAFTLPKLYEMNEASIKRMI-------
Schizosaccharomyces_japonicus              DVLLWKNKYCSMSVLAAIFTALYLPCVLSIHRNLFNISSWIFGVSAVVEYVSRIITSGVLGRLDSVMLVELQRLFLAENPSVSAFFLAWFYYRMSLLLSLKTIFALSVIAAFSLPPFYQANKRVIDGTL-------
```

Supplementary Text File 2. Nucleotide sequences determined during the course of this work (cDNA/genomic DNA) have been deposited at GenBank under accession numbers MN689081–MN689090. For the convenience of the reader, below these deposited sequences are listed (in fasta format), ordered by accession number. Coding sequences are in capital letters.

Lipomyces suomiensis

>MN689081[LIPSUO_RTNA_CDNA][Lipomyces suomiensis][strain=CBS 7251] reticulon-like (rtnA) mRNA complete CDS, 785 bp
gccaaagattgttaaaaATGTCTACTAGTGCTGCGCCCGTGAGGATTGATCCTCCGTCTGATAAATACAAGACGCTTATTTCGGATCTTGTAAC
GTGGAAGAATGTACTGAATTCCGCGATTGCTTTTTCGGGATTGCTTGTTTCGCTGATTGCTGTGAAATATATCAATGTTGTTAATTTGCTATTC
AATACGGCGTATCGCGCGCTCGGAACTGTCGTTGTTATCGAATTTGTTGGACGAGCATTGGGAAGATCTCCGGGGTTTGTTTCTTCGATAAAAA
CATTCAAGGGATACTTTACAGTCTCAAAGGCAGTAGTTGATCCCATCTTCGATGAAATCATTGTGTTGGTCAATTTCTTGCTGGTTGAAGCTCA
GAAGCTTGTTTTTGTTGAGAGTGTTCCGGGTACCTTGCTCGCATTCGTAGGATCGTATTTTGCGTATGTAATGGTTAAATTTGTGTCTATCTGG
ACGCTCGTGTTTTTTGGAGTGACTGTGGCATTCACTGCACCGCCAATCTACTTCACTTTCCAGAAGCAGATCGACGCTCAGATAGACACTGCGA
AAAAGACGATCGATGCAAAGACTGAGAAGGCGCGAGGCCAGCTAAAGGAGCAGTACGATAAGGGCGCGAAGGTTGCTGGCGGATACGTGTCGAA
GGGTCTTGATAAGGTTGGATATAAGCGCAATATGCCTCCTGTGCCTGTTGCTGCGAGCACCGAGACTCCTGCTGCTGCTGCTTCTACGTAAttg
cgtagccggctgagaggggcaagtgtctccctc

>MN689082[LIPSUO_RTNA_SPLINTER][Lipomyces suomiensis][strain=CBS 7251] reticulon-like (rtnA) mRNA precursor RNA, partial CDS, 211 bp
TTGTTCTTCGACAATCCTGCTGAATTCTAATATTTCGCGGATCACAAAAAATTTTTATTAGAAGTTAATCATACTTCATTGAATTCATAGGCCA
AAGATTGTTAAAAATGTCTACTAGTGCTGCGCCCGTGAGGATTGATCgtgagtgaatgccattgaatatgatgatttattgattgagaaaatca
tgaaagactgatcgatgtacaaa

Lipomyces lipofer

>MN689083[LIPLIP_RTNA_CDNA][Lipomyces lipofer][strain=CBS 944] reticulon-like (rtnA) mRNA, complete CDS, 895 bp
cctcccttgttcactgaaatatatccgccacatcttgcaagttttctaaacgaatttcttgtttgcaataaatcgatttatctttaattctttt
aactcgtcgacATGTCGCAAGCCGCTACTACCTCTTCGTCGGTGCCTCCACCCCAATTCAATAAGTACAAGTCTTTCTTCACGGACCTCCTCAC
GTGGAAGAATCTCTATGTCTCTGGGGGGTCTTTTGTCGGGGCCATCGTGCTTCTGTATCTCGTTAAATATGTCAATGTTGTGCAGTTTTTCTTT
AATGCTGCGTACATCACCTTGGGGAGTGCCATTGCTCTCGAATTTGCGGGCCGATCAATCAAGGGTGGACCTGGCTTCGTCAGCTCATTTCGTA
GTGAGAAGTACTTCGAAATTTCCAAGGATGTTGTTGATCCTCTTTTCAGCGAGTTCACCGCTCTGCTGAATTTTGTTCTTATCGAGATTCAGCA
AATTGTTTTTGTTGAGAGCGTTCCTCTGACTGTTTTGGCATTTGTTGTGTCTTACTTCACCTATATTTTGGTTCATTATGTTTCCCTCTGGGCT
CTTGCATTCCTTGGTACCATCCTTGCTTTCACTGCTCCTCCAGTTTATCTGCGTTTCCAGACTCAGATCGACGAGCAGGTCGCCAAAGTTAGCA
AAGTCGTTGAGGACAAAGCTGAGGACGCCAAGGCCAAGGCCAATGAGCACTTCGGGAAGGCTATCGGTATCGCGAAGAACTATGTCGACCAGGG
CCTCGACAAGGTCGGATATAAAAGAAACCTTCCCGCAGTGCCTTCCACTGAGTCTGCTAAACCTGCAGAACCTGCTGCAGAACCTGCCACTATT
CCAGTCGCTACCGAGCCAGTGGCTGCGTCGGAGTAAgcggtgtccgtgc

>MN689084[LIPLIP_RTNA_SPLINTER][Lipomyces lipofer][strain=CBS 944] reticulon-like (rtnA) precursor RNA, partial CDS, 273 bp
cctcccttgttcactgaaatatatccgccacatcttgcaagttttctaaacgaatttcttgtttgcaataaatcgatttatctttaattctttt
aactcgtcgacATGTCGCAAGCCGCTACTACCTCTTCGTCGGTGCCTCgtgagttcagattttgttatcttgttttgttcaaggatgatacatg
tgatcttgatcgcgctgcaattgatccgctctccaaacaagtttccatttgcgcgcatctgaacattcatggaataaataatttg

Lipomyces starkeyi

>MN689085[CCY-33-1-1_RTNA_GDNA][Lipomyces starkeyi][strain=CCY 33-1-1] reticulon-like (rtnA) gene, complete CDS, 1311 bp
cattttgatattcccagtatttgaagtatagaatcagtcaatATGTCCGACTCTGTCCCTACTAGTTCTTCGGTACCTTCATCTTCAGCAGTGT
CTCgtacgtgtaagtagttgatcttcaggcgcacgtttattccgaagggattatgcggtcatgcgaccggattttcctggtataacgtgaaatt
catcgatgcggatggttttaatttctctaccgcagattgatttattttttgtcatattgttctcgcgttttcgaagctgcgatcacatggactc
gctcttctacttcgacttgtgaagagttggactttcgataatccagatgaggactgactcgatcatagCTCAATTTGCCAAATACAAGGCTTGC
TTTTATGATCTTCTTACCTGGAAGAACCTTTATGTCTCTGGCGGTTCATTCGCTGGTGCATTGATTGCTCTTTACCTCTTCAAATATGTCAACG
TTGTGAAGCTATTCTTCAACTTTGCTTATATCGCCTTGGGCAgtatgtagacaatcaatggtaactgttggtatttggttatggattctaatga
gggtgaattatagCTGCTATTGCTGTCGAATTTGCTGGACGAACAATCAAGGGCGGTCCTGGTTTCGTTAGgtgtgtttcctcggtttggattc
cagttagaaactgcaaactaacgtaattgtgaagCTCTTTCCGTGGAAGTGGTAACTACTTCGTCATTTCAAAGGATATTGTCGATCCGCTCTT
CAATGAGTTCACTGTTCTCGTAAACTTTTTGCTGGTTGAGTTCCAGCAGATTGTCTTTGTTGAGAACTTTCCATTGACAGTCTTTgtaagctat
tcatgtcgttaggtttggctagctgtcgcgagattaataattggtttcttagGCATTTGTTGTCTCGTATTTCACTTATTTCCTCGTCCACTAC
GTTTCGCTGTGGTCTTTAGCTGTCGTTGgtacgtcgatgcaagctgcccaactactatggctagatggaaaaactaatagagttagGTGTCACT
GTGGCTTTCGCAGCACCGCCAGTCTACCTCAAGTTCCAGGAAGAGATTGATGCCCAGGTTGCTGCCGCTAACAAGATCATTGACAAGAAGATTA
GTGAGCTTAAGGTGCAGGCTAATGAGCATTTGGGCCAGGCCGCAGGTATCGCTAAAGGATATTTCGACCAAGCCCTCGACAAGGTCGGATACAA
GCGAAATCTTCCTCCGGTCCCAGCGGCTGAGCCTGTTGCTACTGAGCCTTCTCCCCCTGCAGTGCCTGTTGCTGCAGAGTAAggtgctg

>MN689086[CCY-33-1-1_RTNA_LONG][Lipomyces starkeyi][strain=CCY 33-1-1] reticulon-like (rtnA) long mRNA, complete CDS, 823 bp
cattttgatattcccagtatttgaagtatagaatcagtcaATATGTCCGACTCTGTCCCTACTAGTTCTTCGGTACCTTCATCTTCAGCAGTGT
CTCGTACGTCTCAATTTGCCAAATACAAGGCTTGCTTTTATGATCTTCTTACCTGGAAGAACCTTTATGTCTCTGGCGGTTCATTCGCTGGTGC
ATTGATTGCTCTTTACCTCTTCAAATATGTCAACGTTGTGAAGCTATTCTTCAACTTTGCTTATATCGCCTTGGGCACTGCTATTGCTGTCGAA
TTTGCTGGACGAACAATCAAGGGCGGTCCTGGTTTCGTTAGCTCTTTCCGTGGAAGTGGTAACTACTTCGTCATTTCAAAGGATATTGTCGATC
CGCTCTTCAATGAGTTCACTGTTCTCGTAAACTTTTTGCTGGTTGAGTTCCAGCAGATTGTCTTTGTTGAGAACTTTCCATTGACAGTCTTTGC

ATTTGTTGTCTCGTATTTCACTTATTTCCTCGTCCACTACGTTTCGCTGTGGTCTTTAGCTGTCGTTGGTGTCACTGTGGCTTTCGCAGCACCG
CCAGTCTACCTCAAGTTCCAGGAAGAGATTGATGCCCAGGTTGCTGCCGCTAACAAGATCATTGACAAGAAGATTAGTGAGCTTAAGGTGCAGG
CTAATGAGCATTTGGGCCAGGCCGCAGGTATCGCTAAAGGATATTTCGACCAAGCCCTCGACAAGGTCGGATACAAGCGAAATCTTCCTCCGGT
CCCAGCGGCTGAGCCTGTTGCTACTGAGCCTTCTCCCCCTGCAGTGCCTGTTGCTGCAGAGTAAggtgctg

>MN689087**[**CCY-33-1-1_CDNA_SHORT**]**[Lipomyces starkeyi][strain=CCY 33-1-1] reticulon-like (rtnA) short mRNA, complete CDS, 817 bp
catttgatattcccagtatttgaagtatagaatcagtcaatATGTCCGACTCTGTCCCTACTAGTTCTTCGGTACCTTCATCTTCAGCAGTGT
CTCCTCAATTTGCCAAATACAAGGCTTGCTTTTATGATCTTCTTACCTGGAAGAACCTTTATGTCTCTGGCGGTTCATTCGCTGGTGCATTGAT
TGCTCTTTACCTCTTCAAATATGTCAACGTTGTGAAGCTATTCTTCAACTTTGCTTATATCGCCTTGGGCACTGCTATTGCTGTCGAATTTGCT
GGACGAACAATCAAGGGCGGTCCTGGTTTCGTTAGCTCTTTCCGTGGAAGTGGTAACTACTTCGTCATTTCAAAGGATATTGTCGATCCGCTCT
TCAATGAGTTCACTGTTCTCGTAAACTTTTTGCTGGTTGAGTTCCAGCAGATTGTCTTTGTTGAGAACTTTCCATTGACAGTCTTTGCATTTGT
TGTCTCGTATTTCACTTATTTCCTCGTCCACTACGTTTCGCTGTGGTCTTTAGCTGTCGTTGGTGTCACTGTGGCTTTCGCAGCACCGCCAGTC
TACCTCAAGTTCCAGGAAGAGATTGATGCCCAGGTTGCTGCCGCTAACAAGATCATTGACAAGAAGATTAGTGAGCTTAAGGTGCAGGCTAATG
AGCATTTGGGCCAGGCCGCAGGTATCGCTAAAGGATATTTCGACCAAGCCCTCGACAAGGTCGGATACAAGCGAAATCTTCCTCCGGTCCCAGC
GGCTGAGCCTGTTGCTACTGAGCCTTCTCCCCCTGCAGTGCCTGTTGCTGCAGAGTAAggtgctg

>MN689088[CCY-33-1-1_RTNA_SPLINTER][Lipomyces starkeyi][strain=CCY 33-1-1] reticulon-like (rtnA) precursor RNA, partial CDS, 206 bp
catttgatattcccagtatttgaagtatagaatcagtcaatATGTCCGACTCTGTCCCTACTAGTTCTTCGGTACCTTCATCTTCAGCAGTGT
CTCgtacgtattgatttatttttgtcatattgttctcgcgttttcgaagctgcgatcacatggactcgctcttctacttcgacttgtgaagagt
tggactttcgataatcca

Lipomyces kononenkoae

>MN689089[LIPKON_RTNA_CDNA_SHORT][Lipomyces kononenkoae][strain=CBS 2514] reticulon-like (rtnA) mRNA, complete CDS, 839 bp
cctcactatccaccaaaacatagctgaacgtctaccattttgacaataacaaagtctagaatcagtcaatATGTCCGACTCTATCCCGTCTACA
TCTTCCGTAACGCCGTCATCATCGGTGTCACCTCAATTTACGAAATACAAGGCTTGCTTTTATGATCTTCTTACCTGGAAGAATCTTTACTTCT
CTGGTGGTGCATTCGCTGGCACTCTTGTTGCTCTTTATCTCTTTAAATACGTCAACGTTGTGAAGCTCCTTTTCAACTTTGCTTACATCGCCTT
GGGTACTGCTATTGCTGTTGAGTTTGCTGGCCGTACCATCAAGGGCGGTCCAGGCTTCGTTAGCTCTTTCCGTGGAAGTGGCAACTACTTTGTC
ATTTCGAAGGAAGTTGTCGATCCTCTCTTCAACGAGTTCACCGTTCTTGTCAACTTTTTGCTCATTGAGTTCCAACAGATTGTTTTCGTTGAGA
ACTTTAATTTAACTGTCTTCGCATTCGTTGTGTCGTACTTCACATATATTCTCGTCCACTATGTCTCTCTATGGTCTTTGACCGTCTTTGGTGT
TATTGTGTCATTCTCGGCTCCGCCAATCTACATTAAGTTTCAGAAAGAGATTGATGCGCAAGTTGCCGTAGCCACCAAAATTGTTGACGAGAAG
ACTGGCGAGCTGAAGGTGCTTGCCAATGAGTATTTCGGCAAGAGCGCGGGCATCGCGAAGGGATATGTAAACGAAGCTCTCGACAAGGTTGGAT
ACAAGCGAAATCTTCCTCCGGTCCCCGTGGTCGAGCCTACAGCTACTGAGCCTGCTCCTAAGACGGTGCCTGTAGCAGCCGAGTAAa

>MN689090[LIPKON_RTNA_SPLINTER][Lipomyces kononenkoae][strain=CBS 2514] reticulon-like (rtnA) precursor RNA, partial CDS, 239 bp
cctcactatccaccaaaacatagctgaacgtctaccattttgacaataacaaagtctagaatcagtcaatATGTCCGACTCTATCCCGTCTACA
TCTTCCGTAACGCCGTCATCATCGGTGTCACgtacgtatggattattttggtgatgtacttgttgtgtgcttgagaaatgtgatggggcaacgc
aatcacatgaactcgatccgcttggaattaacaagttgcgaatattctata
